# Long-term clinical outcomes of delirium after hospital discharge: a systematic review and meta-analysis

**DOI:** 10.1093/ageing/afaf188

**Published:** 2025-07-08

**Authors:** Yonas Tesfaye, Courtney R Davis, Melissa J Hull, Danielle Greaves, James du Preez, Sally Johns, Alice Bourke, Hannah A D Keage

**Affiliations:** Justice and Society, University of South Australia, Adelaide, Australia; Justice and Society, University of South Australia, Adelaide, Australia; Clinical and Health Sciences, University of South Australia, Adelaide, Australia; Clinical and Health Sciences, University of South Australia, Adelaide, Australia; UniSA Online, University of South Australia, Adelaide, Australia; Clinical and Health Sciences, University of South Australia, Adelaide, Australia; UniSA Online, University of South Australia, Adelaide, Australia; Faculty of Science, Medicine and Health, University of Wollongong, Wollongong, Australia; Northern Adelaide Local Health Network, Adelaide, Australia; Northern Adelaide Local Health Network, Adelaide, Australia; Justice and Society, University of South Australia, Adelaide, Australia

**Keywords:** delirium, older adults, clinical outcomes, hospital discharge, meta-analysis, systematic review

## Abstract

**Background:**

Delirium has been linked to adverse health outcomes. There has not been a comprehensive attempt to synthesise these outcomes.

**Objective:**

To synthesise evidence comparing post-discharge clinical outcomes in individuals who experienced delirium in hospital compared to those who did not.

**Methods:**

A systematic electronic search was conducted in Medline, Embase, CINAHL, PsycINFO and Cochrane databases. Random-effects models were used to assess effect size differences between those who experienced delirium and those who did not: odds ratios (OR) for categorical outcomes and Hedges' g for continuous outcomes. Analyses were conducted for each outcome relative to ≤6 months, >6–12 months, 12+ months and collapsed across time post-discharge.

**Results:**

Data were synthesised from 253 studies representing 29 814 participants who experienced delirium and 107 583 participants who did not experience delirium. The mean (SD) age of participants was 76.0 (9.3) years. Collapsed over follow-up period, results included, those who experienced delirium in hospital showed higher objective cognitive decline (OR = 1.58, *P* < .001), greater subjective cognitive impairment (OR = 2.11, *P = .*041), greater functional decline (g = −0.43, *P = .*001), lower quality of life (g = −0.44, *P* < .001), higher burden of poor mental health (OR = 1.69, *P* < .001), increased risk of dementia (OR = 5.37, *P* < .001), higher likelihood of institutionalisation (OR = 2.80, *P* < .001), greater rates of hospital readmission (OR = 1.70, *P* < .001) and increased mortality (OR = 2.55, *P* < .001) post-hospital discharge compared to those who did not experience delirium in hospital. Time-specific analyses did not reveal any consistent patterns of effects.

**Conclusions:**

Older adults who experience delirium in hospital demonstrate significantly worse long-term clinical outcomes compared to those who do not.

## Key Points

- Those who have delirium in hospital demonstrate worse clinical outcomes across the first-year post-discharge and beyond, as compared to those who do not experience delirium in hospital. The largest effect was on dementia; delirium increased the odds of incident dementia by 5.4 times.
- Medium effects were seen institutionalisation, mortality, objective cognitive performance, functional performance and quality of life.
- Small but notable effects were observed for subjective cognitive performance, functional and cognitive change, readmission and mental health.

## Introduction

Delirium is an acute, fluctuating neuropsychiatric syndrome characterised by disturbances in attention, awareness and other cognitive functions [1]. While common, delirium is under-recognised, with debate around its pathophysiology and a lack of effective treatments [2–4].

Delirium arises from complex and multifactorial causes [2], frequently linked to the pathophysiological impacts of acute medical conditions, medication effects or dementia [5]. while delirium can affect individuals at any age, older adults are at increased risk likely due to age-related structural and functional changes in the brain that diminish reserve [6, 7]. Prevalence rates of delirium vary widely across patient groups and settings [2, 8, 9], ranging from less than 0.5% in the community [10] to as high as 88% among palliative care patients [11]. Delirium is particularly common in hospitalised older adults, affecting up to 60% [5, 12].

Several studies across various settings have reported that patients who experience delirium, as compared to those who do not, face a higher risk of future cognitive impairment [13, 14], dementia [15, 16], functional impairment [17, 18], readmission [19, 20], institutionalisation [21, 22], mortality [23], poor mental health [24, 25] and reduced quality of life (QoL) [26, 27]. Meta-analyses have tried to tackle these long-term outcomes of delirium, but have only assessed one or a few clinical outcomes [14, 23, 28], a specific clinical population [28] or have not examined the outcomes across different time-periods [14, 23, 28, 29].

It is common to hear those in clinical practice comment that ‘delirium is associated with all poor outcomes in older people’, yet no paper has comprehensively tried to report on this. In this review, we aimed to synthesise evidence of all clinical outcomes across time, comparing those who experienced delirium with those who did not experience delirium in hospital, after their discharge.

## Methods

The study protocol was registered on PROSPERO (Table S1) prior to data extraction [30] and conducted in accordance with the PRISMA 2020 statement (Tables S2 and S3, Supplementary Appendix).

### Search strategy and selection criteria

A systematic electronic search was conducted in MEDLINE, Embase and PsycINFO via Ovid, as well as in the CINAHL and Cochrane databases from January 1980 to November 27, 2023. The search was limited to studies published from 1980 onward, reflecting the year delirium was formally established as a diagnostic term in the Diagnostic and Statistical Manual of Mental Disorders (DSM) [23, 31, 32]. The search strategy focused on the terms *(delirium AND (prospective OR longitudinal))*. Other terms related to delirium, such as confusion, encephalopathy or Intensive Care Unit (ICU) psychosis, were not included, as these conditions, while sharing some features, represent distinct diagnostic concepts [33]. The references of included articles were manually reviewed to identify additional studies. After duplicate records were removed, three study authors (YT, CD and JDP) screened the remaining articles by title and abstract, followed by full text. The article screening process was managed using Covidence software [34]. Disagreements during the screening and data extraction process were resolved through discussion and consensus or by consulting another researcher (HADK, DG or MH).

We included peer-reviewed primary studies of older adults with a group mean or median age of 60 years or above at baseline, published in English after 1980 and reporting at least one clinical outcome. Clinical outcomes were defined as any conditions or measures captured in relation to delirium, including but not limited to cognition, QoL, mortality and institutionalisation. Eligible studies were longitudinal quantitative studies that reported an association between past delirium (versus its absence) and clinical outcome(s) as either inferential or descriptive statistics. Our focus was on clinical outcomes after delirium resolution; however, most studies did not report delirium prevalence at follow-up, and we did not exclude studies on these grounds. Where delirium was explicitly reported as still present at follow-up in any participants, the study was excluded.

Studies systematically identifying delirium (all participants needed to be assessed for delirium in the same way within a study) in hospitalised older adults using validated tools or criteria were included. We excluded studies where delirium was not systematically assessed, which were typically retrospective designs using clinical records, as these methods are known to underestimate delirium prevalence [35–37]. Additionally, we excluded studies on terminally ill patients (as we wanted to assess long-term outcomes), unresolved delirium, studies focused solely on in-hospital outcomes, and studies where outcomes were not compared between those who did and did not experience delirium. If studies did not report relevant data and attempts to contact authors failed, or if the delirium and no-delirium groups were not otherwise homogenous (e.g. delirium+dementia group compared to no-delirium+no-dementia group), they were excluded. We further excluded duplicate samples, case series, reviews, conference abstracts, editorials, dissertations, non-English articles and publications without full text. Composite outcomes (e.g. functional impairment or death) were excluded.

### Data extraction

Relevant data were extracted by YT into a template Excel sheet and checked for errors by CD. Extracted information included the first author’s name, country, study design, setting of delirium ascertainment, age, sex, sample size, delirium measurement tool/s, types of clinical outcomes, follow-up duration and inferential or descriptive data on clinical outcomes in delirium and without delirium groups. Disagreements were resolved through discussion and consensus.

Objectively measured (through standardised cognitive test administration) and subjectively measured (self-reported surveys and/or opinions) cognitive outcomes were analysed separately. Both functional and cognitive outcomes were then categorised as follows: change and performance, each divided into dichotomous or continuous outcomes. Change was defined as a within-group change in scores from baseline to follow-up. This could be presented as a dichotomous variable (referred to here as cognitive decline) or a continuous variable (e.g. mean and SD scores compared across times). Performance referred to a cross-sectional evaluation of the outcome at follow-up only, without considering baseline data. Dichotomous performance outcomes reflected a threshold for impairment either reached or not reached (referred to here as cognitive impairment), and continuous performance outcomes reflected absolute performance on a test at follow-up.

For categorical outcomes, preference was given to covariate-adjusted odds ratios (ORs) with 95% confidence intervals (most frequently reported type). Similarly, for continuous outcomes, preference was given to covariate-adjusted data. When studies reported Hazard Ratios or Relative Risks instead of OR, crosstab data were extracted. Where available, adjusted estimates (multivariable) were prioritised given the potential for residual confounders in the context of dementia and ageing. If crosstab or OR data were not available (after attempting to contact authors if necessary), studies were excluded.

### Study quality and certainty of evidence

The quality of included studies for each outcome was assessed by YT and checked by CD using the Newcastle-Ottawa Scale for cohort studies [38, 39]. One randomised controlled trial was included, however was treated as a prospective cohort study for the purposes of this review. The Newcastle-Ottawa Scale criteria were applied as per guideline [40] (Table S16). The overall risk of bias evaluation can be interpreted as high (≤ four points), moderate, (five or six points) or low (≥ seven points) [41].

The certainty of evidence was evaluated using the Grading of Recommendations, Assessment, Development and Evaluation (GRADE) framework (Table S17) [42, 43].

### Data analysis

Meta-analyses of each clinical outcome were performed using Comprehensive Meta-Analysis (CMA) software (version 4, Biostat, New Jersey, USA) [44]. Random-effects models were used to calculate the pooled effect size differences between those who did and did not experience delirium in hospital, reported in two or more studies. Alpha was set at <0.05.

We analysed dichotomous and continuous data separately [45]. Both time-collapsed analyses (clinical outcomes regardless of time) and time-specific analyses (≤6 months, >6–12 months and > 12 months) were presented when data were available (n ≥ 2 studies). Studies lacking specific time points or reporting only average follow-up times were only included in time-collapsed analyses. We used baseline sample sizes to calculate the effect sizes when the included study did not report follow-up sample size.

For all outcomes, preference was given to covariate-adjusted data. Differences in continuous outcomes between delirium and no delirium groups were summarised using Hedges’ g. Hedges' g were calculated by transforming medians, interquartile ranges (IQRs) or confidence intervals into means and standard deviations (SDs) [46–48]. Standardised beta coefficients were converted to Hedges' g using the Campbell Collaboration calculator [49]. Hedges' g effect sizes were interpreted as follows: 0.2 (small effect), 0.5 (medium effect) and 0.8 (large effect) [22]. Negative Hedges' g values indicated worse outcomes in the delirium compared to the no delirium group. An OR of >1 indicates higher odds of experiencing the clinical outcome in patients who experienced delirium as compared to no-delirium group. We considered an OR of around 1.5 = small effect (or weak association), 2.5 = medium (or moderate), 4.0 = large (or strong) and 10.0 = very large (or very strong) [50].

Between-study heterogeneity was assessed by *tau^2^* and the proportion of observed between-study variance using I^2^statistics (low: 25%, moderate: 50% and high: 75%) [51, 52]. For significant heterogeneity, subgroup and sensitivity analyses were conducted by excluding outliers. Additionally, small study (publication bias) was assessed visually through funnel plots and by conducting Egger's intercept tests when at least 10 studies were available. The Duval and Tweedie trim-and-fill method was used to estimate the magnitude of potential bias when funnel plot asymmetry was detected (*P* < .1) [53]. For analyses with fewer than 10 studies, sensitivity analyses were conducted by removing outliers (Table S15).

We conducted sensitivity analyses to assess the robustness of the main findings by comparing (i) adjusted versus unadjusted estimates and (ii) studies who did and did not (i.e. baseline sample size used) report follow-up sample sizes. Additionally, we performed subgroup analyses based on follow-up duration and specific outcome categories, including functional outcomes (by excluding frailty, fall and fatigue outcomes from other functional outcome measures such as ADL and IADL), mental health (including depression, anxiety, post-traumatic stress disorder and sleep disturbances) and QoL (components of the QoL dimension measure), to evaluate whether effect sizes differed across categories (Tables S10–S14).

## Results

### Study characteristics

**Figure 1:**
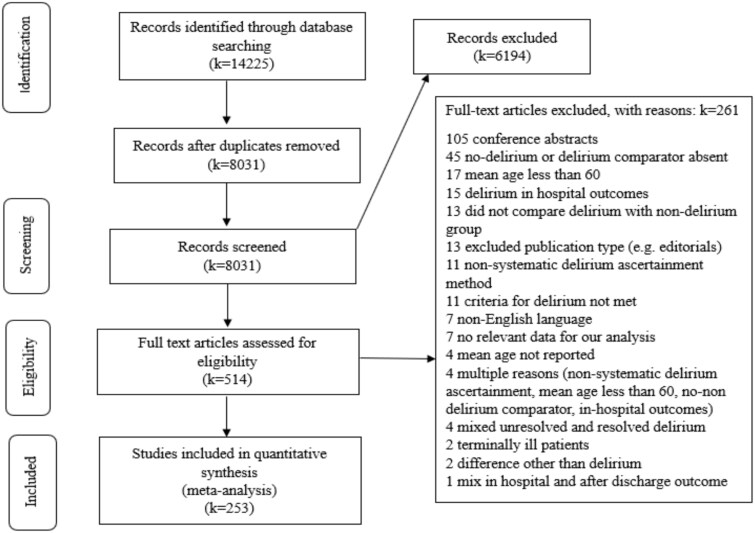
Preferred Reporting Items for Systematic Reviews and Meta-Analysis flow diagram (PRISMA).

The database search identified 14 225 records, of which 6194 duplicates were excluded, leaving 8031 articles for title and abstract screening. Among the 514 full-text articles reviewed, 253 studies met the inclusion criteria and were included in the quantitative synthesis (Figure 1).

The baseline cohort comprised 36 489 individuals who experienced delirium in hospital and 160 902 who did not, and the follow-up cohort included 29 814 who had previously experienced delirium in hospital and 107 583 who had not (Table S4 and S6).

Complete baseline sex data were reported in 243 studies (excluding one study due to reporting errors), with 81 620 males (42.0%) and 112 491 females (58.0%). The mean (SD) age of participants was 76.0 (9.3) years, with a range from 60.0 to 89.4 years (Table S5). The duration of follow-up ranged from 2 days post-hospital discharge to 14 years.

The studies were conducted across 38 countries, with 63 studies (24.8%) from the United States, 28 (11.0%) from the Netherlands, and 22 (8.7%) from the United Kingdom. Four studies were conducted in multiple countries. Over half of the studies (k = 129, 51.0%) were conducted in Europe, followed by North America (k = 71, 28.0%), and Asia (k = 30, 11.5%). Three studies spanned two continents (Europe and North America). Almost all studies (k = 241, 95.3%) were prospective cohort designs. Nearly a third (k = 81, 31.9%) involved surgical patients, followed by ICU patients (k = 36, 14.2%).

Delirium was assessed using versions of the Confusion Assessment Method and/or the DSM in 243 studies (96.0%). The included studies were published between 1990 and 2023, with most (k = 133, 52.6%) published between 2010 and 2019, and 75 studies (29.6%) published in 2020 or later.

### Clinical outcomes comparing those who did and did not experience delirium in hospital

**Figure 2:**
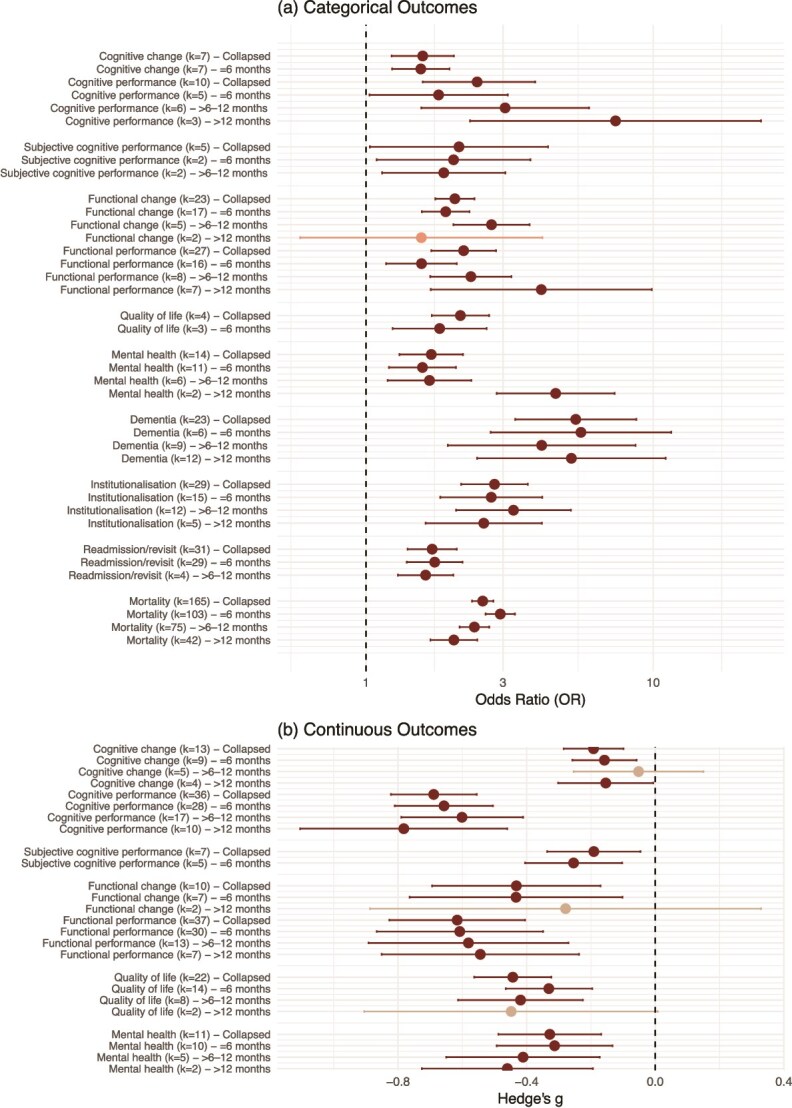
Results of time-collapsed and time-specific analyses for (a) categorical and (b) continuous clinical outcomes (dark = statistically significant, light = not statistically significant).

Complete results of all outcomes, see Figure 2, Table S7, Table S8 and Table S9.

### Objective cognitive performance and change

In 36 studies, those who experienced delirium in hospital demonstrated lower cognitive performance across all timepoints post-discharge, compared to those who did not, with a medium effect (g = −0.69, *P* < .001). Effect sizes were similar across time-specific analyses, with those who experienced delirium in hospital displaying poorer cognitive performance compared to those who did not at ≤6 months (g = −0.66, *P* < .001), >6–12 months (g = −0.60, *P* < .001) and > 12 months (g = −0.78, *P* < .001). Similarly, cognitive impairment (k = 10) was higher among the delirium group collapsed across follow-ups (OR = 2.47, *P* < .001), and in time-specific analyses: ≤6 months (OR = 1.79, *P = .*040), >6–12 months (OR = 3.05, *P = .*001) and > 12 months (OR = 7.40, *P = .*001).

A small but significant difference in cognitive change was observed in those who experienced delirium compared to those who did not (g = −0.19, *P* < .001). This effect was consistent at ≤6 months (g = −0.16, *P = .*002) and > 12 months (g = −0.15, *P = .*042), but not significant at >6–12 months (g = −0.05, *P = .*613). The cognitive decline effect was small (OR = 1.57, *P* < .001), with more decline in those who experienced delirium compared to those who did not. This effect was similar at ≤6 months (OR = 1.55, *P* < .001).

### Subjective cognitive performance

Delirium was associated with poorer subjective cognitive performance across seven studies (g = −0.19, *P = .*010). A similar effect was found at ≤6 months (g = −0.25, *P = .*001). Similarly, subjective cognitive impairment was more prevalent in those who experienced delirium (k = 5, OR = 2.11, *P = .*041). This effect was consistent in the time-specific analyses: ≤ 6 months (OR = 2.02, *P = .*026) and > 6–12 months (OR = 1.86, *P = .*013).

### Functional performance and change

Collapsed across timepoints in 37 studies, functional performance was poorer in the delirium group (g = −0.62, *P* < .001). Medium effects were observed across time: ≤6 months (g = −0.61, *P* < .001), >6–12 months (g = −0.58, *P* < .001) and > 12 months (g = −0.54, *P = .*001). A medium effect was also seen for functional impairment after delirium compared to no delirium across 27 studies (OR = 2.19, *P* < .001). In the time-specific analysis, similar effect sizes were observed at ≤6 months (OR = 1.56, *P* < .002), >6–12 months (OR = 2.32, *P* < .001), and was larger after 12 months (OR = 4.08, *P = .*002).

A medium effect size was observed for functional change in those who experienced delirium compared to those who did not across 10 studies (g = −0.43, *P = .*001). Similar effects were seen at ≤6 months (g = −0.43, *P* < .001). However, at >12 months (k = 2), the effect was no longer statistically significant (g = −0.28, *P = .*368). Functional decline as a dichotomous variable was higher in individuals who experienced delirium than in those who did not, across timepoints in 23 studies (OR = 2.04, *P* < .001). Results were relatively consistent across time: ≤6 months (OR = 1.89, *P* < .001), >6–12 months (OR = 2.73, *P* < .001), >12 months (1.56, *P* < .001).

### QoL

Those who experienced delirium had poorer QoL compared to those who did not across follow-up timepoints (k = 22 studies) (g = −0.44, *P* < .001). Effect sizes were small to medium in the time-specific analyses: ≤6 months (g = −0.33, *P* < .001) and > 6–12 months (g = −0.42, *P* < .001). The difference between groups was not significant at >12 months (g = −0.45, *P = .*054), likely due to low study number (k = 2). In the subgroup analysis, the QoL dimension of physical functioning (g = −0.62, *P* < .001) and social functioning (g = −0.59, *P* < .001) had medium effect sizes, with those who experienced delirium having lower QoL; vitality and general health were also significant but had small effect sizes (Table S8).

### Mental health

In the time-collapsed analyses for poor mental health, there was a small to medium effect in those who experienced delirium compared to those who did not (g = −0.33, *P* < .001). Time-specific analyses showed those who experienced delirium had worse mental health than those who did not at ≤6 months (g = −0.31, *P = .*001), >6–12 months (g = −0.41, *P = .*001) and > 12 months (g = −0.46, *P = .*001). Subgroup analysis showed a small to medium effect size, with higher odds of sleep disturbances (OR = 2.72, *P = .*030), depression (OR = 1.66, *P* ≤ .001), anxiety (OR = 1.60, *P = .*001) and post-traumatic stress disorder (OR = 1.41, *P = .*010) in those who experienced delirium than those who did not (Table S7).

### Dementia

In the time-collapsed analyses, the effect size for risk of incident dementia was large in those who experienced delirium compared to those who did not across 23 studies (OR = 5.37, *P* < .001). In time-specific analyses, those who experienced delirium had higher odds of incident dementia than those who did not at ≤6 months (OR = 5.60, *P* < .001), >6–12 months (OR = 4.09, *P* < .001), >12 months (OR = 5.19, *P* < .001) follow-up.

### Institutionalisation

The odds of institutionalisation were significantly higher, with a medium effect size, in those who experienced delirium compared to those who did not across follow-up timepoints (k = 29, OR = 2.80, *P* < .001). Results were relatively consistent across time: ≤6 months (OR = 2.73, *P* < .001), >6–12 months (OR = 3.26, *P* < .001), >12 months (OR = 2.57, *P* < .001).

### Readmission/revisit

The likelihood of readmission or revisit was higher in those who experienced delirium compared to those who did not across 31 studies and follow-up periods (OR = 1.70, *P* < .001). In time-specific analyses, those who experienced delirium had higher odds of readmission or revisit than those who did not at ≤6 months (OR = 1.73, *P* < .001), and > 6–12 months (OR = 1.61, *P* < .001).

### Mortality

For mortality there was a medium effect (OR = 2.55, *P* < .001) for delirium versus no delirium in the time-collapsed analysis across 165 studies. In time-specific analyses, those who experienced delirium had higher odds of death than those who did not at ≤6 months (OR = 2.93, *P* < .001), >6–12 months (OR = 2.38, *P* < .001), >12 months (OR = 2.02, *P* < .001).

### Additional analyses

No notable differences were observed in subgroup analyses based on follow-up duration or subcategories of clinical outcomes (functional outcomes, mental health and quality of life), full results in Supplementary Material (Subgroup analyses section). The overall risk of bias across outcomes ranged from low to moderate. Most outcomes, except for QoL and mental health, demonstrated a low risk of bias. No studies were excluded from the analysis based on quality (Table S16).

All included outcomes began the GRADE process with a low certainty of evidence due to reliance on data from observational studies [54]. We have moderate confidence in the effect estimates (Table S17).

## Discussion

In this large and comprehensive meta-analysis there was substantial evidence for delirium having adverse effects on all clinical outcomes across the first-year post-discharge and beyond. The largest effect was incident dementia, but medium effects were seen institutionalisation, mortality, objective cognitive performance, functional performance and QoL. Small but notable effects were observed for subjective cognitive performance, functional and cognitive change, readmission and mental health.

This was the first study to meta-analyse QoL, functional status, mental health and readmission outcomes in those who had previously experienced delirium in hospital. Factors such as perceived loss of autonomy, feelings of dependency on caregivers, a sense of rejection due to caregiver burden, social isolation and loneliness may contribute to poor mental health and QoL outcomes [55–59]. Readmission risk was almost twice as high in the first 6 months, suggesting that delirium may interfere with recovery from the original acute insult. Additionally, having a more severe condition in hospital, comorbidities, the compounding effects of functional decline, cognitive decline or poor mental health could lead to further hospitalisations [5, 60].

Delirium increased the odds of dementia by 5.4 times, a lower estimate than the most recent previous meta-analyses from 2021, which reported ORs of 11.9 [61] and 6.1 [13] for dementia. This difference may be due to variations in studies inclusion criteria such as age thresholds (≥60 vs. ≥65 years), setting (hospital inpatients vs. both community and hospital cases) and case types (surgical patients vs. a mix of surgical and non-surgical cases). Risk of institutionalisation was almost three times higher for the delirium group, similar to a 2010 meta-analysis (OR = 2.4) [29]. Our findings for mortality (OR = 2.5) also align well with previous meta-analyses conducted in 2010 (OR = 1.6) [29], 2017 (OR = 4.1) [63], and most recently 2020 (OR = 2.5–3.2) [23, 64] and 2023 (OR = 6.6) [62] . In concordance with an increased risk of dementia, cognitive impairment and decline risks were also higher after delirium, consistent with older meta-analysis findings from 2020 (g = −0.45) [14] and 2021, where short-term effects (g = −0.46), and long-term effects (g = −0.82) [13] were noted.

Delirium is clearly having a negative effect on all areas of health. This range of poor outcomes drives the higher healthcare costs, including greater impact on the primary and tertiary health care systems [65]. Multiple bi-directional relationships are possible between outcomes, e.g. function and mental health, and it is likely that many cascading processes underlie this deterioration observed post-delirium. Experiencing delirium increases the risk of future episodes, meaning that some study participants will have experienced delirium prior or after what we have determined the in-hospital period to be [66]. These multiple insults to the brain could have a compounding effect [15, 67], and it is likely that delirium trigger a cognitive decline process, at least in some. The neurocognitive impact of delirium, comorbid health conditions and limited support systems and rehabilitation resources exacerbate decline [56–59, 68, 69]. Future studies focusing on the brain through structural and physiological measures could provide more valuable insights and strengthen the evidence for the interrelationship between delirium and the risk of clinical outcomes, particularly dementia and cognition. Given delirium is associated with a substantial collection of poor outcomes for older adults, and our time-based analysis suggests intervening in the first 12 months with a multi-disciplinary approach to address clinical areas such as mental health, physical health (nutrition, physical activity), sleep hygiene and cognition is very likely to be beneficial [70]. Programs such as Maintain Your Brain [71, 72] and FINGER [73] used multi-modal interventions addressing physical activity, social interaction, diet etc. and showed improvements in brain health.

This is a large and comprehensive meta-analysis, and updates older meta-analyses looking at single or a few outcomes; and includes functional, mental health, QoL and readmission outcomes, which have not been previously meta-analysed. This study is however, not without limitations. First, the possibility that some unresolved cases of delirium were included. Most of the studies did not report whether delirium was absent at discharge or whether delirium was (again) present at follow-up. We excluded studies that clearly described unresolved delirium or delirium recurrence during follow-up, as well as studies on persistent delirium. However, there is good evidence that delirium symptoms can persist for several months after discharge in a very small percentage [74–76]. Second, for studies that measured absolute performance post-discharge, not relative to pre-hospital function, we cannot rule out that group differences pre-hospitalisation drove group differences. Given effects were larger for performance as compared to change (decline or incident impairment), and that lower cognition and function are known delirium risk factors, this is likely. Third, we could not account for a survivor effect in our analyses, with later estimates reflecting the outcomes in those who were likely healthier at study baselines due to drop-out due to attrition and death. Fourth, minor changes in the delirium diagnostic criteria from DSM-III to DSM-V-TR could have impacted the findings of this study. Finally, only studies published in English were included due to resource constraints in translation.

## Conclusion

Our results highlight older adults who experienced delirium have much worse clinical outcomes than those who did not experience delirium, including cognitive and functional decline, higher odds of readmission, institutionalisation, dementia, mortality, reduced QoL and mental health problems. This underscores the importance of not only delirium prevention, but post-discharge clinical follow-up and the urgent need for effective, holistic and tailored rehabilitation interventions specifically targeting this vulnerable population.

## Supplementary Material

Supplementary_materials_afaf188

## Data Availability

Supplementary data mentioned in the text are available in Age and Ageing online. The full list of references is available in the Supplementary data.
