# Correction to: Long-term clinical outcomes of delirium after hospital discharge: a systematic review and meta-analysis

**DOI:** 10.1093/ageing/afaf229

**Published:** 2025-08-10

This is a correction to: Yonas Tesfaye, Courtney R Davis, Melissa J Hull, Danielle Greaves, James du Preez, Sally Johns, Alice Bourke, Hannah A D Keage, Long-term clinical outcomes of delirium after hospital discharge: a systematic review and meta-analysis, *Age and Ageing*, Volume 54, Issue 7, July 2025, afaf188, https://doi.org/10.1093/ageing/afaf188

In the originally published version of this manuscript, references 50 to 76 were incorrectly numbered as 51 to 77 in the supplementary file.

The supplementary file has been corrected and citations to references 50 to 76 in the main article text have been renumbered for accuracy.

