# Supplementary material for: Long-term clinical outcomes of delirium after hospital discharge: a systematic review and meta-analysis
Source: Age Ageing. 2025 Jul 8;54(7):afaf188. doi: 10.1093/ageing/afaf188 (PMC12236429; doi:10.1093/ageing/afaf188)
Supplement: Supplementary_materials_afaf188 [file supplementary_materials_afaf188.docx]

**SUPPLEMENTAL MATERIAL**

Long-term clinical outcomes of delirium after hospital discharge: a systematic review and meta-analysis

# Table of Contents

Contents

[Table of Contents 2](#_Toc197000097)

[Table S1. Glossary of abbreviations 2](#_Toc197000098)

[Table S2. PRISMA 2020 Main Checklist 4](#_Toc197000099)

[Table S3. PRISMA 2020 for Abstracts Checklist 7](#_Toc197000100)

[Search strategy 8](#_Toc197000101)

[Table S4. Overview of included studies (sample size and time specific clinical outcomes) 8](#_Toc197000102)

[Table S5. Overview of included studies and their demographic characteristics 18](#_Toc197000103)

[Table S6. Summary of included studies on long-term clinical outcomes of delirium after hospital discharge 28](#_Toc197000104)

[Table S7. Follow-up time analysis for long-term clinical outcomes of delirium after hospital discharge: Categorical data results 29](#_Toc197000105)

[Table S8. Follow-up time analysis for long-term clinical outcomes of delirium after hospital discharge. Continuous data results 31](#_Toc197000106)

[Table S9. Forest plots for long-term clinical outcomes of delirium after hospital discharge 33](#_Toc197000107)

[Table S10. Covariates adjusted and unadjusted data analyses. Categorical data results 76](#_Toc197000108)

[Table S11. Covariates adjusted and unadjusted data sensitivity analyses. Continuous data results 78](#_Toc197000109)

[Table S12. Sample size sensitivity analysis. Continuous data results 80](#_Toc197000110)

[Table S13. Functional outcomes sensitivity analyses with and without (frailty, fatigue, and/or fall) 82](#_Toc197000111)

[Table S14. Outlier analyses for long-term clinical outcomes of delirium after hospital discharge 83](#_Toc197000112)

[Table S15. Small study effect /publication bias investigations 84](#_Toc197000113)

[Table S16. Risk of bias assessment 92](#_Toc197000114)

[Table S17. GRADE (Grading of Recommendations, Assessment, Development, and Evaluations) 105](#_Toc197000115)

[Manuscript References (51–77) 107](#_Toc197000116)

[References (Clinical outcomes used for analyses 1–253) 108](#_Toc197000117)

# Table S1. Glossary of abbreviations

| **Abbreviations/Acronyms** |  |
| --- | --- |
| AMT | Abbreviated Mental Test |
| 4AT | Arousal, Attention, Abbreviated Mental Test - 4 |
| ADL | Activities of Daily Living |
| bCAM | Brief Confusion Assessment Method |
| CABG | Coronary Artery Bypass Graft |
| CAC-A | Clinical Assessment of Confusion A |
| CAM | Confusion Assessment Scale |
| CAM-ICU | Confusion Assessment Scale- Intensive Care Unit |
| CAM-S | Instrument Confusion Assessment Method – Severity Scale |
| CAT | Categorical |
| CHART-DEL | Chart-Based Delirium Identification Instrument |
| CI | Confidence interval |
| CINAHL | Cumulative Index to Nursing and Allied Health Literature |
| CMA | Comprehensive Meta-Analysis Software |
| Con | Continuous |
| COVID-19 | Coronavirus disease-2019 |
| DOSS | Delirium Observation Screening Scale |
| DRS | Delirium Rating Scale |
| DRS-R-98 | Delirium Rating Scale-Revised-Version 98 |
| DSI | Delirium Symptom Interview |
| DSM | Diagnostic and Statistical Manual |
| DSM-III | Diagnostic and Statistical Manual- Version III |
| DSM-III-R | Diagnostic and Statistical Manual- Version III-Revised |
| DSM-IV | Diagnostic and Statistical Manual- Version IV |
| DSM-IV-TR | Diagnostic and Statistical Manual- Version IV-Text Revision |
| DSM-V | Diagnostic and Statistical Manual- Version V |
| ED | Emergency Department |
| g | Hedges's g |
| HR | Hazard Ratios |
| IADL | Instrumental Activities of Daily Living |
| I-AGeD | Informed Assessment of Geriatric Delirium |
| ICDSC | Intensive Care Delirium Screening Checklist |
| ICU | Intensive Care Unit |
| IQR | Interquartile Range |
| K | Number of studies |
| MDAS | Memorial Delirium Assessment Scale |
| NA | Not available |
| NEECHAM Confusion Scale | Neelon and Champagne Confusion Scale |
| Nu-DESC | Nursing Delirium Screening Scale |
| OBS | Organic Brain Syndrome Scale |
| OPRAA | Older Persons Routine Acute Assessment |
| OR | Odds Ratio |
| PRISMA | Preferred Reporting Items for Systematic Reviews and Meta-Analyses |
| PROSPERO | International prospective register of systematic reviews |
| PTSD | Posttraumatic Stress Disorder |
| RASS | Richmond Agitation-Sedation Scale |
| RCT | Randomized controlled trial |
| RR | Relative Risks |
| SD | Standard deviation |
| SICU | Surgical Intensive Care Unit |

PRISMA Checklist.

# Table S2. PRISMA 2020 Main Checklist

| **Section and Topic** | **Item #** | **Checklist item** | **Location where item is reported** |
| --- | --- | --- | --- |
| **TITLE** | | |  |
| Title | 1 | Identify the report as a systematic review. | Title page |
| **ABSTRACT** | | |  |
| Abstract | 2 | See the PRISMA 2020 for Abstracts checklist. |  |
| **INTRODUCTION** | | |  |
| Rationale | 3 | Describe the rationale for the review in the context of existing knowledge. | Introduction section |
| Objectives | 4 | Provide an explicit statement of the objective(s) or question(s) the review addresses. | Introduction section |
| **METHODS** | | |  |
| Eligibility criteria | 5 | Specify the inclusion and exclusion criteria for the review and how studies were grouped for the syntheses. | ‘Search strategy and selection criteria’ section |
| Information sources | 6 | Specify all databases, registers, websites, organisations, reference lists and other sources searched or consulted to identify studies. Specify the date when each source was last searched or consulted. | ‘Search strategy and selection criteria’ section |
| Search strategy | 7 | Present the full search strategies for all databases, registers and websites, including any filters and limits used. | ‘Search strategy and selection criteria’ section |
| Selection process | 8 | Specify the methods used to decide whether a study met the inclusion criteria of the review, including how many reviewers screened each record and each report retrieved, whether they worked independently, and if applicable, details of automation tools used in the process. | ‘Search strategy and selection criteria’ section |
| Data collection process | 9 | Specify the methods used to collect data from reports, including how many reviewers collected data from each report, whether they worked independently, any processes for obtaining or confirming data from study investigators, and if applicable, details of automation tools used in the process. | ‘Data extraction’ section |
| Data items | 10a | List and define all outcomes for which data were sought. Specify whether all results that were compatible with each outcome domain in each study were sought (e.g. for all measures, time points, analyses), and if not, the methods used to decide which results to collect. | ‘Data extraction’ and ‘Data analysis’ sections |
|  | 10b | List and define all other variables for which data were sought (e.g. participant and intervention characteristics, funding sources). Describe any assumptions made about any missing or unclear information. | ‘Data extraction’ and ‘Data analysis’ sections |
| Study risk of bias assessment | 11 | Specify the methods used to assess risk of bias in the included studies, including details of the tool(s) used, how many reviewers assessed each study and whether they worked independently, and if applicable, details of automation tools used in the process. | ‘Study quality and certainty of evidence’ section |
| Effect measures | 12 | Specify for each outcome the effect measure(s) (e.g. risk ratio, mean difference) used in the synthesis or presentation of results. |  |
| Synthesis methods | 13a | Describe the processes used to decide which studies were eligible for each synthesis (e.g. tabulating the study intervention characteristics and comparing against the planned groups for each synthesis (item #5)). | ‘Data analysis’ section |
|  | 13b | Describe any methods required to prepare the data for presentation or synthesis, such as handling of missing summary statistics, or data conversions. | ‘Data analysis’ section |
|  | 13c | Describe any methods used to tabulate or visually display results of individual studies and syntheses. | ‘Data analysis’ section |
|  | 13d | Describe any methods used to synthesize results and provide a rationale for the choice(s). If meta-analysis was performed, describe the model(s), method(s) to identify the presence and extent of statistical heterogeneity, and software package(s) used. | ‘Data analysis’ section |
|  | 13e | Describe any methods used to explore possible causes of heterogeneity among study results (e.g. subgroup analysis, meta-regression). | ‘Data analysis’ section |
|  | 13f | Describe any sensitivity analyses conducted to assess robustness of the synthesized results. | ‘Data analysis’ section |
| Reporting bias assessment | 14 | Describe any methods used to assess risk of bias due to missing results in a synthesis (arising from reporting biases). | ‘Data analysis’ section |
| Certainty assessment | 15 | Describe any methods used to assess certainty (or confidence) in the body of evidence for an outcome. | ‘Study quality and certainty of evidence’ section |
| **RESULTS** | | |  |
| Study selection | 16a | Describe the results of the search and selection process, from the number of records identified in the search to the number of studies included in the review, ideally using a flow diagram. | ‘Study characteristics’ section |
|  | 16b | Cite studies that might appear to meet the inclusion criteria, but which were excluded, and explain why they were excluded. | ‘Study characteristics’ section |
| Study characteristics | 17 | Cite each included study and present its characteristics. | ‘Study characteristics’ section |
| Risk of bias in studies | 18 | Present assessments of risk of bias for each included study. | ‘Risk of bias’ and Supplemental’ sections |
| Results of individual studies | 19 | For all outcomes, present, for each study: (a) summary statistics for each group (where appropriate) and (b) an effect estimate and its precision (e.g. confidence/credible interval), ideally using structured tables or plots. | ‘Results’ and ‘Supplemental’ sections |
| Results of syntheses | 20a | For each synthesis, briefly summarise the characteristics and risk of bias among contributing studies. | ‘Results’ and ‘Supplemental’ sections |
|  | 20b | Present results of all statistical syntheses conducted. If meta-analysis was done, present for each the summary estimate and its precision (e.g. confidence/credible interval) and measures of statistical heterogeneity. If comparing groups, describe the direction of the effect. | ‘Results’ and ‘Supplemental’ sections |
|  | 20c | Present results of all investigations of possible causes of heterogeneity among study results. | N/A |
|  | 20d | Present results of all sensitivity analyses conducted to assess the robustness of the synthesized results. | ‘Subgroup analyses’ and ‘Supplemental’ sections |
| Reporting biases | 21 | Present assessments of risk of bias due to missing results (arising from reporting biases) for each synthesis assessed. | ‘Supplemental’ section |
| Certainty of evidence | 22 | Present assessments of certainty (or confidence) in the body of evidence for each outcome assessed. | ‘Certainty of evidence’ and ‘Supplemental’ sections |
| **DISCUSSION** | | |  |
| Discussion | 23a | Provide a general interpretation of the results in the context of other evidence. | ‘Discussion’ section |
|  | 23b | Discuss any limitations of the evidence included in the review. | ‘Strength and limitations of the study’ section |
|  | 23c | Discuss any limitations of the review processes used. | ‘Strength and limitations of the study’ section |
|  | 23d | Discuss implications of the results for practice, policy, and future research. | ‘Conclusion and future directions’ section |
| **OTHER INFORMATION** | | |  |
| Registration and protocol | 24a | Provide registration information for the review, including register name and registration number, or state that the review was not registered. | ‘Methods’ section |
|  | 24b | Indicate where the review protocol can be accessed, or state that a protocol was not prepared. | ‘Methods’ section |
|  | 24c | Describe and explain any amendments to information provided at registration or in the protocol. | N/A |
| Support | 25 | Describe sources of financial or non-financial support for the review, and the role of the funders or sponsors in the review. | ‘Declaration of Sources of Funding’ section |
| Competing interests | 26 | Declare any competing interests of review authors. | ‘Declaration of Conflicts of Interest’ section |
| Availability of data, code and other materials | 27 | Report which of the following are publicly available and where they can be found: template data collection forms; data extracted from included studies; data used for all analyses; analytic code; any other materials used in the review. | ‘Availability of data’ section |

*From:*  Page MJ, McKenzie JE, Bossuyt PM, Boutron I, Hoffmann TC, Mulrow CD, et al. The PRISMA 2020 statement: an updated guideline for reporting systematic reviews. BMJ 2021;372:n71. doi: 10.1136/bmj.n71. This work is licensed under CC BY 4.0. To view a copy of this license, visit <https://creativecommons.org/licenses/by/4.0/>

# Table S3. PRISMA 2020 for Abstracts Checklist

| **Section and Topic** | **Item #** | **Checklist item** | **Reported (Yes/No)** |
| --- | --- | --- | --- |
| **TITLE** | | |  |
| Title | 1 | Identify the report as a systematic review. | Yes |
| **BACKGROUND** | | |  |
| Objectives | 2 | Provide an explicit statement of the main objective(s) or question(s) the review addresses. | Yes |
| **METHODS** | | |  |
| Eligibility criteria | 3 | Specify the inclusion and exclusion criteria for the review. | No |
| Information sources | 4 | Specify the information sources (e.g. databases, registers) used to identify studies and the date when each was last searched. | Yes |
| Risk of bias | 5 | Specify the methods used to assess risk of bias in the included studies. | No |
| Synthesis of results | 6 | Specify the methods used to present and synthesise results. | Yes |
| **RESULTS** | | |  |
| Included studies | 7 | Give the total number of included studies and participants and summarise relevant characteristics of studies. | Yes |
| Synthesis of results | 8 | Present results for main outcomes, preferably indicating the number of included studies and participants for each. If meta-analysis was done, report the summary estimate and confidence/credible interval. If comparing groups, indicate the direction of the effect (i.e. which group is favoured). | Yes |
| **DISCUSSION** | | |  |
| Limitations of evidence | 9 | Provide a brief summary of the limitations of the evidence included in the review (e.g. study risk of bias, inconsistency and imprecision). | No |
| Interpretation | 10 | Provide a general interpretation of the results and important implications. | Yes |
| **OTHER** | | |  |
| Funding | 11 | Specify the primary source of funding for the review. | No |
| Registration | 12 | Provide the register name and registration number. | No |

*From:*  Page MJ, McKenzie JE, Bossuyt PM, Boutron I, Hoffmann TC, Mulrow CD, et al. The PRISMA 2020 statement: an updated guideline for reporting systematic reviews. BMJ 2021;372:n71. doi: 10.1136/bmj.n71

# Search strategy

delirium AND (prospective OR longitudinal)

# Table S4. Overview of included studies (sample size and time specific clinical outcomes)

| Author and year | Sample size (delirium/ no delirium) | Cognition | | | Functional outcome | | | Quality of life | | | Mental health | | | Dementia | | | Institutionalisation | | | Readmission | | | Mortality | | |
| --- | --- | --- | --- | --- | --- | --- | --- | --- | --- | --- | --- | --- | --- | --- | --- | --- | --- | --- | --- | --- | --- | --- | --- | --- | --- |
|  |  | < 6 months | >6–12 months | >12 months | < 6 months | >6–12 months | >12 months | < 6 months | >6–12 months | >12 months | < 6 months | >6–12 months | >12 months | < 6 months | >6–12 months | >12 months | < 6 months | >6–12 months | >12 months | < 6 months | >6–12 months | >12 months | < 6 months | >6–12 months | >12 months |
| Abelha 2013[1] | 89/473 |  |  |  | x |  |  |  |  |  |  |  |  |  |  |  |  |  |  |  |  |  | x |  |  |
| Adamis 2007[2] | 47/117 |  |  |  |  |  |  |  |  |  |  |  |  |  |  |  |  |  |  |  |  |  | x |  |  |
| Alberto 2018[3] | 35/204 |  |  |  |  |  |  |  |  |  |  |  |  |  |  |  |  |  |  |  |  |  |  | x |  |
| AlHuraizi 2023[4] | 153/131 |  |  |  |  |  |  |  |  |  |  |  |  |  |  |  |  |  |  |  |  |  | x | x |  |
| Alzoubi 2022[5] | 35/76 |  |  |  |  |  |  |  |  |  |  |  |  |  |  |  |  |  |  |  |  |  | x |  |  |
| Arneson 2023 [6] | 107/860 |  |  |  |  |  |  |  |  |  |  |  |  |  |  |  |  |  |  |  |  |  | x |  |  |
| Avelino-Silva 2017[7] | 207/527 |  |  |  |  |  |  |  |  |  |  |  |  |  |  |  |  |  |  |  |  |  |  | x |  |
| Avelino-Silva 2018[8] | 457/687 |  |  |  |  |  |  |  |  |  |  |  |  |  |  |  |  |  |  |  |  |  |  | x |  |
| Bagienski 2017[9] | 29/112 |  |  |  |  |  |  |  | x |  |  |  |  |  |  |  |  |  |  |  |  |  |  | x | x |
| Bakker 2012[10] | 63/138 |  |  |  |  |  |  |  |  |  |  |  |  |  |  |  |  |  |  |  |  |  | x |  |  |
| Beishuizen 2020[11] | 14/77 |  |  |  | - | - | - |  |  |  |  |  |  |  |  |  |  |  |  |  |  |  | - | - | - |
| Bellelli 2007[12] | 94/94 |  |  |  |  |  |  |  |  |  |  |  |  |  |  |  |  |  |  |  |  |  |  | x |  |
| Bellelli 2008[13] | 139/933 |  |  |  |  |  |  |  |  |  |  |  |  |  |  |  |  | x |  |  | x |  |  | x |  |
| Bellelli 2018[14] | 220/351 |  |  |  |  |  |  |  |  |  |  |  |  |  |  |  |  |  |  |  |  |  | x |  |  |
| Bickel 2008[15] | 41/159 |  |  | x |  | x |  |  |  |  |  |  |  |  | x | x |  |  |  |  |  |  |  |  | x |
| Brown 2018[16] | 76/66 | x | x |  |  |  |  |  |  |  |  |  |  |  |  |  |  |  |  |  |  |  |  |  |  |
| Brown 2020[17] | 1799/2234 |  |  |  |  |  |  |  |  |  |  | x |  |  | x |  |  |  |  |  |  |  |  |  |  |
| Bruck 2018[18] | 43/82 | x |  |  |  |  |  |  |  |  |  |  |  |  |  |  |  |  |  |  |  |  |  |  |  |
| Bryson 2011[19] | 23/46 | x |  |  |  |  |  |  |  |  |  |  |  |  |  |  |  |  |  |  |  |  |  |  |  |
| Bulic 2020[20] | 37/66 | x | x |  |  |  |  |  |  |  | x | x |  | x | x |  |  |  |  |  |  |  |  | x |  |
| Buurman 2011[21] | 118/504 |  |  |  |  | x |  |  |  |  |  |  |  |  |  |  |  |  |  |  |  |  |  |  |  |
| Cartei 2022[22] | 193/194 |  |  |  |  | x |  |  |  |  |  |  |  |  |  |  |  |  |  |  |  |  |  | x |  |
| Cavallari 2017[23] | 25/88 |  | x |  |  |  |  |  |  |  |  |  |  |  |  |  |  |  |  |  |  |  |  |  |  |
| Chalmers 2021[24] | 831/1640 |  |  |  |  |  |  |  |  |  |  |  |  |  |  |  |  |  |  |  |  |  |  |  | x |
| Chan 2016[25] | 49/104 |  |  |  |  |  |  |  |  |  |  |  |  |  |  |  |  |  |  |  |  |  | x | x |  |
| Chan 2017[26] | 39/117 | x | x |  | x | x |  |  |  |  | x | x |  |  |  |  |  |  |  |  |  |  | x | x |  |
| Chen 2017[27] | 44/92 | x | x | x |  |  |  |  | x |  |  |  |  |  |  |  |  |  |  |  |  |  |  |  |  |
| Cheong 2021[28] | 50/396 |  |  |  |  |  |  |  |  |  |  |  |  |  |  |  |  |  |  |  |  |  | x |  |  |
| Cirbus 2019[29] | 68/91 |  |  |  | x |  |  |  |  |  |  |  |  |  |  |  |  |  |  |  |  |  |  |  |  |
| Cole 2008[30] | 115/95 | x | x |  | x | x |  |  |  |  |  |  |  |  |  |  | x | x |  |  |  |  | x | x |  |
| Curyto 2001[31] | 12/41 |  |  |  |  |  |  |  |  |  |  |  |  |  |  |  |  |  |  |  |  |  |  |  | x |
| Czyzycki 2022[32] | 163/488 |  |  |  | x | x |  |  |  |  |  |  |  |  |  |  |  |  |  |  |  |  | x | x |  |
| Daiello 2019[33] | 129/419 | x |  |  |  |  |  |  |  |  |  |  |  |  |  |  |  |  |  |  |  |  |  |  |  |
| Dani 2018[34] | 72/636 |  |  |  |  |  |  |  |  |  |  |  |  |  |  |  |  |  |  |  |  |  |  |  | x |
| Davis 2012[35] | 121/429 |  |  |  | - | - | - |  |  |  |  |  |  | - | - | - |  |  |  |  |  |  |  |  |  |
| Davis 2014[36] | 122/2075 |  |  |  |  |  |  |  |  |  |  |  |  |  |  | x |  |  |  |  |  |  |  |  | x |
| DeCrane 2011[37] | 70/250 |  |  |  |  |  |  |  |  |  |  |  |  |  |  |  |  |  |  |  |  |  | x |  |  |
| Decrane 2012[38] | 70/250 |  |  |  |  | x |  |  |  |  |  |  |  |  |  |  |  |  |  |  |  |  |  |  |  |
| deHaan 2023 [39] | 326/1725 |  |  |  |  |  |  |  |  |  |  |  |  |  |  |  |  |  |  |  |  |  | x | x |  |
| deJong 2019[40] | 121/342 |  |  |  |  |  |  |  |  |  |  |  |  |  |  |  |  | x |  |  |  |  | x | x |  |
| DelaVarga-Martínez 2022[41] | 55/160 |  |  | x |  |  | x |  |  |  |  |  | x |  |  |  |  |  |  |  |  |  |  |  |  |
| Ditzel 2023[42] | 38/208 | x |  |  |  |  |  |  |  |  |  |  |  |  |  |  |  |  |  |  |  |  | x |  |  |
| Diwell 2018[43] | 56/301 |  |  |  |  |  |  |  |  |  |  |  |  |  |  |  |  |  |  |  |  |  | x |  | x |
| Dostovic 2021[44] | 75/96 |  |  | x |  |  |  |  |  |  |  |  |  |  | x |  |  |  |  |  |  |  |  |  |  |
| Drews 2015[45] | 77/482 |  |  |  |  |  |  |  |  |  | x |  |  |  |  |  |  |  |  |  |  |  |  |  |  |
| Dros 2020[46] | 67/384 |  |  |  |  |  |  |  |  |  |  |  |  |  | x |  |  |  |  |  |  |  |  |  |  |
| Duppils 2004[47] | 32/83 | x |  |  |  |  |  | x |  |  |  |  |  |  |  |  |  |  |  |  |  |  |  |  |  |
| Duprey 2020[48] | 542/953 |  |  |  |  |  |  |  |  |  |  |  |  |  |  |  |  |  |  |  |  |  | x |  |  |
| Durlach 2023[49] | 42/193 |  |  |  | x |  |  |  |  |  |  |  |  |  |  |  | x |  |  | x |  |  | x |  |  |
| Edelstein 2004[50] | 25/613 |  |  |  |  | x |  |  |  |  |  |  |  |  |  |  |  |  |  |  |  |  |  | x |  |
| Edlund 2006[51] | 125/275 |  |  |  |  |  |  |  |  |  |  |  |  |  |  |  |  |  |  |  |  |  |  | x |  |
| Eeles 2010[52] | 37/136 |  |  |  |  |  |  |  |  |  |  |  |  |  |  |  |  | x | x |  |  |  |  |  |  |
| Eeles 2012[53] | 102/171 |  |  |  |  |  | x |  |  |  |  |  |  |  |  |  |  |  |  |  |  |  |  |  |  |
| Eide 2016[54] | 48/25 | x |  |  | x |  |  | x |  |  |  |  |  |  |  |  |  |  |  |  |  |  |  |  |  |
| Eide 2016_1[55] | 76/60 |  |  |  |  |  |  |  |  |  |  |  |  |  |  |  |  |  |  | x |  |  |  |  |  |
| Elsayem 2017[56] | 44/198 |  |  |  |  |  |  |  |  |  |  |  |  |  |  |  |  |  |  |  |  |  | x | x | x |
| Evensen 2021[57] | 83/145 |  |  |  |  |  |  |  |  |  |  |  |  |  |  |  |  |  |  |  |  |  |  | x |  |
| Falsini 2018[58] | 111/615 |  |  |  |  |  |  |  |  |  |  |  |  |  |  |  |  |  |  | x |  |  | x |  |  |
| FialhoSilva 2021[59] | 71/151 |  |  |  | x |  |  |  |  |  |  |  |  |  |  |  |  |  |  |  |  |  | x |  |  |
| Fick 2013[60] | 44/95 |  |  |  | x |  |  |  |  |  |  |  |  |  |  |  |  |  |  |  |  |  | x |  |  |
| Francis 1990[61] | 50/176 |  |  |  | x |  |  |  |  |  |  |  |  |  |  |  | x |  |  |  |  |  | x |  |  |
| Francis 1992[62] | 45/160 |  |  | x |  |  | x |  |  |  |  |  |  |  |  |  |  |  |  |  |  |  |  |  | x |
| Franck 2016[63] | 270/580 | x |  |  |  |  |  |  |  |  |  |  |  |  |  |  |  |  |  |  |  |  |  |  |  |
| Furlaneto 2007[64] | 25/60 |  |  |  |  |  | x |  |  |  |  |  |  |  |  |  |  |  |  |  |  |  |  |  | x |
| Gandossi 2023[65] | 46/210 |  |  |  | x |  |  |  |  |  |  |  |  |  |  |  |  |  |  |  |  |  |  |  |  |
| Garcez 2019[66] | 66/243 |  |  |  |  |  |  |  |  |  |  |  |  |  |  | x |  |  |  |  |  |  |  |  |  |
| George 1997[67] | 171/95 |  |  |  |  |  |  |  |  |  |  |  |  |  |  |  | x | x |  | x | x |  | x | x |  |
| Giroux 2021[68] | 69/310 | x |  |  | x |  |  |  |  |  |  |  |  |  |  |  |  |  |  |  |  |  |  |  |  |
| Givens 2008[69] | 52/74 |  |  |  | x |  |  |  |  |  |  |  |  |  |  |  |  |  |  |  |  |  |  |  |  |
| Givens 2009[70] | 39/420 |  |  |  | x |  |  |  |  |  |  |  |  |  |  |  |  | x |  |  |  |  |  | x |  |
| Gleason 2015[71] | 115/404 |  |  |  |  |  |  |  |  |  |  |  |  |  |  |  |  |  |  | x |  |  |  |  |  |
| Gonçalves 2023[72] | 276/829 |  | x |  |  |  |  |  |  |  |  |  |  |  |  |  |  |  |  |  |  |  |  |  |  |
| Gonzalez 2005[73] | 58/91 |  |  |  |  |  |  |  |  |  |  |  |  |  |  |  |  |  |  |  |  |  | x |  |  |
| Gonzalez 2009[74] | 192/350 |  |  |  |  |  |  |  |  |  |  |  |  |  |  |  |  |  |  |  |  |  | x |  |  |
| Gottschalk 2015[75] | 151/308 |  |  |  |  |  |  |  |  |  |  |  |  |  |  |  |  |  |  |  |  |  |  |  | x |
| Gou 2021[76] | 122/375 |  |  |  |  |  |  |  |  |  |  |  |  |  |  |  |  |  |  |  |  |  |  | x |  |
| Goudzwaard 2020[77] | 75/468 |  |  |  |  |  |  |  |  |  |  |  |  |  |  |  |  |  |  |  |  |  |  |  | x |
| Gual 2018[78] | 352/557 |  |  |  |  |  |  |  |  |  |  |  |  |  |  |  |  |  |  | x |  |  |  |  |  |
| Guenther 2020[79] | 39/86 |  |  |  | x |  |  |  |  |  |  |  |  |  |  |  |  |  |  |  |  |  |  |  |  |
| Han 2010[80] | 108/520 |  |  |  |  |  |  |  |  |  |  |  |  |  |  |  |  |  |  |  |  |  | x |  |  |
| Han 2017[81] | 155/929 |  |  |  |  |  |  |  |  |  |  |  |  |  |  |  |  |  |  |  |  |  | x |  |  |
| Han 2022[82] | 87/957 |  |  |  |  |  |  |  |  |  |  |  |  |  |  |  |  |  |  | x |  |  | x |  |  |
| Hapca 2018[83] | 1065/4344 |  |  |  |  |  |  |  |  |  |  |  |  |  |  |  |  |  |  |  |  |  | x | x | x |
| Hawley 2023[84] | 1058/17761 |  |  |  | x |  |  |  |  |  |  |  |  |  |  |  | x |  |  |  |  |  |  |  |  |
| Hempenius 2016[85] | 31/222 | x |  |  | x |  |  | x |  |  |  |  |  |  |  |  |  |  |  | x |  |  | x |  |  |
| Hofhuis 2022[86] | 182/300 |  |  |  |  |  |  |  | x |  |  |  |  |  |  |  |  |  |  |  |  |  |  |  |  |
| Hölttä 2011[87] | 66/189 |  |  |  |  |  |  |  |  |  |  |  |  |  |  |  |  |  | x |  |  |  |  |  | x |
| Honda 2016[88] | 134/465 |  |  |  |  |  |  |  |  |  |  |  |  |  |  |  |  |  |  |  |  |  | x | x | x |
| Hoogma 2023[89] | 93/157 | x |  |  | x |  |  | x |  |  |  |  |  |  |  |  |  |  |  | x |  |  | x |  |  |
| Hshieh 2017[90] | 135/431 |  |  |  | x |  | x |  |  |  |  |  |  |  |  |  |  |  |  |  |  |  |  |  |  |
| Hshieh 2023[91] | 56/179 |  |  |  |  |  |  |  |  |  |  |  |  |  |  |  |  |  |  |  |  |  | x | x |  |
| Hughes 2021[92] | 740/300 |  |  |  |  |  |  |  |  |  |  |  |  |  |  |  |  |  |  |  |  |  |  | x |  |
| Humbert 2021[93] | 15/62 | x |  |  | x |  |  |  |  |  |  |  |  |  |  |  |  |  |  |  |  |  |  |  |  |
| Inouye 1998[94] | 81/599 |  |  |  | x |  |  |  |  |  |  |  |  |  |  |  | x |  |  |  |  |  |  |  |  |
| Inouye 2016[95] | 129/419 | x | x | x |  |  |  |  |  |  |  |  |  |  |  |  |  |  |  |  |  |  |  |  | x |
| Isaia 2009[96] | 14/130 |  |  |  | x |  |  |  |  |  |  |  |  |  |  |  |  |  |  |  |  |  | x |  |  |
| Iwata 2020[97] | 109/299 |  |  |  |  |  |  |  |  |  |  |  |  |  |  |  |  |  |  |  |  |  | x | x |  |
| Jackson 2014[98] | 352/115 |  |  |  | x | x |  | x | x |  | x | x |  |  |  |  |  |  |  |  |  |  |  |  |  |
| Jankowski 2011[99] | 37/33 | x |  |  | x |  |  |  |  |  |  |  |  |  |  |  |  |  |  |  |  |  |  |  |  |
| Janssen 2021[100] | 9/194 | x | x |  |  |  |  | x | x |  | x | x |  |  |  |  |  |  |  |  |  |  |  |  |  |
| Juliebø 2010[101] | 101/50 |  |  |  |  |  |  |  |  |  |  |  |  |  |  |  |  |  |  |  |  |  |  | x |  |
| Kainz 2022[102] | 72/150 | x |  |  |  |  |  | x |  |  |  |  |  |  |  |  |  |  |  |  |  |  |  |  |  |
| Kakuma 2003[103] | 30/77 |  |  |  |  |  |  |  |  |  |  |  |  |  |  |  |  |  |  |  |  |  | x | x | x |
| Kat 2008[104] | 71/41 | - | - | - |  |  |  |  |  |  | - | - | - | - | - | - | - | - | - |  |  |  | - | - | - |
| Kat 2011[105] | 67/522 |  |  |  |  |  |  |  |  |  |  |  |  |  |  |  |  |  |  |  |  |  |  |  | x |
| Katz 2001[106] | 12/35 | - | - | - | - | - | - |  |  |  |  |  |  |  |  |  |  |  |  |  |  |  |  |  |  |
| Kennedy 2014[107] | 63/613 |  |  |  |  |  |  |  |  |  |  |  |  |  |  |  |  |  |  | x |  |  | x |  |  |
| Kilicaslan 2022[108] | 44/371 |  |  |  |  |  |  |  |  |  |  |  |  |  |  |  |  |  |  |  |  |  | x | x | x |
| Knauf 2019[109] | 202/193 |  |  |  |  |  |  |  |  |  |  |  |  |  |  |  |  |  |  |  |  |  |  |  | x |
| Korber 2021[110] | 16/161 |  |  |  |  |  |  |  |  |  |  |  |  |  |  |  |  |  |  |  |  |  |  | x | x |
| Koster 2012[111] | 52/248 | x |  |  |  |  |  | x |  |  | x |  |  |  |  |  |  |  |  | x |  |  | x |  |  |
| Kotfis 2019[112] | 121/639 |  |  |  |  |  |  |  |  |  |  |  |  |  |  |  |  |  |  |  |  |  | x | x |  |
| Krogseth 2011[113] | 29/77 |  |  |  |  |  |  |  |  |  |  |  |  | x |  |  |  |  |  |  |  |  |  |  |  |
| Krogseth 2014[114] | 80/127 |  |  |  |  |  |  |  |  |  |  |  |  |  |  |  | x |  |  |  |  |  |  |  |  |
| Krogseth 2016[115] | 201/86 | - | - | - |  |  |  |  |  |  |  |  |  |  |  |  |  |  |  |  |  |  |  |  |  |
| Krogseth 2023[116] | 39/63 |  |  | x |  |  |  |  |  |  |  |  |  |  |  |  |  |  |  |  |  |  |  |  |  |
| Krzych 2014[117] | 237/5544 |  |  |  |  |  |  |  |  |  |  |  |  |  |  |  |  |  |  |  |  |  | - | - | - |
| Kunicki 2023[118] | 134/560 | x | x | x |  |  |  |  |  |  |  |  |  |  |  |  |  |  |  |  |  |  | x | x | x |
| Labaste 2020[119] | 61/112 |  |  |  |  |  |  |  |  | x |  |  |  |  |  |  |  |  |  |  |  |  |  |  | x |
| Labaste 2023[120] | 14/49 |  |  |  |  |  |  |  |  |  |  |  |  |  |  |  | x |  |  |  |  |  |  |  |  |
| Large 2013[121] | 14/35 |  |  |  |  |  |  |  |  |  |  |  |  |  |  |  |  |  |  | x |  |  | x | x |  |
| Lee 2011[122] | 56/162 |  |  |  |  |  | x |  |  |  |  |  |  |  |  |  |  |  |  |  |  |  |  |  | x |
| Lee 2017[123] | 73/248 |  |  |  |  |  |  |  |  |  |  |  |  |  |  |  |  |  |  |  |  |  | x | x |  |
| Lee 2018[124] | 83/517 |  |  |  |  |  |  |  |  |  |  |  |  |  |  |  |  |  |  |  |  |  | x |  |  |
| Leslie 2005[125] | 115/804 |  |  |  |  |  |  |  |  |  |  |  |  |  |  |  |  |  |  |  |  |  |  | x |  |
| Leslie 2008[126] | 109/732 |  |  |  |  |  |  |  |  |  |  |  |  |  |  |  |  |  |  |  |  |  |  | x |  |
| Lewis 1995[127] | 20/362 |  |  |  |  |  |  |  |  |  |  |  |  |  |  |  |  |  |  |  |  |  | x |  |  |
| Li 2019[128] | 32/79 |  |  |  |  |  |  |  |  |  |  |  |  |  |  |  |  |  |  | x |  |  |  | x |  |
| Li 2021[129] | 15/198 |  |  |  |  |  |  |  |  |  |  |  |  |  |  |  |  |  |  |  |  |  | x | x |  |
| Liang 2014[130] | 17/165 |  |  |  | x | x |  |  |  |  |  |  |  |  |  |  |  |  |  |  |  |  |  |  |  |
| Lima 2010[131] | 63/133 |  |  |  |  |  |  |  |  |  |  |  |  |  |  |  |  |  |  |  |  |  |  | x |  |
| Lingehall 2017[132] | 64/50 |  |  | x |  |  |  |  |  |  |  |  |  |  |  | x |  |  |  |  |  |  |  |  |  |
| Lundström 2003[133] | 29/49 |  |  |  |  |  |  |  |  |  |  |  |  |  |  | x |  |  |  |  |  |  |  |  | x |
| Luz 2020[134] | 89/80 |  |  |  |  |  |  | x |  |  |  |  |  |  |  |  |  |  |  |  |  |  | x |  |  |
| Maclullich 2019[135] | 95/690 |  |  |  |  |  |  |  |  |  |  |  |  |  |  |  | x |  |  |  |  |  | x |  |  |
| Marcantonio 2005[136] | 188/316 |  |  |  |  |  |  |  |  |  |  |  |  |  |  |  |  |  |  | x |  |  |  |  |  |
| Mariz 2013[137] | 28/50 |  |  |  |  |  |  |  |  |  |  |  |  |  |  |  | x |  |  |  |  |  | x |  |  |
| Marrama 2022[138] | 74/125 |  |  |  |  |  |  |  |  |  |  |  |  |  | x | x |  |  |  |  |  |  |  | x | x |
| Mathies 2020[139] | 21/47 |  | x |  |  |  |  |  |  |  |  |  |  |  |  |  |  |  |  |  |  |  |  |  |  |
| Mauri 2021[140] | 66/595 |  |  |  |  |  |  |  |  |  |  |  |  |  |  |  |  |  |  |  |  |  | x | x | x |
| Mazzola 2015[141] | 135/140 |  |  |  |  |  |  |  |  |  |  |  |  |  |  |  |  |  |  |  |  |  | x |  |  |
| McAvay 2006[142] | 31/378 |  |  |  |  |  |  |  |  |  |  |  |  |  |  |  |  | x |  |  |  |  |  | x |  |
| McCusker 2001[143] | 220/95 | x | x |  | x | x |  |  |  |  |  |  |  |  |  |  |  | x |  |  |  |  |  | x |  |
| McCusker 2002[144] | 243/118 |  |  |  |  |  |  |  |  |  |  |  |  |  |  |  |  |  |  |  |  |  |  | x |  |
| McCusker 2014[145] | 31/243 | x |  |  | x |  |  |  |  |  |  |  |  |  |  |  |  |  |  |  |  |  | x |  |  |
| Minden 2005[146] | 8/27 |  |  |  |  |  |  | x |  |  |  |  |  |  |  |  |  |  |  |  |  |  | x |  |  |
| Miu 2013[147] | 86/228 |  |  |  | x | x |  |  |  |  |  |  |  |  |  |  | x | x |  |  |  |  |  | x |  |
| Miyamoto 2021[148] | 22/59 |  |  |  | x | x |  |  |  |  | x | x |  |  |  |  |  |  |  |  |  |  |  |  |  |
| Monacelli 2018[149] | 85/133 |  |  |  | x |  |  |  |  |  |  |  |  |  |  |  |  |  |  |  |  |  | x |  |  |
| Moon 2018[150] | 83/90 |  |  |  |  |  |  |  |  |  |  |  |  |  |  |  |  |  |  | x |  |  | x |  |  |
| Morandi 2014[151] | 323/2319 |  |  |  |  | x |  |  |  |  |  |  |  |  |  |  |  | x |  |  |  |  |  | x |  |
| Moreno-Gavino 2012[152] | 198/1236 |  |  |  |  |  |  |  |  |  |  |  |  |  |  |  |  |  |  |  |  |  |  | x |  |
| Moskowitz 2017[153] | 70/94 |  |  |  |  |  |  |  |  |  |  |  |  |  |  |  |  |  |  |  |  |  |  |  | x |
| Muller 2023[154] | 11/46 | x |  |  |  |  |  |  |  |  |  |  |  |  |  |  |  |  |  |  |  |  |  |  |  |
| Muresan 2016[155] | 46/153 |  |  |  |  |  |  |  |  |  |  |  |  |  |  |  |  |  |  |  |  |  |  | x |  |
| Murray 1993[156] | 35/143 |  |  |  | x |  |  |  |  |  |  |  |  |  |  |  |  |  |  |  |  |  |  |  |  |
| Muzzana 2022[157] | 15/187 |  |  |  |  |  |  |  |  |  |  |  |  |  |  |  |  |  |  | x |  |  |  |  |  |
| Naidech 2013[158] | 29/56 |  |  |  |  |  |  |  | x |  |  |  |  |  |  |  |  |  |  |  |  |  |  |  |  |
| Neerland 2017[159] | 336/360 |  |  |  |  |  |  |  |  |  |  |  |  |  | x |  |  |  |  |  |  |  |  |  |  |
| Nerdal 2022[160] | 10/117 | x |  | x |  |  |  |  |  |  | x |  | x | x |  | x |  |  |  |  |  |  |  |  |  |
| Neufeld 2015[161] | 36/45 |  |  | x |  |  | x |  |  |  |  |  |  |  |  |  |  |  | x |  |  | x |  |  |  |
| Nguyen 2018[162] | 44/153 | x |  |  |  |  |  |  |  |  | x |  |  |  |  |  |  |  |  |  |  |  |  |  |  |
| Nishizawa 2023[163] | 334/743 |  |  |  |  |  |  |  |  |  |  |  |  |  |  |  |  |  |  |  |  |  | x | x |  |
| Noriega 2015[164] | 35/168 |  |  |  | x | x |  |  |  |  |  |  |  |  |  |  |  |  |  | x | x |  | x | x |  |
| Ogawa 2017[165] | 43/283 |  |  |  | - | - | - |  |  |  |  |  |  |  |  |  |  |  |  |  |  |  |  |  |  |
| Ojagbemi 2020[166] | 29/121 | x |  |  | x |  |  |  |  |  |  |  |  | x |  |  |  |  |  |  |  |  |  |  |  |
| Ojagbemi 2021[167] | 29/121 |  |  |  |  |  |  |  |  |  |  |  |  |  |  |  |  |  |  |  |  |  | x | x |  |
| O'Keeffe 1997[168] | 94/131 |  |  |  |  |  |  |  |  |  |  |  |  |  |  |  | x |  |  |  |  |  | x |  |  |
| Oldenbeuving 2011[169] | 62/465 |  |  |  | x |  |  |  |  |  |  |  |  |  |  |  |  |  |  |  |  |  |  |  |  |
| Olofsson 2018[170] | 75/60 | x | x | x |  |  |  |  |  |  | x | x | x | x | x | x |  |  |  |  |  |  | x | x | x |
| Pak 2020[171] | 36/96 |  |  |  |  |  |  |  |  |  |  |  |  |  |  |  |  |  |  |  |  |  | x |  |  |
| Pandharipande 2013[172] | 352/115 | x | x |  |  |  |  |  |  |  |  |  |  |  |  |  |  |  |  |  |  |  |  |  |  |
| Pasinska 2019[173] | 164/518 |  |  |  | x | x |  |  |  |  |  |  |  |  |  |  |  |  |  |  |  |  | x | x |  |
| Patel 2014[174] | 87/10 |  |  |  |  |  |  |  |  |  |  |  |  |  |  |  |  |  |  |  |  |  | x | x |  |
| Paulino 2023[175] | 34/72 |  |  |  | x |  |  |  |  |  |  |  |  |  |  |  | x |  |  | x |  |  | x |  |  |
| Pendlebury 2015[176] | 81/202 |  |  |  |  |  |  |  |  |  |  |  |  |  |  |  |  |  |  | x |  |  |  |  |  |
| Penfold 2023[177] | 189/1151 |  |  |  |  |  |  |  |  |  |  |  |  |  |  |  |  |  |  | x |  |  | x |  |  |
| Pitkala 2005[178] | 106/319 |  |  |  |  |  |  |  |  |  |  |  |  |  |  |  |  |  | x |  |  |  |  | x | x |
| Pompei 1994[179] | 57/359 |  |  |  |  |  |  |  |  |  |  |  |  |  |  |  |  |  |  |  |  |  | x |  |  |
| Praditsuwan 2013[180] | 110/115 |  |  |  |  |  |  |  |  |  |  |  |  |  |  |  |  |  |  |  |  |  | x |  |  |
| Qu 2018[181] | 38/223 |  |  |  | x |  |  |  |  |  |  |  |  |  |  |  |  |  |  |  |  |  | x |  |  |
| Quinlan 2011[182] | 61/887 |  |  |  | x |  |  |  |  |  |  |  |  |  |  |  |  |  |  |  |  |  |  |  |  |
| Raats 2015[183] | 35/197 |  |  |  |  |  |  |  |  |  |  |  |  |  |  |  |  |  |  |  |  |  | x | x |  |
| Racine 2018[184] | 27/34 |  |  |  | x |  |  |  |  |  |  |  |  |  |  |  | x |  |  | x |  |  |  |  |  |
| Racine 2020[185] | 30/110 | x |  | x |  |  |  |  |  |  |  |  |  |  |  |  |  |  |  |  |  |  |  |  |  |
| Radcliffe 2023[186] | 109/161 |  |  |  |  |  |  |  |  |  |  |  |  |  |  |  |  |  |  |  |  |  | x |  |  |
| Radinovic 2014[187] | 88/99 |  |  |  |  |  |  |  |  |  |  |  |  |  |  |  |  |  |  |  |  |  | x |  |  |
| Rahkonen 2001[188] | 20/179 |  |  |  |  |  |  |  |  |  |  |  |  |  |  | x |  |  |  |  |  |  |  |  | x |
| Rawle 2021[189] | 77/500 |  |  |  |  |  | x |  |  |  |  |  |  |  |  | x |  |  | x |  |  |  |  |  |  |
| Rego 2023[190] | 101/176 |  |  |  |  |  |  |  |  |  |  |  |  |  |  |  |  |  |  |  |  |  |  | x |  |
| Reynish 2017[191] | 901/3584 |  |  |  |  |  |  |  |  |  |  |  |  |  |  |  |  |  |  |  |  |  | x | x |  |
| Richardson 2021[192] | 82/123 |  |  |  |  |  |  |  |  |  |  |  |  |  | x |  |  |  |  |  |  |  |  |  |  |
| Richardson 2021_2[193] | 48/87 | - | - | - |  |  |  |  |  |  |  |  |  |  |  |  |  |  |  |  |  |  |  |  |  |
| Rizzi 2015[194] | 35/204 |  |  |  |  |  |  |  |  |  |  |  |  |  |  |  |  |  |  |  |  |  | x |  |  |
| Robinson 2009[195] | 64/89 |  |  |  |  |  |  |  |  |  |  |  |  |  |  |  |  |  |  |  |  |  | x |  |  |
| Robinson 2011[196] | 74/98 |  |  |  |  |  |  |  |  |  |  |  |  |  |  |  |  |  |  |  |  |  | x |  |  |
| Rockwood 1999[197] | 38/165 |  |  |  |  |  |  |  |  |  |  |  |  |  |  | x |  |  |  |  |  |  |  |  | x |
| Rolandi 2020[198] | 12/1088 |  |  |  |  |  |  |  |  |  |  |  |  | - | - | - |  |  |  |  |  |  |  |  |  |
| Rollo 2022[199] | 31/72 |  |  |  | x |  |  |  |  |  |  |  |  |  |  |  |  |  |  |  |  |  | x |  |  |
| Rosenthal 2017[200] | 18/47 |  |  |  |  |  |  | x | x |  |  |  |  |  |  |  |  |  |  |  |  |  |  |  |  |
| Rudolph 2008[201] | 61/885 | x |  |  |  |  |  |  |  |  |  |  |  |  |  |  |  |  |  |  |  |  |  |  |  |
| Rudolph 2010[202] | 33/64 |  |  |  | x | x |  |  |  |  |  |  |  |  |  |  |  |  |  |  |  |  |  |  |  |
| Ruggiero 2017[203] | 133/381 |  |  |  |  |  |  |  |  |  |  |  |  |  |  |  |  |  |  |  |  |  |  | x |  |
| Saczynski 2012[204] | 103/122 | x | x |  |  |  |  |  |  |  |  |  |  |  |  |  |  |  |  |  |  |  |  |  |  |
| Sánchez-Lozano 2023[205] | 96/273 | x |  |  | x |  |  | x |  |  |  |  |  |  |  |  |  |  |  |  |  |  | x |  |  |
| Sanguanwit 2023[206] | 49/124 |  |  |  |  |  |  |  |  |  |  |  |  |  |  |  |  |  |  | x |  |  | x |  |  |
| Sasajima 2012[207] | 88/211 |  |  |  |  |  |  |  |  |  |  |  |  |  |  |  |  |  |  |  |  |  |  | x | x |
| Sato 2017[208] | 35/128 |  |  |  |  |  |  |  |  |  |  |  |  |  |  |  |  |  |  |  |  |  | x |  |  |
| Sauer 2017[209] | 22/154 | x | x |  |  |  |  |  |  |  |  |  |  |  |  |  |  |  |  |  |  |  | x | x |  |
| Serrano-Duenas 2005[210] | 21/21 | x | x | x |  |  |  |  |  |  |  |  |  |  |  |  |  |  |  |  |  |  |  |  | x |
| Sheng 2006[211] | 39/117 | x |  |  | x |  |  |  |  |  |  |  |  |  |  |  |  | x |  |  |  |  | x | x |  |
| Shi 2019[212] | 28/77 |  |  |  | x | x |  |  |  |  |  |  |  |  |  |  |  |  |  |  |  |  |  |  | x |
| Shi 2019_2[213] | 20/68 |  |  |  |  |  | x |  |  |  |  |  |  |  |  |  |  |  |  |  |  |  |  |  |  |
| Shim 2015[214] | 90/206 |  |  |  | x |  |  |  |  |  |  |  |  |  |  |  |  |  |  |  |  |  |  |  |  |
| Shintani 2009[215] | 182/42 |  |  |  |  |  |  |  |  |  |  |  |  |  |  |  |  |  |  |  |  |  | x |  |  |
| Singler 2014[216] | 19/114 |  |  |  | x |  |  |  |  |  |  |  |  |  |  |  |  |  |  | x |  |  |  |  |  |
| Slor 2013[217] | 23/30 |  |  |  |  |  |  |  |  |  | x |  |  |  |  |  |  |  |  |  |  |  |  |  |  |
| Sri-on 2016[218] | 27/205 |  |  |  |  |  |  |  |  |  |  |  |  |  |  |  |  |  |  |  |  |  | x |  |  |
| Suraarunsumrit 2022[219] | 42/247 |  |  |  | x |  |  | x |  |  |  |  |  |  |  |  |  |  |  | x |  |  | x |  |  |
| Svenningsen 2014[220] | 148/131 | x |  |  | x |  |  | x |  |  |  |  |  |  |  |  |  |  |  |  |  |  |  |  |  |
| Svenningsen 2015[221] | 158/222 |  |  |  |  |  |  |  |  |  | x |  |  |  |  |  |  |  |  |  |  |  |  |  |  |
| Tahir 2018[222] | 70/91 |  |  |  |  |  |  |  |  |  |  |  |  |  |  |  |  |  |  |  |  |  | x | x |  |
| Tan AH 2015[223] | 28/460 |  |  |  |  |  |  |  |  |  |  |  |  |  |  |  |  |  |  |  |  |  | x |  |  |
| Tavares 2021[224] | 10/235 |  |  |  | x |  |  |  |  |  |  |  |  |  |  |  |  |  |  |  |  |  |  |  |  |
| To-Adithep 2023[225] | 170/934 |  |  |  |  | x |  |  |  |  |  |  |  |  |  |  |  |  |  |  |  |  |  | x |  |
| Traissac 2011[226] | 241/53 |  |  |  |  |  |  |  |  |  |  |  |  |  |  |  |  |  |  | x |  |  |  |  |  |
| Trevisan 2023[227] | 132/442 |  |  |  |  |  |  |  |  |  |  |  |  |  |  |  |  |  |  |  |  |  |  | x |  |
| Tripathy 2014[228] | 56/687 |  |  |  |  |  |  |  |  |  |  |  |  |  |  |  |  |  |  |  |  |  | x | x |  |
| Tsai 2012[229] | 172/44 |  |  |  |  |  |  |  |  |  |  |  |  |  |  |  |  |  |  |  |  |  |  | x | x |
| Uthamalingam 2011[230] | 134/744 |  |  |  |  |  |  |  |  |  |  |  |  |  |  |  |  |  |  | x |  |  | x |  |  |
| vandenBoogaard 2012[231] | 171/744 |  |  | x |  |  | x |  |  | x |  |  |  |  |  |  |  |  |  |  |  |  |  |  |  |
| VanderHeijden 2023[232] | 529/1871 |  | x |  |  | x |  |  | x |  |  | x |  |  |  |  |  |  |  |  |  |  |  |  |  |
| VanDerWulp 2019[233] | 116/587 |  |  |  |  |  |  |  |  |  |  |  |  |  |  |  |  |  |  |  |  |  |  | x | x |
| vanEijsden 2015[234] | 29/63 |  |  |  |  |  |  |  |  |  |  |  |  |  |  |  |  |  |  |  |  |  | x | x | x |
| vanRijsbergen 2011[235] | 52/44 |  |  | x |  |  |  |  |  |  |  |  |  |  |  | x |  |  |  |  |  |  |  |  | x |
| VanRompaey 2009[236] | 20/85 |  |  |  |  |  |  | x |  |  |  |  |  |  |  |  |  |  |  |  |  |  | x |  |  |
| Vasunilashorn 2016[237] | 620/299 |  |  |  |  |  |  |  |  |  |  |  |  |  |  |  |  |  |  |  |  |  | x |  |  |
| Vasunilashorn 2018[238] | 285/229 | x | x | x |  |  |  |  |  |  |  |  |  |  |  |  |  |  |  |  |  |  |  |  |  |
| Vasunilashorn 2022[239] | 184/168 |  |  |  |  |  |  |  |  |  |  |  |  |  |  |  |  |  |  | x | x |  | x | x |  |
| Veiga 2012[240] | 128/552 |  |  |  |  |  |  |  |  |  |  |  |  |  |  |  |  |  |  |  |  |  | x |  |  |
| Verloo 2016[241] | 20/94 | x |  |  | x |  |  |  |  |  | x |  |  |  |  |  |  |  |  |  |  |  |  |  |  |
| Vida 2006[242] | 60/72 |  |  |  | x | x | x |  |  |  |  |  |  |  |  |  |  |  |  |  |  |  |  |  |  |
| Visser 2015[243] | 22/441 |  |  |  |  |  |  |  |  |  |  |  |  |  |  |  |  | x |  |  |  |  |  | x |  |
| Vives-Borrás 2019[244] | 37/490 | x |  |  | x |  |  |  |  |  |  |  |  |  |  |  |  |  |  |  |  |  | x |  |  |
| Wang 2021[245] | 19/108 |  |  |  |  |  | x |  |  |  |  |  |  |  |  |  |  |  |  |  |  |  |  |  | x |
| Weng 2019[246] | 85/64 |  |  |  | x |  |  |  |  |  |  |  |  |  |  |  |  |  |  |  |  |  |  |  |  |
| Whittamore 2014[247] | 107/142 | x |  |  | x |  |  |  |  |  | x |  |  |  |  |  |  |  |  | x |  |  | x |  |  |
| Witlox 2013[248] | 22/26 | x |  |  | x |  |  |  |  |  | x |  |  |  |  |  |  |  |  |  |  |  |  |  |  |
| Wolters 2014[249] | 412/689 | x | x |  |  |  |  |  | x |  |  |  |  |  |  |  |  |  |  |  |  |  |  | x |  |
| Wolters 2017[250] | 186/177 | - | - | - |  |  |  |  |  |  |  |  |  |  |  |  |  |  |  |  |  |  |  |  |  |
| Zakriya 2004[251] | 19/69 |  |  |  | x |  |  |  |  |  |  |  |  |  |  |  | x |  |  | x |  |  | x |  |  |
| Ziman 2020[252] | 332/983 |  |  |  |  |  |  |  |  |  |  |  |  |  |  |  |  |  |  |  |  |  |  |  | x |
| Zipprich 2020[253] | 64/527 |  |  |  | x |  |  | x |  |  |  |  |  |  |  |  |  |  |  |  |  |  | x | x | x |

Footnote: The highest sample size was included when the study has different sample size for different follow-up time and multiple outcomes

Studies that don’t have specific time points (-)

# Table S5. Overview of included studies and their demographic characteristics

| **Author and year** | **Country** | **Study design** | **Sex**  **M/F, N** | **Age**  **(M/SD)** | **Setting** | **Delirium tool/s** |
| --- | --- | --- | --- | --- | --- | --- |
| Abelha 2013[1] | Portugal | Prospective cohort | 354/208 | 64.6/14.87 | Surgical ICU | ICDSC |
| Adamis 2007[2] | UK | Prospective cohort | 54/110 | 84.6/6.57 | Medical unit | CAM |
| Alberto 2018[3] | Spain | Prospective cohort | 93/146 | 81.7/9.4 | ED | bCAM |
| AlHuraizi 2023[4] | Oman | Prospective cohort | 136/148 | 71.7/8.94 | Medical wards | 3D-CAM |
| Alzoubi 2022[5] | Jordan | Prospective cohort | 56/55 | 64.7/16.3 | ICU | CAM-ICU |
| Arneson 2023 [6] | USA | Retrospective cohort | 441/526 | 83.35/6.68 | ED | bCAM |
| Avelino-Silva 2017[7] | Brazil | Prospective cohort | 549/860 | 80/9 | Geriatric ward | s-CAM |
| Avelino-Silva 2018[8] | Brazil | Prospective cohort | 549/860 | 80/9 | Geriatric ward | CAM |
| Bagienski 2017[9] | Poland | Prospective cohort | 52/89 | 81.47/5.61 | Cardiac surgery | CHART-DEL |
| Bakker 2012[10] | Netherlands | Prospective cohort | 121/80 | 76.1/NA | Cardiothoracic surgery | CAM-ICU |
| Beishuizen 2020[11] | Netherlands | Prospective cohort | 37/89 | 80.89/NA | Cardiology unit | DSM-V |
| Bellelli 2007[12] | Italy | Prospective cohort | 40/148 | NA/NA | Post acute rehabilitation facility | CAM |
| Bellelli 2008[13] | Italy | Prospective cohort | 373/950 | 76.6/10.5 | Rehabilitation and Aged Care Unit | CAM |
| Bellelli 2018[14] | Italy | Prospective cohort | 93/146 | NA/NA | Orthogeriatric Unit | DSM-V  s-CAM  4AT |
| Bickel 2008[15] | Germany | Prospective cohort | 61/139 | 73.8/9 | Surgery | CAM |
| Brown 2018[16] | USA | Prospective cohort | 107/35 | 70/8 | Surgery | CAM  CAM-ICU  Medical records |
| Brown 2020[17] | Canada | Retrospective cohort | 2744/1293 | 58.94/18.53 | ICU | ICDSC |
| Bruck 2018[18] | Sweden | Prospective cohort | 76/49 | 60.12/NA | ICU | CAM-ICU |
| Bryson 2011[19] | Canada | Prospective cohort | 64/23 | 71.14/NA | Surgery | CAM |
| Bulic 2020[20] | Australia | Prospective cohort | 53/50 | 60/16 | ICU | CAM-ICU |
| Buurman 2011[21] | Netherlands | Prospective cohort | 295/344 | 78.2/7.8 | Internal medicine wards | CAM |
| Cartei 2022[22] | Italy | Prospective cohort | 108/279 | 82/8 | Trauma and Orthopaedics Centre | CAM |
| Cavallari 2017[23] | USA | Prospective cohort | 45/68 | 76/5 | Surgery | CAM  Chart review |
| Chalmers 2021[24] | UK | Prospective cohort | 1093/1378 | 85.35/6.67 | Acute geriatric medicine service | 4AT |
| Chan 2016[25] | Hong Kong | Prospective cohort | 134/19 | 74.2/11.1 | Respiratory wards | DSM-IV |
| Chan 2017[26] | Australia | Prospective cohort | NA | NA/NA | Stroke unit | DSM-IV |
| Cheong 2021[28] | Malaysia | Prospective cohort | 207/240 | 73.23/5.94 | Operating theatre and surgical wards | 4AT  CAM |
| Chen 2017[27] | China | Prospective cohort | 104/32 | 60.85/NA | Cardiac surgery (CABG) | CAM-ICU |
| Cirbus 2019[29] | USA | Prospective cohort | 102/126 | 74.64/NA | Hospitalized from ED | bCAM  CAM-ICU |
| Cole 2008[30] | Canada | Prospective cohort | 71/139 | 83.48/NA | Medical or geriatric services | CAM |
| Curyto 2001[31] | USA | Prospective cohort | 39/63 | 83.3/NA | Residential care facility | DSM-III-R |
| Czyzycki 2022[32] | Poland | Prospective cohort | 323/375 | NA/NA | Department of Neurology | DSM-5 |
| Daiello 2019[33] | USA | Retrospective cohort | 231/320 | 77/5 | Surgery | CAM  chart review |
| Dani 2018[34] | UK | Prospective cohort | 291/419 | 83.1/7.41 | Acute medical unit | CAM  Medical chart |
| Davis 2012[35] | Finland | Prospective cohort | 113/440 | 88/NA | Community | DSM-III-R |
| Davis 2014[36] | UK | Prospective cohort | 794/1403 | 77.35/9.64 | Community | DSM-IV |
| DeCrane 2011[37] | USA | Prospective cohort | 71/249 | 86.75/NA | Long-Term Care Facilities | CAM  NEECHAM  CAC-A  Vigilance A  MMSE |
| Decrane 2012[38] | USA | Prospective cohort | 71/249 | 86.75/NA | Long-term care facilities | CAM  NEECHAM  CAC-A  Vigilance A  MMSE |
| DeHaan 2023 [39] | Netherlands | Prospective cohort | 656/1395 | 80/10 | Geriatric trauma unit/orthopedic trauma ward | DSM-V |
| DeJong 2019[40] | Netherlands | Prospective cohort | 153/310 | 81/8 | Geriatric trauma unit/orthopedic trauma ward | CAM  DOS  Clinical evaluation |
| DelaVarga-Martínez 2023[41] | Spain | Prospective cohort | NA | 74.3/NA | ICU | CAM-ICU |
| Ditzel 2023[42] | Netherlands & Germany | Prospective cohort | 161/85 | 71/4.48 | Surgery | CAM-ICU  Nu-DESC  Chart review |
| Diwell 2018[43] | UK | Prospective cohort | 250/360 | 83/7 | ED | s-CAM |
| Dostovic 2021[44] | Bosnia and Herzegovina | Prospective cohort | NA | NA/NA | Medical admission-stroke | DSM-IV  DRS-R-98 |
| Drews 2015[45] | Germany | Prospective cohort | 304/255 | NA/NA | Surgery | CAM |
| Dros 2020[46] | Poland | Prospective cohort | 202/221 | 69.4/NA | Stroke unit | bCAM  CAM-ICU  DSM-5 |
| Duppils 2004[47] | Sweden | Prospective cohort | NA | 83.1/NA | surgery | DSM IV |
| Duprey 2020[48] | Netherlands | Prospective cohort | NA | 66.3/12.6 | ICU | CAM-ICU |
| Durlach 2023[49] | Argentina | Prospective cohort | 128/142 | 67.83/47.73 | ICU | CAM-ICU |
| Edelstein 2004[50] | USA | Prospective cohort | 729/192 | NA/NA | Surgery-hip fracture | DSM-IV  Chart notes |
| Edlund 2006[51] | Sweden | Prospective cohort | 177/223 | 80.15/NA | General internal medicine wards | DSM-IV  OBS scale |
| Eeles 2010[52] | UK | Prospective cohort | 117/161 | 82.5/5.6 | Medical admission unit | DSM-IV  Medical notes |
| Eeles 2012[53] | UK | Prospective cohort | 112/161 | 82.3/7.5 | Medical admission unit | DSM-IV |
| Eide 2016[54] | Norway | Prospective cohort | 60/76 | 83.5/2.7 | surgery | CAM |
| Eide2016_1[55] | Norway | Prospective cohort | 60/76 | 83.5/2.7 | surgery | CAM |
| Elsayem 2017[56] | USA | Prospective cohort | NA | 56.38/52.2 | ED | CAM  MDAS |
| Evensen 2021[57] | Norway | Prospective cohort | 89/139 | 86.6/5.2 | Medical geriatric ward | 4AT  DSM-V |
| Falsini 2018[58] | Italy | Prospective cohort | 413/313 | 79.1/7.8 | Cardiac intensive care units | CAM |
| FialhoSilva 2021[59] | Brazil | Prospective cohort | 121/106 | 62.5/13.5 | Stroke Unit | CAM-ICU  RASS |
| Fick 2013[60] | USA | Prospective cohort | 80/57 | 83/7 | Acute care hospitalization | CAM |
| Francis 1990[61] | USA | Prospective cohort | 85/144 | 78/NA | Medical ward | DSM III-R |
| Francis 1992[62] | USA | Prospective cohort | 79/126 | 77.96/NA | General medical wards | DSM-III-R |
| Franck 2016[63] | Germany | Prospective cohort | 461/389 | 69.6/6.3 | Post-anaesthesia care unit | DSM-IV-TR |
| Furlaneto 2007[64] | Brazil | Prospective cohort | 14/71 | 80.26/NA | Geriatric orthopaedic ward | CAM |
| Gandossi 2023[65] | Italy | Prospective cohort | 241/743 | 84/7.45 | Orthogeriatric centres | 4AT |
| Garcez 2019[66] | Brazil | Retrospective cohort | 123/186 | 78/9 | Geriatric ward | s-CAM |
| George 1997[67] | UK | Prospective cohort | 115/151 | 80.6/NA | Geriatric, medical, surgical, and orthopaedic wards | DSM III |
| Giroux 2021[68] | Canada | Prospective cohort | 300/308 | 76.8/7.8 | ED | CAM |
| Givens 2008[69] | USA | Prospective cohort | 27/99 | 79/8 | Surgery | CAM |
| Givens 2009[70] | USA | Prospective cohort | 182/277 | 80/6.5 | General medical service | CAM |
| Gleason 2015[71] | USA | Prospective cohort | 236/330 | 76.7/5.2 | Surgery | CAM |
| Gonçalves 2023[72] | Brazil | Prospective cohort | 1089/789 | 66.7/10.4 | COVID-19 admission unit | CAM |
| Gonzale 2005[73] | Spain | Prospective cohort | 65/84 | 78.18/NA | Medical and trauma wards | DSM-IV  CAM |
| Gonzalez 2009[74] | Spain | Prospective cohort | 208/334 | 77.9/7.6 | General medical ward | CAM |
| Gottschalk 2015[75] | USA | Prospective cohort | 123/336 | 81.3/7.1 | Surgery-hip fracture | CAM |
| Gou 2021[76] | USA | Prospective cohort | 216/281 | 76.8/5.1 | Surgery | CAM |
| Goudzwaard 2020[77] | Netherlands | Prospective cohort | 297/246 | 79.1/8 | Cardiac surgery | DSM-IV |
| Gual 2018[78] | Spain | Prospective cohort | 364/545 | 85.8/6.7 | Surgery | CAM |
| Guenther 2020[79] | Germany | Prospective cohort | 94/31 | 70.71/NA | ICU | CAM-ICU |
| Han 2010[80] | USA | Prospective cohort | 263/365 | 75/8.91 | ED | CAM-ICU |
| Han 2017[81] | USA | Prospective cohort | 430/499 | 74.35/8.16 | ED | CAM-ICU |
| Han 2022[82] | USA | Prospective cohort | 582/462 | 61.35/14.11 | ED | bCAM |
| Hapca 2018[83] | UK | Prospective cohort | 2940/3784 | 79.2/NA | Acute hospital admission | OPRAA  CAM |
| Hawley 2023[84] | UK | Prospective cohort | 19230/44272 | 81/8.7 | Surgery-hip fracture | 4AT |
| Hempenius 2016[85] | Netherlands | Randomized controlled trial | 99/161 | 77.4/NA | Surgery | DSM IV  DOS |
| Hofhuis 2022[86] | Netherlands | Prospective cohort | 586/435 | 69.5/NA | Medical-surgical ICU | CAM-ICU |
| Hölttä 2011[87] | Finland | Prospective cohort | 37/218 | 86.4/NA | Acute geriatric wards and nursing homes | DSM-IV |
| Honda 2016[88] | Japan | Retrospective cohort | 373/238 | 75.2/12.3 | Cardiac admission unit | ICDSC |
| Hoogma 2023[89] | Belgium | Prospective cohort | 148/102 | 80/NA | Cardiac surgery | CAM |
| Hshieh 2017[90] | USA | Prospective cohort | 236/330 | 77/5.2 | Surgery | CAM |
| Hshieh 2023[91] | USA | Prospective cohort | 135/176 | 80.3/6.8 | General medicine | CAM  chart review |
| Hughes 2021[92] | USA | Prospective cohort | 627/413 | 62.35/14.1 | Medical and surgical ICU | CAM-ICU |
| Humbert 2021[93] | Switzerland | Prospective cohort | 51/42 | NA/NA | Surgery-cardiac | CAM |
| Inouye 1998[94] | USA | Prospective cohort | 291/436 | 78.9/6.9 | Non-intensive care wards | CAM |
| Inouye 2016[95] | USA | Prospective cohort | 235/325 | 76.7/5.2 | Surgery | CAM |
| Isaia 2009[96] | Italy | Prospective cohort | 38/106 | 85.5/NA | Geriatric home hospitalisation service and geriatric hospital ward | CAM |
| Iwata 2020[97] | Japan | Prospective cohort | 214/194 | 79.72/NA | ICU | CAM-ICU |
| Jackson 2014[98] | USA | Prospective cohort | 420/401 | 61/14.87 | Medical-surgical ICU | CAM-ICU |
| Jankowski 2011[99] | USA | Prospective cohort | 206/212 | 72.1/NA | Surgery | CAM |
| Janssen 2021[100] | Belgium | Prospective cohort | 171/94 | 76.7/5.98 | Surgery | DOSS  DSM-V |
| Juliebø 2010[101] | Norway | Prospective cohort | 82/249 | 83.3/6.9 | Orthopaedic departments | CAM |
| Kainz 2022[102] | Germany | Prospective cohort | 222/0 | 67.09/NA | Post-anaesthesia care unit | CAM-ICU |
| Kakuma 2003[103] | Canada | Prospective cohort | 40/67 | 80.1/7.9 | ED | CAM |
| Kat 2008[104] | Netherlands | Prospective cohort | 30/82 | 82.7/NA | Surgery | CAM  DSM-IV |
| Kat 2011[105] | Netherlands | Prospective cohort | 138/465 | 77.9/6 | Surgery | CAM |
| Katz 2001[106] | USA | Prospective cohort | 33/63 | 84.7/NA | Nursing home and congregate apartment | DSM-III-R |
| Kennedy 2014[107] | USA | Prospective cohort | 328/348 | 77/8 | ED | CAM |
| Kilicaslan 2022[108] | Turkey | Prospective cohort | 192/223 | 74/8.92 | ED | CAM |
| Knauf 2019[109] | Germany | Prospective cohort | 109/286 | 81/8.3 | ICU | CAM-ICU |
| Korber 2021[110] | Germany | Prospective cohort | 103/74 | 77.29/7.47 | Surgery | RASS  CAM-ICU |
| Koster 2012[111] | Netherlands | Prospective cohort | 204/96 | 70.5/9.3 | Department of thoracic surgery | DOSS |
| Kotfis 2019[112] | Poland | Prospective cohort | 393/367 | 71.63/NA | Stroke unit | CAM-ICU |
| Krogseth 2011[113] | Norway | Prospective cohort | 27/79 | 82.7/6.9 | Orthopaedic departments | CAM |
| Krogseth 2014[114] | Norway | Prospective cohort | 56/151 | 82.6/7.1 | Orthopaedic departments | CAM |
| Krogseth 2016[115] | Norway | Prospective cohort | 74/213 | 80.79/31.29 | Orthopaedic departments | CAM |
| Krogseth 2023[116] | Norway | Prospective cohort | 72/138 | 84.5/8.3 | Domiciliary care services | DSM-V |
| Krzych 2014[117] | Poland | Prospective cohort | 4031/1750 | NA/NA | Surgery-cardiac | DSM-IV |
| Kunicki 2023[118] | USA | Prospective cohort | 234/326 | 76.7/5.2 | Surgery | CAM  Medical record |
| Labaste 2020[119] | France | Prospective cohort | 125/48 | 73.29/8.97 | Cardiovascular surgery department | CAM-ICU |
| Labaste 2023[120] | France | Prospective cohort | 12/51 | 87.11/6.44 | Surgery-hip fracture | CAM |
| Large 2013[121] | USA | Prospective cohort | 40/9 | 74.68/NA | Urology clinic | CAM |
| Lee 2011[122] | South Korea | Prospective cohort | 59/173 | 79/7.7 | Surgery-hip fracture | CAM |
| Lee 2017[123] | USA | Prospective cohort | 123/343 | 80.8/7 | Surgery | CAM |
| Lee 2018[124] | Hong-Kong | Prospective cohort | 413/187 | NA/NA | ICU | CAM-ICU |
| Leslie 2005[125] | USA | Prospective cohort | 365/554 | 80/6.5 | General medical units | CAM |
| Leslie 2008[126] | USA | Prospective cohort | 329/512 | 80.2/6.4 | General medical units | CAM |
| Lewis 1995[127] | USA | Prospective cohort | NA | NA/NA | ED | CAM |
| Li 2019[128] | China | Prospective cohort | 58/53 | 68.95/12.16 | Department of cardiology | CAM-ICU |
| Li 2021[129] | Taiwan | Prospective cohort | 195/103 | 61.9/12.5 | Surgery-cardiac | CAM |
| Liang 2014[130] | Taiwan | Prospective cohort | 108/124 | 74.7/7.8 | Orthopaedic surgery | CAM |
| Lima 2010[131] | Brazil | Prospective cohort | 93/106 | 77.9/NA | Geriatric Unit | DSM-IV |
| Lingehall 2017[132] | Sweden | Prospective cohort | 79/35 | 76.5/4.4 | Cardiac surgery | DSM-IV-TR |
| Lundström 2003[133] | Sweden | Prospective cohort | 18/60 | 79.1/8.1 | Orthopaedic Surgery | DSM-IV |
| Luz 2020[134] | Brazil | Prospective cohort | 120/96 | 65.25/NA | ICU | CAM-ICU |
| Maclullich 2019[135] | UK | Prospective cohort | 349/436 | 81.4/6.4 | ED and  acute general medical wards | 4AT  CAM  DSM-4  DRS-R98 |
| Marcantonio 2005[136] | USA | Prospective cohort | 156/340 | NA | Skilled nursing facilities | CAM |
| Mariz 2013[137] | Portugal | Prospective cohort | 131/107 | 61.7 | ED | CAM-ICU |
| Marrama 2022[138] | France | Prospective cohort | 134/114 | 70.4/12.7 | ED | DSM-IV  DRS |
| Mathies 2020[139] | Germany | Prospective cohort | 23/45 | 81.5/5.7 | Geriatric unit | Nu-DESC  DRS |
| Mauri 2021[140] | Germany | Prospective cohort | 322/339 | 82.3/6.6 | Surgery-cardiac | CAM-ICU |
| Mazzola 2015[141] | Italy | Prospective cohort | 40/235 | 89.4/3.8 | Orthogeriatric Unit | DSM-IV-TR |
| McAvay 2006[142] | USA | Prospective cohort | 172/261 | 79.8/6.3 | General medicine service | CAM |
| McCusker 2001[143] | Canada | Prospective cohort | 117/198 | NA/NA | ED | CAM |
| McCusker 2002[144] | Canada | Prospective cohort | 128/233 | NA/NA | Medical services unit | CAM |
| McCusker 2014[145] | Canada | Prospective cohort | 120/154 | NA/NA | Long-term care | CAM |
| Minden 2005[146] | USA | Prospective cohort | 31/4 | 67.5/8 | Surgery | CAM  MDAS |
| Miu 2013[147] | Hong Kong | Prospective cohort | 163/151 | 72.9/10.3 | Acute stroke unit | CAM |
| Miyamoto 2021[148] | Japan | Prospective cohort | 121/83 | 71.29/15.09 | ICU | CAM-ICU |
| Monacelli 2012[149] | Italy | Prospective cohort | 58/160 | 86.54/6.02 | Orthogeriatric Unit | DSM-V  4AT |
| Moon 2018[150] | South Korea | Prospective cohort | 93/80 | 76/11.44 | Long-term care facilities | S-CAM |
| Morandi 2014[151] | Italy | Prospective cohort | 734/1908 | 77/8.9 | Rehabilitation unit | CAM  DSM-IV-TR |
| Moreno-Gavino 2012[152] | Spain | Prospective cohort | 760/674 | 77.9/9.8 | Medical admission unit | CAM |
| Moskowitz 2017[153] | USA | Prospective cohort | 167/5 | 64/8 | Surgery | CAM-ICU |
| Muller 2023[154] | Germany | Prospective cohort | 50/49 | 70.3/7 | Department of neurosurgery | Nu-DESC  DSM-V |
| Muresan 2016[155] | Ireland | Prospective cohort | 100/100 | 81.13/6.45 | Medical inpatients | CAM |
| Murray 1993[156] | USA | Prospective cohort | 96/195 | 80.5/7.6 | Medical admission unit | DSM-III  DSI  medical chart |
| Muzzana 2022[157] | Italy | Prospective cohort | 98/104 | 76.24/6.72 | Surgical and traumatological/  orthopaedic wards | DSM-V  DOSS  4A's |
| Naidech 2013[158] | USA | Prospective cohort | 62/52 | 63/13.8 | ICU | CAM-ICU |
| Neerland 2017[159] | Norway | Prospective cohort | 405/131 | 83.78/NA | Orthopaedic ward | CAM |
| Nerdal 2022[160] | Norway | Prospective cohort | 71/68 | 71.4/13.4 | Medical admission unit -cardiac | CAM |
| Neufeld 2015[161] | USA | Prospective cohort | 34/47 | 77.5/5.4 | Post-anaesthesia care unit | DSM IV |
| Nguyen 2018[162] | Canada | Prospective cohort | 137/60 | 68.88/11.57 | Surgery-cardiac | CAM-ICU  CAM |
| Nishizawa 2023[163] | Japan | Retrospective cohort | 532/545 | 67.6/14 | Hospital or emergency room | CAM-ICU  DRS  DOSS |
| Noriega 2015[164] | Spain | Prospective cohort | 103/100 | 81.6/4.7 | Cardiology unit | CAM |
| Ogawa 2017[165] | Japan | Prospective cohort | 183/143 | 68.6/14.8 | Surgery-cardiac | ICDSC |
| Ojagbemi 2020[166] | Nigeria | Prospective cohort | 89/61 | 60.1/12.7 | Medical admission unit-Stroke | CAM  DSM V |
| Ojagbemi 2021[167] | Nigeria | Prospective cohort | 89/61 | 60.2/12.8 | Medical admission unit -Stroke | CAM  DSM V |
| O'Keeffe 1997[168] | Ireland | Prospective cohort | 146/79 | 82/NA | Geriatric unit | DSM III |
| Oldenbeuving 2011[169] | Netherlands | Prospective cohort | 288/239 | 65.34/49.8 | Stroke units | CAM |
| Olofsson 2018[170] | Sweden | Prospective cohort | 34/101 | 82.17/NA | Femoral neck surgery | Modified OBS scale  DSM‐IV‐TR |
| Pak 2020[171] | Japan | Prospective cohort | 68/64 | 81.59/8.99 | Medical admission unit -cardiac | DSM-V |
| Pandharipande 2013[172] | USA | Prospective cohort | 234/233 | 59.59/14.86 | Medical or surgical ICU | CAM-ICU |
| Pasinska 2019[173] | Poland | Prospective cohort | 352/398 | 71.8/13.1 | Medical admission unit -stroke | bCAM  CAM-ICU  DSM-5 |
| Patel 2014[174] | USA | Prospective cohort | 57/45 | 59.62/15.72 | Medical ICU | CAM-ICU |
| Paulino 2023[175] | Portugal | Prospective cohort | 60/46 | NA/NA | Admitted to medical and surgical ICU | ICDSC |
| Pendlebury 2015[176] | UK | Prospective cohort | 236/267 | 61.83/61.84 | Acute general medicine | CAM  DSM-IV |
| Penfold 2023[177] | UK | Retrospective cohort | 372/968 | 79.5/10.3 | ED and orthopaedic ward | 4AT |
| Pitkala 2005[178] | Finland | Prospective cohort | 78/347 | NA/NA | Acute geriatric wards and nursing homes | DSM IV |
| Pompei 1994[179] | USA | Prospective cohort | 190/242 | 74.35/NA | Medical and surgical wards | DSM-III-R |
| Praditsuwan 2013[180] | Thailand | Prospective cohort | 114/111 | 78/5.9 | General medical wards | DSM-IV |
| Qu 2018[181] | China | Prospective cohort | 184/77 | 61.3/14.8 | Medical admission unit -Stroke | CAM |
| Quinlan 2011[182] | Denmark, France, Germany, Great Britain, Greece, Netherlands, Spain, USA | Prospective cohort | 484/464 | 68.62/NA | Surgery-non-cardiac | Medical record  DSM-III |
| Raats 2015[183] | Netherlands | Prospective cohort | 148/103 | NA/NA | Surgery | DOSS |
| Racine 2018[184] | USA | Prospective cohort | 234/326 | 76.7/5.2 | Surgery | CAM |
| Racine 2020[185] | USA | Prospective cohort | 55/85 | 76.1/4.5 | Surgery | CAM  chart review |
| Radcliffe 2023[186] | Australia | Prospective cohort | 114/156 | 86.68/NA | Aged care | CAM |
| Radinovic 2014[187] | Serbia | Prospective cohort | 71/206 | 78/8.2 | Surgery-hip fracture | CAM |
| Rahkonen 2001[188] | Finland | Prospective cohort | 46/153 | 87.3/2.4 | Community | DSM-III-R |
| Rawle 2021[189] | UK | Prospective cohort | 237/340 | 83.2/7.4 | Acute medical unit | s-CAM |
| Rego 2023[190] | Brazil | Prospective cohort | 195/82 | 68.43/NA | ICU | CAM-ICU  CAM-ICU-7 |
| Reynish 2017[191] | UK | Prospective cohort | 9215/799 | 79.3/NA | Acute medical unit | CAM  Clinical diagnosis  AMT |
| Richardson 2021[192] | UK | Prospective cohort | 96/109 | 82/6.5 | Community | DSM V |
| Richardson 2021-2[193] | UK | Prospective cohort | 61/74 | 76.14/NA | Community | I-AGeD |
| Rizzi 2015[194] | Spain | Prospective cohort | 93/146 | 81.7/9.4 | ED | bCAM |
| Robinson 2009[195] | USA | Prospective cohort | NA | 64.55/NA | Surgery | CAM-ICU |
| Robinson 2011[196] | USA | Prospective cohort | 166/6 | 64/8 | Surgery | CAM-ICU |
| Rockwood 1999[197] | Canada | Prospective cohort | 87/116 | 79/NA | General medicine services | DSM-IV |
| Rolandi 2020[198] | Italy | Prospective cohort | 511/589 | NA/NA | Community | DSM-IV-TR |
| Rollo 2022[199] | Italy | Prospective cohort | 62/41 | 72.88/13.53 | Stroke unit | CAM-ICU |
| Rosenthal 2017[200] | USA | Prospective cohort | 92/82 | 63.5/NA | Neuro/ Spine ICU | CAM-ICU |
| Rudolph 2008[201] | Denmark, France, Germany, Great Britain, Greece, Netherlands, Spain, USA | Prospective cohort | 483/463 | 68.6/5.9 | Surgery-non-cardiac | DSM-III |
| Rudolph 2010[202] | USA | Prospective cohort | 150/40 | 73.7/6.7 | Cardiac surgery | CAM |
| Ruggiero 2017[203] | Italy | Prospective cohort | 116/398 | 83.1/7.3 | Orthopaedic and orthogeriatric wards | DSM-IV-TR |
| Saczynski 2012[204] | USA | Prospective cohort | 201/24 | 73/6.7 | Surgery-cardiac | CAM |
| Sánchez-Lozano 2023[205] | Colombia | Nested case–control | 27/54 | 61.28/NA | ICU | CAM-ICU |
| Sanguanwit 2023[206] | Thailand | Prospective cohort | 82/91 | 77.57/NA | ED | CAM-ICU |
| Sasajima 2012[207] | Japan | Prospective cohort | 261/38 | NA/NA | Surgery | CAM |
| Sato 2017[208] | Japan | Prospective cohort | 83/80 | 80.08/NA | Intensive cardiac care unit | CAM-ICU |
| Sauer 2017[209] | Netherlands | Prospective cohort | 127/49 | 66.56/NA | Surgery-cardiac | CAM  Chart review |
| Serrano-Duenas 2005[210] | Ecuador | Prospective cohort | 41/22 | NA/NA | Neurology Service | CAM |
| Sheng 2006[211] | Australia | Prospective cohort | 83/73 | 79.2/6.7 | Medical admission unit -stroke | DSM-IV |
| Shi 2019[212] | Canada, USA, France | Prospective cohort | 97/90 | 81.32/NA | Surgery-cardiac | DSI  CAM  CAM-ICU |
| Shi 2019_2[213] | China | Prospective cohort | 31/101 | 80/6 | Orthopaedic- surgery | CAM |
| Shim 2015[214] | USA | Prospective cohort | 314/317 | NA/NA | Surgery-non-cardiac | CAM |
| Shintani 2009[215] | USA | Prospective cohort | NA | NA/NA | Medical and coronary intensive care units | CAM-ICU |
| Singler 2014[216] | Germany | Prospective cohort | 53/80 | 83.4/5.5 | ED | CAM |
| Slor 2013[217] | Netherlands | Prospective cohort | 12/41 | 82.9/NA | Surgery-hip fracture | CAM |
| Sri-on 2016[218] | Thailand | Prospective cohort | 98/134 | 76/6 | ED | CAM-ICU |
| Suraarunsumrit 2022[219] | Thailand | Prospective cohort | 164/125 | 72/NA | Surgery | CAM-ICU  DSM-5 |
| Svenningsen 2014[220] | Denmark | Prospective cohort | 204/156 | 61/15 | ICU | CAM-ICU |
| Svenningsen 2015[221] | Denmark | Prospective cohort | 166/133 | 59.89/28.31 | ICU | CAM-ICU |
| Tahir 2018[222] | UK | Prospective cohort | 117/264 | NA/NA | Surgery-femoral neck | DSM-IV |
| Tan AH 2015[223] | New Zealand | Retrospective cohort | NA | NA/NA | Medical, orthopaedic and rehabilitation ward | CAM |
| Tavares 2021[224] | Portugal | Prospective cohort | 47/54 | 82.47/6.57 | Internal medicine services | CAM |
| To-Adithep 2023[225] | Thailand | Prospective cohort | 333/297 | 64.1/16.6 | SICU | CAM-ICU |
| Traissac 2011[226] | France | Prospective cohort | 154/322 | 86.5/6 | Geriatric unit | CAM |
| Trevisan 2023[227] | Italy | Prospective cohort | 842/482 | 66.7/14.6 | Hospitalized for COVID-19 | DSM-5 |
| Tripathy 2014[228] | India | Prospective cohort | 80/29 | 74.7/8.4 | Medical surgical ICU | ICDSC |
| Tsai 2012[229] | Taiwan | Prospective cohort | 320/294 | 74.7/6.4 | Psychiatric consultation | DSM-IV-TR |
| Uthamalingam 2011[230] | UK | Retrospective cohort | 422/461 | 79/8 | Medical admission unit -cardiac | CAM |
| VandenBoogaard 2012[231] | Netherlands | Prospective cohort | 609/306 | 69.55/20.7 | ICU | CAM-ICU  DSM-IV |
| VanderHeijden 2023[232] | Netherlands | Prospective cohort | 1651/749 | 63.3/12.5 | ICU | CAM-ICU |
| VanDerWulp 2019[233] | Netherlands | Prospective cohort | 338/365 | 79.64/6.68 | Surgery-cardiac | DSM-IV  CAM  DOS |
| van Eijsden 2015[234] | Netherlands | Prospective cohort | 53/39 | 76.35/8.28 | Vascular surgical ward | DOSS |
| vanRijsbergen 2011[235] | Netherlands | Nested case–control | 29/21 | 75.13/NA | Stroke unit | CAM |
| VanRompaey 2009[236] | Belgium | Prospective cohort | 66/49 | 62/NA | ICU | NEECHAM Confusion Scale  CAM-ICU |
| Vasunilashorn 2016[237] | USA | Prospective cohort | 601/884 | 78.75/NA | Surgical and medical | CAM  CAM-S |
| Vasunilashorn 2018[238] | USA | Prospective cohort | 240/326 | 76.7/5.2 | Surgery-non-cardiac | CAM  CAM-S |
| Vasunilashorn 2022[239] | USA | Prospective cohort | 148/204 | 79.94/8.11 | Medical or surgical services | CAM  Medical record |
| Veiga 2012[240] | Portugal | Prospective cohort | 413/267 | 64.1/NA | Post-anaesthesia Care Unit | ICDSC |
| Verloo 2016[241] | Switzerland | Prospective cohort | 40/74 | 83.2/7.2 | Hospital | CAM |
| Vida 2006[242] | Canada | Prospective cohort | 56/71 | 78.67/NA | ED | CAM |
| Visser 2015[243] | Netherlands | Prospective cohort | 356/107 | 71.64/8.18 | Surgery-cardiac | DOS  DSM-IV |
| Vives-Borrás 2019[244] | Spain | Prospective cohort | 322/205 | 84.45/3.71 | Medical admission unit -cardiac | CAM  DSM-IV |
| Wang 2021[245] | China | Prospective cohort | 127/0 | 70.6/0.4 | Surgery-laryngectomy | CAM |
| Weng 2019[246] | Taiwan | Prospective cohort | 62/87 | 81.8/7.8 | Geriatric ward | CAM |
| Whittamore 2014[247] | UK | Prospective cohort | 85/164 | 84/7.45 | Acute medical care | DRS-R-98  DSM-IV |
| Witlox 2013[248] | Netherlands | Prospective cohort | 12/36 | 83.16/NA | Hip fracture surgery | CAM |
| Wolters 2014[249] | Netherlands | Prospective cohort | 677424 | 59.8/16.5 | ICU | CAM-ICU |
| Wolters 2017[250] | Netherlands | Prospective cohort | 222/141 | NA/NA | Medical-surgical ICU | CAM-ICU |
| Zakriya 2004[251] | USA | Prospective cohort | 26/66 | 78.29/NA | Surgery-hip fracture | CAM |
| Ziman 2020[252] | USA | Prospective cohort | 663/662 | 72.7/6 | Surgery-non-cardiac | CAM |
| Zipprich 2020[253] | Germany | Prospective cohort | 288/303 | 74.1/8.4 | In-patient care, excluding psychiatric wards | CAM  CAM-ICU |

M/F = male/female. M/sd = mean/standard deviation. ICDSC = intensive care delirium screening checklist. CAM = confusion assessment method. CHART-DEL = Chart-based Delirium Identification Instrument. 4AT = rapid clinical test for delirium detection. DSM = Diagnostic and Statistical Manual of Mental Illness. DOSS/DOS = Delirium observation screening scale. I-AGeD = Informant Assessment Geriatric Delirium Scale. DRS-R-98/DRS = delirium rating scale-R-98. CAC-A = Clinical Assessment of Confusion-A. MMSE = Mini-mental state examination. MDAS = Memorial Delirium Assessment Scale. RASS = Richmond Agitation-Sedation Scale. Nu-DESC – Nursing Delirium Screening Scale. OBS = Overt Behaviour Scale. OPRAA = Older Persons Routine Acute Assessment. DSI = Delirium Symptom Interview. AMT = Abbreviated Mental Test, ED = Emergency Department

Footnote: Age: mean(SD), may be less than 60 when converted from median (IQR) to mean(sd).

# Table S6. Summary of included studies on long-term clinical outcomes of delirium after hospital discharge

| Clinical outcomes | Assessment | Outcome | Data type | Total number of studies | Follow-up sample | | Total sample size | Follow-up duration | | |
| --- | --- | --- | --- | --- | --- | --- | --- | --- | --- | --- |
|  |  |  |  |  | delirium | No-delirium |  | Median duration (month) | Min  (month) | Max  (month) |
| Cognition | Objective | Change | Categorical | 7 | 405 | 2383 | 2788 | 4.45 | 1 | 12 |
|  |  |  | Continuous | 13 | 825 | 1881 | 2706 | 11.82 | 1 | 72 |
|  |  | Performance | Categorical | 10 | 820 | 2085 | 2905 | 13.48 | 0.067 | 38 |
|  |  |  | Continuous | 36 | 2283 | 4318 | 6601 | 13.83 | 0.067 | 72 |
|  | Subjective | Performance | Categorical | 5 | 838 | 2651 | 3489 | 17.73 | 3 | 36 |
|  |  |  | Continuous | 7 | 599 | 1398 | 1997 | 6.46 | 2 | 18 |
|  | Total | | | 62 | 4923 | 12071 | 16994 |  |  |  |
| Functional outcome |  | Change | Categorical | 23 | 973 | 5867 | 6840 | 6.74 | 1 | 48 |
|  |  |  | Continuous | 10 | 357 | 1005 | 1362 | 7.20 | 1 | 36 |
|  |  | Performance | Categorical | 27 | 2942 | 23478 | 26420 | 7.95 | 0.067 | 60 |
|  |  |  | Continuous | 37 | 2498 | 6631 | 9129 | 7.87 | 0.067 | 60 |
|  |  | Total | | 80 | 6171 | 34615 | 40786 |  |  |  |
| Quality of life |  |  | Categorical | 4 | 674 | 2496 | 3170 | 5.4 | 3 | 12 |
|  |  |  | Continuous | 22 | 2248 | 5422 | 7670 | 7.02 | 1 | 36 |
|  |  | Total | | 24 | 2300 | 5890 | 8190 |  |  |  |
| Mental health |  |  | Categorical | 14 | 3196 | 5701 | 8897 | 8.65 | 0.067 | 36 |
|  |  |  | Continuous | 11 | 488 | 899 | 1387 | 10.80 | 1 | 36 |
|  |  | Total | | 19 | 3333 | 6219 | 9552 |  |  |  |
| Dementia |  | | Categorical | 23 | 3033 | 7627 | 10660 | 34.05 | 3 | 120 |
| Institutionalisation |  | | Categorical | 29 | 2990 | 24104 | 27094 | 13.12 | 1 | 60 |
| Readmission |  | | Categorical | 31 | 2617 | 8470 | 11087 | 4.21 | 1 | 18 |
| Mortality |  | | Categorical | 165 | 19079 | 67094 | 86173 | 14.19 | 0.16 | 168 |
| Total (follow-up) |  | |  | 253 | 29814 | 107583 | 137397 |  | | |
| Total (baseline) |  |  |  | 253 | 36489 | 160902 | 197391 |  |  |  |

Footnote: the total numbers is different because a single paper could report more than one outcome

# Table S7. Follow-up time analysis for long-term clinical outcomes of delirium after hospital discharge: Categorical data results

| **Clinical outcome** | **Sub-group**  **(number of studies)** | **Timepoint** | **OR** | **95% CI** | **p-value** | **I²** (%) | **τ²** |
| --- | --- | --- | --- | --- | --- | --- | --- |
| Cognition | *Objective cognition measure* | | | | | | |
|  | Cognitive change (k=7) | Collapsed | 1.575 | 1.227–2.022 | <0.001 | 23.0 | 0.024 |
|  | Cognitive change (k=7) | ≤6 months | 1.551 | 1.232–1.953 | <0.001 | 11.9 | 0.013 |
|  | Cognitive performance (k=10) | Collapsed | 2.437 | 1.575–3.883 | <0.001 | 72.2 | 0.355 |
|  | Cognitive performance (k=5) | ≤6 months | 1.789 | 1.028–3.114 | 0.040 | 56.4 | 0.216 |
|  | Cognitive performance (k=6) | >6–12 months | 3.050 | 1.558–5.974 | 0.001 | 75.4 | 0.512 |
|  | Cognitive performance (k=3) | >12 months | 7.395 | 2.303–23.748 | 0.001 | 77.6 | 0.803 |
|  | *Subjective cognition measure* | | | | | | |
|  | Cognitive performance (k=5) | Collapsed | 2.107 | 1.031–4.306 | 0.041 | 86.6 | 0.537 |
|  | Cognitive performance (k=2) | ≤6 months | 2.017 | 1.089–3.735 | 0.026 | 1.8 | 0.004 |
|  | Cognitive performance (k=2) | >6–12 months | 1.865 | 1.138–3.057 | 0.013 | 64.2 | 0.082 |
| Functional outcome | Functional change (k=23) | Collapsed | 2.038 | 1.742–2.384 | <0.001 | 20.1 | 0.028 |
|  | Functional change (k=17) | ≤6 months | 1.894 | 1.564–2.293 | <0.001 | 28.0 | 0.042 |
|  | Functional change (k=5) | >6–12 months | 2.734 | 2.015–3.709 | <0.001 | 0.0 | 0.000 |
|  | Functional change (k=2) | >12 months | 1.558 | 0.590–4.115 | 0.371 | 0.0 | 0.000 |
|  | Functional performance (k=27) | Collapsed | 2.187 | 1.687–2.836 | <0.001 | 90.8 | 0.363 |
|  | Functional performance (k=16) | ≤6 months | 1.560 | 1.175–2.070 | 0.002 | 86.5 | 0.227 |
|  | Functional performance (k=8) | >6–12 months | 2.318 | 1.674–3.210 | <0.001 | 75.0 | 0.127 |
|  | Functional performance (k=7) | >12 months | 4.077 | 1.680–9.894 | 0.002 | 89.3 | 1.250 |
|  | Frailty(k=4) | Collapsed | 2.547 | 0.862–7.528 | 0.091 | 93.4 | 1.078 |
|  | Falls (k=2) | Collapsed | 0.692 | 0.343–1.397 | 0.305 | 0.0 | 0.000 |
| Quality of life | Poor quality of life (k=4) | Collapsed | 2.131 | 1.693–2.682 | <0.001 | 0.0 | 0.00 |
|  | Poor quality of life (k=3) | ≤6 months | 1.804 | 1.237–2.631 | 0.002 | 0.0 | 0.00 |
| Mental health | Poor mental health (k=14) | Collapsed | 1.687 | 1.308–2.175 | <0.001 | 79.7 | 0.161 |
|  | Sleep related problems (k=3) | Collapsed | 2.724 | 1.105–6.176 | 0.030 | 69.7 | 0.442 |
|  | Depression/ depressive disorders (k=10) | Collapsed | 1.656 | 1.246–2.200 | <0.001 | 65.5 | 0.107 |
|  | Anxiety/anxiety disorders (k=6) | Collapsed | 1.600 | 1.211–2.114 | 0.001 | 41.8 | 0.041 |
|  | PTSD (k=7) | Collapsed | 1.406 | 1.085–1.823 | 0.010 | 30.4 | 0.036 |
|  | Poor mental health (k=11) | ≤6 months | 1.572 | 1.202–2.057 | 0.001 | 54.8 | 0.100 |
|  | Poor mental health (k=6) | >6–12 months | 1.663 | 1.189–2.327 | 0.003 | 72.4 | 0.104 |
|  | Poor mental health (k=2) | >12 months | 4.574 | 2.851–7.340 | 0.001 | 0.0 | 0.00 |
| Dementia | Dementia (k=23) | Collapsed | 5.373 | 3.307–8.732 | <0.001 | 88.2 | 1.112 |
|  | Dementia (k=6) | ≤6 months | 5.603 | 2.716–11.557 | <0.001 | 20.6 | 0.169 |
|  | Dementia (k=9) | >6–12 months | 4.089 | 1.926–8.681 | <0.001 | 84.1 | 0.926 |
|  | Dementia (k=12) | >12 months | 5.189 | 2.437–11.050 | <0.001 | 89.8 | 1.463 |
| Institutionalisation | Institutionalisation (k=29) | Collapsed | 2.801 | 2.144–3.659 | <0.001 | 74.9 | 0.335 |
|  | Institutionalisation (k=15) | ≤6 months | 2.729 | 1.814–4.105 | <0.001 | 75.5 | 0.403 |
|  | Institutionalisation (k=12) | >6–12 months | 3.262 | 2.058–5.171 | <0.001 | 74.1 | 0.474 |
|  | Institutionalisation (k=5) | >12 months | 2.570 | 1.612–4.097 | <0.001 | 50.7 | 0.135 |
| Readmission/revisit | Readmission/revisit (k=31) | Collapsed | 1.699 | 1.395–2.070 | <0.001 | 66.4 | 0.163 |
|  | Readmission/revisit (k=29) | ≤6 months | 1.734 | 1.387–2.168 | <0.001 | 67.3 | 0.203 |
|  | Readmission/revisit (k=4) | >6–12 months | 1.612 | 1.291–2.014 | <0.001 | 0.0 | 0.000 |
| Mortality | Mortality (k=165) | Collapsed | 2.548 | 2.339–2.776 | <0.001 | 82.4 | 0.190 |
|  | Mortality (k=103) | ≤6 months | 2.934 | 2.609–3.299 | <0.001 | 73.4 | 0.186 |
|  | Mortality (k=75) | >6–12 months | 2.384 | 2.117–2.685 | <0.001 | 73.9 | 0.161 |
|  | Mortality (k=42) | >12 months | 2.022 | 1.675–2.441 | <0.001 | 84.9 | 0.260 |

Footnote: Categorical data cognitive change is cognitive decline vs no cognitive decline results. Categorical data functional change is functional decline vs no functional decline results.

Categorical data cognitive performance is cognitive impairment vs no cognitive impairment results. Categorical data functional performance is functional impairment vs no functional impairment results

Data presented < 6 months, >6–12 months and > 12 months when available for 2 or more studies.

# Table S8. Follow-up time analysis for long-term clinical outcomes of delirium after hospital discharge. Continuous data results

| **Clinical outcome** | **Sub-group**  **(number of studies)** | **Timepoint** | **Hedges's g** | **95% CI** | **p-value** | **I²** (%) | **τ²** |
| --- | --- | --- | --- | --- | --- | --- | --- |
| Cognition | *Objective cognitive measure* | | | | | | |
|  | Cognitive change (k=13) | Collapsed | -0.192 | -0.285– -0.099 | <0.001 | 73.1 | 0.017 |
|  | Cognitive change (k=9) | ≤6 months | -0.158 | -0.258– -0.058 | 0.002 | 52.5 | 0.011 |
|  | Cognitive change (k=5) | >6–12 months | -0.052 | -0.254– 0.150 | 0.613 | 71.0 | 0.035 |
|  | Cognitive change (k=4) | >12 months | -0.154 | -0.302– -0.006 | 0.042 | 73.5 | 0.015 |
|  | Cognitive performance (k=36) | Collapsed | -0.689 | -0.822– -0.555 | <0.001 | 94.7 | 0.141 |
|  | Cognitive performance (k=28) | ≤6 months | -0.657 | -0.810– -0.505 | <0.001 | 91.5 | 0.136 |
|  | Cognitive performance (k=17) | >6–12 months | -0.601 | -0.790– -0.411 | <0.001 | 88.2 | 0.127 |
|  | Cognitive performance (k=10) | >12 months | -0.782 | -1.104– -0.460 | <0.001 | 96.9 | 0.242 |
|  | *Subjective cognitive measure* | | | | | | |
|  | Cognitive performance (k=7) | Collapsed | -0.191 | -0.336– -0.046 | 0.010 | 72.6 | 0.025 |
|  | Cognitive performance (k=5) | ≤6 months | -0.254 | -0.405– -0.103 | 0.001 | 48.2 | 0.014 |
| Functional outcome | Functional change (k=10) | Collapsed | -0.432 | -0.694– -0.170 | 0.001 | 87.3 | 0.143 |
|  | Functional change (k=7) | ≤6 months | -0.433 | -0.764– -0.102 | 0.010 | 90.3 | 0.172 |
|  | Functional change (k=2) | >12 months | -0.279 | -0.887– 0.329 | 0.368 | 76.7 | 0.149 |
|  | Functional performance (k=37) | Collapsed | -0.616 | -0.827– -0.405 | <0.001 | 97.7 | 0.404 |
|  | Functional performance (k=30) | ≤6 months | -0.608 | -0.866– -0.349 | <0.001 | 97.6 | 0.502 |
|  | Functional performance (k=13) | >6–12 months | -0.581 | -0.892– -0.270 | <0.001 | 95.6 | 0.297 |
|  | Functional performance (k=7) | >12 months | -0.544 | -0.851– -0.237 | 0.001 | 92.2 | 0.144 |
| Quality of life | Poor quality of life (k=22) | Collapsed | -0.443 | -0.563– -0.323 | <0.001 | 93.6 | 0.068 |
|  | Poor quality of life (k=14) | ≤6 months | -0.331 | -0.465– -0.196 | <0.001 | 88.8 | 0.051 |
|  | Poor quality of life (k=8) | >6–12 months | -0.419 | -0.613– -0.225 | <0.001 | 89.5 | 0.062 |
|  | Poor quality of life (k=2) | >12 months | -0.448 | -0.905– 0.008 | 0.054 | 87.5 | 0.096 |
|  | Poor quality of life: physical (k=5) | Collapsed | -0.242 | -0.367– -0.117 | <0.001 | 48.5 | 0.010 |
|  | Poor quality of life: mental (k=5) | Collapsed | -0.191 | -0.266– -0.115 | <0.001 | 0.0 | 0.00 |
|  | Poor quality of life: role physical (k=5) | Collapsed | -0.351 | -0.629– -0.073 | 0.013 | 79.6 | 0.068 |
|  | Poor quality of life: physical functioning (k=8) | Collapsed | -0.619 | -0.934– -0.304 | <0.001 | 87.7 | 0.159 |
|  | Poor quality of life: vitality (k=6) | Collapsed | -0.231 | -0.424– -0.037 | 0.020 | 65.4 | 0.034 |
|  | Poor quality of life: social functioning (k=9) | Collapsed | -0.588 | -0.888– -0.289 | <0.001 | 87.6 | 0.163 |
|  | Poor quality of life: general health (k=7) | Collapsed | -0.332 | -0.619– -0.045 | 0.023 | 84.0 | 0.110 |
|  | Poor quality of life: role emotional (k=6) | Collapsed | -0.129 | -0.275– 0.017 | 0.084 | 39.4 | 0.012 |
|  | Poor quality of life: bodily pain (k=5) | Collapsed | -0.048 | -0.182– 0.087 | 0.488 | 33.6 | 0.008 |
|  | Poor quality of life: mental health (k=6) | Collapsed | -0.178 | -0.428– 0.072 | 0.162 | 70.1 | 0.062 |
| Mental health | Poor mental health (k=11) | Collapsed | -0.328 | -0.488– -0.168 | <0.001 | 76.1 | 0.050 |
|  | Depression/depressive disorders (k=9) | Collapsed | -0.354 | -0.576– -0.133 | 0.002 | 70.7 | 0.072 |
|  | Anxiety/ anxiety disorders (k=5) | Collapsed | -0.270 | -0.520– -0.021 | 0.034 | 61.9 | 0.047 |
|  | Post-traumatic stress/post-traumatic stress disorder (PTSD) (k=4) | Collapsed | -0.135 | -0.289– -0.018 | 0.084 | 6.4 | 0.002 |
|  | Other mental/behavioural symptoms (k=3) | Collapsed | -0.317 | -0.519– -0.115 | 0.002 | 0.0 | 0.00 |
|  | Poor mental health (k=10) | ≤6 months | -0.313 | 0.493– -0.133 | 0.001 | 71.8 | 0.052 |
|  | Poor mental health (k=5) | >6–12 months | -0.411 | -0.650– -0.173 | 0.001 | 24.1 | 0.018 |
|  | Poor mental health (k=2) | >12 months | -0.460 | -0.726– -0.194 | 0.001 | 0.0 | 0.000 |

Footnote: Continuous data cognitive change is a change from the baseline results. Continuous data functional change is a change from the baseline results

Continuous data cognitive performance is a score at follow-up results. Continuous data functional performance is a score at follow-up results

Data presented < 6 months, >6–12 months and > 12 months when available for 2 or more studies.

# Table S9. Forest plots for long-term clinical outcomes of delirium after hospital discharge

| **Clinical outcomes** | **Data type** | **Timepoints** | **Forest Plot** |
| --- | --- | --- | --- |
| **Objective cognition (change)** | Cat | Collapsed | **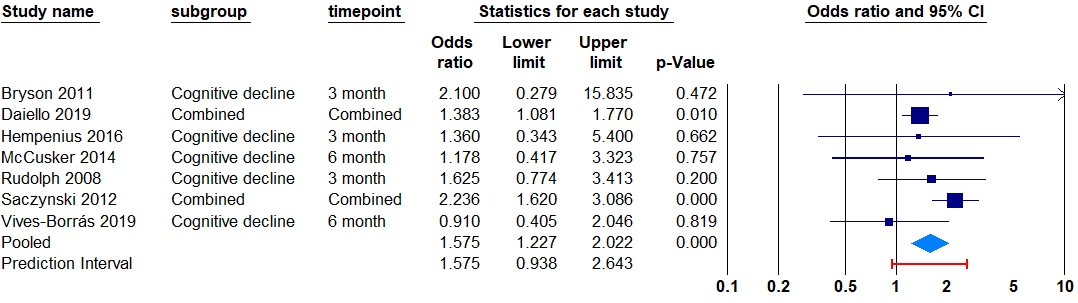** |
| **Objective cognition (change)** | Cat | ≤6 months | **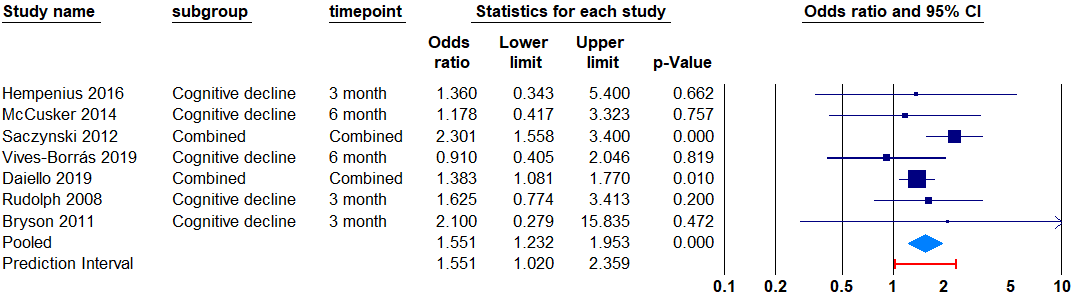** |
| **Objective cognition (change)** | Con | Collapsed | **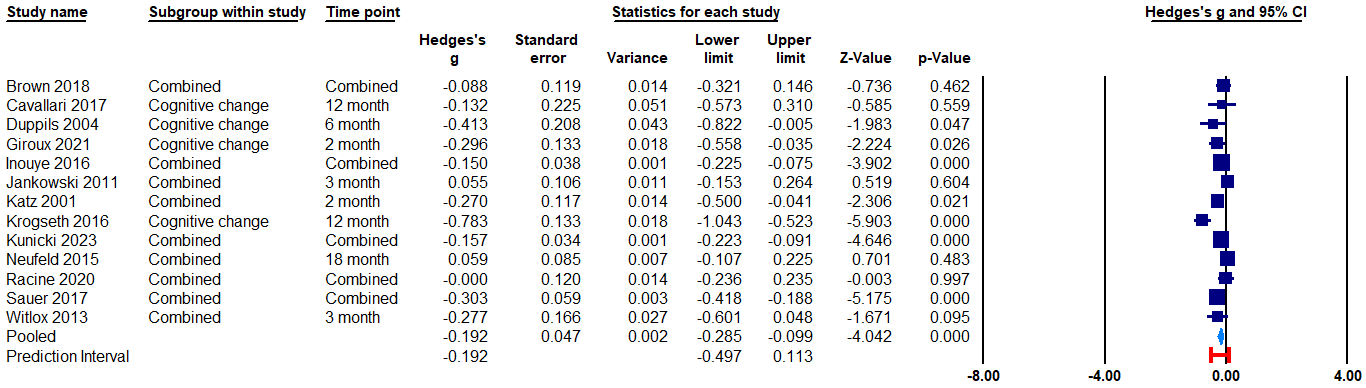** |
| **Objective cognition (change)** | Con | ≤6 months | **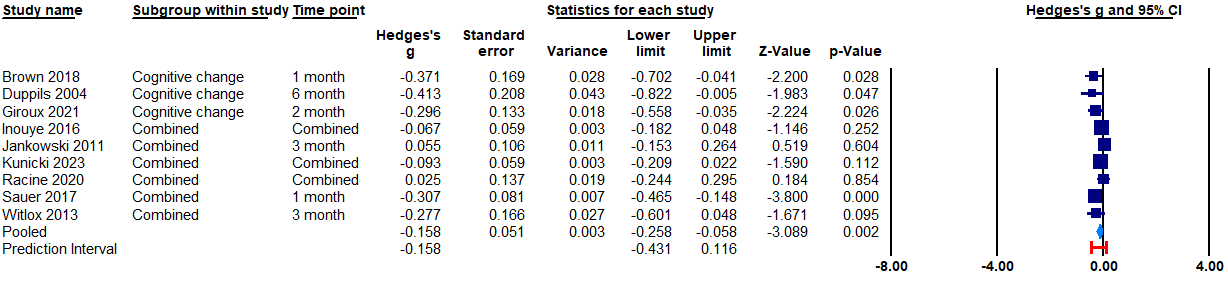** |
| **Objective cognition (change)** | Con | >6-12 months | **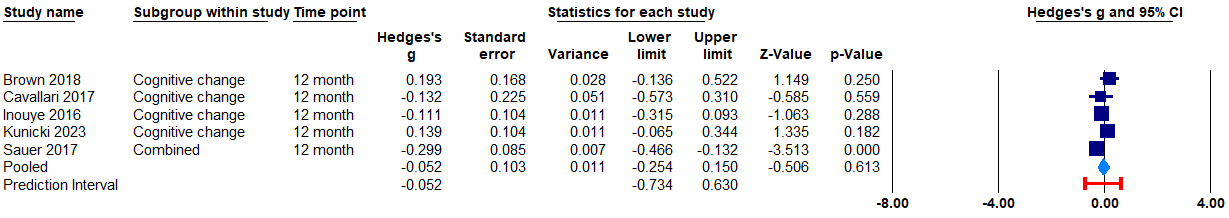** |
| **Objective cognition (change)** | Con | >12 months | **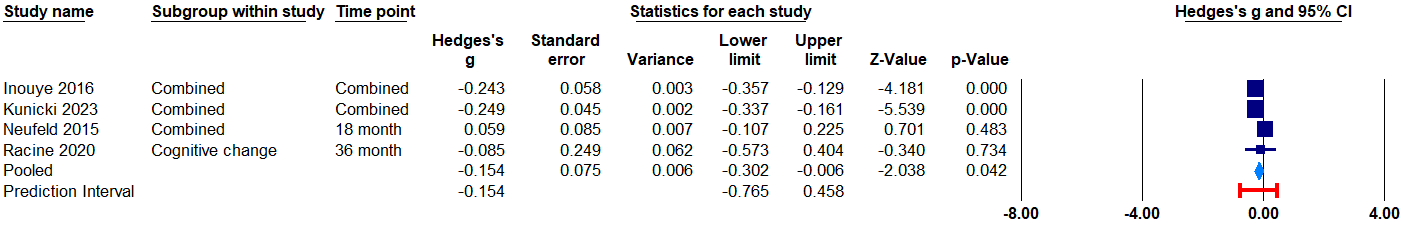** |
| **Objective cognition**  **(performance)** | Cat | Collapsed | **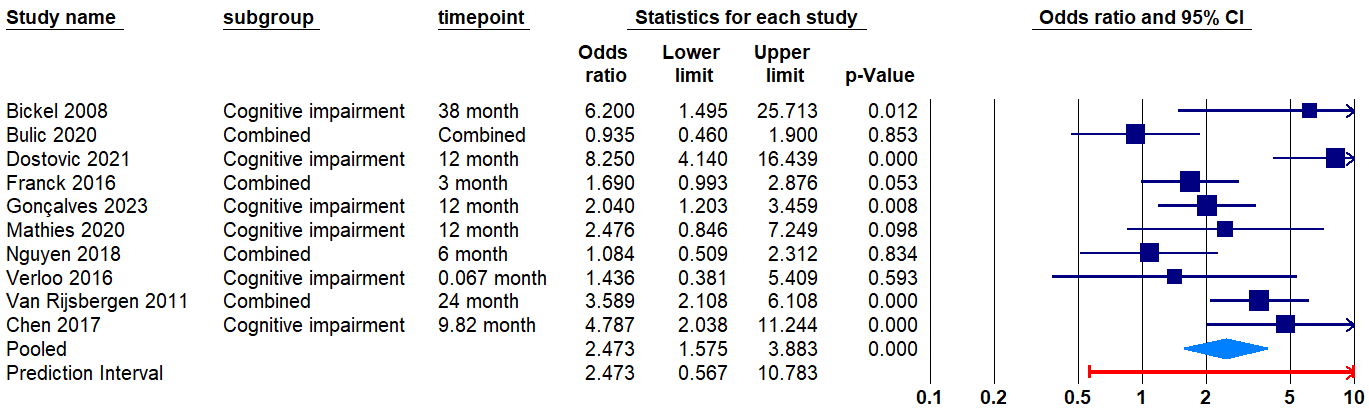** |
| **Objective cognition**  **(performance)** | Cat | ≤6 months | **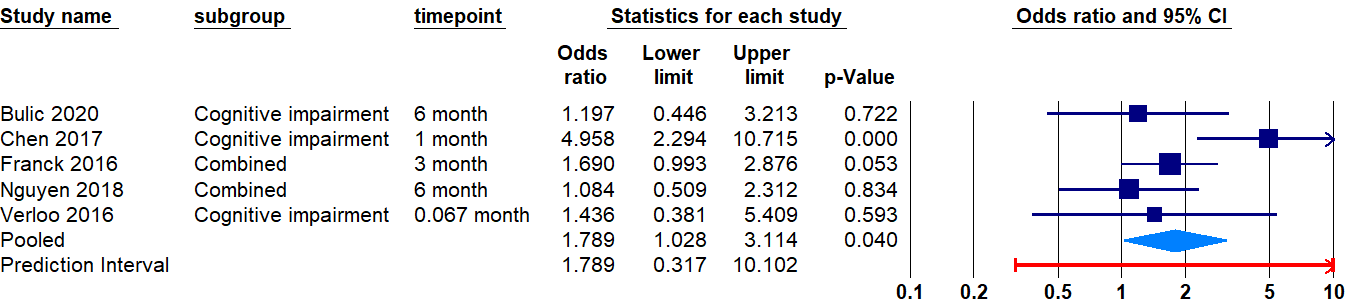** |
| **Objective cognition**  **(performance)** | Cat | >6-12 months | **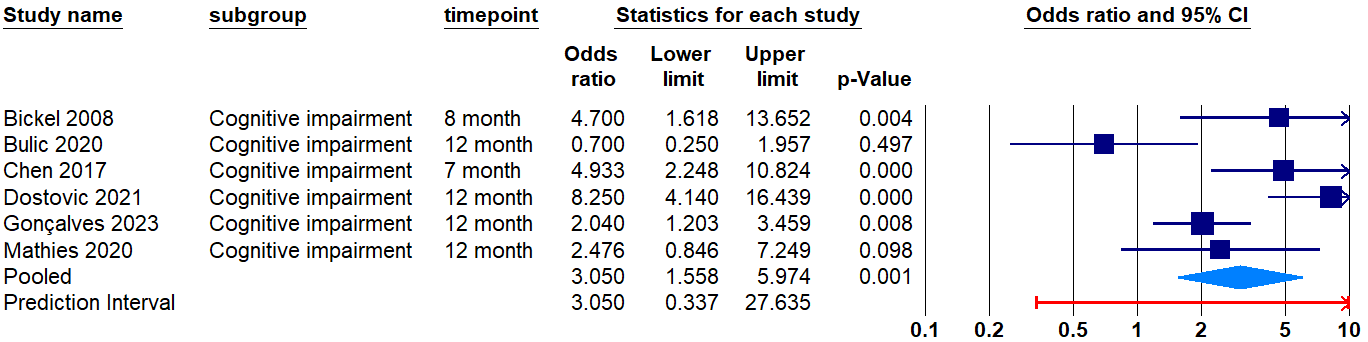** |
| **Objective cognition**  **(performance)** | Cat | >12 months | **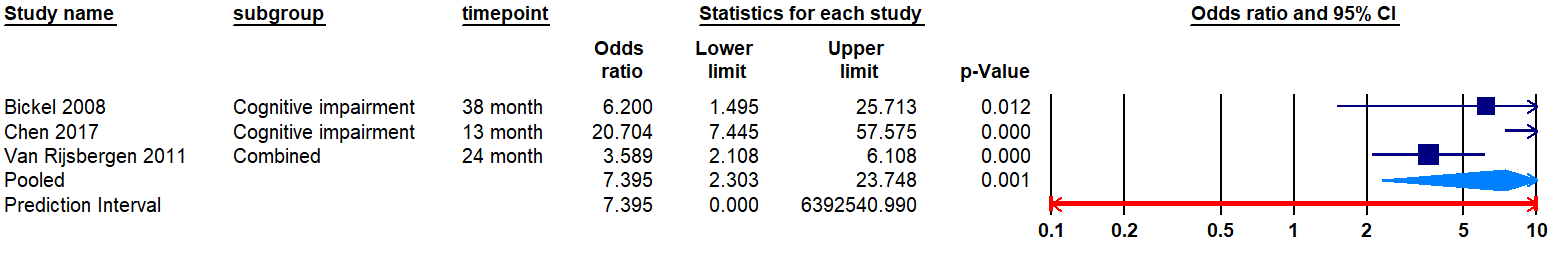** |
| **Objective cognition**  **(performance)** | Con | Collapsed | **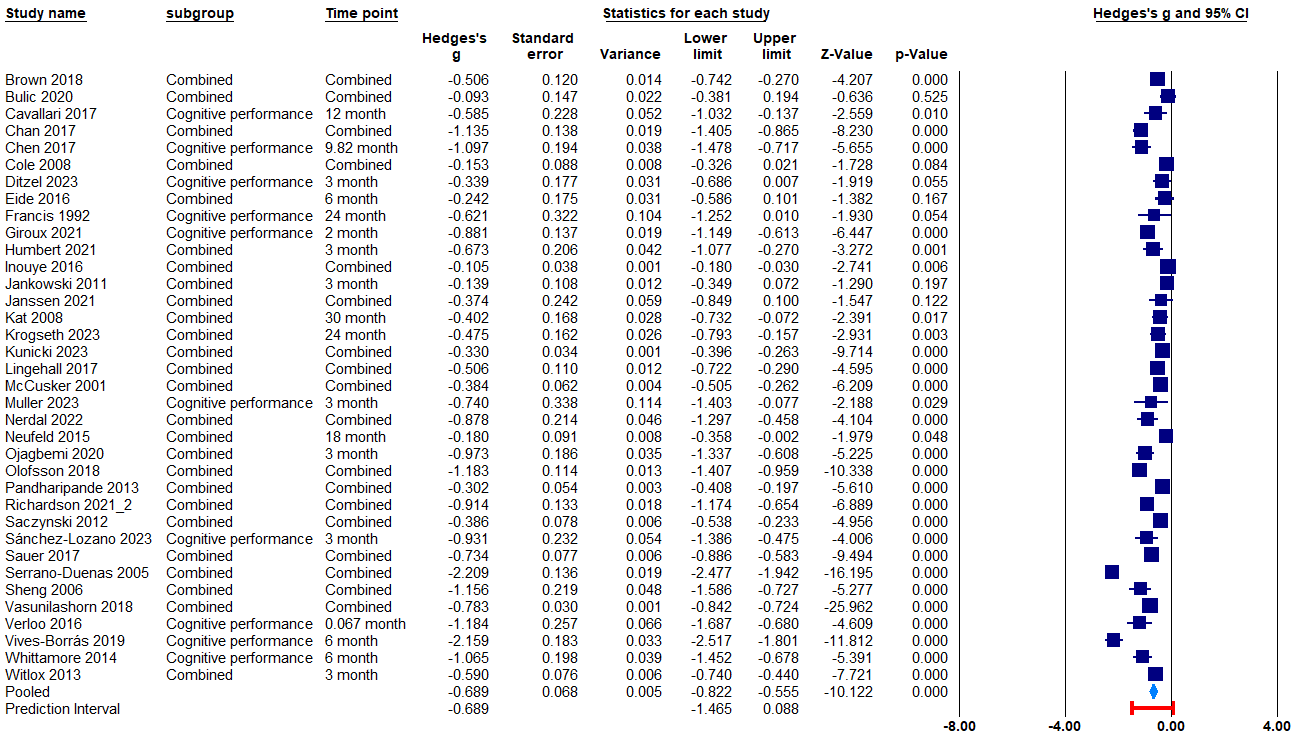** |
| **Objective cognition**  **(performance)** | Con | ≤6 months | **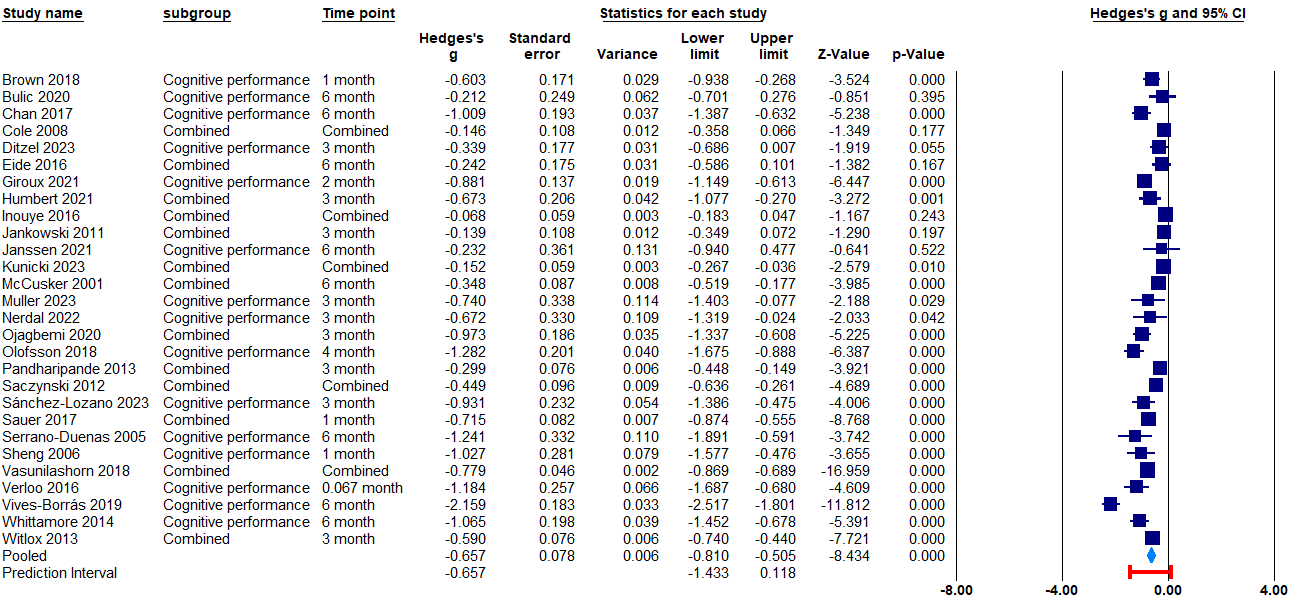** |
| **Objective cognition**  **(performance)** | Con | >6-12 months | **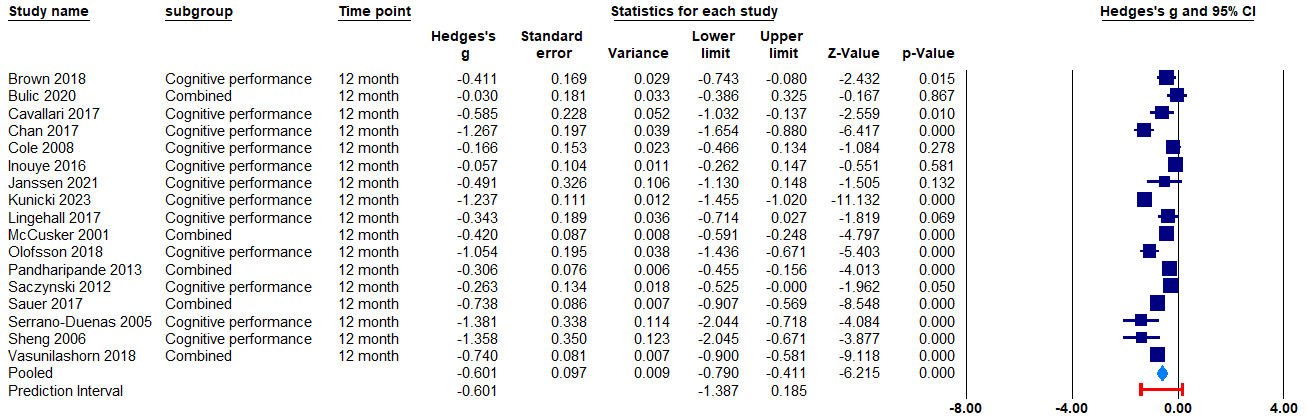** |
| **Objective cognition**  **(performance)** | Con | >12 months | **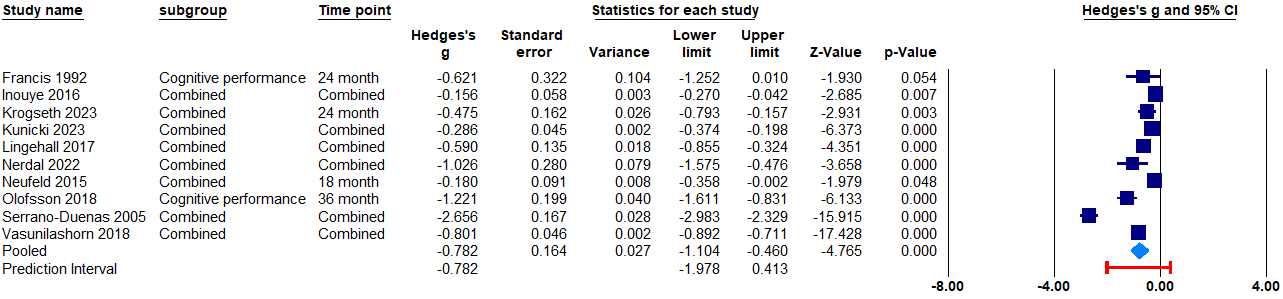** |
| **Subjective cognition**  **(performance)** | Cat | Collapsed | **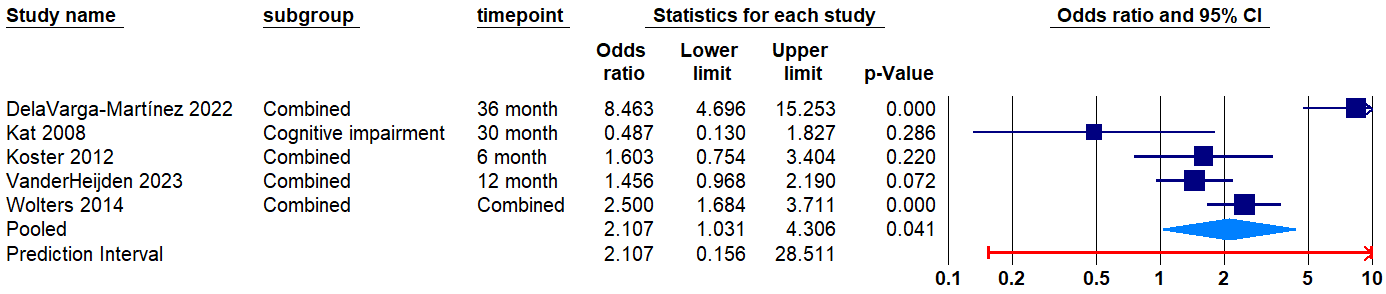** |
| **Subjective cognition**  **(performance)** | Cat | ≤6 months | **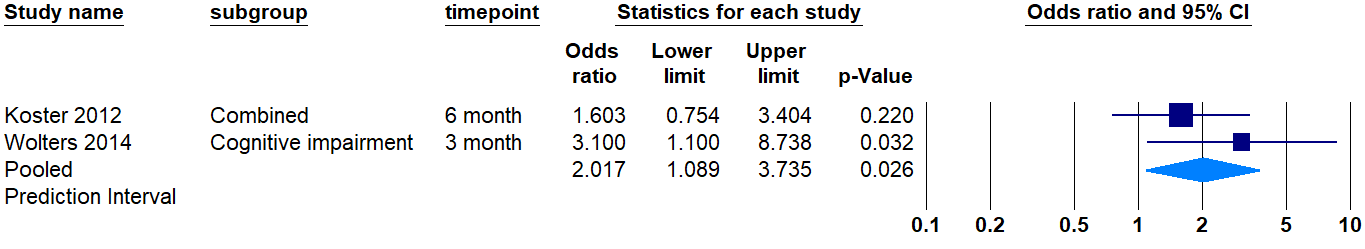** |
| **Subjective cognition**  **(performance)** | Cat | >6-12 months | **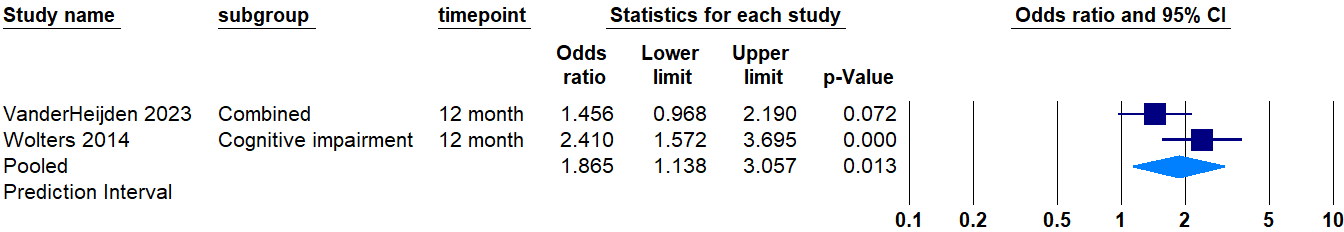** |
| **Subjective cognition**  **(performance)** | Con | Collapsed | **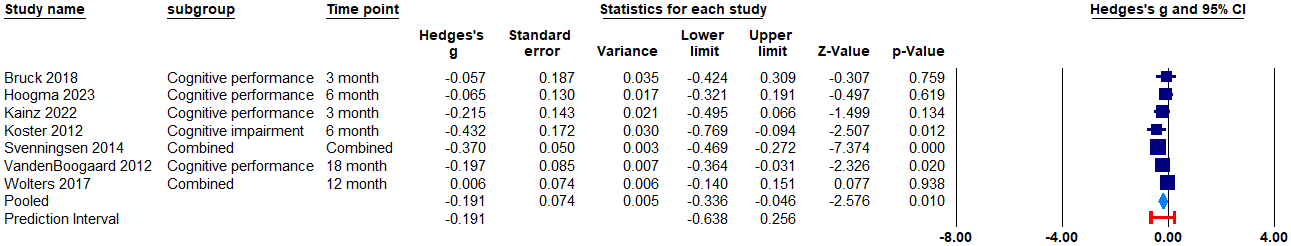** |
| **Subjective cognition**  **(performance)** | Con | ≤6 months | **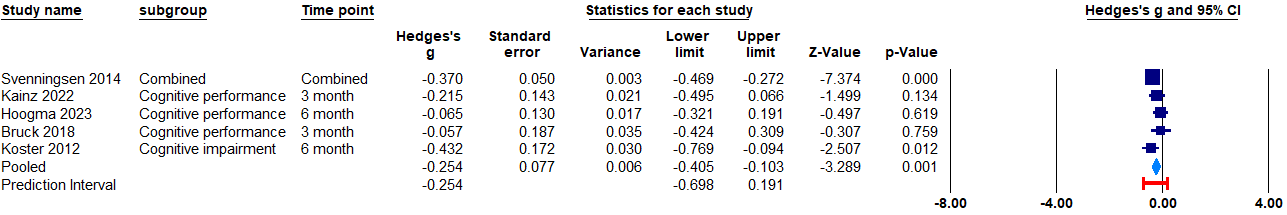** |
| **Functional change** | **Cat** | Collapsed | **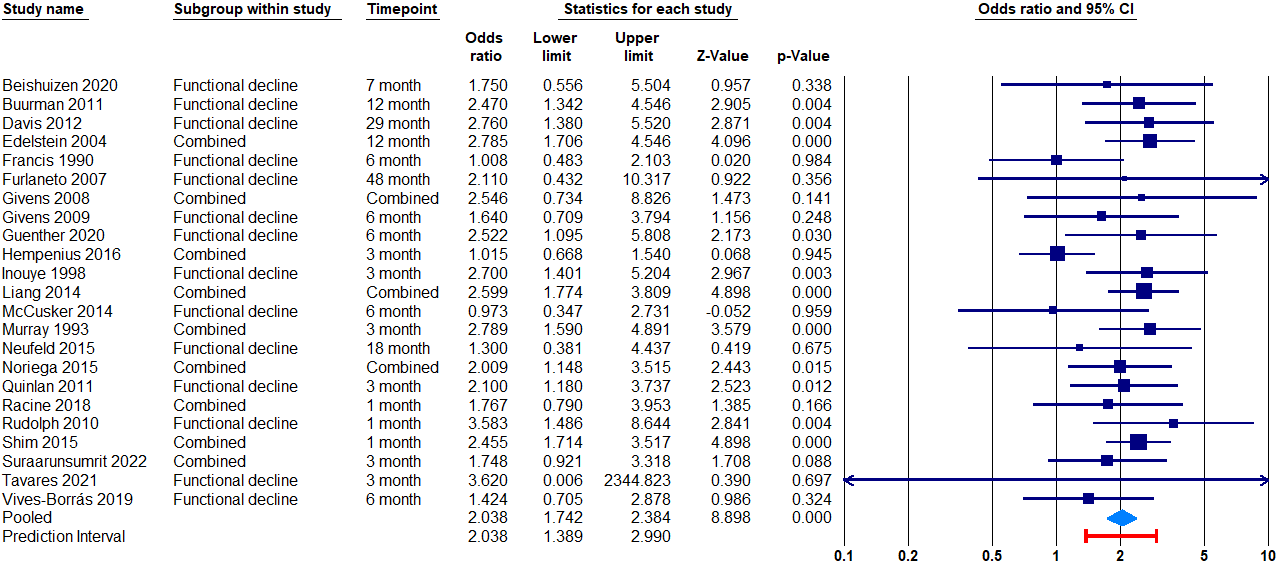** |
|  |  | ≤6 months | **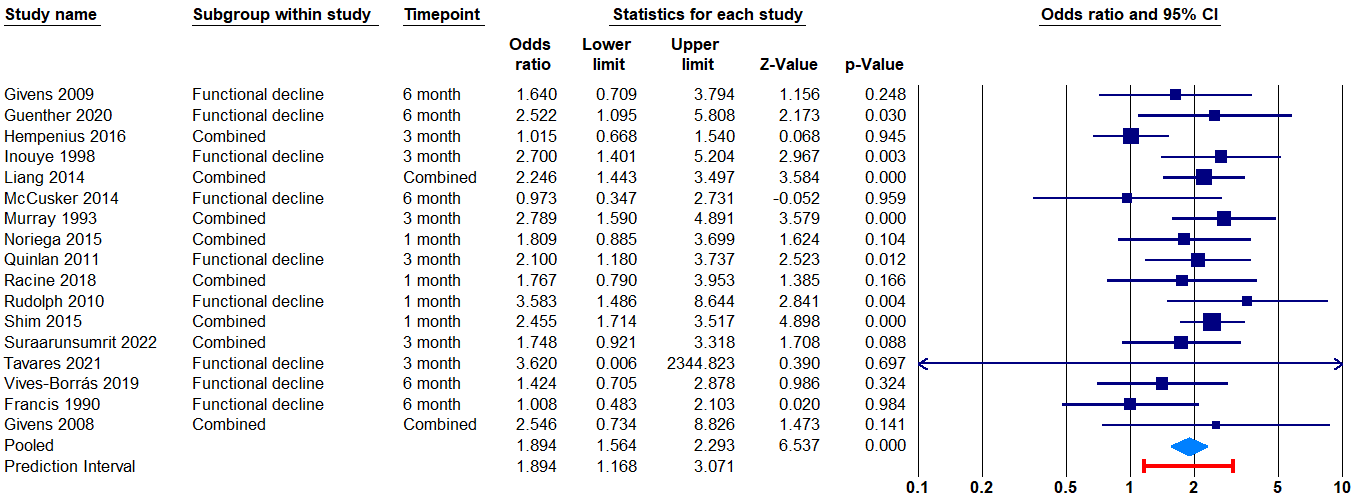** |
|  |  | >6-12 months | **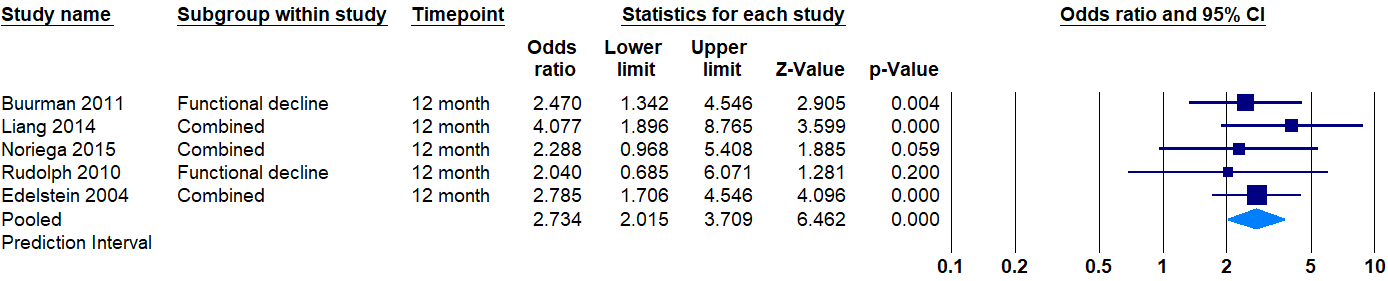** |
|  |  | >12 months | **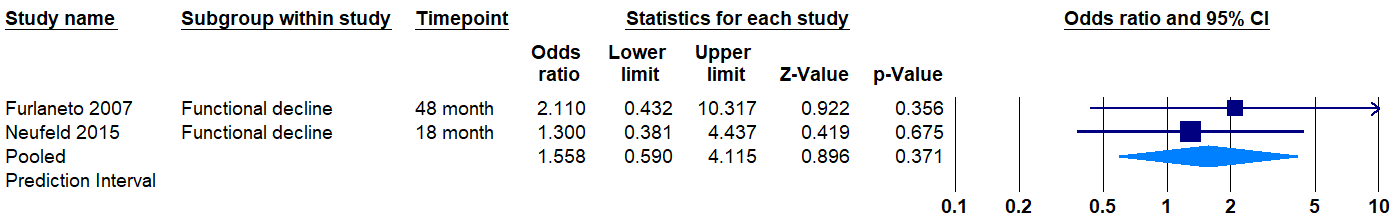** |
| **Functional change** | **Con** | Collapsed | **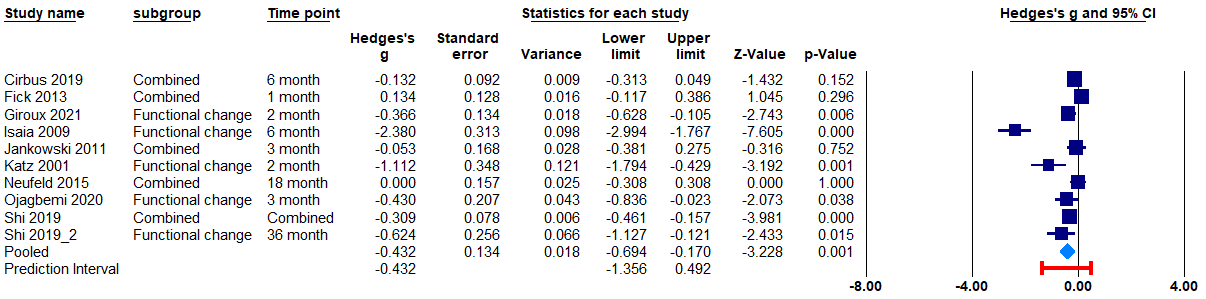** |
|  |  | ≤6 months | **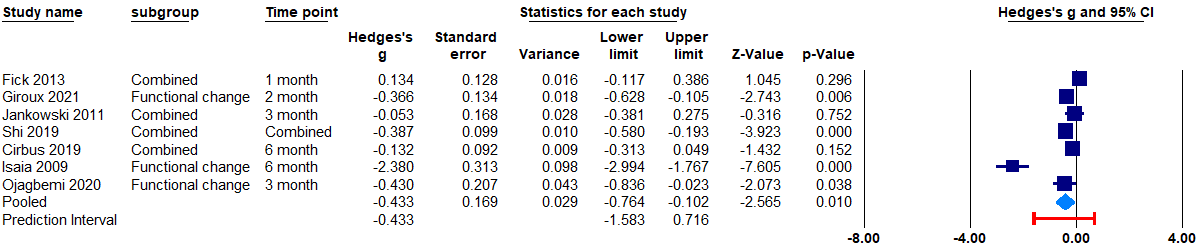** |
|  |  | >12 months | **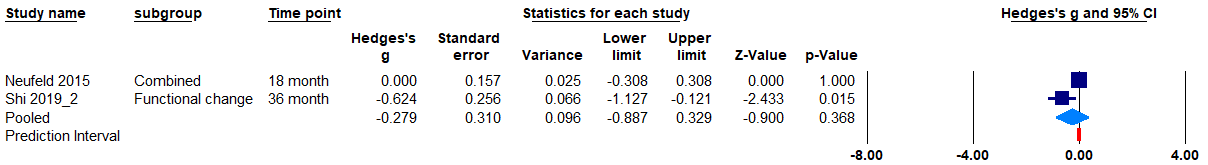** |
| **Functional performance** | **Cat** | Collapsed | **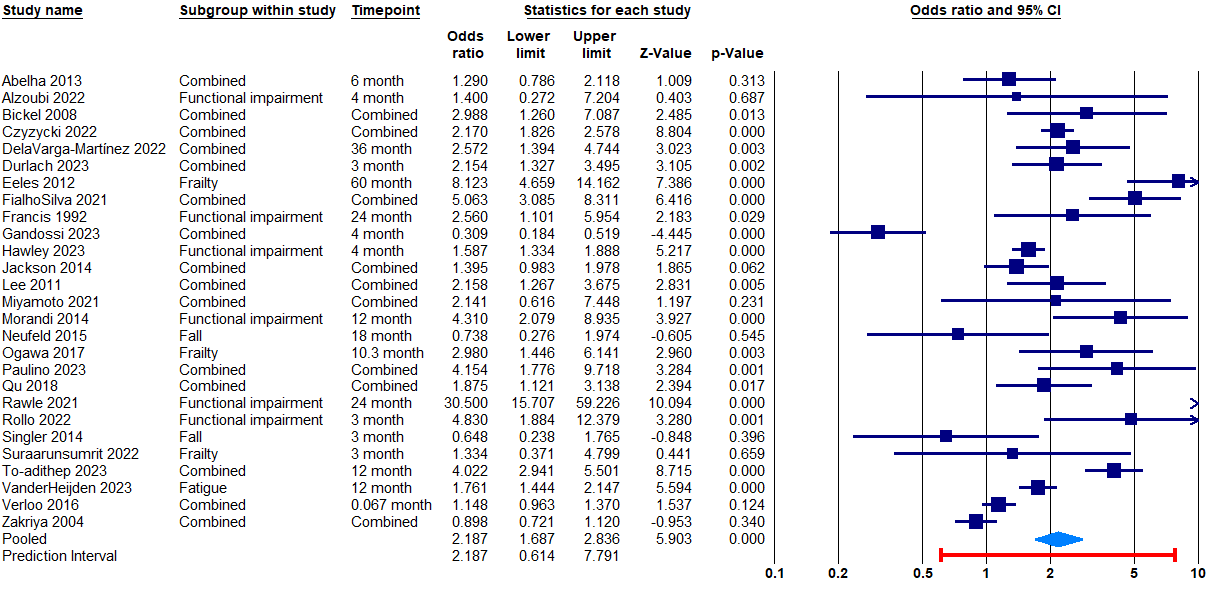** |
|  |  | ≤6 months | **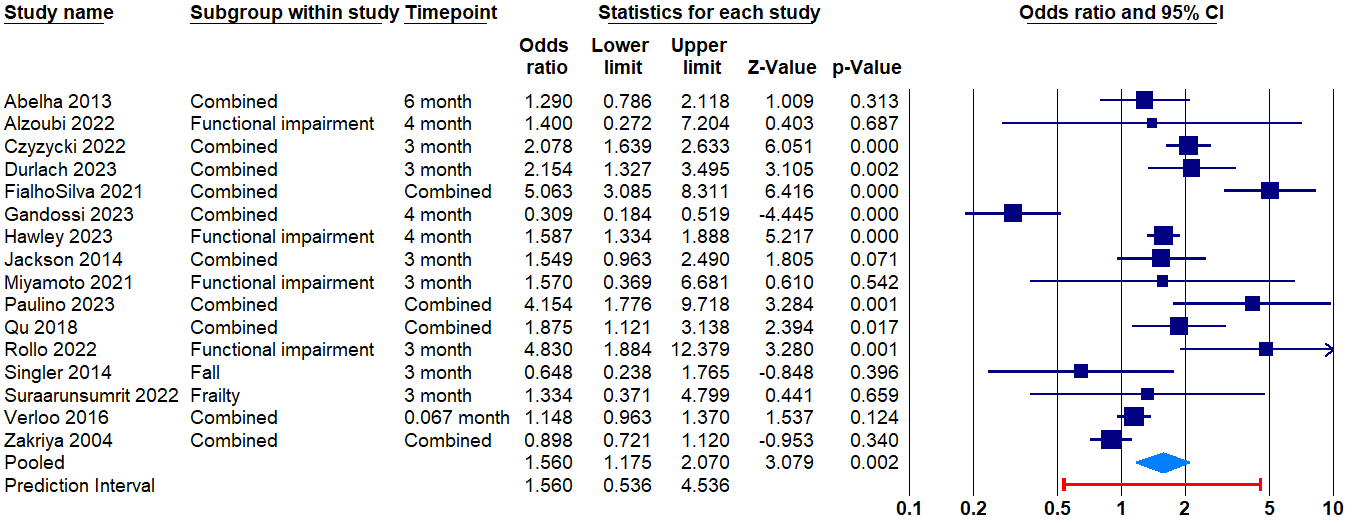** |
|  |  | >6-12 months | **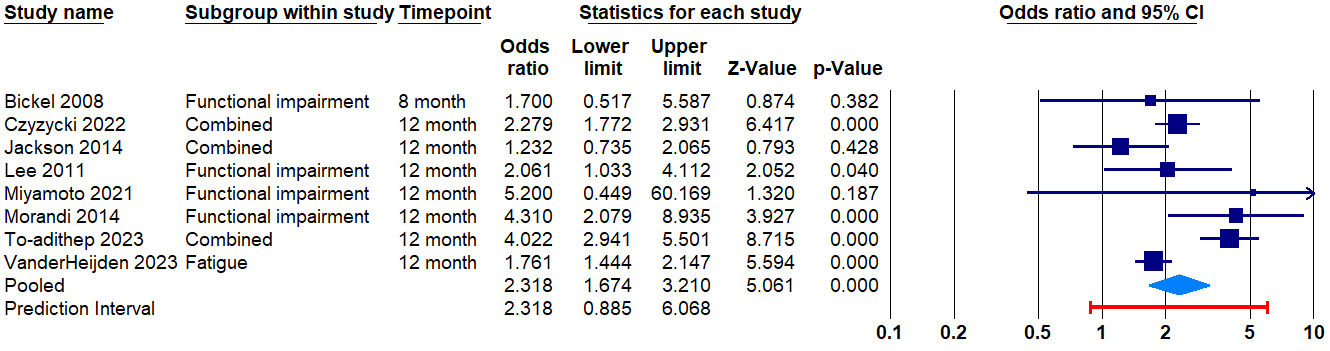** |
|  |  | >12 months | **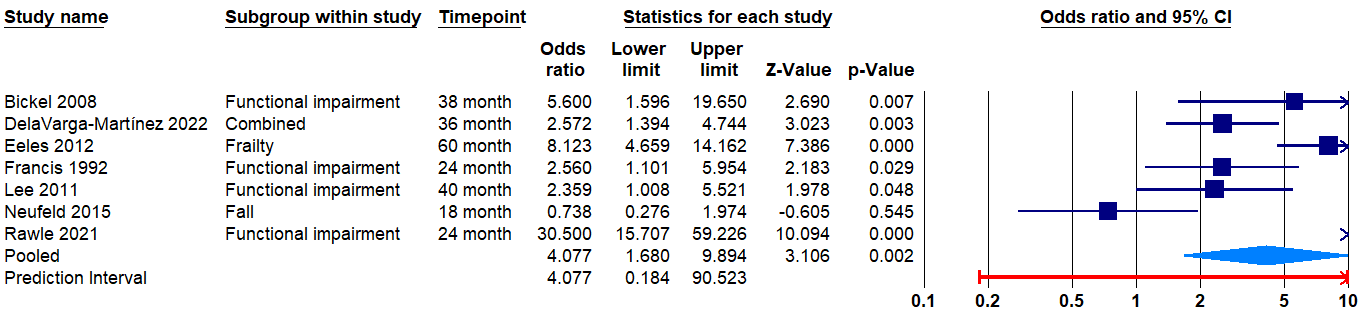** |
| **Functional performance** | **Con** | Collapsed | **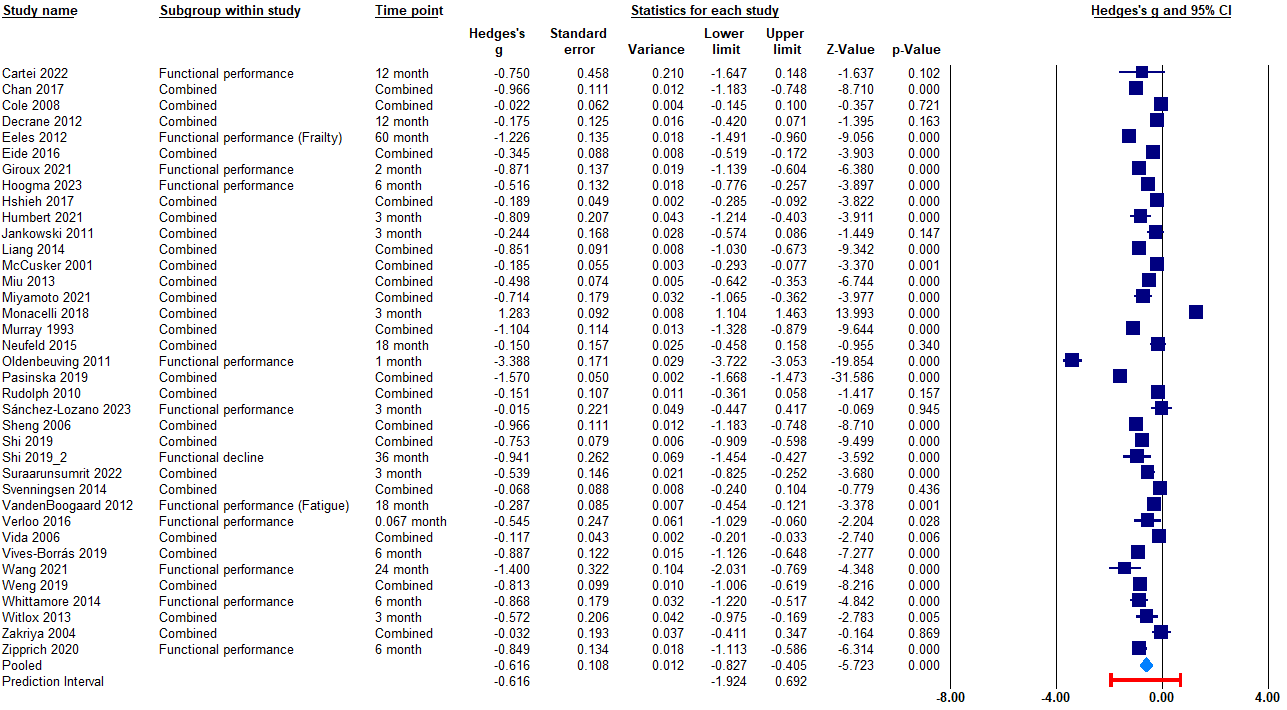** |
|  |  | ≤6 months | 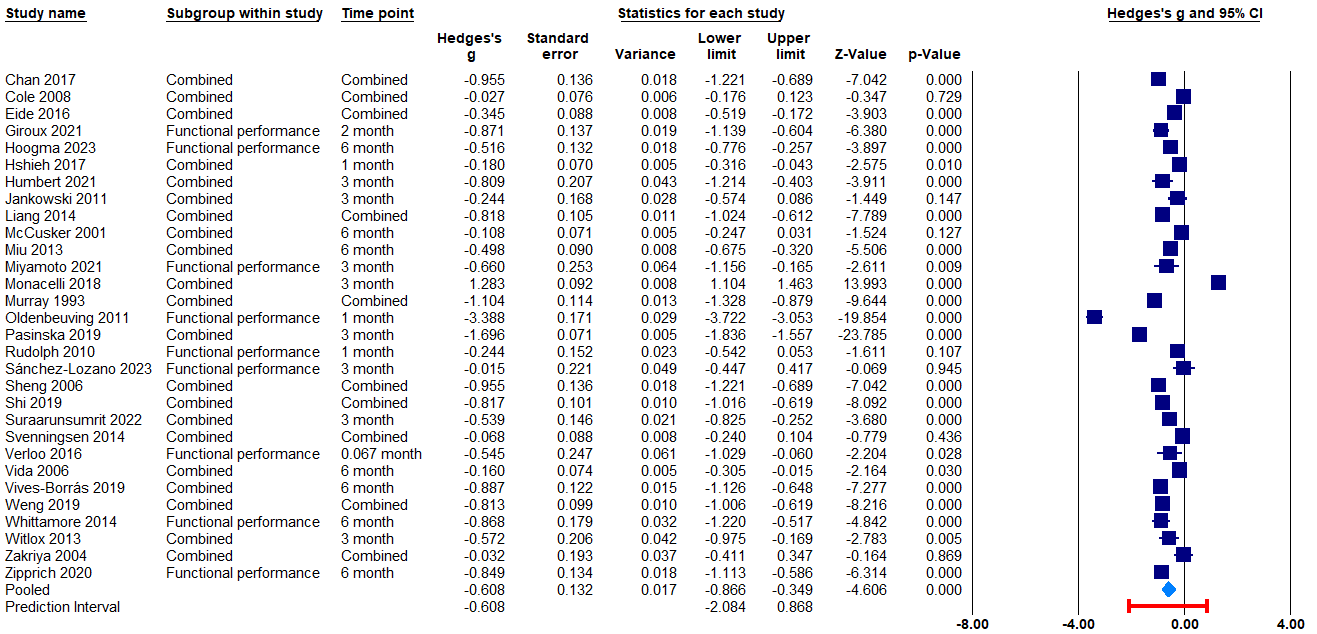 |
|  |  | >6-12 months | 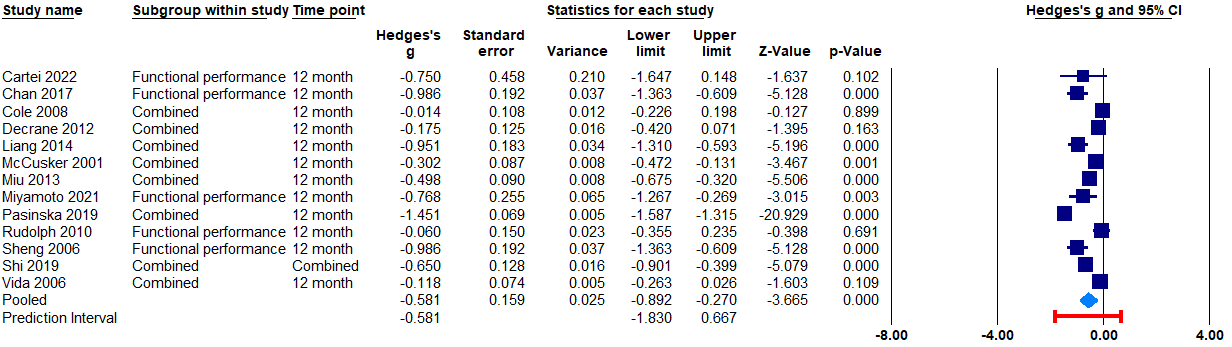 |
|  |  | >12 months | 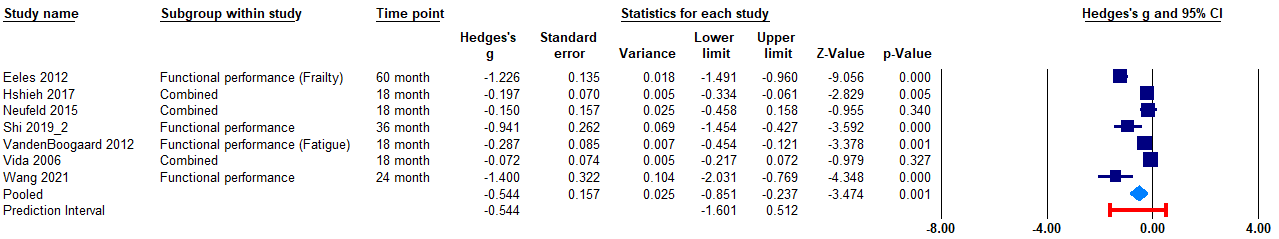 |
| **Quality of life** | **Cat** | Collapsed | **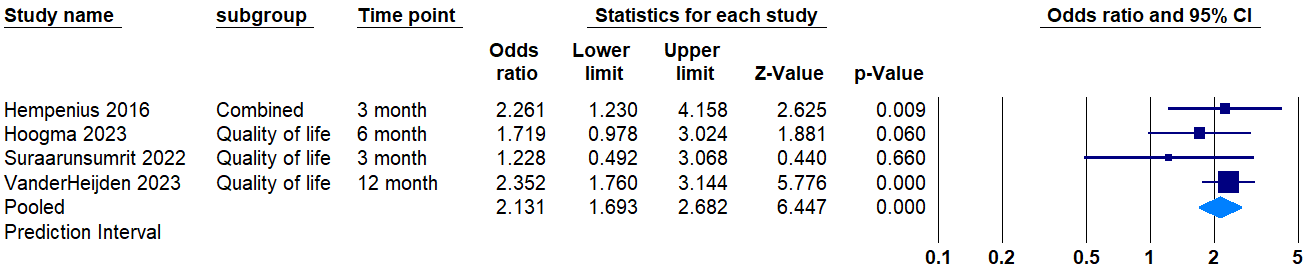** |
|  |  | ≤6 months | **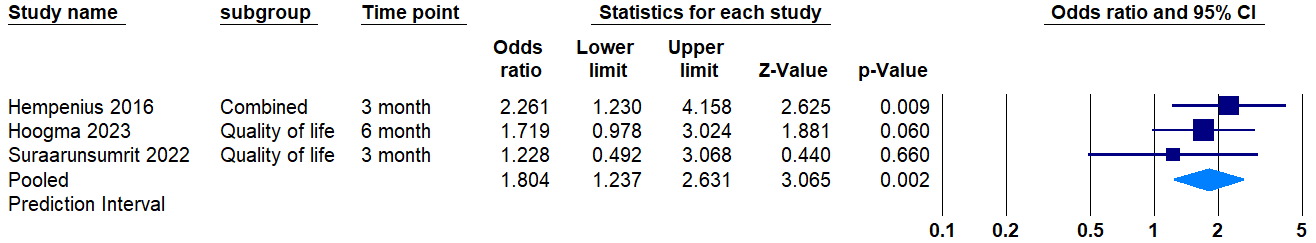** |
| **Quality of life** | **Con** | Collapsed | **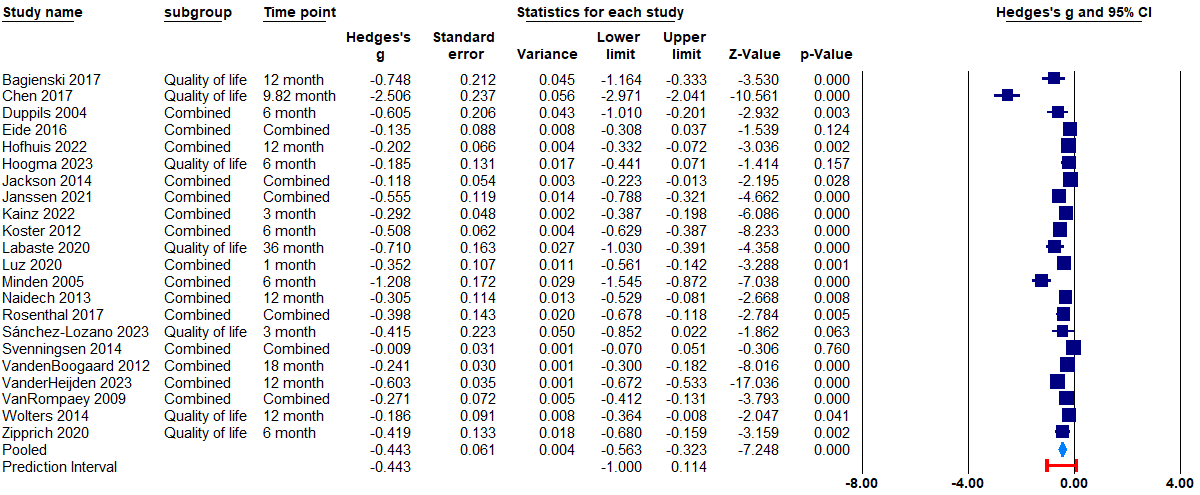** |
|  |  | ≤6 months | **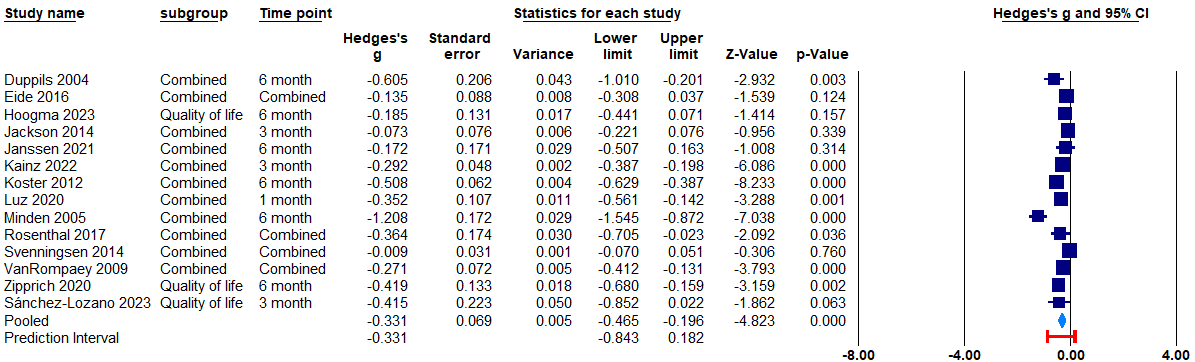** |
|  |  | >6-12 months | **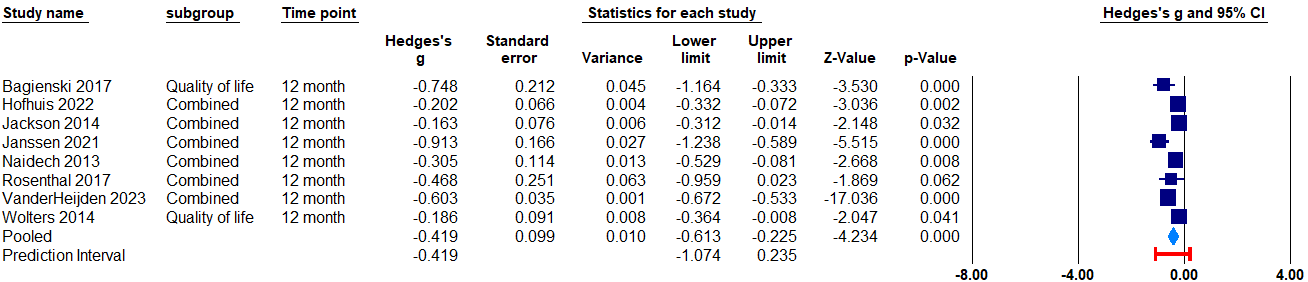** |
|  |  | >12 months | **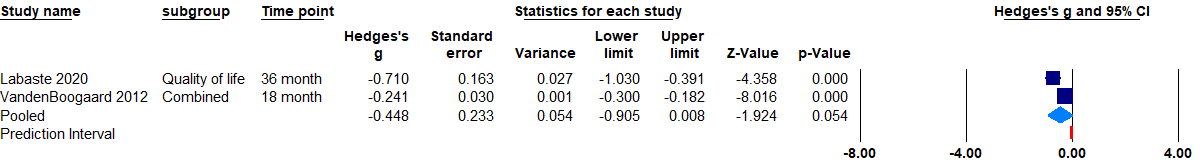** |
| **Mental health** | **Cat** | Collapsed | **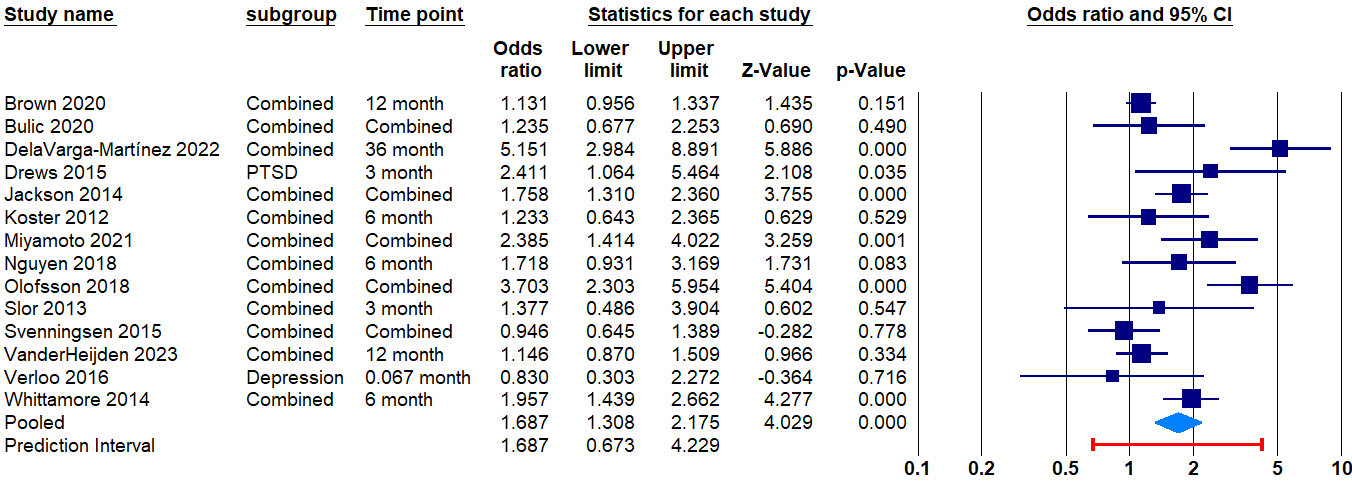** |
|  |  | ≤6 months | **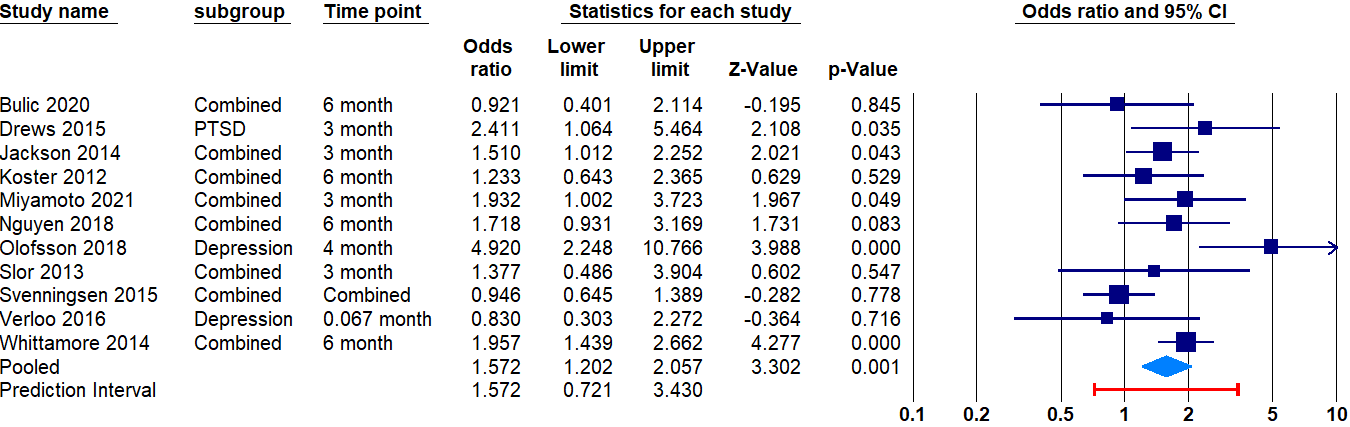** |
|  |  | >6-12 months | **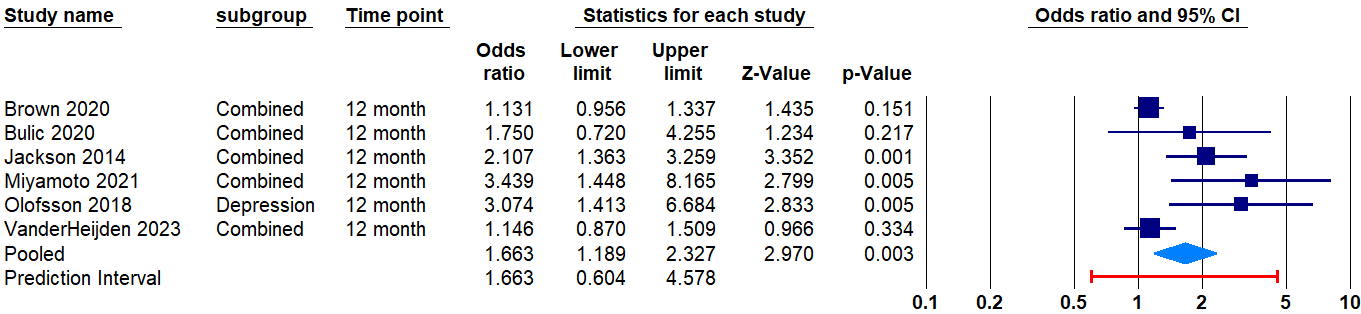** |
|  |  | >12 months | **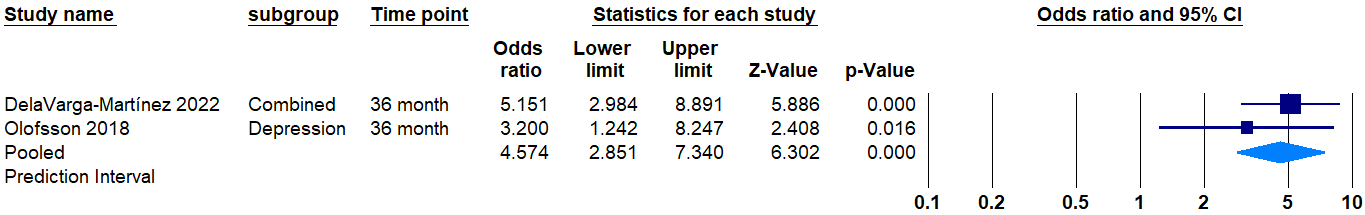** |
| **Mental health** | **Con** | Collapsed | **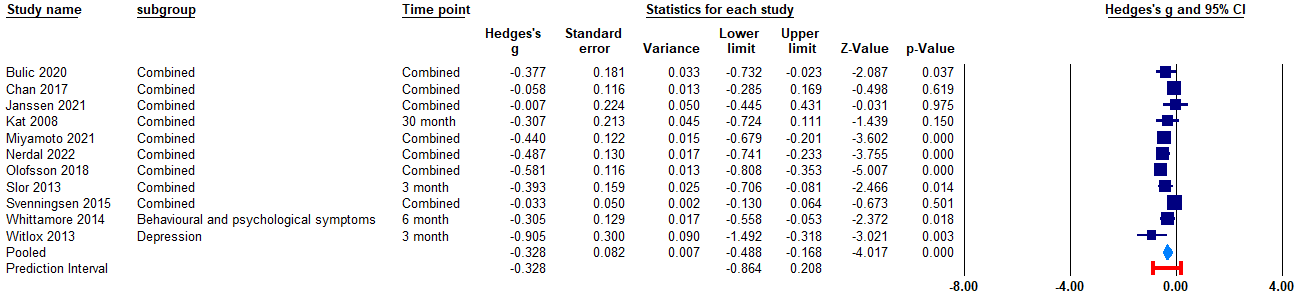** |
|  |  | ≤6 months | **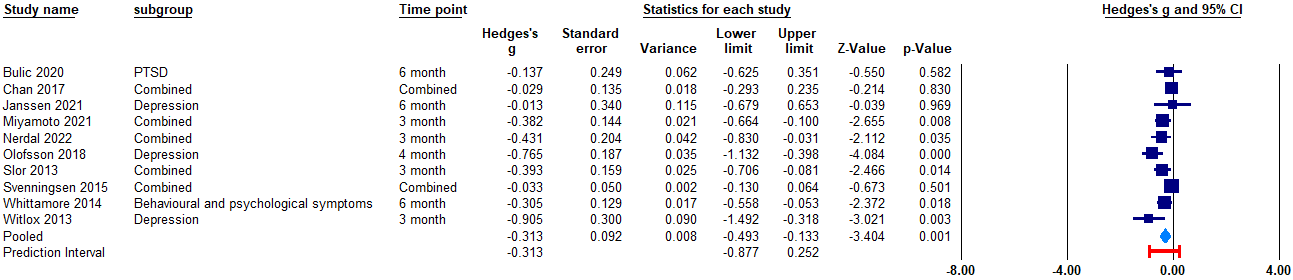** |
|  |  | >6-12 months | **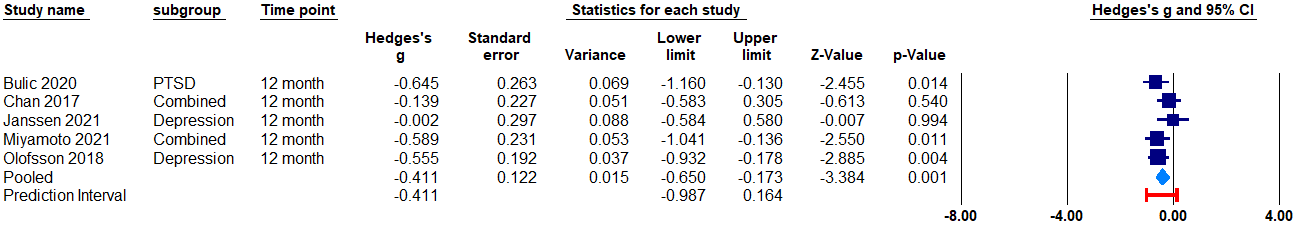** |
|  |  | >12 months | **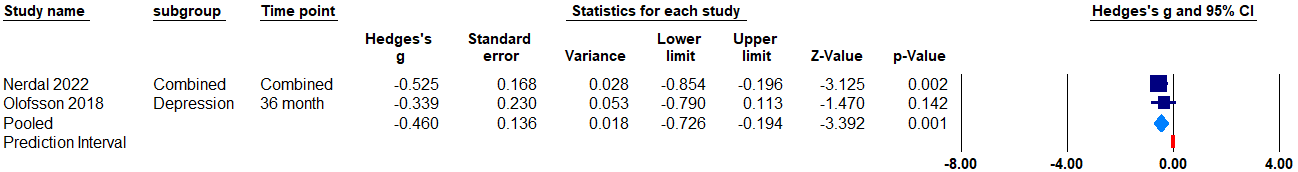** |
| **Dementia** | **Cat** | Collapsed | **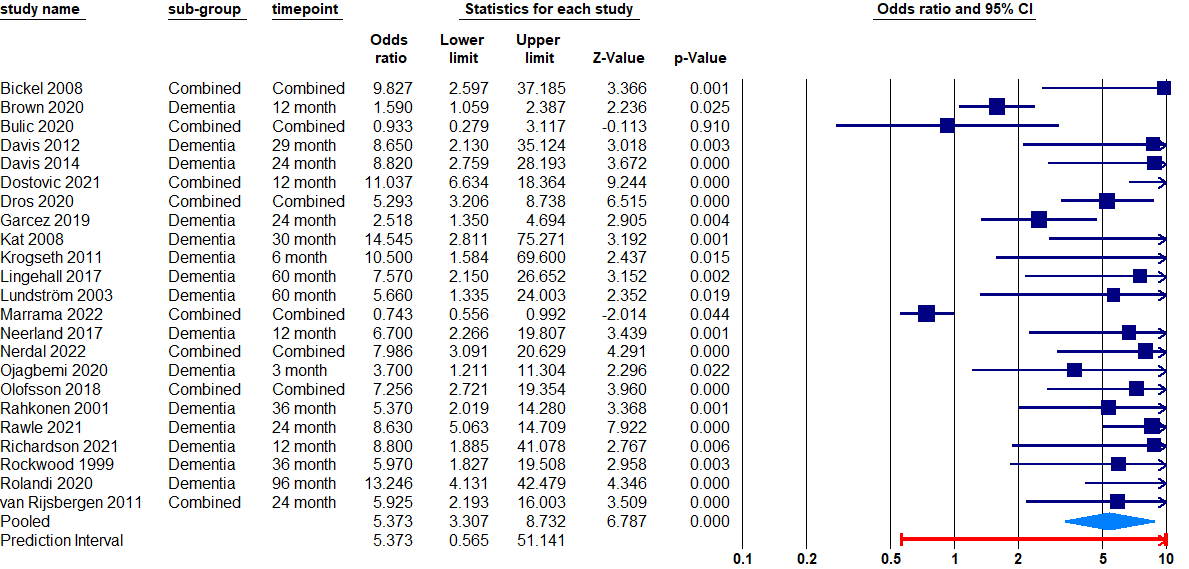** |
|  |  | ≤6 months | **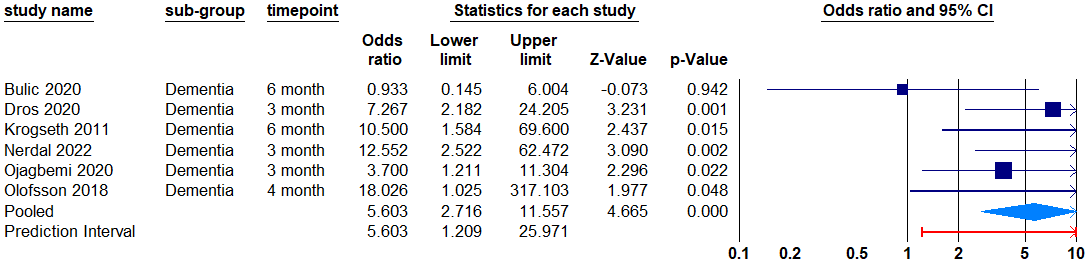** |
|  |  | >6-12 months | **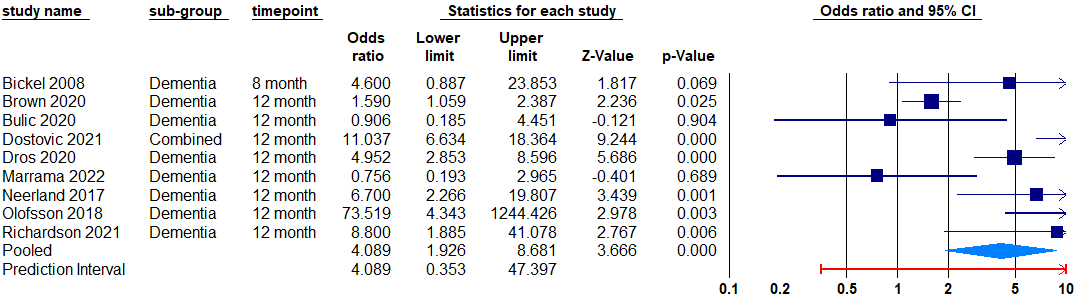** |
|  |  | >12 months | **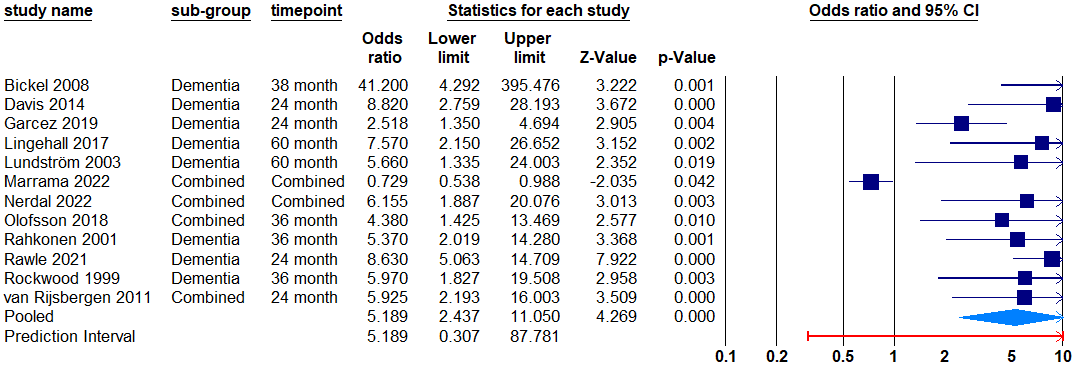** |
| **Institutionalisation** | **Cat** | Collapsed | **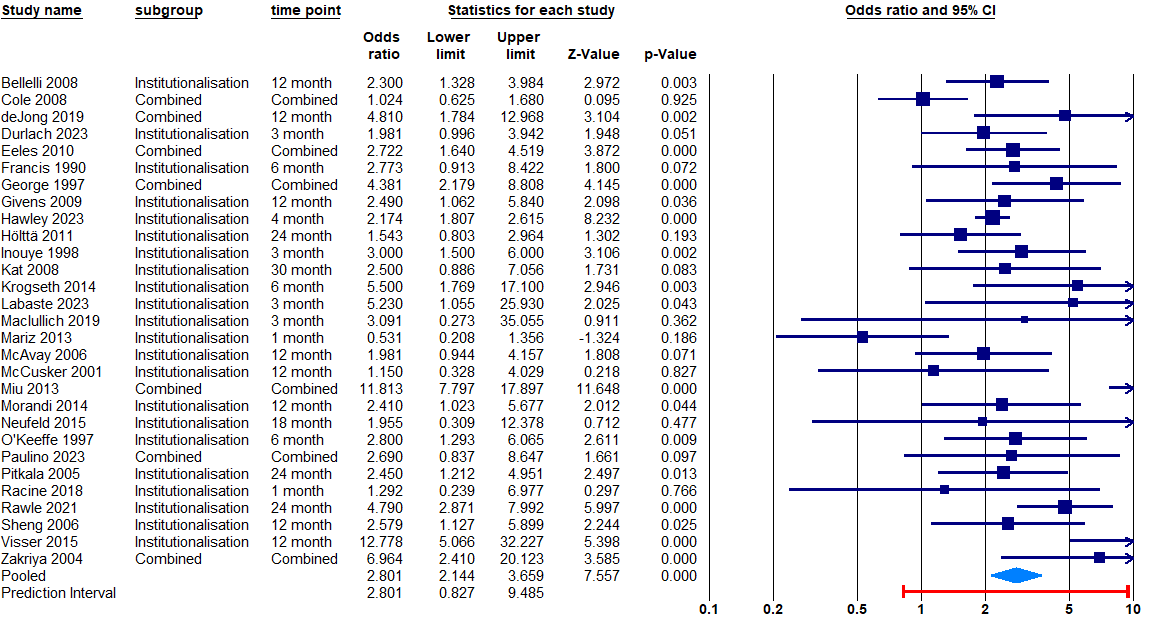** |
|  |  | ≤6 months | **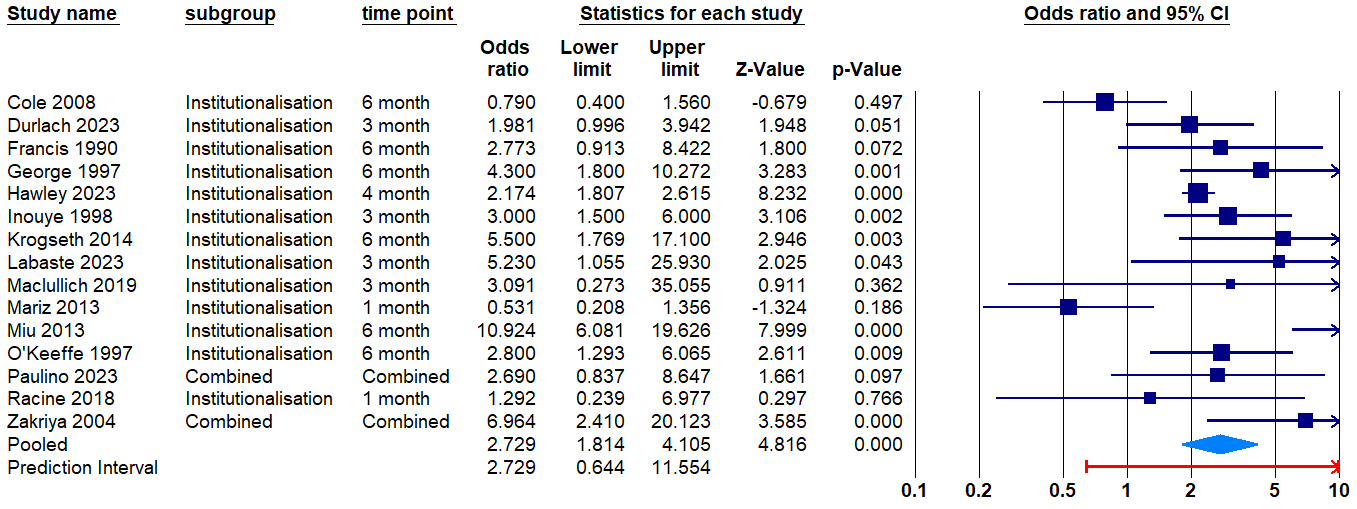** |
|  |  | >6-12 months | **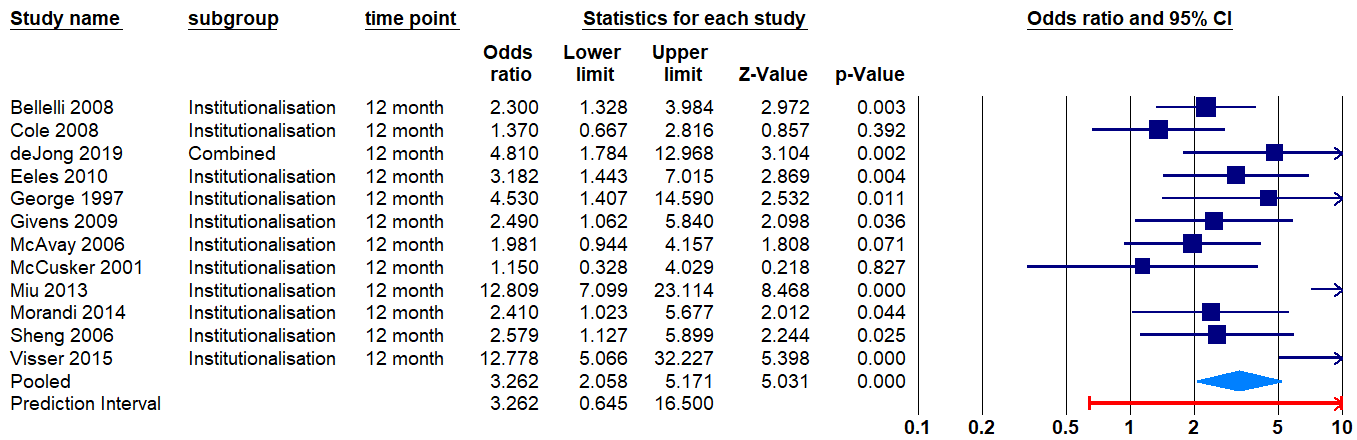** |
|  |  | >12 months | **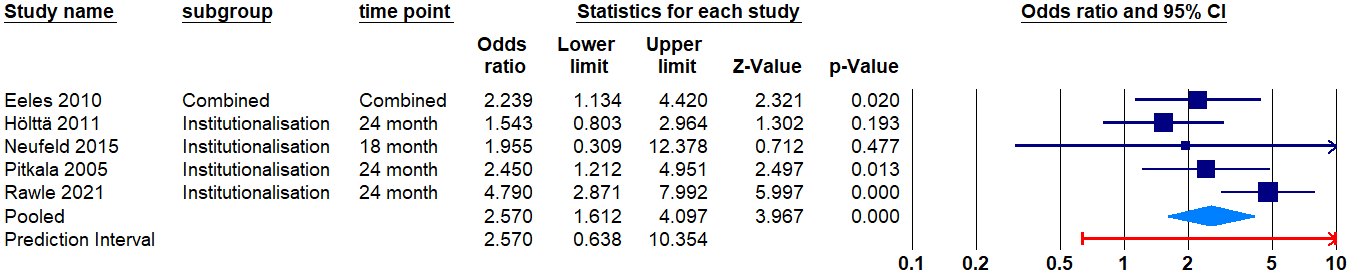** |
| **Readmission/revisit** | **Cat** | Collapsed | **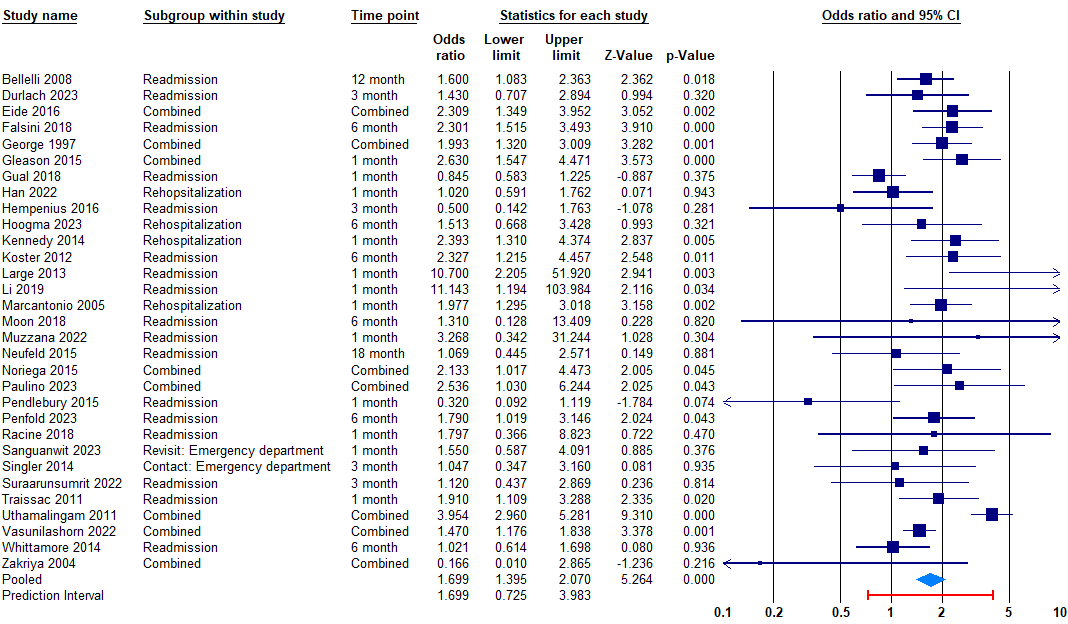** |
|  |  | ≤6 months | **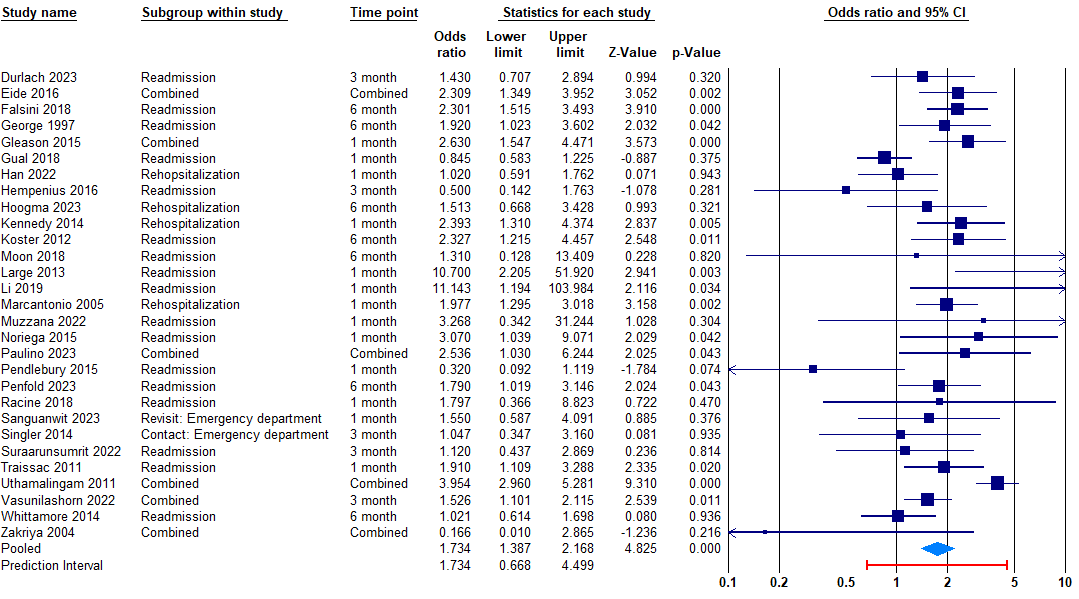** |
|  |  | >6–12 months | **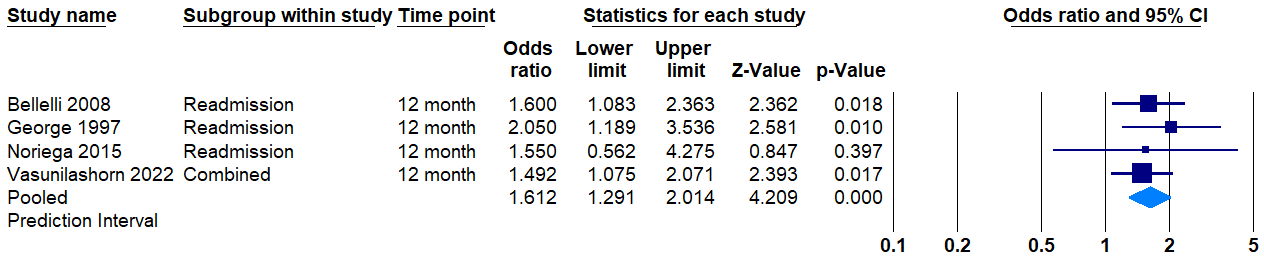** |
| **Mortality** | **Cat** | Collapsed | 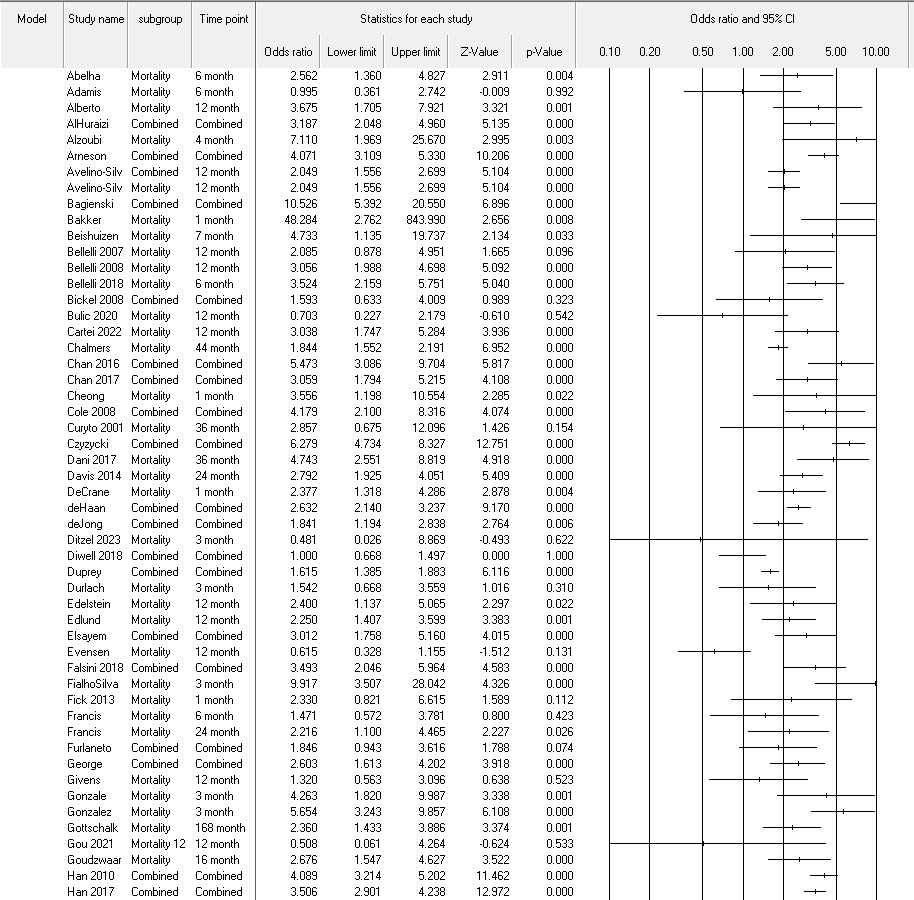 |
|  |  |  | 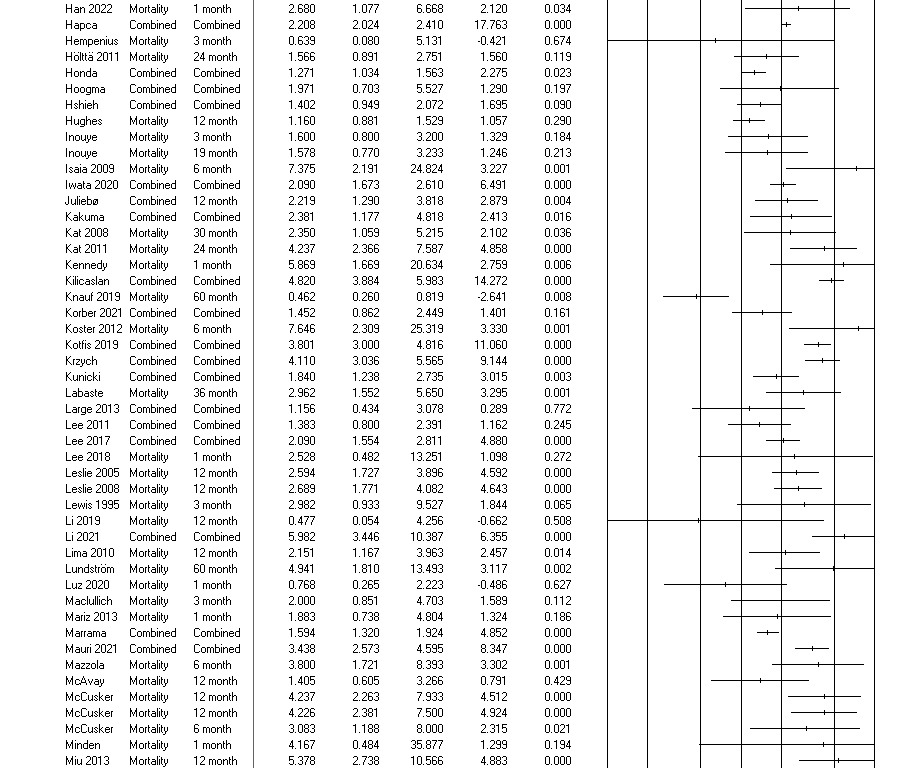 |
|  |  |  | 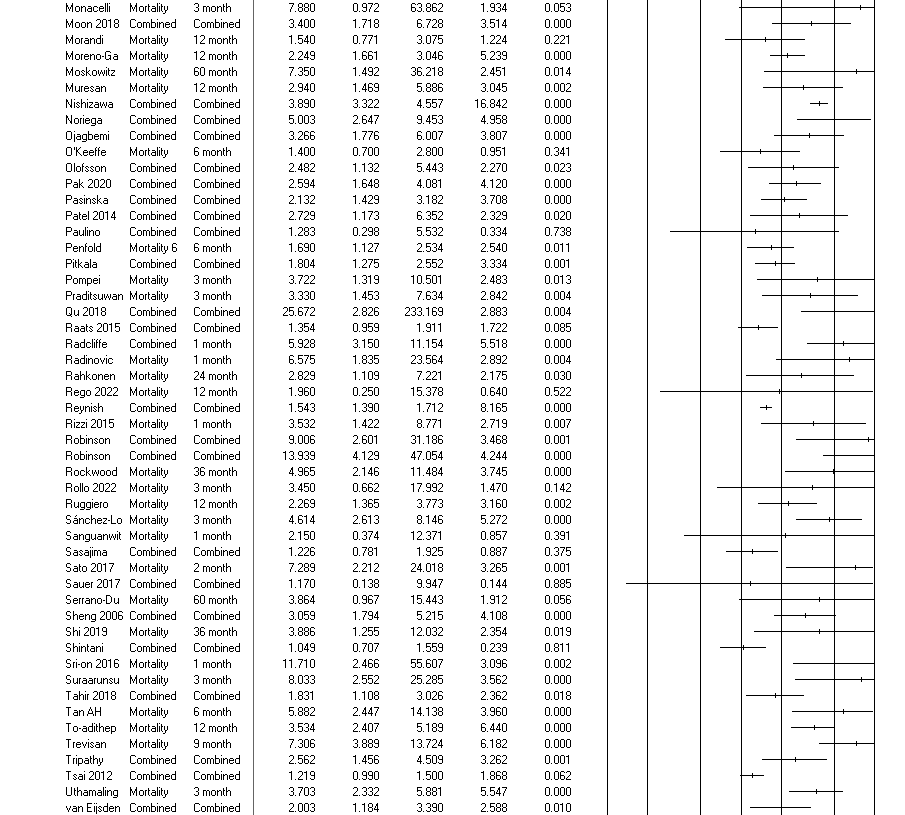 |
|  |  |  | 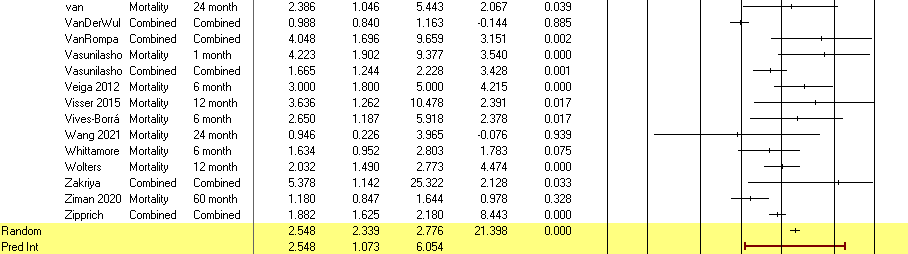 |
|  |  | ≤6 months | 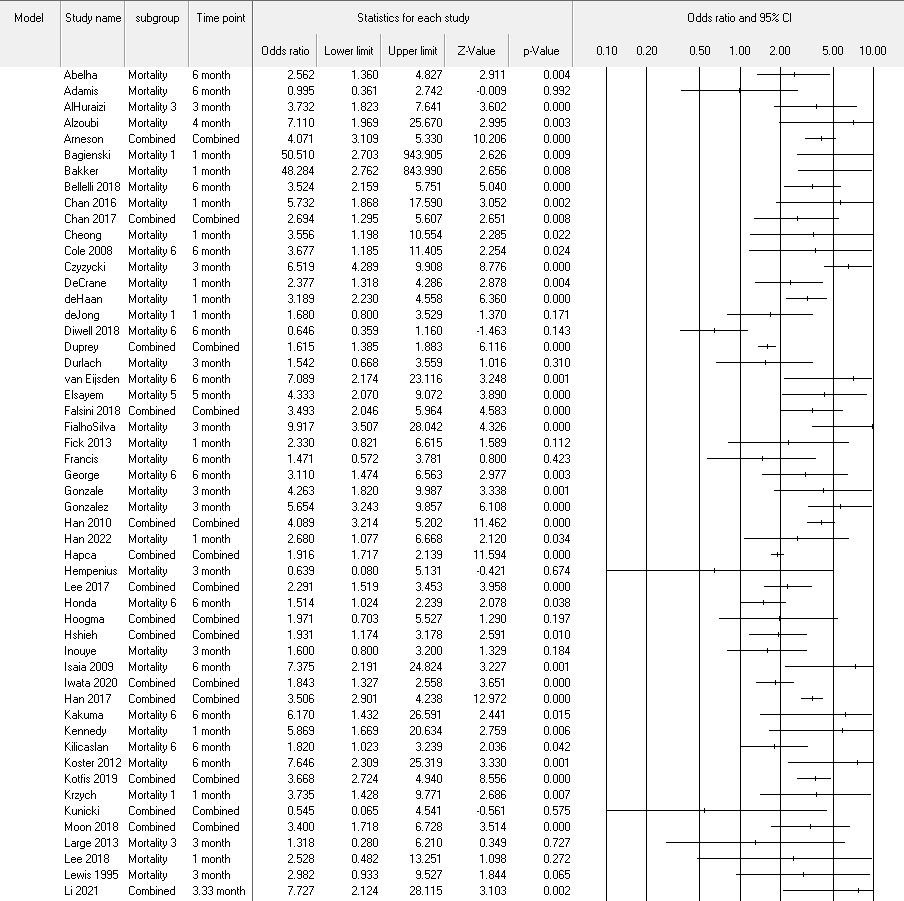 |
|  |  |  | 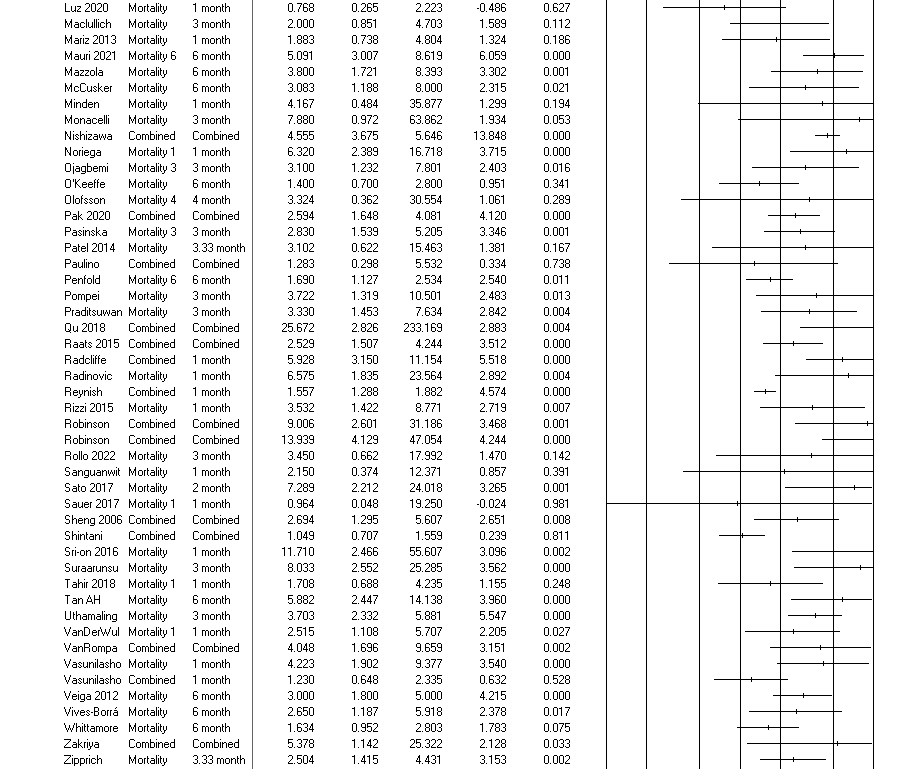 |
|  |  |  | 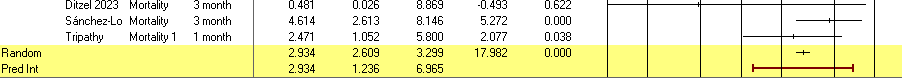 |
|  |  | >6-12 months | 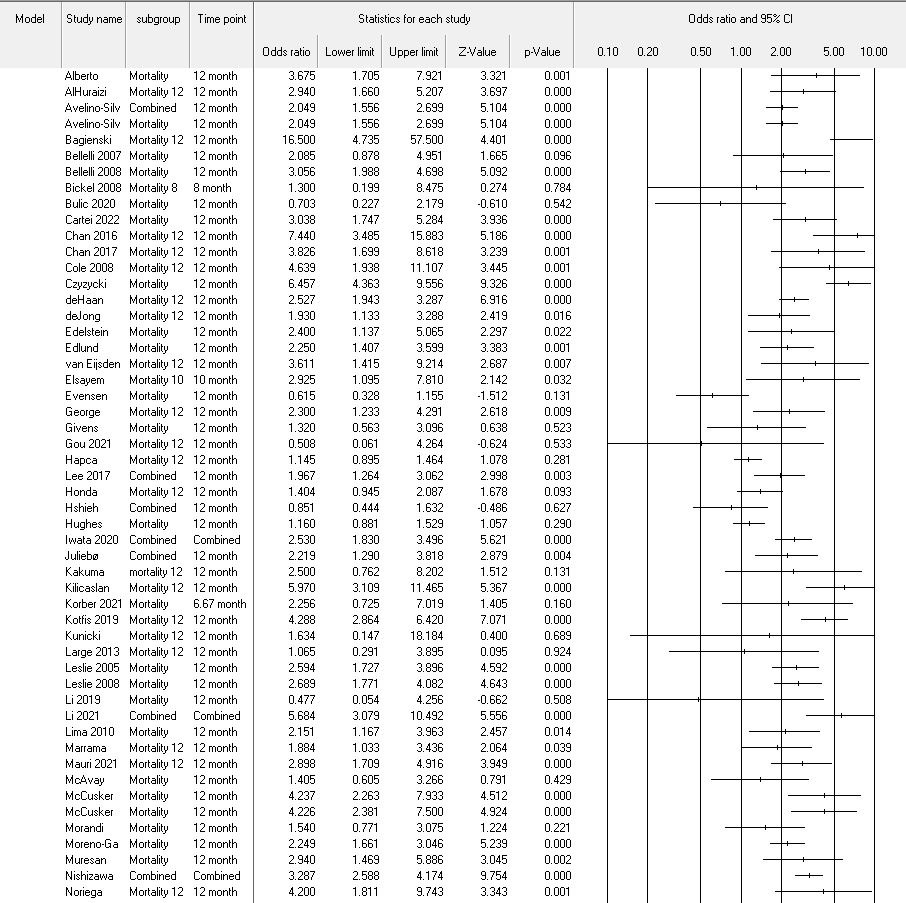 |
|  |  |  | 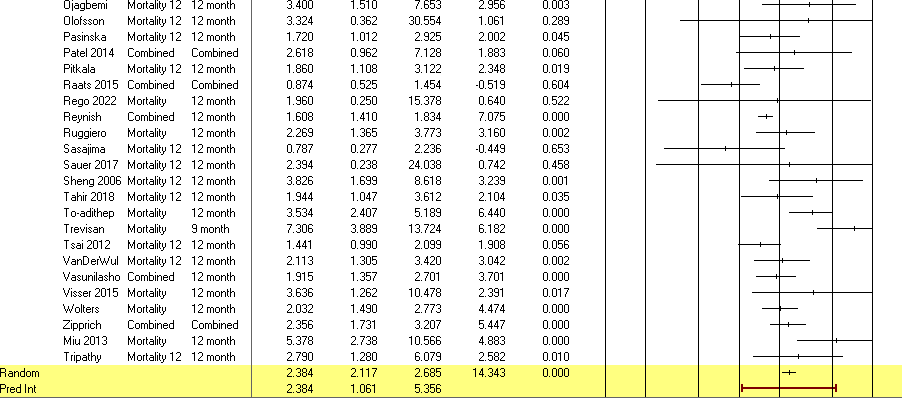 |
|  |  | >12 months | **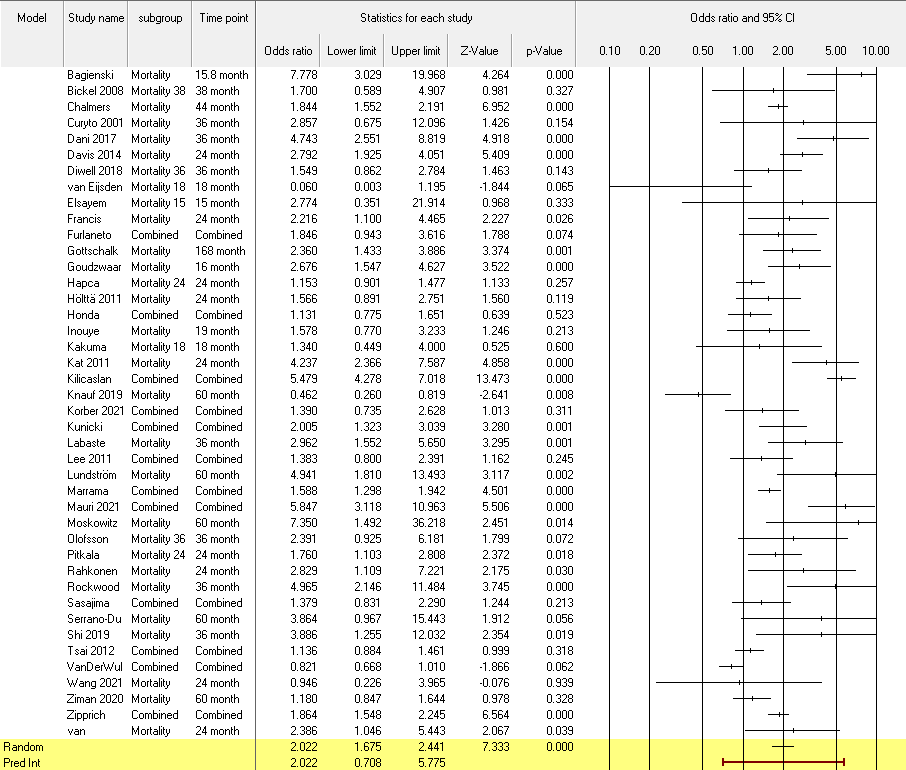** |

**Sensitivity analyses**

# Table S10. Covariates adjusted and unadjusted data analyses. Categorical data results

| **Clinical outcome** | **Sub-group**  **(number of studies)** | **Timepoint** | **Model adjustment** | **OR** | **95% CI** | **p-value** | **I²** (%) | **τ²** |
| --- | --- | --- | --- | --- | --- | --- | --- | --- |
| Cognition | *Objective cognition measure* | | | | | | | |
|  | Cognitive performance (k=4) | Collapsed | Adjusted | 2.382 | 1.154–4.917 | 0.019 | 67.9 | 0.353 |
|  | Cognitive performance (k=3) | ≤6 months | Adjusted | 2.058 | 0.927–4.569 | 0.076 | 75.7 | 0.374 |
|  | Cognitive performance (k=3) | >6–12 months | Adjusted | 4.079 | 2.365–7.035 | <0.001 | 0.0 | 0.000 |
|  | Cognitive performance (k=2) | >12 months | Adjusted | 12.590 | 3.934–40.299 | <0.001 | 45.0 | 0.327 |
|  | Cognitive performance (k=6) | Collapsed | Unadjusted | 2.518 | 1.350–4.697 | 0.004 | 77.0 | 0.440 |
|  | Cognitive performance (k=2) | ≤6 months | Unadjusted | 1.277 | 0.578–2.820 | 0.545 | 0.0 | 0.000 |
|  | Cognitive performance (k=4) | >6–12 months | Unadjusted | 2.422 | 0.928–6.319 | 0.071 | 83.1 | 0.774 |
| Functional outcome | Functional change (k=6) | Collapsed | Adjusted | 2.312 | 1.706–3.133 | <0.001 | 0.0 | 0.000 |
|  | Functional change (k=4) | ≤6 months | Adjusted | 2.204 | 1.526–3.183 | <0.001 | 0.0 | 0.000 |
|  | Functional change (k=19) | Collapsed | Unadjusted | 1.942 | 1.608–2.346 | <0.001 | 30.6 | 0.048 |
|  | Functional change (k=14) | ≤6 months | Unadjusted | 1.811 | 1.448–2.266 | <0.001 | 36.9 | 0.061 |
|  | Functional change (k=4) | >6–12 months | Unadjusted | 2.805 | 2.024–3.886 | <0.001 | 0.0 | 0.000 |
|  | Functional change (k=2) | >12 months | Unadjusted | 1.558 | 0.590–4.115 | 0.371 | 0.0 | 0.000 |
|  | Functional performance (k=13) | Collapsed | Adjusted | 2.017 | 1.675–2.456 | <0.001 | 49.7 | 0.046 |
|  | Functional performance (k=8) | ≤6 months | Adjusted | 1.757 | 1.470–2.099 | <0.001 | 26.6 | 0.015 |
|  | Functional performance (k=5) | >6–12 months | Adjusted | 2.176 | 1.387–3.413 | 0.001 | 54.6 | 0.122 |
|  | Functional performance (k=3) | >12 months | Adjusted | 2.893 | 1.692–4.945 | <0.001 | 0.0 | 0.000 |
|  | Functional performance (k=15) | Collapsed | Unadjusted | 2.180 | 1.396–3.404 | 0.001 | 94.5 | 0.666 |
|  | Functional performance (k=8) | ≤6 months | Unadjusted | 1.362 | 0.823–2.254 | 0.230 | 91.3 | 0.420 |
|  | Functional performance (k=3) | >6–12 months | Unadjusted | 2.466 | 1.330–4.573 | 0.004 | 89.5 | 0.252 |
|  | Functional performance (k=3) | >12 months | Unadjusted | 4.270 | 1.293–14.099 | 0.017 | 91.8 | 1.690 |
| Quality of life | Poor quality of life (k=4) | Collapsed | Unadjusted | 2.131 | 1.693–2.682 | <0.001 | 0.0 | 0.000 |
|  | Poor quality of life (k=3) | ≤6 months | Unadjusted | 1.804 | 1.237–2.631 | 0.002 | 0.0 | 0.000 |
| Mental health | Poor mental health (k=8) | Collapsed | Adjusted | 1.775 | 1.283–2.458 | 0.001 | 81.9 | 0.159 |
|  | Poor mental health (k=5) | ≤6 months | Adjusted | 1.628 | 1.259–2.106 | <0.001 | 0.0 | 0.000 |
|  | Poor mental health (k=4) | >6–12 months | Adjusted | 1.495 | 1.052–2.124 | 0.025 | 76.0 | 0.086 |
|  | Poor mental health (k=6) | Collapsed | Unadjusted | 1.539 | 0.962–2.462 | 0.072 | 78.4 | 0.244 |
|  | Poor mental health (k=6) | ≤6 months | Unadjusted | 1.483 | 0.899–2.447 | 0.123 | 74.9 | 0.259 |
|  | Poor mental health (k=2) | >6–12 months | Unadjusted | 2.408 | 1.342–4.322 | 0.003 | 0.0 | 0.000 |
| Dementia | Dementia (k=14) | Collapsed | Adjusted | 5.595 | 3.551–8.815 | <0.001 | 56.8 | 0.383 |
|  | Dementia (k=3) | ≤6 months | Adjusted | 5.675 | 2.677–12.028 | <0.001 | 0.0 | 0.000 |
|  | Dementia (k=4) | >6–12 months | Adjusted | 3.959 | 1.461–10.728 | 0.007 | 71.1 | 0.686 |
|  | Dementia (k=7) | >12 months | Adjusted | 6.717 | 4.206–10.725 | <0.001 | 0.0 | 0.000 |
|  | Dementia (k=10) | Collapsed | Unadjusted | 4.903 | 2.066–11.634 | <0.001 | 94.0 | 1.654 |
|  | Dementia (k=3) | ≤6 months | Unadjusted | 5.373 | 0.810–35.655 | 0.082 | 61.2 | 1.685 |
|  | Dementia (k=5) | >6–12 months | Unadjusted | 4.090 | 1.428–11.716 | 0.009 | 82.8 | 0.998 |
|  | Dementia (k=5) | >12 months | Unadjusted | 3.572 | 1.047–12.185 | 0.042 | 94.7 | 1.741 |
| Institutionalisation | Institutionalisation (k=11) | Collapsed | Adjusted | 2.199 | 1.726–2.802 | <0.001 | 33.9 | 0.048 |
|  | Institutionalisation (k=6) | ≤6 months | Adjusted | 2.332 | 1.473–3.693 | <0.001 | 62.9 | 0.177 |
|  | Institutionalisation (k=5) | >6–12 months | Adjusted | 1.986 | 1.412–2.792 | <0.001 | 0.0 | 0.000 |
|  | Institutionalisation (k=18) | Collapsed | Unadjusted | 3.132 | 2.139–4.588 | <0.001 | 71.8 | 0.440 |
|  | Institutionalisation (k=9) | ≤6 months | Unadjusted | 2.903 | 1.427–5.906 | 0.003 | 78.0 | 0.845 |
|  | Institutionalisation (k=7) | >6–12 months | Unadjusted | 4.818 | 2.588–8.970 | <0.001 | 74.4 | 0.511 |
|  | Institutionalisation (k=5) | >12 months | Unadjusted | 2.559 | 1.383–4.736 | <0.003 | 62.4 | 0.226 |
| Readmission/revisit | Readmission/revisit (k=8) | Collapsed | Adjusted | 1.881 | 1.258–2.812 | 0.002 | 74.4 | 0.217 |
|  | Readmission/revisit (k=6) | ≤6 months | Adjusted | 1.992 | 1.220–3.254 | 0.006 | 74.6 | 0.278 |
|  | Readmission/revisit (k=2) | >6–12 months | Adjusted | 1.593 | 1.107–2.293 | 0.012 | 0.0 | 0.000 |
|  | Readmission/revisit (k=23) | Collapsed | Unadjusted | 1.626 | 1.309–2.020 | <0.001 | 57.7 | 0.125 |
|  | Readmission/revisit (k=22) | ≤6 months | Unadjusted | 1.649 | 1.303–2.087 | <0.001 | 57.9 | 0.148 |
|  | Readmission/revisit (k=2) | >6–12 months | Unadjusted | 1.623 | 1.226–2.150 | 0.001 | 0.0 | 0.000 |
| Mortality | Mortality (k=32) | Collapsed | Adjusted | 2.230 | 1.826–2.725 | <0.001 | 60.9 | 0.174 |
|  | Mortality (k=21) | ≤6 months | Adjusted | 2.670 | 2.230–3.196 | <0.001 | 18.1 | 0.031 |
|  | Mortality (k=9) | >6–12 months | Adjusted | 1.964 | 1.549–2.491 | <0.001 | 0.0 | 0.000 |
|  | Mortality (k=6) | >12 months | Adjusted | 1.270 | 0.733–1.203 | 0.394 | 73.3 | 0.289 |
|  | Mortality (k=138) | Collapsed | Unadjusted | 2.626 | 2.392–2.883 | <0.001 | 84.0 | 0.193 |
|  | Mortality (k=83) | ≤6 months | Unadjusted | 2.999 | 2.620–3.434 | <0.001 | 77.2 | 0.206 |
|  | Mortality (k=66) | >6–12 months | Unadjusted | 2.431 | 2.139–2.762 | <0.001 | 76.5 | 0.172 |
|  | Mortality (k=37) | >12 months | Unadjusted | 2.167 | 1.780–2.636 | <0.001 | 85.1 | 0.251 |

Footnote**.** Data not presented when less than 2 studies available. Cognitive change categorical: all unadjusted results

The total number of articles in specific categories may differ from the overall total because a single article can be included in multiple follow-up time points. Additionally, an article may be categorized as both adjusted and unadjusted depending on the adjustment status at specific follow-up points (e.g., in McCusker’s study, the 6-month data were unadjusted, while the 1-year data were adjusted). As a result, the total number of articles in the adjusted and unadjusted analyses may differ from the total number in the main analysis. For example, in the cognitive continuous data category, the total number of articles is 47, but when classified by model adjustment, it becomes 48 because McCusker’s study is counted twice—once as adjusted and once as unadjusted.

# Table S11. Covariates adjusted and unadjusted data sensitivity analyses. Continuous data results

| **Clinical outcome** | **Sub-group**  **(number of studies)** | **Timepoint** | **Model adjustment** | **Hedges's g** | **95% CI** | **p-value** | **I²** (%) | **τ²** |
| --- | --- | --- | --- | --- | --- | --- | --- | --- |
| Cognition | *Objective cognition measure* | | | | | | | |
|  | Cognitive change (k=6) | Collapsed | Adjusted | -0.158 | -0.242– -0.075 | <0.001 | 61.2 | 0.006 |
|  | Cognitive change (k=6) | ≤6 months | Adjusted | -0.115 | -0.223– -0.008 | 0.035 | 57.9 | 0.010 |
|  | Cognitive change (k=3) | >6–12 months | Adjusted | -0.095 | -0.350– 0.159 | 0.463 | 81.1 | 0.041 |
|  | Cognitive change (k=3) | >12 months | Adjusted | -0.243 | -0.312– -0.174 | <0.001 | 0.0 | 0.000 |
|  | Cognitive change (k=7) | Collapsed | Unadjusted | -0.265 | -0.497– -0.033 | 0.025 | 80.8 | 0.076 |
|  | Cognitive change (k=3) | ≤6 months | Unadjusted | -0.345 | -0.547– -0.143 | 0.001 | 0.0 | 0.000 |
|  | Cognitive change (k=2) | >6–12 months | Unadjusted | 0.065 | -0.245– 0.376 | 0.680 | 25.1 | 0.013 |
|  | Cognitive performance (k=14) | Collapsed | Adjusted | -0.452 | -0.622– -0.283 | <0.001 | 95.1 | 0.086 |
|  | Cognitive performance (k=13) | ≤6 months | Adjusted | -0.422 | -0.619– -0.225 | <0.001 | 92.3 | 0.105 |
|  | Cognitive performance (k=8) | >6–12 months | Adjusted | -0.462 | -0.728– -0.196 | 0.001 | 91.8 | 0.128 |
|  | Cognitive performance (k=4) | >12 months | Adjusted | -0.519 | -0.875– -0.162 | 0.004 | 97.0 | 0.117 |
|  | Cognitive performance (k=24) | Collapsed | Unadjusted | -0.800 | -1.007–0.593 | <0.001 | 92.9 | 0.238 |
|  | Cognitive performance (k=16) | ≤6 months | Unadjusted | -0.832 | -1.052– -0.613 | <0.001 | 87.5 | 0.161 |
|  | Cognitive performance (k=9) | >6–12 months | Unadjusted | -0.747 | -1.021– -0.473 | <0.001 | 79.8 | 0.130 |
|  | Cognitive performance (k=6) | >12 months | Unadjusted | -0.957 | -1.702– -0.212 | 0.012 | 97.2 | 0.830 |
|  | *Subjective cognition measure* | | | | | | | |
|  | Cognitive performance (k=2) | Collapsed | Adjusted | -0.174 | -0.325–0.022 | 0.025 | 0.0 | 0.000 |
|  | Cognitive performance (k=5) | Collapsed | Unadjusted | -0.207 | -0.405– -0.009 | 0.040 | 80.7 | 0.038 |
|  | Cognitive performance (k=4) | ≤6 months | Unadjusted | -0.283 | -0.438– -0.127 | <0.001 | 47.9 | 0.012 |
| Functional outcome | Functional change (k=5) | Collapsed | Adjusted | -0.206 | -0.384– -0.028 | 0.024 | 66.7 | 0.026 |
|  | Functional change (k=5) | ≤6 months | Adjusted | -0.223 | -0.423– -0.023 | 0.029 | 70.6 | 0.035 |
|  | Functional change (k=5) | Collapsed | Unadjusted | -0.798 | -1.539– -0.056 | 0.035 | 92.8 | 0.650 |
|  | Functional change (k=2) | ≤6 months | Unadjusted | -1.202 | -3.482–1.079 | 0.302 | 97.7 | 2.645 |
|  | Functional change (k=2) | >12 months | Unadjusted | -0.279 | -0.887–0.329 | 0.368 | 76.7 | 0.149 |
|  | Functional performance (k=10) | Collapsed | Adjusted | -0.590 | -0.930– -0.249 | 0.001 | 97.7 | 0.281 |
|  | Functional performance (k=7) | ≤6 months | Adjusted | -0.715 | -1.251– -0.179 | 0.009 | 98.4 | 0.511 |
|  | Functional performance (k=5) | >6–12 months | Adjusted | -0.289 | -0.543– -0.034 | 0.026 | 77.3 | 0.058 |
|  | Functional performance (k=3) | >12 months | Adjusted | -0.206 | -0.301– -0.111 | <0.001 | 0.5 | 0.000 |
|  | Functional performance (k=28) | Collapsed | Unadjusted | -0.604 | -0.867– -0.342 | <0.001 | 97.5 | 0.477 |
|  | Functional performance (k=23) | ≤6 months | Unadjusted | -0.574 | -0.878– -0.271 | <0.001 | 97.3 | 0.527 |
|  | Functional performance (k=8) | >6–12 months | Unadjusted | -0.737 | -1.177– -0.297 | 0.001 | 96.6 | 0.378 |
|  | Functional performance (k=5) | >12 months | Unadjusted | -0.726 | -1.307– -0.145 | 0.014 | 93.7 | 0.397 |
| Quality of life | Poor quality of life (k=7) | Collapsed | Adjusted | -0.315 | -0.464– -0.166 | <0.001 | 85.1 | 0.031 |
|  | Poor quality of life (k=4) | ≤6 months | Adjusted | -0.422 | -0.823– -0.021 | 0.039 | 92.2 | 0.150 |
|  | Poor quality of life (k=4) | >6–12 months | Adjusted | -0.211 | -0.310– -0.111 | <0.001 | 0.0 | 0.000 |
|  | Poor quality of life (k=15) | Collapsed | Unadjusted | -0.509 | -0.681– -0.338 | <0.001 | 95.0 | 0.097 |
|  | Poor quality of life (k=10) | ≤6 months | Unadjusted | -0.306 | -0.454– -0.158 | <0.001 | 88.3 | 0.043 |
|  | Poor quality of life (k=4) | >6–12 months | Unadjusted | -0.583 | -0.874– -0.293 | <0.001 | 91.4 | 0.072 |
| Mental health | Poor mental health (k=2) | Collapsed | Adjusted | -0.281 | -0.747– 0.184 | 0.236 | 71.0 | 0.082 |
|  | Poor mental health (k=2) | ≤6 months | Adjusted | -0.310 | -0.681– -0.061 | 0.101 | 10.0 | 0.009 |
|  | Poor mental health (k=9) | Collapsed | Unadjusted | -0.336 | -0.514– -0.157 | <0.001 | 78.0 | 0.052 |
|  | Poor mental health (k=8) | ≤6 months | Unadjusted | -0.321 | -0.523– -0.119 | 0.002 | 76.6 | 0.057 |
|  | Poor mental health (k=4) | >6–12 months | Unadjusted | -0.478 | -0.698– -0.257 | <0.001 | 1.0 | 0.001 |

Footnote: Data not presented when less than 2 studies available. Subjective cognitive measure cognitive change categorical and continuous data not available (study not available).

Subjective cognitive measure cognitive performance categorical: all studies were adjusted except one study

# Table S12. Sample size sensitivity analysis. Continuous data results

| **Clinical outcome** | **Sub-group**  **(number of studies)** | **Reported sample size** | **Timepoint** | **Hedges's g** | **95% CI** | **p-value** | **I²** (%) | **τ²** |
| --- | --- | --- | --- | --- | --- | --- | --- | --- |
| Cognition | *Objective cognition* |  | | | | | | |
|  | Cognitive change (k=13) | Follow-up and baseline | Collapsed | -0.192 | -0.285– -0.099 | <0.001 | 73.1 | 0.017 |
|  | Cognitive change (k=9) | Follow-up and baseline | ≤6 months | -0.158 | -0.258– -0.058 | 0.002 | 52.5 | 0.011 |
|  | Cognitive change (k=5) | Follow-up and baseline | >6–12 months | -0.052 | -0.254– 0.150 | 0.613 | 71.0 | 0.035 |
|  | Cognitive change (k=4) | Follow-up and baseline | >12 months | -0.154 | -0.302– -0.006 | 0.042 | 73.5 | 0.015 |
|  | Cognitive change (k=8) | Follow-up only | Collapsed | -0.185 | -0.258– -0.112 | <0.001 | 44.3 | 0.004 |
|  | Cognitive change (k=6) | Follow-up only | ≤6 months | -0.143 | -0.258– -0.029 | 0.014 | 58.1 | 0.011 |
|  | Cognitive change (k=4) | Follow-up only | >6–12 months | -0.102 | -0.316– 0.113 | 0.354 | 71.7 | 0.032 |
|  | Cognitive change (k=2) | Follow-up only | >12 months | -0.247 | -0.316– -0.177 | <0.001 | 0.0 | 0.000 |
|  | Cognitive performance (k=36) | Follow-up and baseline | Collapsed | -0.689 | -0.822– -0.555 | <0.001 | 94.7 | 0.141 |
|  | Cognitive performance (k=28) | Follow-up and baseline | ≤6 months | -0.657 | -0.810– -0.505 | <0.001 | 91.5 | 0.136 |
|  | Cognitive performance (k=17) | Follow-up and baseline | >6–12 months | -0.601 | -0.790– -0.411 | <0.001 | 88.2 | 0.127 |
|  | Cognitive performance (k=11) | Follow-up and baseline | >12 months | -0.794 | -1.096– -0.492 | <0.001 | 96.7 | 0.235 |
|  | Cognitive performance (k=30) | Follow-up only | Collapsed | -0.752 | -0.911– -0.593 | <0.001 | 95.3 | 0.168 |
|  | Cognitive performance (k=23) | Follow-up only | ≤6 months | -0.707 | -0.890– -0.524 | <0.001 | 92.6 | 0.163 |
|  | Cognitive performance (k=14) | Follow-up only | >6–12 months | -0.663 | -0.901– -0.424 | <0.001 | 88.9 | 0.169 |
|  | Cognitive performance (k=9) | Follow-up only | >12 months | -0.856 | -1.209– -0.503 | <0.001 | 97.2 | 0.263 |
|  | *Subjective cognition* |  | | | | | | |
|  | Cognitive performance (k=7) | Follow-up and baseline | Collapsed | -0.191 | -0.336– -0.046 | 0.010 | 72.6 | 0.025 |
|  | Cognitive performance (k=5) | Follow-up and baseline | ≤6 months | -0.254 | -0.405– -0.103 | 0.001 | 48.2 | 0.014 |
|  | Cognitive performance (k=5) | Follow-up only | Collapsed | -0.209 | -0.393– -0.026 | 0.026 | 80.1 | 0.032 |
|  | Cognitive performance (k=3) | Follow-up only | ≤6 months | -0.333 | -0.491– -0.174 | <0.001 | 29.0 | 0.007 |
| Functional outcome | Functional change (k=10) | Follow-up and baseline | Collapsed | -0.432 | -0.694– -0.170 | 0.001 | 87.3 | 0.143 |
|  | Functional change (k=7) | Follow-up and baseline | ≤6 months | -0.433 | -0.764– -0.102 | 0.010 | 90.3 | 0.172 |
|  | Functional change (k=2) | Follow-up and baseline | >12 months | -0.279 | -0.887– 0.329 | 0.368 | 76.7 | 0.149 |
|  | Functional change (k=7) | Follow-up only | Collapsed | -0.258 | -0.462– -0.054 | 0.013 | 71.2 | 0.047 |
|  | Functional change (k=5) | Follow-up only | ≤6 months | -0.165 | -0.367– 0.036 | 0.108 | 68.4 | 0.034 |
|  | Functional performance (k=37) | Follow-up and baseline | Collapsed | -0.616 | -0.827– -0.405 | <0.001 | 97.7 | 0.404 |
|  | Functional performance (k=30) | Follow-up and baseline | ≤6 months | -0.608 | -0.866– -0.349 | <0.001 | 97.6 | 0.502 |
|  | Functional performance (k=13) | Follow-up and baseline | >6–12 months | -0.581 | -0.892– -0.270 | <0.001 | 95.6 | 0.297 |
|  | Functional performance (k=7) | Follow-up and baseline | >12 months | -0.544 | -0.851– -0.237 | 0.001 | 92.2 | 0.144 |
|  | Functional performance (k=31) | Follow-up only | Collapsed | -0.644 | -0.895– -0.393 | <0.001 | 98.0 | 0.481 |
|  | Functional performance (k=25) | Follow-up only | ≤6 months | -0.624 | -0.940– -0.309 | <0.001 | 97.9 | 0.624 |
|  | Functional performance (k=12) | Follow-up only | >6–12 months | -0.607 | -0.951– -0.264 | 0.001 | 95.8 | 0.335 |
|  | Functional performance (k=6) | Follow-up only | >12 months | -0.617 | -0.964– -0.269 | 0.001 | 93.4 | 0.160 |
| Quality of life | Poor quality of life (k=22) | Follow-up and baseline | Collapsed | -0.443 | -0.563 – -0.323 | <0.001 | 93.6 | 0.068 |
|  | Poor quality of life (k=14) | Follow-up and baseline | ≤6 months | -0.331 | -0.465 – -0.196 | <0.001 | 88.8 | 0.051 |
|  | Poor quality of life (k=8) | Follow-up and baseline | >6–12 months | -0.419 | -0.613 – -0.225 | <0.001 | 89.5 | 0.062 |
|  | Poor quality of life (k=15) | Follow-up only | Collapsed | -0.549 | -0.718 – -0.381 | <0.001 | 95.5 | 0.094 |
|  | Poor quality of life (k=8) | Follow-up only | ≤6 months | -0.420 | -0.675 – -0.165 | 0.001 | 93.1 | 0.116 |
|  | Poor quality of life (k=6) | Follow-up only | >6–12 months | -0.493 | -0.729 – -0.257 | <0.001 | 89.6 | 0.067 |

Footnote: Data not presented when less than 2 studies available. Baseline sample size was used when a study did not report follow-up sample size separately for delirium and no-delirium group.

Baseline sample sizes were used for 10 studies on cognition, 8 studies on functional outcomes, and 7 studies on quality-of-life outcomes due to the absence of follow-up sample sizes.

All studies in this analyses were continuous data.

For all other outcomes, follow-up sample sizes were used.

A single study could report multiple outcomes

# Table S13. Functional outcomes sensitivity analyses with and without (frailty, fatigue, and/or fall)

| **Outcome** | **Data type** | **Time** | **Summary statistics with (frailty, fatigue, and/or fall) outcomes** | **Time** | **Summary statistics without (frailty, fatigue, and/or fall) outcomes** |
| --- | --- | --- | --- | --- | --- |
| Functional performance (impairment vs no impairment) | Cat | Collapsed (k=27) | OR=2.187, 95% CI: 1.687–2.836, p < 0.00, I² = 90.8, τ²= 0.363 | Collapsed (k=21) | OR= 2.200, 95% CI: 1.594–3.036, p<0.001, I² = 91.4, τ²= 0.452 |
|  |  | ≤6 months (k=16) | OR= 1.560, 95% CI: 1.175–2.070, p=0.002, I² = 86.5, τ²= 0.227 | ≤6 months (k=14) | OR= 1.573, 95% CI: 1.116–2.216, p=0.010, I² = 88.2, τ²= 0.314 |
|  |  | >6–12 months (k=8) | OR= 2.318, 95% CI: 1.674–3.210, p < 0.001, I² = 75.0, τ²= 0.127 | >6-12 months (k=7) | OR=2.481, 95% CI: 1.688–3.645, p<0.001, I² = 69.4, τ²= 0.150 |
|  |  | >12 months: (k=7) | OR= 4.077, 95% CI: 1.680 – 9.894, p= 0.002, I² = 89.3, τ²= 1.250 | >12 months (k=5) | OR= 4.870, 95% CI: 1.609–14.741, p=0.005, I² = 89.6, τ²= 1.405 |
| Functional performance (scores at follow-up) | Con | Collapsed (k=37) | g= -0.616, 95% CI: -0.827 – -0.405, p<0.001, I² = 97.7, τ²= 0.404 | Collapsed (k=34) | g= -0.621, 95% CI: -0.847 – -0.396, p<0.001, I² = 97.8, τ²= 0.423 |
|  |  | ≤6 months (k=30) | The outcomes were not available for this time point | ≤6 months (k=30) | g= -0.608, 95% CI: -0.866 – -0.349, p<0.001, I² = 97.6, τ²= 0.502 |
|  |  | >6–12 months (k=13) | g= -0.581, 95% CI: -0.892 – -0.270, p<0.001, I² = 95.6, τ²= 0.297 | >6-12 month (k=12) | g= -0.617, 95% CI: 0.948 – -0.287, p<0.001, I² = 95.8, τ²= 0.309 |
|  |  | >12 months (k=7) | g= -0.544, 95% CI: -0.851 – -0.237, p=0.001, I² = 92.2, τ²= 0.144 | >12 months (k=5) | g= -0.404, 95% CI: -0.687 – -0.121, p=0.005, I² = 84.0, τ²= 0.074 |

Footnote**:** Frailty, fatigue, fall were considered as functional impairment in this review

# Table S14. Outlier analyses for long-term clinical outcomes of delirium after hospital discharge

| **Clinical outcomes** | **Data type** | **Time** | **Summary statistics before the outlier removed** | **Outliers** | **Relative weight of outliers** | **Summary statistics after the outlier removed** |
| --- | --- | --- | --- | --- | --- | --- |
| Objective (cognitive change) | Con | Collapsed | g= -0.192, 95%CI: -0.285– -0.099, p<0.001, I² = 73.1, τ²= 0.017 | Krogseth 2016 | 6.53% | g= -0.150, 95%CI: -0.222– -0.078, p<0.001, I²=51.9, τ²= 0.006 |
| Objective (Cognitive performance) | Con | Collapsed | g=-0.689, 95%CI: -0.822– -0.555, p<0.001, I² = 94.7, τ²= 0.141 | Serrano-Duenas 2005 | 2.90% | g=-0.593, 95%CI: -0.706– -0.480, p<0.001, I²=91.1, τ²= 0.089 |
|  |  |  |  | Vives-Borrás 2019 | 2.65% |  |
|  |  | ≤6 months | g=-0.657, 95%CI: -0.810– -0.505, p<0.001, I² =91.5, τ²= 0.136 | Serrano-Duenas 2005 | 2.47% | g=-0.578, 95%CI: -0.715– -0.440, p<0.001, I²=89.1, τ²= 0.097 |
|  |  |  |  | Vives-Borrás 2019 | 3.58% |  |
|  |  | >6–12 months | g=-0.601, 95%CI: -0.790– -0.411, p<0.001, I² =88.2, τ²= 0.127 | Serrano-Duenas 2005 | 3.88% | g= -0.569, 95%CI: -0.759– -0.378, p<0.001, I²=88.4, τ²= 0.122 |
|  |  | >12 months | g=-0.794, 95%CI: -1.096– -0.492, p<0.001, I² =96.7, τ²= 0.235 | Serrano-Duenas 2005 | 8.29% | g=-0.575, 95%CI: -0.785– -0.365, p<0.001, I²=92.8, τ²= 0.102 |
| Functional change | Cat | Collapsed | OR= 2.038, 95%CI:1.742–2.384, p<0.01, I² = 20.1, τ²= 0.028 | Tavares 2021 | 0.06% | OR=2.036, 95%CI: 1.734–2.390, p<0.001, I²=23.7, τ²= 0.032 |
|  |  | ≤6 months | OR=1.894, 95%CI: 1.564–2.293, p<0.001, I² =28.0, τ²= 0.042 | Tavares 2021 | 0.09% | OR=1.892, 95%CI: 1.555–2.303, p<0.001, I²=32.4, τ²= 0.048 |
| Functional change | Con | Collapsed | g=-0.432, 95%CI: -0.694– -0.170, p=0.001, I² = 87.3, τ²= 0.143 | Isaia 2009 | 7.45% | g=-0.236, 95%CI: -0.402– 0.071, p=0.005, I²=66.6, τ²= 0.038 |
|  |  | ≤6 months | g= -0.433, 95%CI: -0.764– -0.102, p= 0.010, I² =90.3, τ²= 0.172 | Isaia 2009 | 9.76% | g=-0.199, 95%CI: -0.375– -0.023, p=0.027, I²=65.4, τ²= 0.030 |
| Functional performance | Cat | Collapsed | OR=2.187, 95%CI: 1.687–2.836, p<0.001, I² = 90.8, τ²= 0.363 | Rawle 2021 | 3.68% | OR=1.971, 95%CI: 1.559–2.492, p<0.001, I²=88.2, τ²= 0.266 |
|  |  | >12 months | OR=4.077, 95%CI: 1.680–9.894, p=0.002, I² = 89.3, τ²= 1.250 | Rawle 2021 | 15.0% | OR=2.896, 95%CI: 1.478– 5.673, p=0.002, I²=76.5, τ²= 0.519 |
| Functional performance | Con | Collapsed | g=-0.616, 95%CI: -0.827– -0.405, p<0.001, I² = 97.7, τ²= 0.404 | Oldenbeuving 2011 | 2.68% | g=-0.591, 95%CI: -0.761– -0.421, p<0.001, I²=96.3, τ²= 0.240 |
|  |  |  |  | Monacelli 2018 | 2.81% |  |
|  |  | ≤6 months | g=-0.608, 95%CI: -0.866– -0.349, p<0.001, I² =97.6, τ²= 0.502 | Oldenbeuving 2011 | 3.28% | g=-0.580, 95%CI: -0.767– -0.393, p<0.001, I²=95.0, τ²= 0.234 |
|  |  |  |  | Monacelli 2018 | 3.41% |  |
| Quality of life | Con | Collapsed | g= -0.443, 95% CI: -0.563– -0.323, p <0.001, I² = 93.6, τ² = 0.068 | Chen 2017 | 3.01% | g= -0.373, 95% CI: -0.479– -0.267, p<0.001, I² = 91.7, τ² = 0.049 |
| Dementia | Cat | ≤6 months | OR= 5.603, 95% CI: 2.716–11.557, p< 0.001, I² = 20.6 τ² = 0.169 | Olofsson 2018 | 5.91% | OR=5.204, 95% CI: 2.390–11.329, p<0.001, I² = 28.7, τ² = 0.225 |
|  |  | >6–12 months | OR= 4.089, 95% CI: 1.926–8.681, p< 0.001, I² = 84.1, τ² = 0.926 | Olofsson 2018 | 4.90% | OR=3.531, 95% CI: 1.665–7.489, p=0.001, I² = 84.8, τ² = 0.865 |
| Mortality | Cat | ≤6 months | OR=2.934, 95%CI: 2.609–3.299, p<0.001, I² = 73.4, τ² = 0.186 | Bagienski 2017 | 0.15% | OR=2.891, 95%CI: 2.573–3.248, p<0.001, I² = 73.3, τ² = 0.180 |
|  |  |  |  | Bakker 2012 | 0.15% |  |
|  |  |  |  | Qu 2018 | 0.25% |  |
|  |  | >6–12 months | OR=2.384, 95%CI: 2.117–2.685, p<0.001, I² = 73.9, τ² = 0.161 | Bagienski 2017 | 0.65% | OR=2.354, 95%CI: 2.093–2.647, p<0.001, I² = 73.3, τ² = 0.154 |

Outliers: Studies whose effects estimates differ very substantially from the others through observation of the forest plot, funnel plot and the relative weight of the study against other included studies in the analysis. Data not presented when extreme outliers not observed

Subgroup analyses

No notable differences were observed for QoL, mental health, institutionalisation, mortality and dementia outcomes when comparing covariate-adjusted data with a mix of adjusted and unadjusted data, except for higher effect estimates for incident dementia in adjusted data compared to unadjusted data at >12-month follow-up (Table S11). Results for cognitive, functional and readmission outcomes were not consistent (Table S10 and Table S11). No meaningful differences were observed when using follow-up sample sizes alone compared to mixed follow-up and baseline sample sizes (Table S12). Removing extreme outliers led to a slight decrease in heterogeneity and effect sizes for cognition, function, QoL, dementia and mortality outcomes (Table S14).

# Table S15. Small study effect /publication bias investigations

Funnel plots for long-term clinical outcomes of delirium after hospital discharge

| Clinical outcomes | Data type | Funnel plots | Egger’s Test | | Trim and Fill | | |
| --- | --- | --- | --- | --- | --- | --- | --- |
|  | | | Intercept | p value  (1-tailed) | No. imputed  studies | OR/g | 95%CI |
| Objective (cognitive change) | Categorical | Only 7 studies available | - | - | - | - | - |
| Objective (cognitive change) | Continuous | 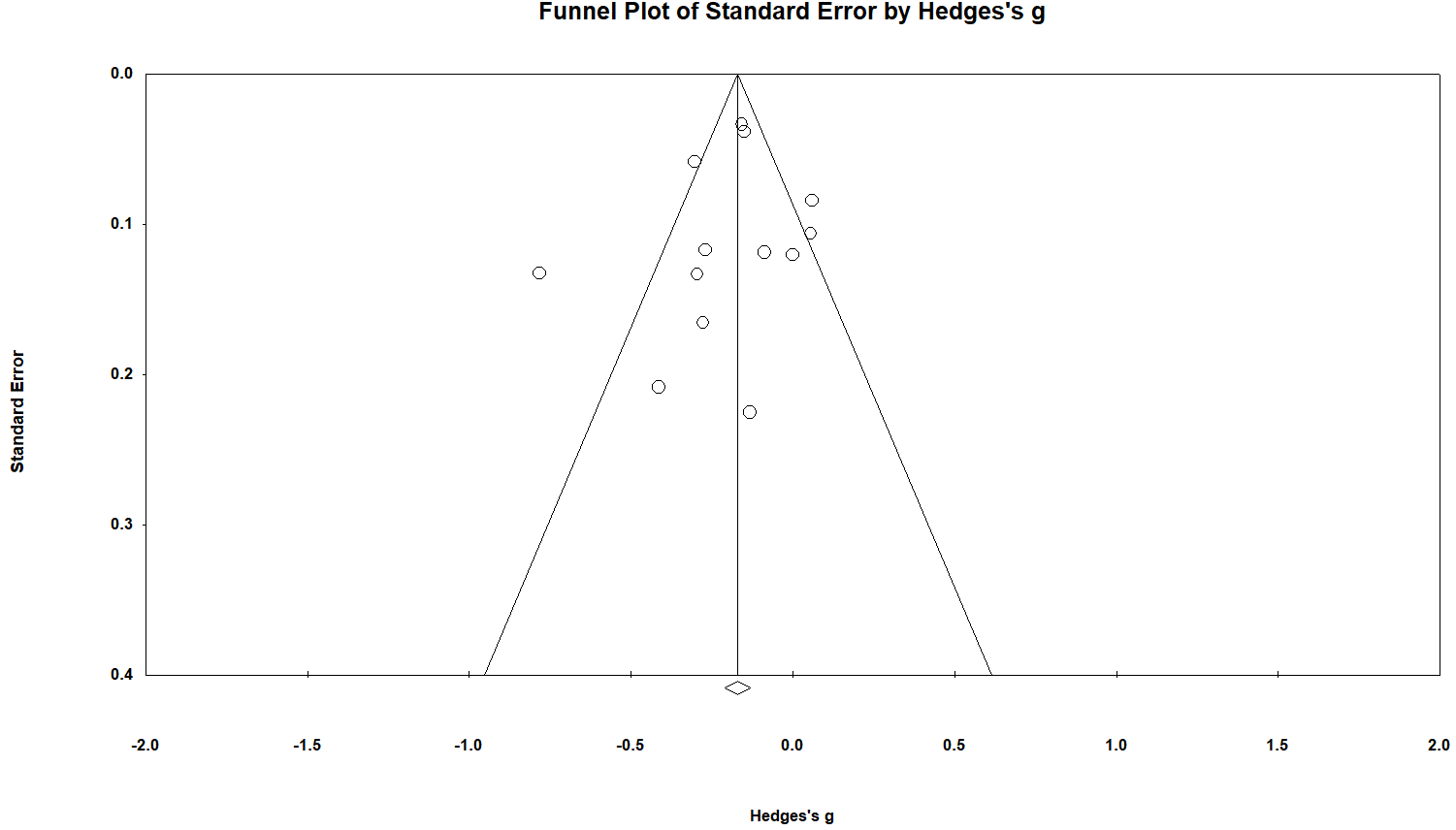 | -0.59 | 0.28 | - | - | - |
| Objective (cognitive performance) | Categorical | 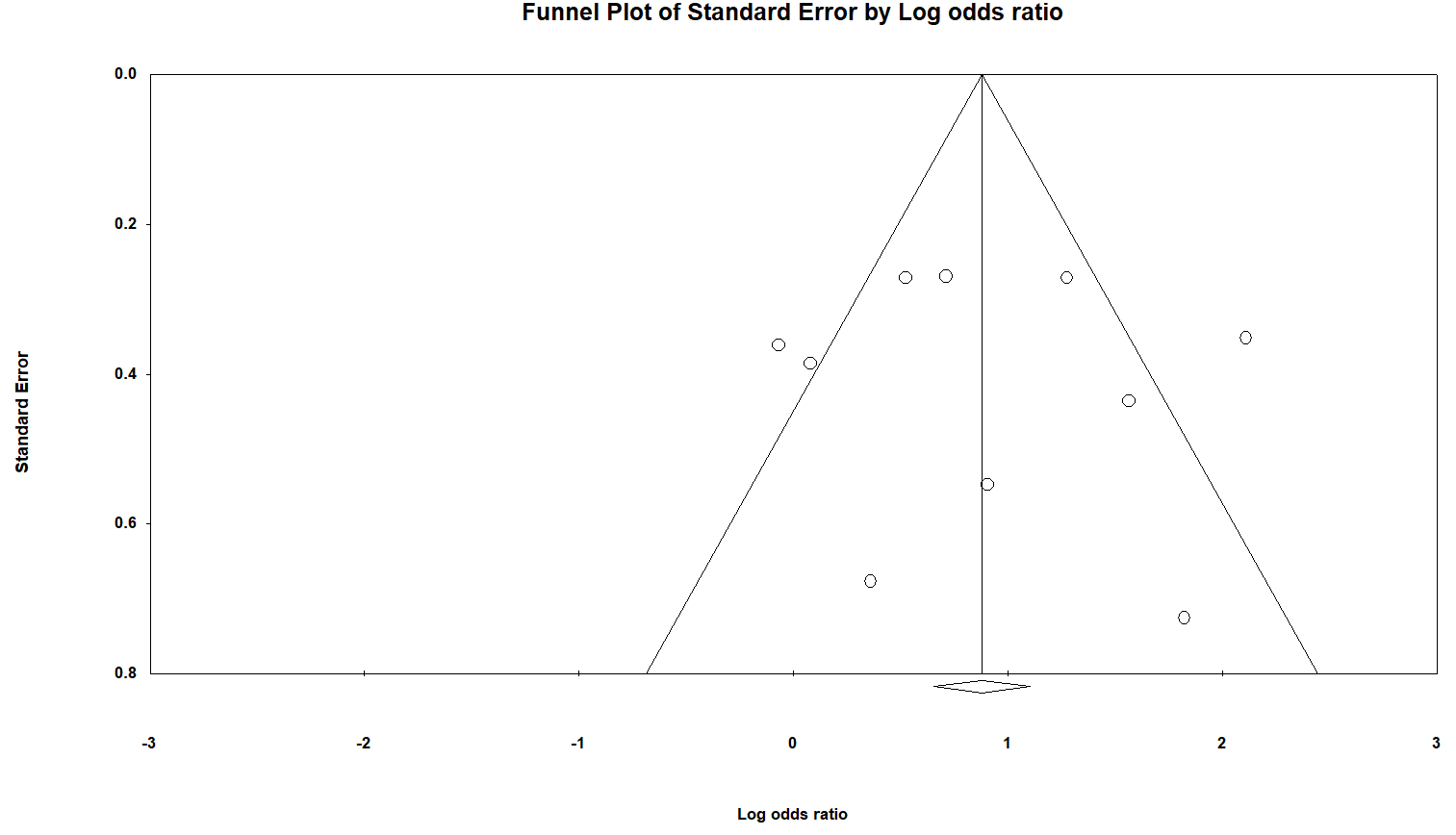 | 0.55 | 0.40 | - | - | - |
| Objective (cognitive performance) | Continuous | 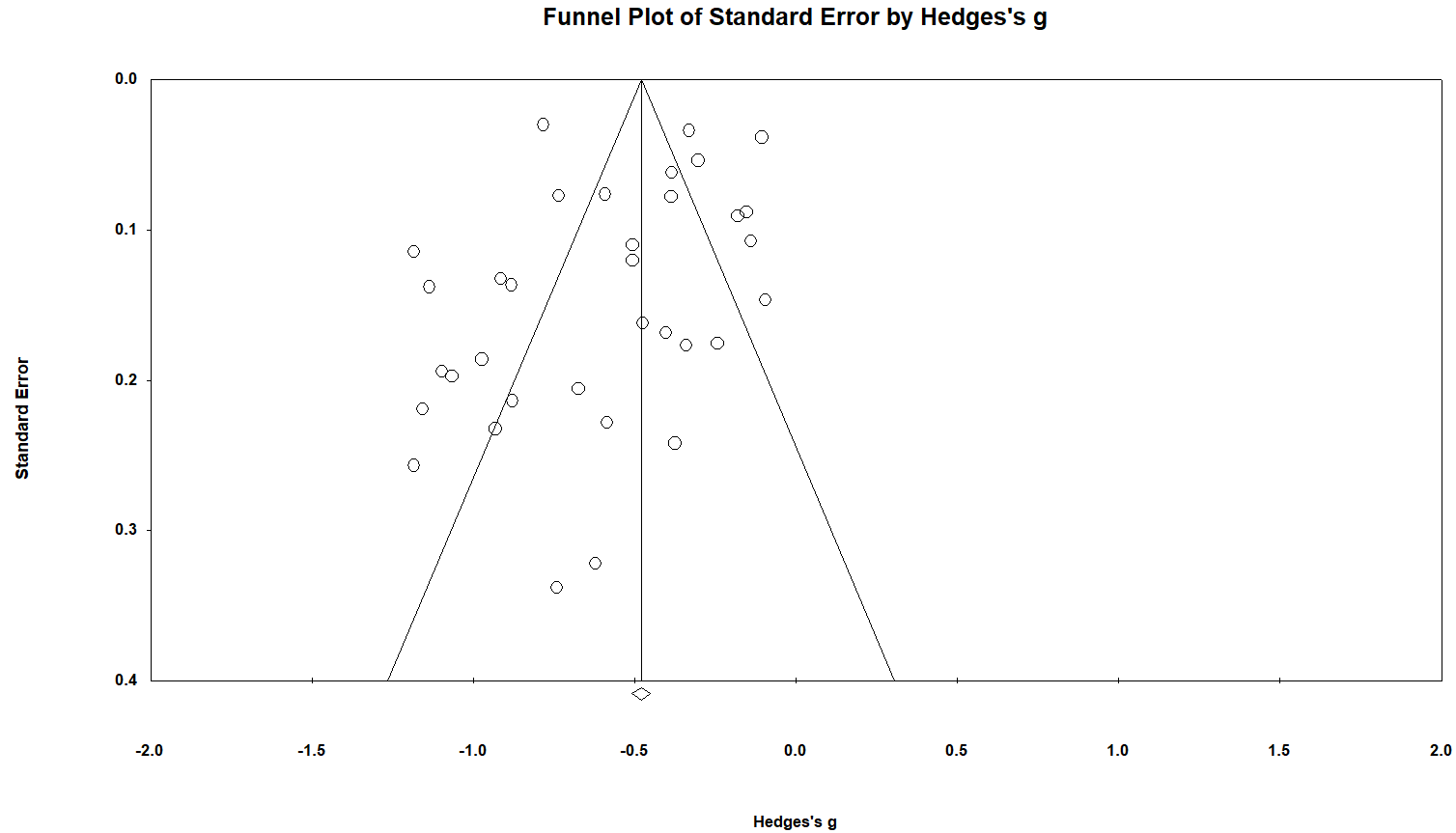 | -1.37 | 0.08 | - | - | - |
| Subjective (cognitive change | Categorical | No study available | - | - | - | - | - |
| Subjective (cognitive change | Continuous | No study available | - | - | - | - | - |
| Subjective (cognitive performance) | Categorical | Only 5 studies available | - | - | - | - | - |
| Subjective (cognitive performance) | Continuous | Only 7 studies available | - | - | - | - | - |
| Functional change | Categorical | 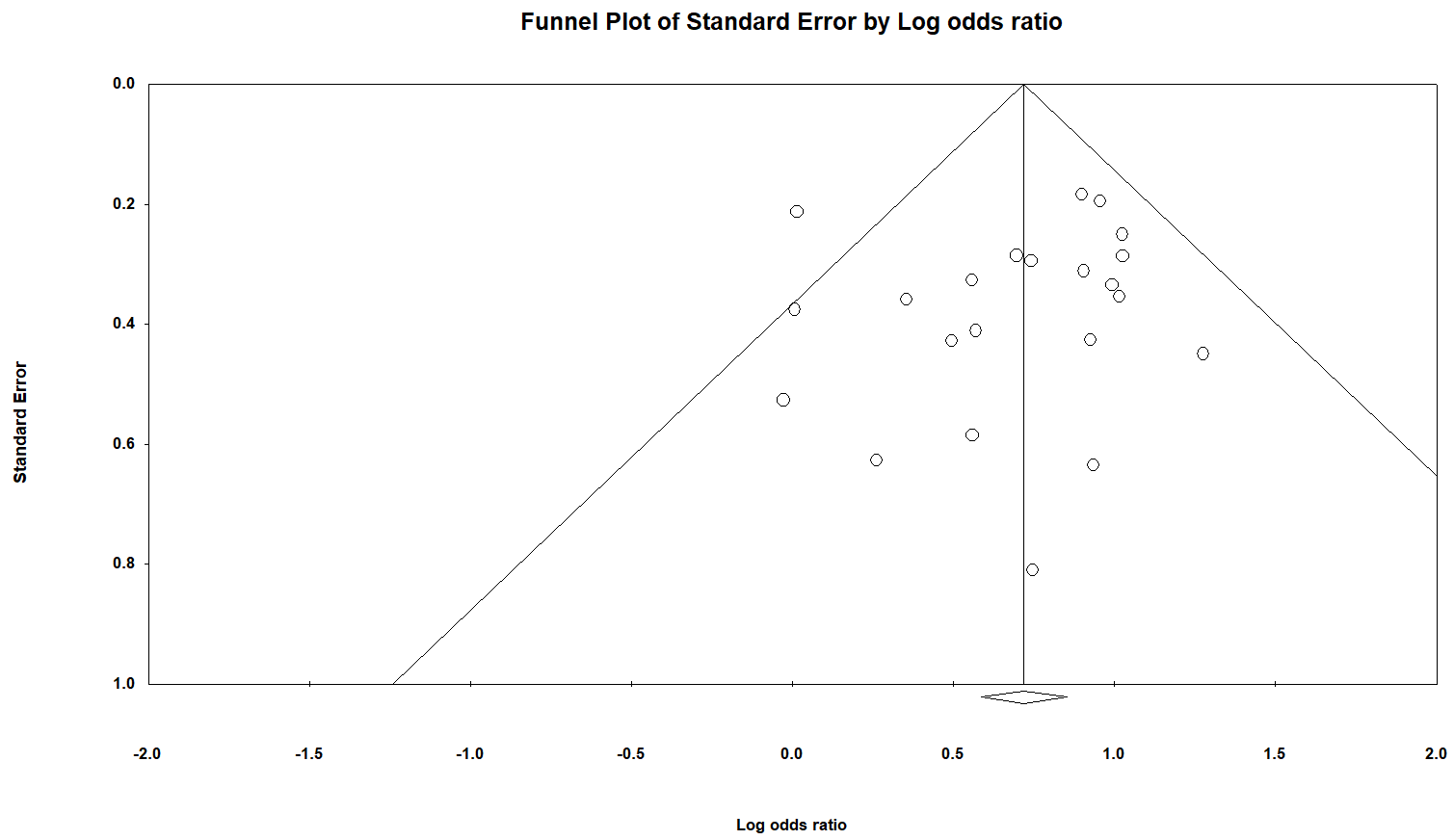 | -0.43 | 0.27 | - | - | - |
| Functional change | Continuous | Only 9 studies available | - | - | - | - | - |
| Functional performance | Categorical | 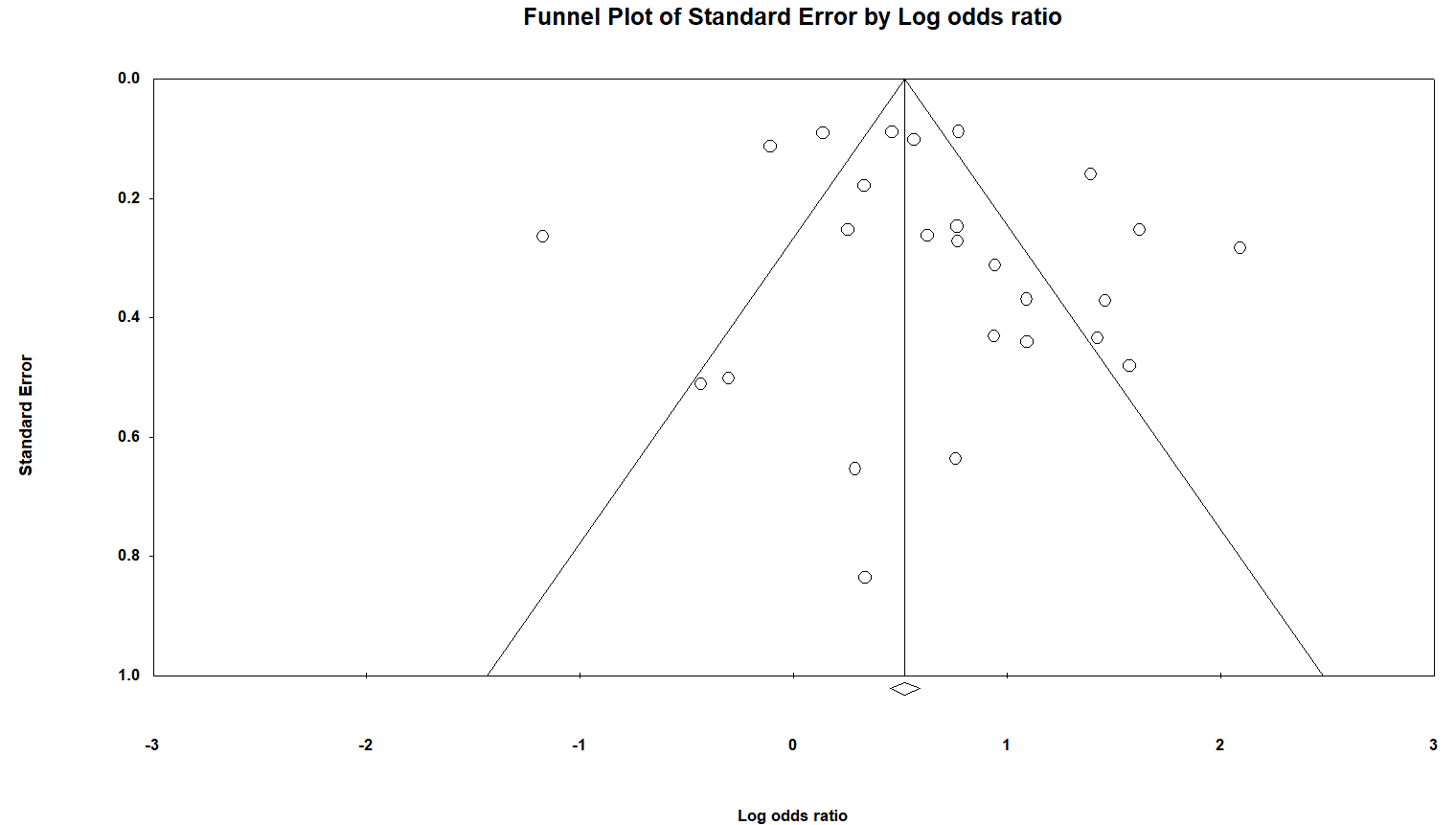 | 1.17 | 0.12 | - | - | - |
| Functional performance | Continuous | 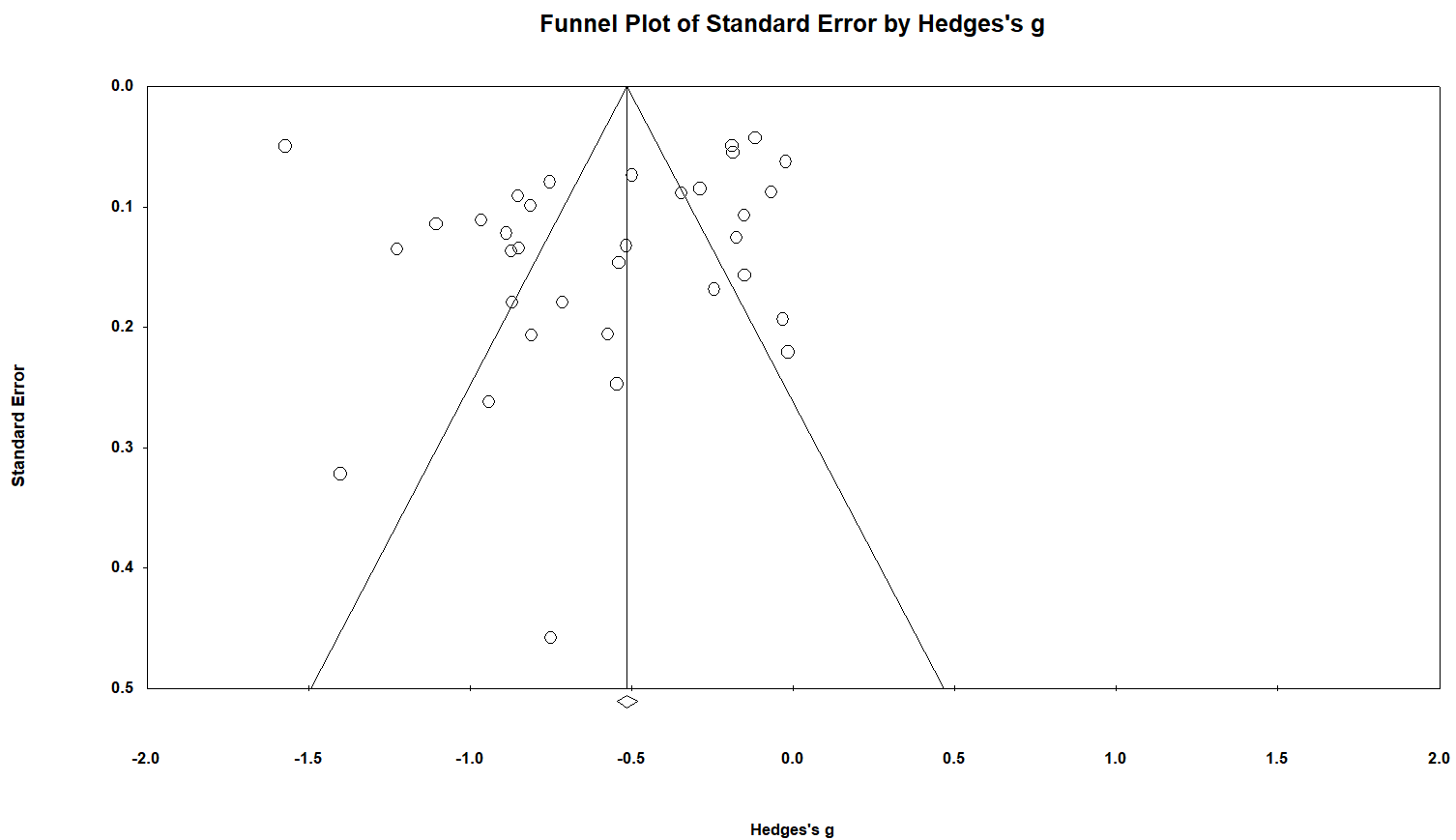 | -1.66 | 0.18 |  |  |  |
| Quality of life | Categorical | Only 4 studies available | - | - | - | - | - |
| Quality of life | Continuous | 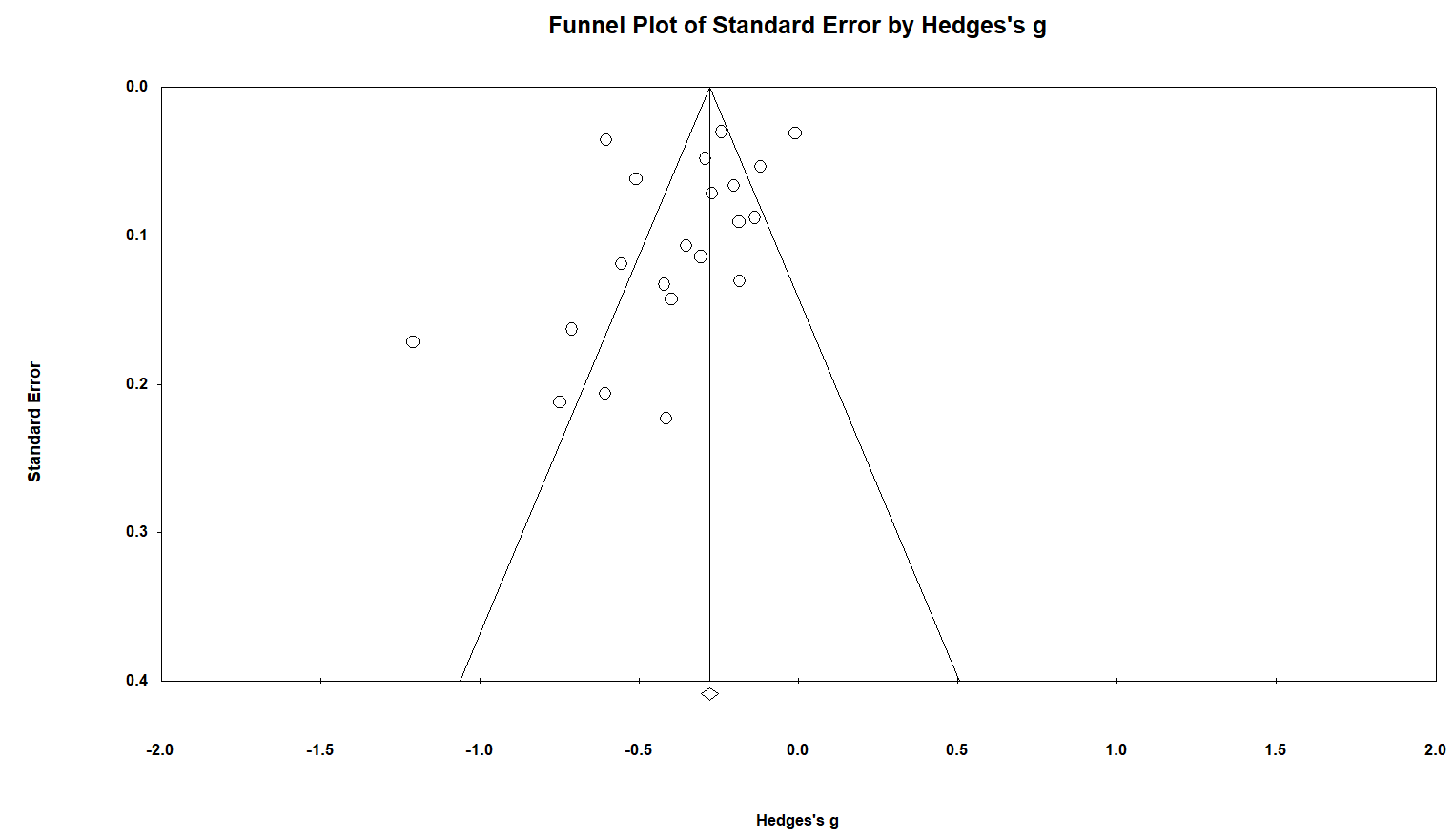 | -2.03 | 0.07 | - | - | - |
| Mental health | Categorical | 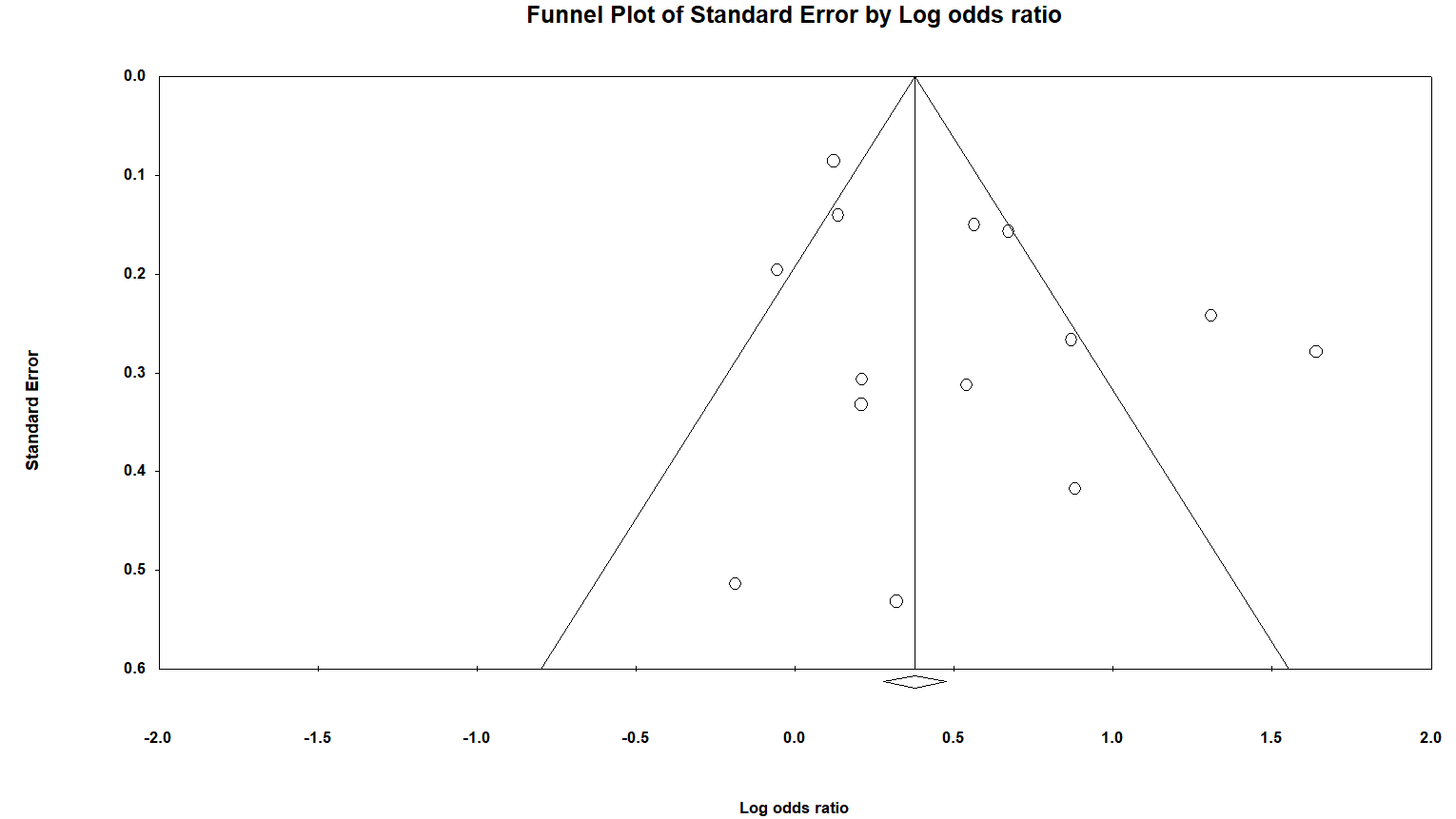 | 1.78 | 0.07 | - | - | - |
| Mental health | Continuous | 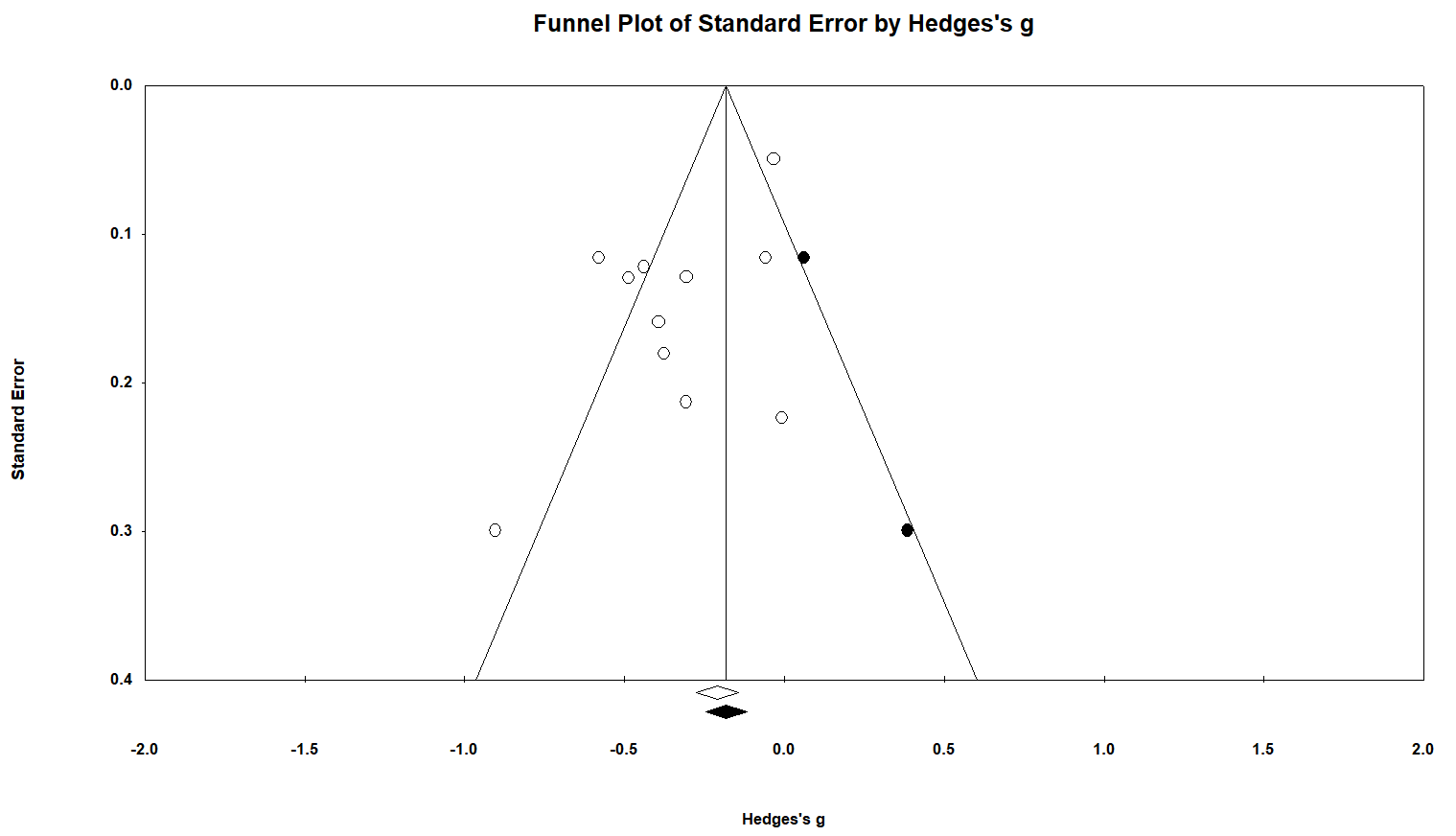 | -2.72 | 0.01 | 2 | -0.26 | -0.41 – -0.11 |
| Dementia |  | 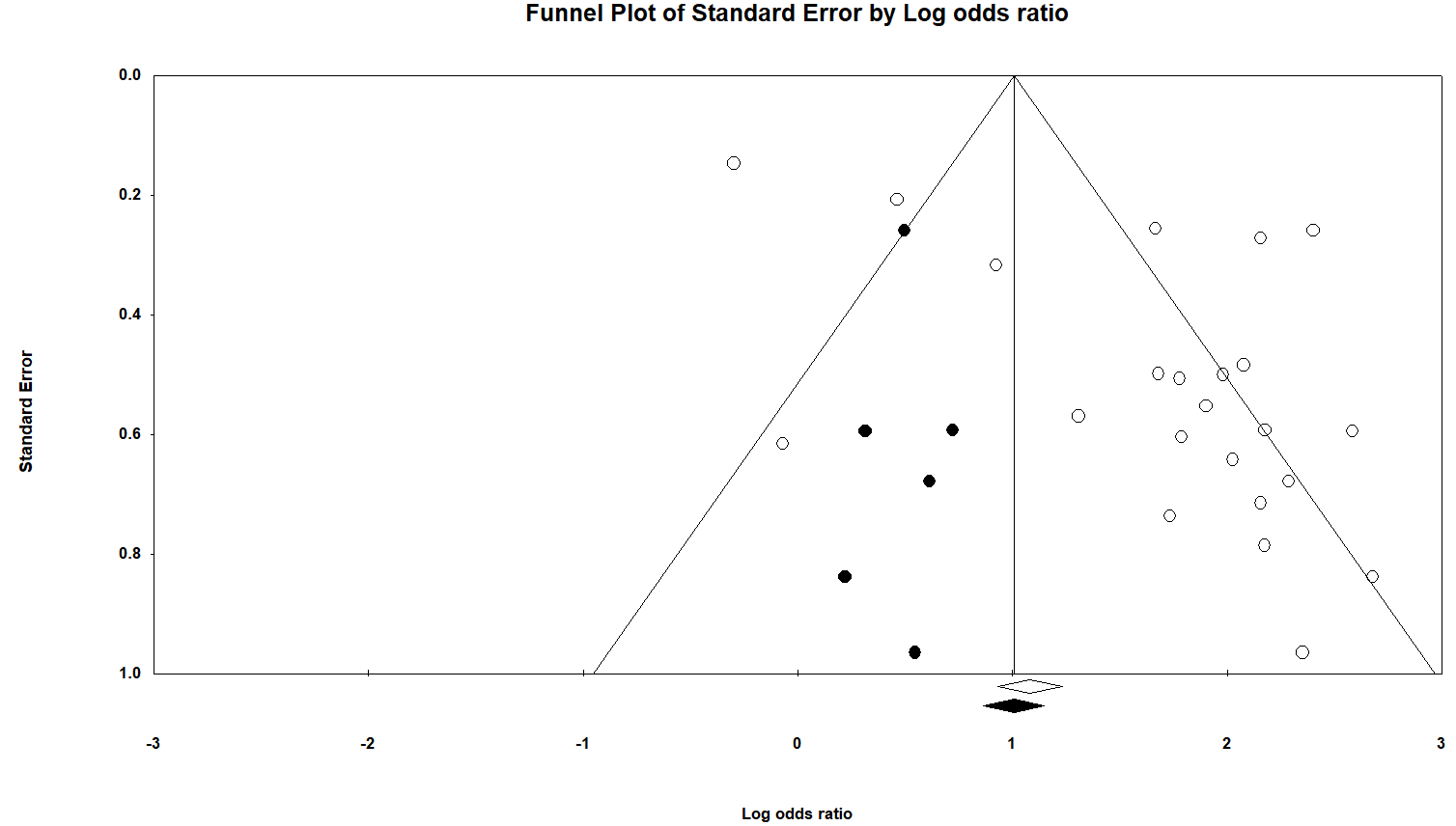 | 3.51 | <0.001 | 6 | 4.26 | 2.82 – 6.42 |
| Institutionalisation | Categorical | 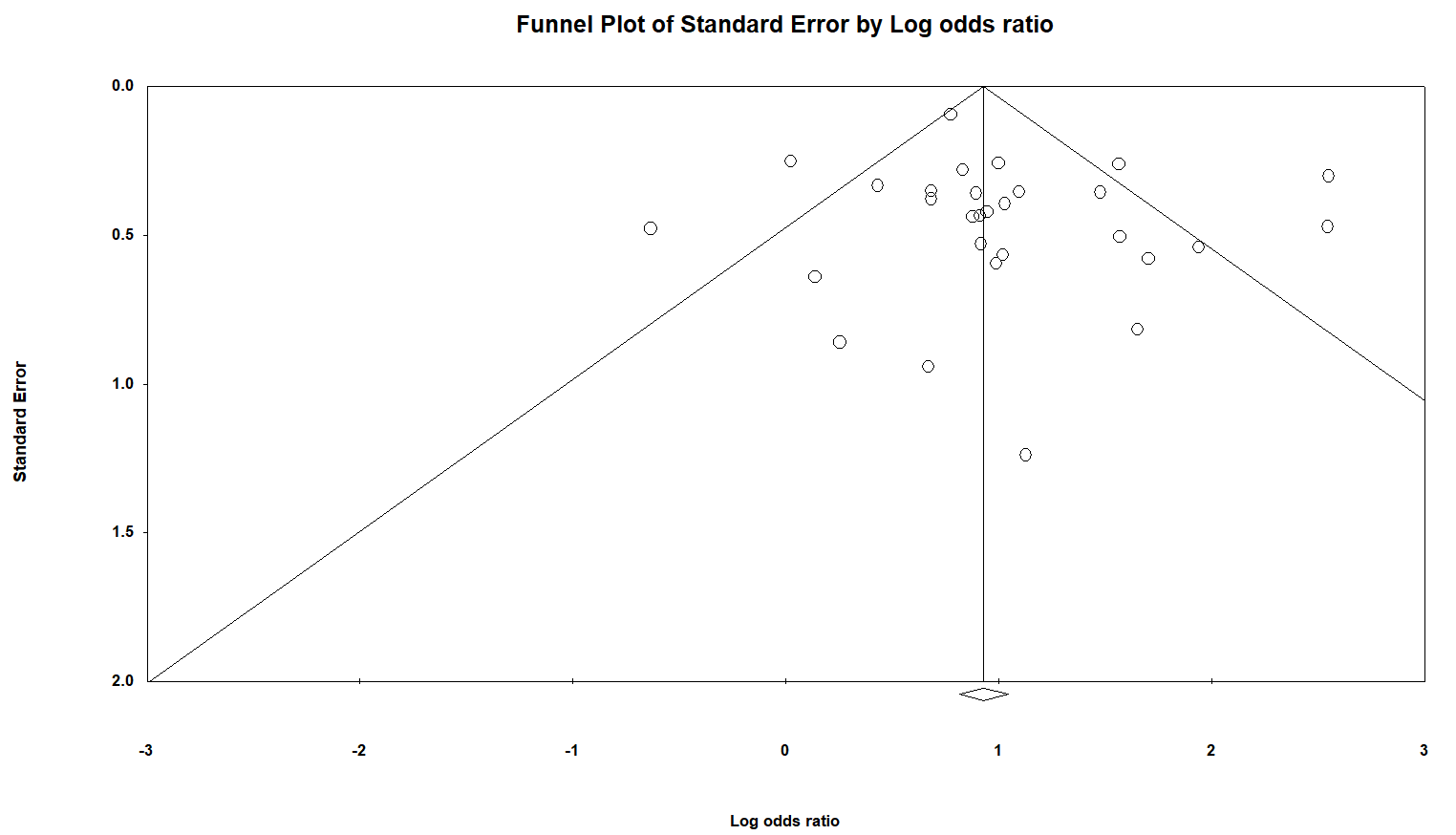 | 0.61 | 0.16 | - | - | - |
| Readmission | Categorical | 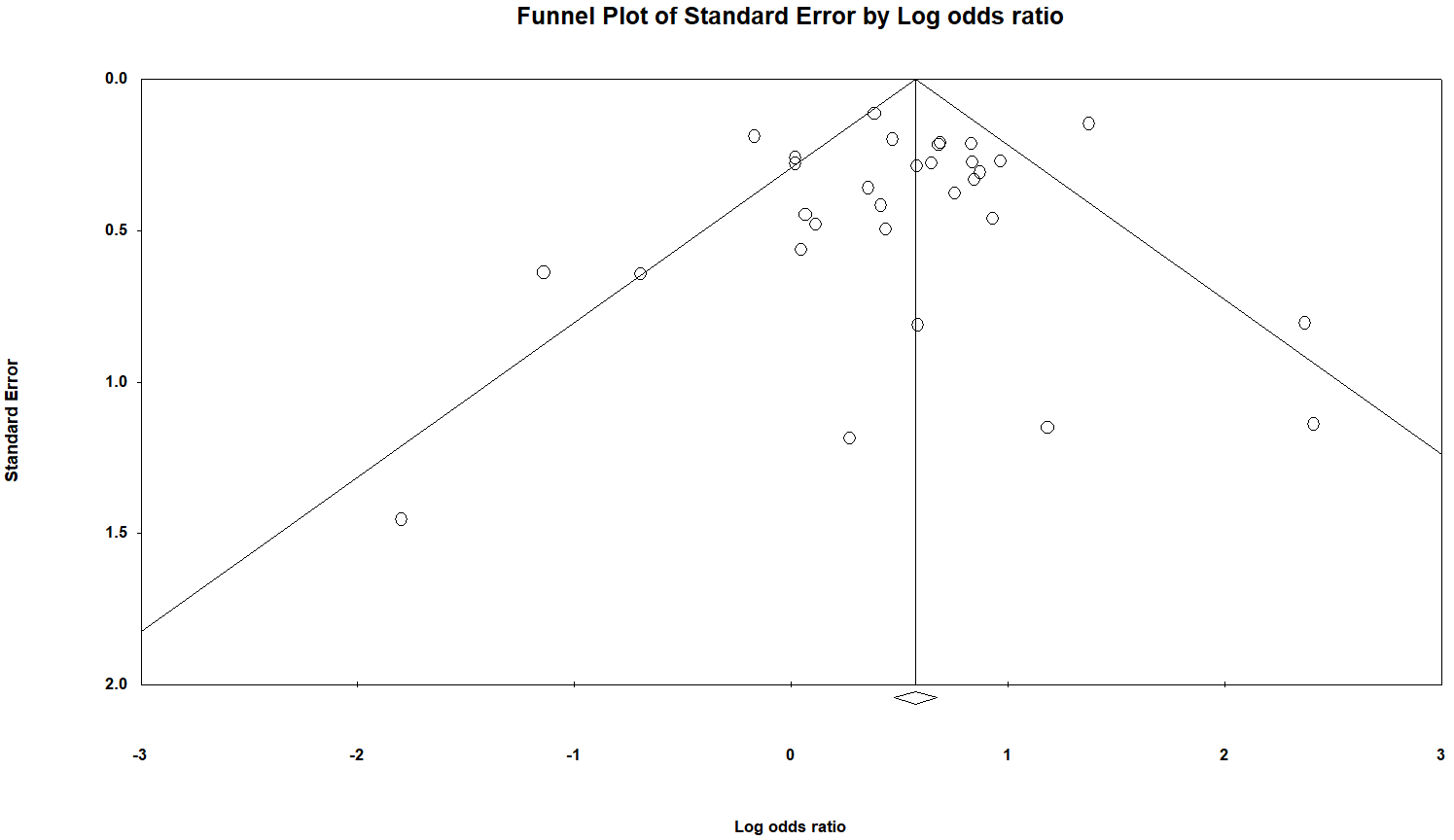 | -0.38 | 0.27 | - | - | - |
| Mortality | Categorical | 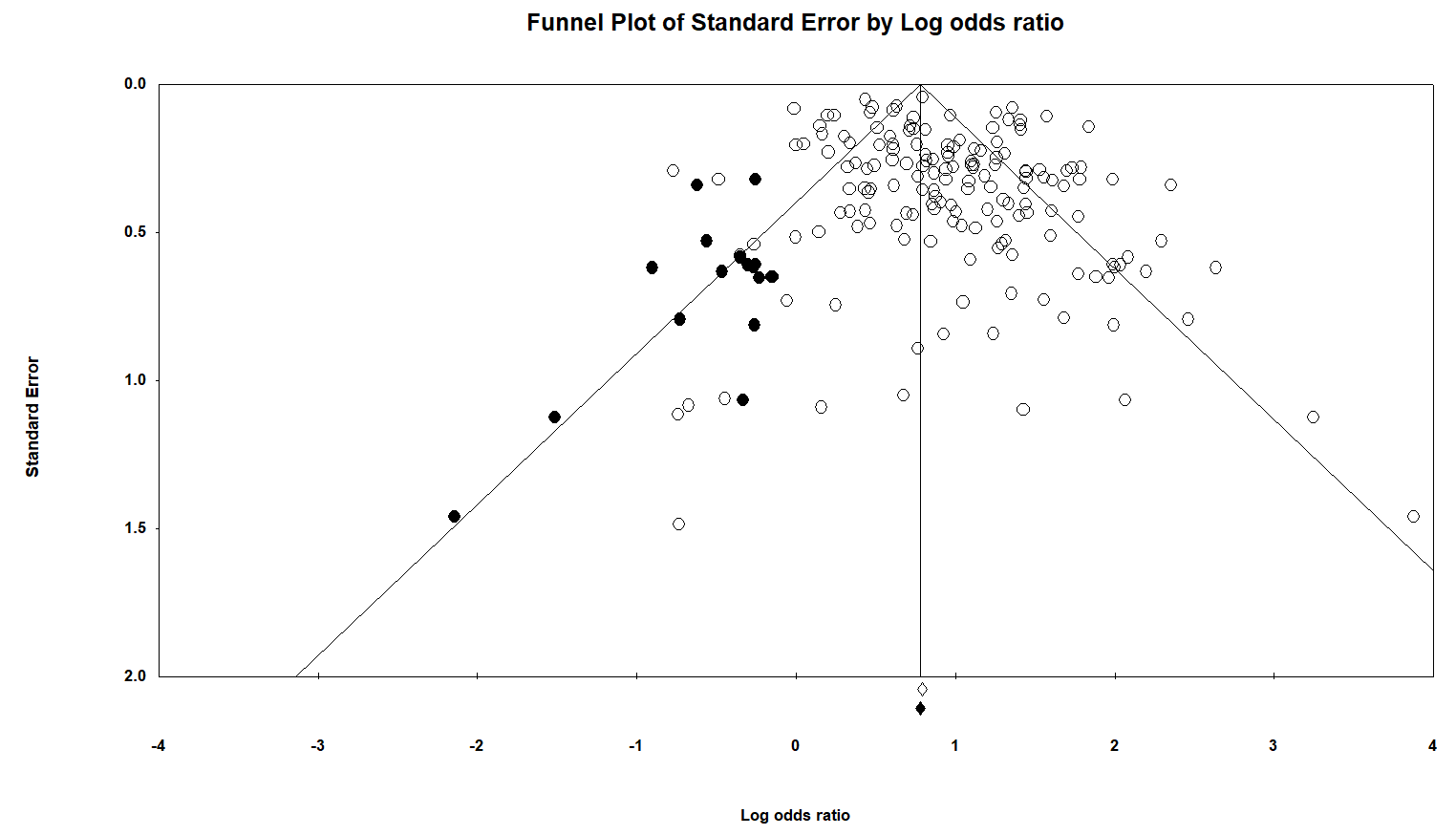 | 0.96 | <0.001 | 16 | 2.38 | 2.19 – 2.59 |

Footnote: Small study investigation conducted when more than 10 studies available

# Table S16. Risk of bias assessment

**Cognition**

| **Author name and year** | **Selection** | **Comparability** | **Outcome** | **Total** | **Overall quality** |
| --- | --- | --- | --- | --- | --- |
| Bickel 2008 | 4 | 2 | 3 | 9 | Good |
| Brown 2018 | 4 | 0 | 3 | 7 | Fair |
| Bruck 2018 | 4 | 2 | 1 | 7 | Fair |
| Bryson 2011 | 4 | 0 | 3 | 7 | Fair |
| Bulic 2020 | 4 | 0 | 2 | 6 | Fair |
| Cavallari 2017 | 4 | 0 | 3 | 7 | Fair |
| Chan 2017 | 3 | 0 | 2 | 5 | Fair |
| Chen 2017 | 3 | 2 | 2 | 7 | Good |
| Cole 2008 | 4 | 2 | 3 | 9 | Good |
| Daiello 2019 | 3 | 0 | 3 | 6 | Fair |
| delaVarga-Martínez 2022 | 4 | 2 | 3 | 9 | Good |
| Ditzel 2023 | 4 | 0 | 2 | 6 | Fair |
| Dostovic 2021 | 4 | 0 | 3 | 7 | Fair |
| Duppils 2004 | 3 | 0 | 3 | 6 | Fair |
| Eide 2016 | 4 | 2 | 2 | 8 | Good |
| Francis 1992 | 4 | 0 | 2 | 6 | Fair |
| Franck 2016 | 4 | 2 | 2 | 8 | Good |
| Giroux 2021 | 4 | 2 | 2 | 8 | Good |
| Gonçalves 2023 | 2 | 0 | 3 | 5 | Fair |
| Hempenius 2016 | 4 | 0 | 2 | 6 | Fair |
| Hoogma 2023 | 4 | 0 | 1 | 5 | Fair |
| Humbert 2021 | 4 | 1 | 3 | 8 | Good |
| Inouye 2016 | 4 | 2 | 2 | 8 | Good |
| Jankowski 2011 | 4 | 2 | 2 | 8 | Good |
| Janssen 2021 | 4 | 2 | 2 | 8 | Good |
| Kainz 2022 | 4 | 0 | 3 | 7 | Fair |
| Kat 2008 | 4 | 0 | 2 | 6 | Fair |
| Katz 2001 | 4 | 0 | 3 | 7 | Fair |
| Koster 2012 | 3 | 1 | 2 | 6 | Good |
| Krogseth 2016 | 4 | 1 | 2 | 7 | Good |
| Krogseth 2023 | 4 | 2 | 2 | 8 | Good |
| Kunicki 2023 | 4 | 2 | 2 | 8 | Good |
| Lingehall 2017 | 4 | 0 | 2 | 6 | Fair |
| van Rijsbergen 2011 | 4 | 0 | 3 | 7 | Fair |
| Mathies 2020 | 4 | 0 | 2 | 6 | Fair |
| McCusker 2001 | 4 | 2 | 3 | 9 | Good |
| McCusker 2014 | 4 | 2 | 3 | 9 | Good |
| Muller 2023 | 4 | 2 | 2 | 8 | Good |
| Nerdal 2022 | 4 | 2 | 3 | 9 | Good |
| Neufeld 2015 | 4 | 0 | 3 | 7 | Fair |
| Nguyen 2018 | 4 | 2 | 2 | 8 | Good |
| Ojagbemi 2020 | 4 | 2 | 3 | 9 | Good |
| Olofsson 2018 | 3 | 0 | 2 | 5 | Fair |
| Pandharipande 2013 | 4 | 2 | 2 | 8 | Good |
| Racine 2020 | 4 | 2 | 3 | 9 | Good |
| Richardson 2021_2 | 4 | 0 | 2 | 6 | Fair |
| Rudolph 2008 | 4 | 2 | 3 | 9 | Good |
| Saczynski 2012 | 4 | 2 | 3 | 9 | Good |
| Sánchez-Lozano 2023 | 3 | 0 | 2 | 5 | Fair |
| Sauer 2017 | 4 | 2 | 3 | 9 | Good |
| Serrano-Duenas 2005 | 4 | 0 | 2 | 6 | Fair |
| Sheng 2006 | 3 | 0 | 2 | 5 | Fair |
| Svenningsen 2014 | 3 | 0 | 2 | 5 | Fair |
| vandenBoogaard 2012 | 3 | 2 | 2 | 7 | Good |
| vanderHeijden 2023 | 4 | 2 | 0 | 6 | Fair |
| Vasunilashorn 2018 | 4 | 2 | 3 | 9 | Good |
| Verloo 2016 | 3 | 0 | 2 | 5 | Fair |
| Vives-Borrás 2019 | 4 | 0 | 3 | 7 | Fair |
| Whittamore 2014 | 4 | 0 | 3 | 7 | Fair |
| Witlox 2013 | 4 | 0 | 2 | 6 | Fair |
| Wolters 2014 | 3 | 2 | 1 | 6 | Fair |
| Wolters 2017 | 3 | 2 | 2 | 7 | Good |

Footnote**:** Newcastle - Ottawa quality assessment scale: Cohort studies

Good quality: 3 or 4 stars in selection domain AND 1 or 2 stars in comparability domain AND 2 or 3 stars in outcome/exposure domain

Fair quality: 2 stars in selection domain AND 1 or 2 stars in comparability domain AND 2 or 3 stars in outcome/exposure domain

Poor quality: 0 or 1 star in selection domain OR 0 stars in comparability domain OR 0 or 1 stars in outcome/exposure domain

**Functional outcomes**

| **Author name and year** | **Selection** | **Comparability** | **Outcome** | **Total** | **Overall quality** |
| --- | --- | --- | --- | --- | --- |
| Abelha 2013 | 4 | 2 | 2 | 8 | Good |
| Alzoubi 2023 | 3 | 2 | 3 | 8 | Good |
| Beishuizen 2020 | 4 | 0 | 3 | 7 | Good |
| Bickel 2008 | 4 | 2 | 3 | 9 | Good |
| Buurman 2011 | 4 | 0 | 3 | 7 | Fair |
| Cartei 2022 | 4 | 2 | 3 | 9 | Good |
| Chan 2017 | 3 | 0 | 2 | 5 | Fair |
| Cirbus 2019 | 4 | 2 | 1 | 7 | Fair |
| Cole 2008 | 4 | 2 | 3 | 9 | Good |
| Czyzycki 2022 | 4 | 2 | 3 | 9 | Good |
| Davis 2012 | 4 | 2 | 2 | 8 | Good |
| Decrane 2012 | 3 | 0 | 3 | 6 | Fair |
| delaVarga-Martínez 2022 | 4 | 2 | 3 | 9 | Good |
| Miu 2013 | 4 | 0 | 3 | 7 | Fair |
| Durlach 2023 | 4 | 0 | 3 | 7 | Fair |
| Edelstein 2004 | 4 | 0 | 3 | 7 | Fair |
| Eeles 2012 | 4 | 0 | 3 | 7 | Fair |
| Eide 2016 | 4 | 2 | 3 | 9 | Good |
| FialhoSilva 2021 | 3 | 0 | 3 | 6 | Fair |
| Fick 2013 | 3 | 0 | 3 | 6 | Fair |
| Francis 1990 | 4 | 0 | 3 | 7 | Good |
| Francis 1992 | 4 | 2 | 3 | 9 | Good |
| Furlaneto 2007 | 4 | 0 | 2 | 6 | Fair |
| Gandossi 2023 | 4 | 1 | 2 | 7 | Fair |
| Giroux 2021 | 4 | 2 | 2 | 8 | Good |
| Givens 2008 | 4 | 2 | 3 | 9 | Good |
| Givens 2009 | 4 | 2 | 3 | 9 | Good |
| Guenther 2020 | 4 | 0 | 2 | 6 | Fair |
| Hawley 2023 | 4 | 2 | 1 | 7 | Fair |
| Hempenius 2016 | 4 | 0 | 3 | 7 | Fair |
| Hoogma 2023 | 4 | 0 | 3 | 7 | Fair |
| Hshieh 2017 | 4 | 2 | 2 | 8 | Good |
| Humbert 2021 | 4 | 1 | 3 | 8 | Good |
| Inouye 1998 | 4 | 2 | 3 | 9 | Good |
| Isaia 2009 | 4 | 0 | 2 | 6 | Fair |
| Jackson 2014 | 4 | 2 | 3 | 9 | Good |
| Jankowski 2011 | 4 | 0 | 2 | 6 | Fair |
| Katz 2001 | 4 | 0 | 3 | 7 | Fair |
| Lee 2011 | 4 | 0 | 3 | 7 | Fair |
| Liang 2014 | 4 | 2 | 2 | 8 | Good |
| McCusker 2001 | 4 | 2 | 3 | 9 | Good |
| McCusker 2014 | 4 | 2 | 3 | 9 | Good |
| Miyamoto 2021 | 4 | 2 | 1 | 7 | Fair |
| Monacelli 2018 | 4 | 0 | 1 | 5 | Fair |
| Morandi 2014 | 4 | 2 | 3 | 9 | Good |
| Murray 1993 | 4 | 2 | 2 | 8 | Good |
| Neufeld 2015 | 4 | 0 | 3 | 7 | Fair |
| Noriega 2015 | 4 | 2 | 2 | 8 | Good |
| Ogawa 2017 | 4 | 2 | 2 | 8 | Good |
| Ojagbemi 2020 | 4 | 2 | 3 | 9 | Good |
| Oldenbeuving 2011 | 3 | 2 | 3 | 8 | Good |
| Pasinska 2019 | 4 | 0 | 3 | 7 | Fair |
| Paulino 2023 | 4 | 0 | 2 | 6 | Fair |
| Qu 2018 | 3 | 2 | 3 | 8 | Good |
| Quinlan 2011 | 4 | 2 | 3 | 9 | Good |
| Racine 2018 | 4 | 2 | 3 | 9 | Good |
| Rawle 2021 | 4 | 0 | 2 | 6 | Fair |
| Rollo 2022 | 4 | 2 | 3 | 9 | Good |
| Rudolph 2010 | 4 | 2 | 3 | 9 | Good |
| Sánchez-Lozano 2023 | 3 | 0 | 2 | 5 | Fair |
| Sheng 2006 | 4 | 0 | 3 | 7 | Fair |
| Shi 2019 | 4 | 2 | 2 | 8 | Good |
| Shi 2019_2 | 4 | 0 | 2 | 6 | Fair |
| Shim 2015 | 4 | 0 | 1 | 5 | Fair |
| Singler 2014 | 4 | 0 | 3 | 7 | Fair |
| Suraarunsumrit 2022 | 4 | 2 | 3 | 9 | Good |
| Svenningsen 2014 | 3 | 0 | 3 | 6 | Fair |
| Tavares 2021 | 4 | 0 | 3 | 7 | Fair |
| To-adithep 2023 | 3 | 2 | 3 | 8 | Good |
| vandenBoogaard 2012 | 3 | 2 | 2 | 7 | Good |
| vanderHeijden 2023 | 4 | 2 | 0 | 6 | Fair |
| Verloo 2016 | 3 | 0 | 2 | 5 | Fair |
| Vida 2006 | 4 | 2 | 3 | 9 | Good |
| Vives-Borrás 2019 | 4 | 0 | 3 | 7 | Fair |
| Wang 2021 | 4 | 0 | 2 | 6 | Fair |
| Weng 2019 | 4 | 2 | 2 | 8 | Good |
| Whittamore 2014 | 4 | 0 | 3 | 7 | Fair |
| Witlox 2013 | 4 | 0 | 2 | 6 | Fair |
| Zakriya 2004 | 3 | 0 | 3 | 6 | Fair |
| Zipprich 2020 | 3 | 0 | 2 | 5 | Fair |

Footnote**:** Newcastle - Ottawa quality assessment scale: Cohort studies

Good quality: 3 or 4 stars in selection domain AND 1 or 2 stars in comparability domain AND 2 or 3 stars in outcome/exposure domain

Fair quality: 2 stars in selection domain AND 1 or 2 stars in comparability domain AND 2 or 3 stars in outcome/exposure domain

Poor quality: 0 or 1 star in selection domain OR 0 stars in comparability domain OR 0 or 1 stars in outcome/exposure domain

**Quality of life**

| **Author name and year** | **Selection** | **Comparability** | **Outcome** | **Total** | **Overall quality** |
| --- | --- | --- | --- | --- | --- |
| Bagienski 2017 | 4 | 0 | 1 | 5 | Fair |
| Chen 2017 | 3 | 2 | 2 | 7 | Good |
| Duppils 2004 | 3 | 0 | 3 | 6 | Fair |
| Eide 2016 | 4 | 0 | 1 | 5 | Fair |
| Hofhuis 2022 | 3 | 2 | 1 | 6 | Fair |
| Hempenius 2016 | 4 | 0 | 3 | 7 | Fair |
| Hoogma 2023 | 4 | 0 | 1 | 5 | Fair |
| Jackson 2014 | 4 | 2 | 3 | 9 | Fair |
| Janssen 2021 | 4 | 0 | 3 | 7 | Fair |
| Kainz 2022 | 3 | 0 | 3 | 6 | Fair |
| Koster 2012 | 3 | 0 | 2 | 5 | Fair |
| Labaste 2020 | 3 | 0 | 3 | 6 | Fair |
| Luz 2020 | 3 | 0 | 2 | 5 | Fair |
| Minden 2005 | 4 | 2 | 2 | 8 | Good |
| Naidech 2012 | 3 | 2 | 1 | 6 | Fair |
| Rosenthal 2017 | 3 | 2 | 1 | 6 | Fair |
| Sánchez-Lozano 2023 | 3 | 0 | 2 | 5 | Fair |
| Suraarunsumrit 2022 | 4 | 0 | 3 | 7 | Fair |
| Svenningsen 2014 | 3 | 0 | 2 | 5 | Fair |
| vandenBoogaard 2012 | 3 | 2 | 2 | 7 | Good |
| vanderHeijden 2023 | 4 | 0 | 0 | 4 | Poor |
| VanRompaey 2009 | 3 | 0 | 2 | 5 | Fair |
| Wolters 2014 | 3 | 2 | 1 | 6 | Fair |
| Zipprich 2020 | 3 | 0 | 2 | 5 | Fair |

Footnote**:** Newcastle - Ottawa quality assessment scale: Cohort studies

Good quality: 3 or 4 stars in selection domain AND 1 or 2 stars in comparability domain AND 2 or 3 stars in outcome/exposure domain

Fair quality: 2 stars in selection domain AND 1 or 2 stars in comparability domain AND 2 or 3 stars in outcome/exposure domain

Poor quality: 0 or 1 star in selection domain OR 0 stars in comparability domain OR 0 or 1 stars in outcome/exposure domain

**Mental health**

| **Author name and year** | **Selection** | **Comparability** | **Outcome** | **Total** | **Overall quality** |
| --- | --- | --- | --- | --- | --- |
| Brown 2020 | 3 | 2 | 2 | 7 | Good |
| Bulic 2020 | 3 | 2 | 2 | 7 | Good |
| Chan 2017 | 3 | 0 | 2 | 5 | Fair |
| delaVarga-Martínez 2022 | 4 | 2 | 3 | 9 | Good |
| Drews 2015 | 3 | 0 | 1 | 4 | Fair |
| Jackson 2014 | 4 | 2 | 3 | 9 | Good |
| Janssen 2021 | 4 | 2 | 3 | 9 | Good |
| Kat 2008 | 4 | 0 | 2 | 6 | Fair |
| Koster 2012 | 3 | 1 | 2 | 6 | Good |
| Miyamoto 2021 | 4 | 2 | 1 | 7 | Fair |
| Nerdal 2022 | 3 | 2 | 3 | 8 | Good |
| Nguyen 2018 | 4 | 2 | 2 | 8 | Good |
| Olofsson 2018 | 4 | 0 | 2 | 6 | Fair |
| Slor 2013 | 4 | 0 | 3 | 7 | Fair |
| Svenningsen 2015 | 3 | 0 | 3 | 6 | Fair |
| vanderHeijden 2023 | 4 | 2 | 0 | 6 | Fair |
| Verloo 2016 | 3 | 0 | 2 | 5 | Fair |
| Whittamore 2014 | 4 | 0 | 3 | 7 | Fair |
| Witlox 2013 | 4 | 0 | 2 | 6 | Fair |

Footnote**:** Newcastle - Ottawa quality assessment scale: Cohort studies

Good quality: 3 or 4 stars in selection domain AND 1 or 2 stars in comparability domain AND 2 or 3 stars in outcome/exposure domain

Fair quality: 2 stars in selection domain AND 1 or 2 stars in comparability domain AND 2 or 3 stars in outcome/exposure domain

Poor quality: 0 or 1 star in selection domain OR 0 stars in comparability domain OR 0 or 1 stars in outcome/exposure domain

**Dementia**

| **Author name and year** | **Selection** | **Comparability** | **Outcome** | **Total** | **Overall quality** |
| --- | --- | --- | --- | --- | --- |
| Bickel 2008 | 4 | 2 | 3 | 9 | Good |
| Brown 2020 | 3 | 2 | 2 | 7 | Good |
| Bulic 2020 | 4 | 0 | 2 | 6 | Fair |
| Davis 2012 | 4 | 2 | 2 | 8 | Good |
| Davis 2014 | 4 | 2 | 3 | 9 | Good |
| Dostovic 2021 | 4 | 0 | 3 | 7 | Fair |
| Dros 2020 | 4 | 2 | 3 | 9 | Good |
| Garcez 2019 | 4 | 2 | 3 | 9 | Good |
| Kat 2008 | 4 | 0 | 2 | 6 | Fair |
| Krogseth 2011 | 4 | 2 | 2 | 8 | Good |
| Lingehall 2017 | 4 | 2 | 2 | 8 | Good |
| Lundström 2003 | 4 | 2 | 2 | 8 | Good |
| van Rijsbergen 2011 | 4 | 2 | 3 | 9 | Good |
| Marrama 2022 | 4 | 2 | 3 | 9 | Good |
| Neerland 2017 | 4 | 2 | 2 | 8 | Good |
| Nerdal 2022 | 4 | 0 | 3 | 7 | Fair |
| Ojagbemi 2020 | 4 | 2 | 3 | 9 | Good |
| Olofsson 2018 | 3 | 2 | 2 | 7 | Good |
| Rahkonen 2001 | 4 | 0 | 2 | 6 | Fair |
| Rawle 2021 | 4 | 0 | 2 | 6 | Fair |
| Richardson 2021 | 4 | 2 | 2 | 8 | Good |
| Rockwood 1999 | 4 | 2 | 2 | 8 | Good |
| Rolandi 2020 | 4 | 2 | 3 | 9 | Good |

Footnote**:** Newcastle - Ottawa quality assessment scale: Cohort studies

Good quality: 3 or 4 stars in selection domain AND 1 or 2 stars in comparability domain AND 2 or 3 stars in outcome/exposure domain

Fair quality: 2 stars in selection domain AND 1 or 2 stars in comparability domain AND 2 or 3 stars in outcome/exposure domain

Poor quality: 0 or 1 star in selection domain OR 0 stars in comparability domain OR 0 or 1 stars in outcome/exposure domain

**Institutionalisation**

| **Author name and year** | **Selection** | **Comparability** | **Outcome** | **Total** | **Overall quality** |
| --- | --- | --- | --- | --- | --- |
| Bellelli 2008 | 4 | 2 | 3 | 9 | Good |
| Cole 2008 | 4 | 2 | 3 | 9 | Good |
| deJong 2019 | 4 | 0 | 2 | 6 | Fair |
| Miu 2013 | 4 | 0 | 3 | 7 | Fair |
| Durlach 2023 | 4 | 0 | 3 | 7 | Fair |
| Eeles 2010 | 4 | 2 | 3 | 9 | Good |
| Francis 1990 | 4 | 0 | 3 | 7 | Good |
| George 1997 | 4 | 0 | 2 | 6 | Fair |
| Givens 2009 | 4 | 2 | 3 | 9 | Good |
| Hawley 2023 | 4 | 2 | 1 | 7 | Fair |
| Hölttä 2011 | 4 | 2 | 3 | 9 | Good |
| Inouye 1998 | 4 | 2 | 3 | 9 | Good |
| Kat 2008 | 4 | 0 | 2 | 6 | Fair |
| Krogseth 2014 | 4 | 2 | 3 | 9 | Good |
| Labaste 2023 | 4 | 2 | 3 | 9 | Good |
| MacLullich 2019 | 3 | 0 | 2 | 5 | Fair |
| Mariz 2013 | 4 | 0 | 3 | 7 | Fair |
| McAvay 2006 | 3 | 0 | 2 | 5 | Fair |
| McCusker 2001 | 4 | 2 | 2 | 8 | Good |
| Morandi 2014 | 4 | 2 | 2 | 8 | Fair |
| Neufeld 2015 | 4 | 0 | 3 | 7 | Fair |
| O'Keeffe 1997 | 4 | 2 | 2 | 8 | Good |
| Paulino 2023 | 3 | 0 | 2 | 5 | Fair |
| Pitkala 2005 | 4 | 2 | 2 | 8 | Good |
| Racine 2018 | 3 | 2 | 3 | 8 | Good |
| Rawle 2021 | 4 | 0 | 2 | 6 | Fair |
| Sheng 2006 | 3 | 0 | 2 | 5 | Fair |
| Visser 2015 | 4 | 0 | 1 | 5 | Fair |
| Zakriya 2004 | 3 | 0 | 3 | 6 | Fair |

Footnote**:** Newcastle - Ottawa quality assessment scale: Cohort studies

Good quality: 3 or 4 stars in selection domain AND 1 or 2 stars in comparability domain AND 2 or 3 stars in outcome/exposure domain

Fair quality: 2 stars in selection domain AND 1 or 2 stars in comparability domain AND 2 or 3 stars in outcome/exposure domain

Poor quality: 0 or 1 star in selection domain OR 0 stars in comparability domain OR 0 or 1 stars in outcome/exposure domain

**Readmission**

| **Author name and year** | **Selection** | **Comparability** | **Outcome** | **Total** | **Overall quality** |
| --- | --- | --- | --- | --- | --- |
| Bellelli 2008 | 4 | 2 | 3 | 9 | Good |
| Durlach 2023 | 4 | 0 | 3 | 7 | Fair |
| Eide 2016_1 | 4 | 0 | 1 | 5 | Fair |
| Falsini 2018 | 4 | 0 | 3 | 7 | Fair |
| George 1997 | 4 | 0 | 2 | 6 | Fair |
| Gleason 2015 | 4 | 2 | 3 | 9 | Good |
| Gual 2018 | 4 | 0 | 2 | 6 | Fair |
| Han 2022 | 4 | 2 | 3 | 9 | Good |
| Hempenius 2016 | 4 | 0 | 3 | 7 | Fair |
| Hoogma 2023 | 4 | 0 | 2 | 6 | Fair |
| Kennedy 2014 | 4 | 0 | 3 | 7 | Fair |
| Koster 2012 | 3 | 0 | 2 | 5 | Fair |
| Moon 2018 | 4 | 2 | 2 | 8 | Good |
| Large 2013 | 4 | 0 | 2 | 6 | Fair |
| Li 2019 | 4 | 0 | 1 | 5 | Fair |
| Marcantonio 2005 | 4 | 2 | 2 | 8 | Good |
| Muzzana 2022 | 4 | 0 | 2 | 6 | Fair |
| Neufeld 2015 | 4 | 0 | 3 | 7 | Fair |
| Noriega 2015 | 4 | 2 | 2 | 8 | Good |
| Paulino 2023 | 4 | 0 | 2 | 6 | Fair |
| Pendlebury 2015 | 4 | 0 | 2 | 6 | Fair |
| Penfold 2023 | 4 | 2 | 2 | 8 | Good |
| Racine 2018 | 4 | 2 | 3 | 9 | Good |
| Sanguanwit 2023 | 4 | 2 | 2 | 8 | Good |
| Singler 2014 | 4 | 0 | 3 | 7 | Fair |
| Suraarunsumrit 2022 | 4 | 0 | 3 | 7 | Fair |
| Traissac 2011 | 4 | 2 | 2 | 8 | Good |
| Uthamalingam 2011 | 4 | 2 | 2 | 8 | Good |
| Vasunilashorn 2022 | 4 | 2 | 2 | 8 | Good |
| Whittamore 2014 | 4 | 0 | 3 | 7 | Fair |
| Zakriya 2004 | 3 | 0 | 3 | 6 | Fair |

Footnote**:** Newcastle - Ottawa quality assessment scale: Cohort studies

Good quality: 3 or 4 stars in selection domain AND 1 or 2 stars in comparability domain AND 2 or 3 stars in outcome/exposure domain

Fair quality: 2 stars in selection domain AND 1 or 2 stars in comparability domain AND 2 or 3 stars in outcome/exposure domain

Poor quality: 0 or 1 star in selection domain OR 0 stars in comparability domain OR 0 or 1 stars in outcome/exposure domain

**Mortality**

| **Author name and year** | **Selection** | **Comparability** | **Outcome** | **Total** | **Overall quality** |
| --- | --- | --- | --- | --- | --- |
| Abelha 2013 | 4 | 2 | 3 | 9 | Good |
| Adamis 2007 | 4 | 0 | 3 | 7 | Fair |
| Alberto 2018 | 4 | 2 | 3 | 9 | Good |
| AlHuraizi 2023 | 4 | 0 | 3 | 7 | Fair |
| Alzoubi 2023 | 4 | 2 | 3 | 9 | Good |
| Arneson 2023 | 4 | 2 | 3 | 9 | Good |
| Avelino-Silva 2017 | 4 | 2 | 3 | 9 | Good |
| Avelino-Silva 2018 | 4 | 2 | 3 | 9 | Good |
| Bagienski 2017 | 4 | 2 | 1 | 7 | Good |
| Bakker 2012 | 4 | 0 | 3 | 7 | Fair |
| Beishuizen 2020 | 4 | 0 | 3 | 7 | Fair |
| Bellelli 2007 | 4 | 2 | 2 | 8 | Good |
| Bellelli 2008 | 4 | 0 | 3 | 7 | Good |
| Bellelli 2018 | 4 | 2 | 3 | 9 | Good |
| Bickel 2008 | 4 | 2 | 3 | 9 | Good |
| Bulic 2020 | 4 | 0 | 2 | 6 | Fair |
| Cartei 2022 | 4 | 0 | 3 | 7 | Fair |
| Chalmers 2021 | 4 | 2 | 3 | 9 | Good |
| Chan 2016 | 4 | 2 | 3 | 9 | Good |
| Chan 2017 | 4 | 0 | 2 | 6 | Fair |
| Cheong 2021 | 4 | 0 | 3 | 7 | Fair |
| Cole 2008 | 4 | 2 | 3 | 9 | Good |
| Curyto 2001 | 4 | 2 | 2 | 8 | Good |
| Czyzycki 2022 | 4 | 2 | 2 | 8 | Good |
| Dani 2017 | 4 | 2 | 3 | 9 | Good |
| Davis 2014 | 4 | 2 | 3 | 9 | Good |
| DeCrane 2011 | 4 | 2 | 3 | 9 | Good |
| deHaan 2023 | 4 | 0 | 3 | 7 | Fair |
| deJong 2019 | 4 | 2 | 2 | 8 | Good |
| Ditzel 2023 | 4 | 0 | 1 | 5 | Fair |
| Diwell 2018 | 4 | 2 | 3 | 9 | Good |
| Miu 2013 | 4 | 2 | 3 | 9 | Good |
| Duprey 2020 | 4 | 2 | 2 | 8 | Good |
| Durlach 2023 | 4 | 0 | 3 | 7 | Fair |
| Edelstein 2004 | 4 | 0 | 3 | 7 | Fair |
| Edlund 2006 | 4 | 0 | 3 | 7 | Fair |
| van Eijsden 2015 | 4 | 0 | 1 | 5 | Fair |
| Elsayem 2017 | 4 | 1 | 3 | 8 | Good |
| Evensen 2021 | 4 | 2 | 3 | 9 | Good |
| Falsini 2018 | 4 | 2 | 3 | 9 | Good |
| FialhoSilva 2021 | 4 | 0 | 3 | 7 | Fair |
| Fick 2013 | 4 | 2 | 3 | 9 | Good |
| Francis 1990 | 4 | 0 | 3 | 7 | Good |
| Francis 1992 | 4 | 0 | 3 | 7 | Fair |
| Furlaneto 2007 | 4 | 2 | 3 | 9 | Good |
| George 1997 | 4 | 0 | 2 | 6 | Fair |
| Givens 2009 | 4 | 2 | 3 | 9 | Good |
| Gonzalez 2005 | 4 | 0 | 3 | 7 | Fair |
| Gonzalez 2009 | 4 | 2 | 2 | 8 | Good |
| Gottschalk 2015 | 4 | 0 | 3 | 7 | Fair |
| Gou 2021 | 4 | 0 | 2 | 6 | Fair |
| Goudzwaard 2020 | 4 | 2 | 3 | 9 | Good |
| Han 2010 | 4 | 3 | 2 | 9 | Good |
| Han 2017 | 4 | 2 | 2 | 8 | Good |
| Han 2022 | 4 | 2 | 3 | 9 | Good |
| Hapca 2018 | 4 | 2 | 2 | 8 | Good |
| Hempenius 2016 | 4 | 0 | 3 | 7 | Fair |
| Hölttä 2011 | 4 | 2 | 3 | 9 | Good |
| Honda 2018 | 4 | 2 | 2 | 8 | Good |
| Hoogma 2023 | 4 | 0 | 1 | 5 | Fair |
| Hshieh 2023 | 4 | 2 | 2 | 8 | Good |
| Hughes 2021 | 4 | 2 | 2 | 8 | Good |
| Inouye 2016 | 4 | 2 | 2 | 8 | Good |
| Isaia 2009 | 4 | 0 | 2 | 6 | Fair |
| Iwata 2020 | 4 | 1 | 1 | 6 | Fair |
| Juliebø 2010 | 4 | 0 | 3 | 7 | Fair |
| Kakuma 2003 | 4 | 2 | 3 | 9 | Good |
| Kat 2008 | 4 | 0 | 2 | 6 | Fair |
| Kat 2011 | 4 | 0 | 3 | 7 | Fair |
| Kennedy 2014 | 4 | 0 | 3 | 7 | Fair |
| Kilicaslan 2022 | 4 | 0 | 3 | 7 | Fair |
| Knauf 2019 | 4 | 2 | 3 | 9 | Good |
| Korber 2021 | 4 | 2 | 3 | 9 | Good |
| Koster 2012 | 3 | 1 | 2 | 6 | Good |
| Kotfis 2019 | 4 | 0 | 2 | 6 | Fair |
| Krzych 2014 | 4 | 2 | 1 | 7 | Fair |
| Kunicki 2023 | 4 | 2 | 2 | 8 | Good |
| Moon 2018 | 4 | 2 | 2 | 8 | Good |
| Labaste 2020 | 3 | 0 | 3 | 6 | Fair |
| Large 2013 | 4 | 0 | 2 | 6 | Fair |
| Lee 2011 | 4 | 0 | 3 | 7 | Fair |
| Lee 2017 | 4 | 2 | 2 | 8 | Good |
| Lee 2018 | 4 | 0 | 3 | 7 | Fair |
| Leslie 2005 | 4 | 2 | 2 | 8 | Good |
| Leslie 2008 | 4 | 0 | 2 | 6 | Fair |
| Lewis 1995 | 4 | 0 | 2 | 6 | Fair |
| Li 2019 | 4 | 0 | 1 | 5 | Fair |
| Li 2021 | 4 | 2 | 3 | 9 | Good |
| Lima 2010 | 4 | 2 | 2 | 8 | Good |
| Lundström 2003 | 4 | 0 | 2 | 6 | Fair |
| Luz 2020 | 3 | 0 | 2 | 5 | Fair |
| MacLullich 2019 | 3 | 0 | 2 | 5 | Fair |
| Mariz 2013 | 4 | 0 | 3 | 7 | Fair |
| van Rijsbergen 2011 | 4 | 2 | 3 | 9 | Good |
| Marrama 2022 | 4 | 2 | 3 | 9 | Good |
| Mauri 2021 | 4 | 2 | 1 | 7 | Fair |
| Mazzola 2015 | 4 | 2 | 2 | 8 | Good |
| McAvay 2006 | 3 | 0 | 2 | 5 | Fair |
| McCusker 2001 | 4 | 2 | 3 | 9 | Good |
| McCusker 2002 | 4 | 2 | 2 | 8 | Good |
| McCusker 2014 | 4 | 2 | 3 | 9 | Good |
| Minden 2005 | 4 | 2 | 2 | 8 | Good |
| Monacelli 2018 | 4 | 0 | 1 | 5 | Fair |
| Morandi 2014 | 4 | 2 | 3 | 9 | Good |
| Moreno-Gavino 2012 | 4 | 0 | 1 | 5 | Fair |
| Moskowitz 2017 | 4 | 2 | 3 | 9 | Good |
| Muresan 2016 | 4 | 0 | 2 | 6 | Fair |
| Nishizawa 2023 | 4 | 2 | 3 | 9 | Good |
| Noriega 2015 | 4 | 2 | 2 | 8 | Good |
| Ojagbemi 2021 | 4 | 2 | 2 | 8 | Good |
| O'Keeffe 1997 | 4 | 2 | 2 | 8 | Good |
| Olofsson 2018 | 3 | 0 | 2 | 5 | Fair |
| Pak 2020 | 4 | 2 | 1 | 7 | Fair |
| Pasinska 2019 | 4 | 2 | 3 | 9 | Good |
| Patel 2014 | 4 | 0 | 3 | 7 | Fair |
| Paulino 2023 | 4 | 0 | 2 | 6 | Fair |
| Penfold 2023 | 4 | 2 | 2 | 8 | Good |
| Pitkala 2005 | 4 | 2 | 2 | 8 | Good |
| Pompei 1994 | 4 | 2 | 2 | 8 | Good |
| Praditsuwan 2013 | 4 | 2 | 1 | 7 | Fair |
| Qu 2018 | 3 | 2 | 3 | 8 | Good |
| Raats 2015 | 4 | 0 | 3 | 7 | Fair |
| Radcliffe 2023 | 4 | 2 | 3 | 9 | Good |
| Radinovic 2014 | 4 | 0 | 3 | 7 | Fair |
| Rahkonen 2001 | 4 | 0 | 2 | 6 | Fair |
| Rego 2023 | 4 | 2 | 2 | 8 | Good |
| Reynish 2017 | 4 | 0 | 2 | 6 | Fair |
| Rizzi 2015 | 4 | 2 | 2 | 8 | Good |
| Robinson 2009 | 4 | 0 | 3 | 7 | Fair |
| Robinson 2011 | 4 | 0 | 3 | 7 | Fair |
| Rockwood 1999 | 4 | 2 | 2 | 8 | Good |
| Rollo 2022 | 4 | 2 | 3 | 9 | Good |
| Ruggiero 2017 | 4 | 0 | 3 | 7 | Good |
| Sánchez-Lozano 2023 | 3 | 0 | 2 | 5 | Fair |
| Sanguanwit 2023 | 4 | 2 | 2 | 8 | Good |
| Sasajima 2012 | 4 | 0 | 1 | 5 | Fair |
| Sato 2017 | 4 | 2 | 2 | 8 | Good |
| Sauer 2017 | 4 | 2 | 3 | 9 | Good |
| Serrano-Duenas 2005 | 4 | 0 | 2 | 6 | Fair |
| Sheng 2006 | 3 | 0 | 2 | 5 | Fair |
| Shi 2019 | 4 | 2 | 2 | 8 | Good |
| Shintani 2009 | 4 | 2 | 2 | 8 | Good |
| Sri-on 2016 | 4 | 0 | 3 | 7 | Fair |
| Suraarunsumrit 2022 | 4 | 2 | 3 | 9 | Good |
| Tahir 2018 | 4 | 0 | 3 | 7 | Fair |
| Tan AH 2015 | 4 | 0 | 3 | 7 | Fair |
| To-adithep 2023 | 4 | 2 | 3 | 9 | Good |
| Trevisan 2023 | 4 | 2 | 3 | 9 | Good |
| Tripathy 2014 | 4 | 2 | 3 | 9 | Good |
| Tsai 2012 | 4 | 0 | 3 | 7 | Fair |
| Uthamalingam 2011 | 4 | 2 | 2 | 8 | Good |
| VanDerWulp 2019 | 4 | 2 | 2 | 8 | Good |
| van Eijsden 2015 | 4 | 0 | 1 | 5 | Fair |
| VanRompaey 2009 | 3 | 0 | 2 | 5 | Fair |
| Vasunilashorn 2016 | 4 | 0 | 2 | 6 | Fair |
| Vasunilashorn 2022 | 4 | 2 | 2 | 8 | Good |
| Veiga 2012 | 4 | 2 | 2 | 8 | Good |
| Visser 2015 | 4 | 0 | 1 | 5 | Fair |
| Vives-Borrás 2019 | 4 | 0 | 3 | 7 | Fair |
| Wang 2021 | 4 | 2 | 2 | 8 | Fair |
| Whittamore 2014 | 4 | 0 | 3 | 7 | Fair |
| Wolters 2014 | 3 | 2 | 2 | 7 | Good |
| Zakriya 2004 | 3 | 0 | 3 | 6 | Fair |
| Ziman 2020 | 4 | 2 | 3 | 9 | Good |
| Zipprich 2020 | 3 | 0 | 2 | 5 | Fair |

Footnote**:** Newcastle - Ottawa quality assessment scale: Cohort studies

Good quality: 3 or 4 stars in selection domain AND 1 or 2 stars in comparability domain AND 2 or 3 stars in outcome/exposure domain

Fair quality: 2 stars in selection domain AND 1 or 2 stars in comparability domain AND 2 or 3 stars in outcome/exposure domain

Poor quality: 0 or 1 star in selection domain OR 0 stars in comparability domain OR 0 or 1 stars in outcome/exposure domain

Risk of bias

The overall risk of bias across outcomes ranged from low to moderate. Most outcomes, except for QoL and mental health, demonstrated a low risk of bias. However, confounding bias was evident, as many of the studies failed to adequately control confounding variables during study design and analysis. Attrition bias was also noted due to participant loss during follow-up. Additionally, some studies lacked clarity on how the outcomes were assessed and others selectively reported some of the outcomes. Selection bias was generally low across the studies. No studies were excluded from the analysis based on quality. Risk of bias assessment for individual studies presented in Table S16.

# Table S17. GRADE (Grading of Recommendations, Assessment, Development, and Evaluations)

Summary of findings

All included outcomes began the GRADE process with a low certainty of evidence due to reliance on data from observational studies. We identified no serious quality concerns related to imprecision, indirectness, or publication bias. However, most outcomes had a serious risk of bias and inconsistency. For this reason, we have moderate confidence in the effect estimates: the true effect is likely to be close to the estimate of the effect, but there is a possibility that it is substantially different.

| **Outcomes** | **Measure** | **Outcome type** | Data type | **Relative effect**  **(95% CI)** | **No of participants**  **(number of studies)** | **Certainty of the evidence**  **(GRADE)** | **comments** |
| --- | --- | --- | --- | --- | --- | --- | --- |
| **Cognition** | Objective | Change | Categorical | 1.58  (1.22–2.02) | 2788  (7 studies) | Moderate | The certainty of evidence is moderate regarding cognitive outcomes among individuals who experienced delirium compared to those who did not. |
|  |  |  | Continuous | -0.19  (-0.29– -0.09) | 2706  (13 studies) |  |  |
|  | Objective | Performance | Categorical | 2.44  (1.58– -3.88) | 2905  (10 studies) |  |  |
|  |  |  | Continuous | -0.69  (-0.82– -0.56) | 6601  (36 studies) |  |  |
|  | Subjective | Performance | Categorical | 2.11  (1.03– -4.31) | 3489  (5 studies) |  |  |
|  |  |  | Continuous | -0.19  (-0.34– -0.05) | 1997  (7 studies) |  |  |
| **Functional outcome** |  | Change | Categorical | 2.04  (1.74– 2.38) | 6840  (23 studies) | 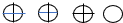  Moderate | The certainty of evidence is moderate regarding functional outcomes among individuals who experienced delirium compared to those who did not. |
|  |  |  | Continuous | -0.43  (-0.69– -0.17) | 1362  (10 studies) |  |  |
|  |  | Performance | Categorical | 2.19  (1.69–2.84) | 26420  (27 studies) |  |  |
|  |  |  | Continuous | -0.62  (-0.83– -0.41) | 9129  (37 studies) |  |  |
| **Quality of life** |  | | Categorical | 2.13  (1.70 –2.68) | 3170  (4 studies) | 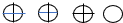  Moderate | The certainty of evidence is moderate regarding quality-of-life outcomes among individuals who experienced delirium compared to those who did not. |
|  |  |  | Continuous | -0.44  (-0.56– -0.32) | 7670  (22 studies) | 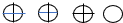  Moderate |  |
| **Mental health** |  | | Categorical | 1.69  (1.31– 2.17 | 8897  (14 studies) | 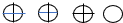  Moderate | The certainty of evidence is moderate regarding mental health outcomes among individuals who experienced delirium compared to those who did not. |
|  |  |  | Continuous | -0.33  (-0.49– -0.17) | 1387  (11 studies) | 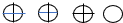  Moderate |  |
| **Dementia** |  | | Categorical | 5.37  (3.31–8.73) | 10660  (23 studies) | 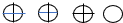  Moderate | The certainty of evidence is moderate regarding dementia outcomes among individuals who experienced delirium compared to those who did not. |
| **Institutionalisation** |  | | Categorical | 2.79  (2.18– 3.56) | 27094  (29 studies) | 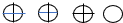  Moderate | The certainty of evidence is moderate regarding Institutionalisation outcomes among individuals who experienced delirium compared to those who did not. |
| **Readmission** |  | | Categorical | 1.70  (1.39– 2.07) | 11087  (31 studies) | 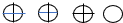  Moderate | The certainty of evidence is moderate regarding readmission outcomes among individuals who experienced delirium compared to those who did not. |
| **Mortality** |  | | Categorical | 2.53  (2.32–2.76) | 86173  (165 studies) | 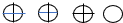  Moderate | The certainty of evidence is moderate regarding mortality outcomes among individuals who experienced delirium compared to those who did not. |

Title: Long-term clinical outcomes of delirium after hospital discharge

Patients or population: Those who experienced delirium and did not

Setting: After hospital discharge

Footnote. **GRADE working groups grades of evidence**

**High**: We are very confident that the true effect lies close to that of the estimate of the effect.

**Moderate**: We are moderately confident in the effect estimate: The true effect is likely to be close to the estimate of the effect, but there is a possibility that it is substantially different

**Low**: Our confidence in the effect estimate is limited: The true effect may be substantially different from the estimate of the effect.

**Very Low**: We have very little confidence in the effect estimate: The true effect is likely to be substantially different from the estimate of effect**.**

# Manuscript References (50–76)

50. Rosenthal JA. Qualitative Descriptors of Strength of Association and Effect Size. Journal of Social Service Research. 1996;21(4):37-59.

51. Higgins JP, Thompson SG, Deeks JJ, Altman DG. Measuring inconsistency in meta-analyses. Bmj. 2003 Sep 6;327(7414):557-60.

52. Borenstein M, Higgins JP, Hedges LV, Rothstein HR. Basics of meta-analysis: I(2) is not an absolute measure of heterogeneity. Res Synth Methods. 2017 Mar;8(1):5-18.

53. Dalton JE, Bolen SD, Mascha EJ. Publication Bias: The Elephant in the Review. Anesth Analg. 2016 Oct;123(4):812-3.

54. Schünemann H, Brożek J, Guyatt G, Oxman A. GRADE handbook for grading quality of evidence and strength of recommendations. Updated October 2013. The GRADE Working Group, 2013. 2021.

55. Kourkouta L, Iliadis C, Monios A. Psychosocial issues in elderly. Progress in health sciences. 2015;5(1):232-7.

56. Major ME, van Nes F, Ramaekers S, Engelbert RHH, van der Schaaf M. Survivors of Critical Illness and Their Relatives. A Qualitative Study on Hospital Discharge Experience. Ann Am Thorac Soc. 2019 Nov;16(11):1405-13.

57. Graf C. Functional Decline in Hospitalized Older Adults: It’s often a consequence of hospitalization, but it doesn’t have to be. AJN The American Journal of Nursing. 2006;106(1):58-67.

58. Larsson L, Degens H, Li M, Salviati L, Lee YI, Thompson W, et al. Sarcopenia: Aging-Related Loss of Muscle Mass and Function. Physiol Rev. 2019 Jan 1;99(1):427-511.

59. Sager MA, Rudberg MA. Functional Decline Associated with Hospitalization for Acute Illness. Clinics in Geriatric Medicine. 1998;14(4):669-80.

60. Fong TG, Jones RN, Shi P, Marcantonio ER, Yap L, Rudolph JL, et al. Delirium accelerates cognitive decline in Alzheimer disease. Neurology. 2009;72(18):1570-5.

61. Pereira JV, Aung Thein MZ, Nitchingham A, Caplan GA. Delirium in older adults is associated with development of new dementia: a systematic review and meta-analysis. Int J Geriatr Psychiatry. 2021 Jul;36(7):993-1003.

62. Yan E, Veitch M, Saripella A, Alhamdah Y, Butris N, Tang-Wai DF, et al. Association between postoperative delirium and adverse outcomes in older surgical patients: A systematic review and meta-analysis. J Clin Anesth. 2023 Nov;90:111221.

63. Hamilton GM, Wheeler K, Di Michele J, Lalu MM, McIsaac DI. A Systematic Review and Meta-analysis Examining the Impact of Incident Postoperative Delirium on Mortality. Anesthesiology. 2017 Jul;127(1):78-88.

64. Bai J, Liang Y, Zhang P, Liang X, He J, Wang J, et al. Association between postoperative delirium and mortality in elderly patients undergoing hip fractures surgery: a meta-analysis. Osteoporos Int. 2020 Feb;31(2):317-26.

65. Pezzullo L, Streatfeild J, Hickson J, Teodorczuk A, Agar MR, Caplan GA. Economic impact of delirium in Australia: a cost of illness study. BMJ Open. 2019 Sep 17;9(9):e027514.

66. Boncyk C, Rengel K, Stollings J, Marshall M, Feng X, Shotwell M, et al. Recurrent delirium episodes within the intensive care unit: Incidence and associated factors. J Crit Care. 2024 Feb;79:154490.

67. Quispel-Aggenbach DW, Zuidema SU, Luijendijk HJ. The prognosis of delirium in older outpatients. Psychogeriatrics. 2024 Mar;24(2):329-35.

68. Mortensen CB, Collet MO, Samuelson K. Struggling to return to everyday life-The experiences of quality of life 1 year after delirium in the intensive care unit. Nurs Crit Care. 2023 Sep;28(5):670-8.

69. Mahmoud A, Raghuraman S, Richards E, Morgan-Trimmer S, Goodwin VA, Anderson R, et al. Experience of carers for older people with delirium: a qualitative study. Aging Ment Health. 2024 Nov 22:1-8.

70. Allan L, O'Connell A, Raghuraman S, Bingham A, Laverick A, Chandler K, et al. A rehabilitation intervention to improve recovery after an episode of delirium in adults over 65 years (RecoverED): study protocol for a multi-centre, single-arm feasibility study. Pilot Feasibility Stud. 2023 Sep 15;9(1):162.

71. Brodaty H, Chau T, Heffernan M, Ginige JA, Andrews G, Millard M, et al. An online multidomain lifestyle intervention to prevent cognitive decline in at-risk older adults: a randomized controlled trial. Nature Medicine. 2025 2025/01/28.

72. Brodaty H, Valenzuela M, Singh MAF, Sachdev PS, McNeil J, Lautenschlager NT, et al. Maintain Your Brain: outcomes of an online program to prevent cognitive decline with aging. Alzheimer's & Dementia. 2023;19(S22).

73. Ngandu T, Lehtisalo J, Solomon A, Levalahti E, Ahtiluoto S, Antikainen R, et al. A 2 year multidomain intervention of diet, exercise, cognitive training, and vascular risk monitoring versus control to prevent cognitive decline in at-risk elderly people (FINGER): a randomised controlled trial. Lancet. 2015 Jun 6;385(9984):2255-63.

74. Cole MG. Persistent delirium in older hospital patients. Curr Opin Psychiatry. 2010 May;23(3):250-4.

75. Miu DK, Chan CW, Kok C. Delirium among elderly patients admitted to a post-acute care facility and 3-months outcome. Geriatr Gerontol Int. 2016 May;16(5):586-92.

76. Rieck KM, Pagali S, Miller DM. Delirium in hospitalized older adults. Hosp Pract (1995). 2020 Mar;48(sup1):3-16.

# References (Clinical outcomes used for analyses 1–253)

1. Abelha FJ, Luís C, Veiga D, Parente D, Fernandes V, Santos P, et al. Outcome and quality of life in patients with postoperative delirium during an ICU stay following major surgery. Crit Care. 2013 Oct 29;17(5):R257.

2. Adamis D, Treloar A, Darwiche FZ, Gregson N, Macdonald AJ, Martin FC. Associations of delirium with in-hospital and in 6-months mortality in elderly medical inpatients. Age Ageing. 2007 Nov;36(6):644-9.

3. Alberto RM, Domingo R, Aitor A, Sergio HM, Pascual P, Mireia P, et al. Long-term prognostic value of functional status and delirium in emergency patients with decompensated heart failure. Eur Geriatr Med. 2018 Aug;9(4):515-22.

4. Al Huraizi AR, Al-Maqbali JS, Al Farsi RS, Al Zeedy K, Al-Saadi T, Al-Hamadani N, et al. Delirium and Its Association with Short- and Long-Term Health Outcomes in Medically Admitted Patients: A Prospective Study. J Clin Med. 2023 Aug 17;12(16).

5. Alzoubi E, Shaheen F, Yousef K. Delirium incidence, predictors and outcomes in the intensive care unit: A prospective cohort study. Int J Nurs Pract. 2022 Feb;30(1):e13154.

6. Arneson ML, Oliveira JESL, Stanich JA, Jeffery MM, Lindroth HL, Ginsburg AD, et al. Association of delirium with increased short-term mortality among older emergency department patients: A cohort study. Am J Emerg Med. 2023 Apr;66:105-10.

7. Avelino-Silva TJ, Campora F, Curiati JA, Jacob-Filho W. Association between delirium superimposed on dementia and mortality in hospitalized older adults: A prospective cohort study. PLoS Med. 2017 Mar;14(3):e1002264.

8. Avelino-Silva TJ, Campora F, Curiati JAE, Jacob-Filho W. Prognostic effects of delirium motor subtypes in hospitalized older adults: A prospective cohort study. PLoS One. 2018;13(1):e0191092.

9. Bagienski M, Kleczynski P, Dziewierz A, Rzeszutko L, Sorysz D, Trebacz J, et al. Incidence of Postoperative Delirium and Its Impact on Outcomes After Transcatheter Aortic Valve Implantation. Am J Cardiol. 2017 Oct 1;120(7):1187-92.

10. Bakker RC, Osse RJ, Tulen JH, Kappetein AP, Bogers AJ. Preoperative and operative predictors of delirium after cardiac surgery in elderly patients. Eur J Cardiothorac Surg. 2012 Mar;41(3):544-9.

11. Beishuizen SJ, Festen S, Loonstra YE, van der Werf HW, de Rooij SE, van Munster BC. Delirium, functional decline and quality of life after transcatheter aortic valve implantation: An explorative study. Geriatr Gerontol Int. 2020 Dec;20(12):1202-7.

12. Bellelli G, Frisoni GB, Turco R, Lucchi E, Magnifico F, Trabucchi M. Delirium superimposed on dementia predicts 12-month survival in elderly patients discharged from a postacute rehabilitation facility. The Journals of Gerontology Series A: Biological Sciences and Medical Sciences. 2007;62(11):1306-9.

13. Bellelli G, Magnifico F, Trabucchi M. Outcomes at 12 months in a population of elderly patients discharged from a rehabilitation unit. J Am Med Dir Assoc. 2008 Jan;9(1):55-64.

14. Bellelli G, Carnevali L, Corsi M, Morandi A, Zambon A, Mazzola P, et al. The impact of psychomotor subtypes and duration of delirium on 6-month mortality in hip-fractured elderly patients. Int J Geriatr Psychiatry. 2018 May 31.

15. Bickel H, Gradinger R, Kochs E, Forstl H. High risk of cognitive and functional decline after postoperative delirium. A three-year prospective study. Dement Geriatr Cogn Disord. 2008;26(1):26-31.

16. Brown CHt, Probert J, Healy R, Parish M, Nomura Y, Yamaguchi A, et al. Cognitive Decline after Delirium in Patients Undergoing Cardiac Surgery. Anesthesiology. 2018 Sep;129(3):406-16.

17. Brown KN, Soo A, Faris P, Patten SB, Fiest KM, Stelfox HT. Association between delirium in the intensive care unit and subsequent neuropsychiatric disorders. Crit Care. 2020 Jul 31;24(1):476.

18. Bruck E, Schandl A, Bottai M, Sackey P. The impact of sepsis, delirium, and psychological distress on self-rated cognitive function in ICU survivors-a prospective cohort study. J Intensive Care. 2018;6:2.

19. Bryson GL, Wyand A, Wozny D, Rees L, Taljaard M, Nathan H. A prospective cohort study evaluating associations among delirium, postoperative cognitive dysfunction, and apolipoprotein E genotype following open aortic repair. Can J Anaesth. 2011 Mar;58(3):246-55.

20. Bulic D, Bennett M, Georgousopoulou EN, Shehabi Y, Pham T, Looi JCL, et al. Cognitive and psychosocial outcomes of mechanically ventilated intensive care patients with and without delirium. Ann Intensive Care. 2020 Aug 3;10(1):104.

21. Buurman BM, Hoogerduijn JG, de Haan RJ, Abu-Hanna A, Lagaay AM, Verhaar HJ, et al. Geriatric conditions in acutely hospitalized older patients: prevalence and one-year survival and functional decline. PLoS One. 2011;6(11):e26951.

22. Cartei A, Mossello E, Ceccofiglio A, Rubbieri G, Polidori G, Ranalli C, et al. Independent, Differential Effects of Delirium on Disability and Mortality Risk After Hip Fracture. J Am Med Dir Assoc. 2022 Apr;23(4):654-9 e1.

23. Cavallari M, Dai W, Guttmann CRG, Meier DS, Ngo LH, Hshieh TT, et al. Longitudinal diffusion changes following postoperative delirium in older people without dementia. Neurology. 2017 Sep 5;89(10):1020-7.

24. Chalmers LA, Searle SD, Whitby J, Tsui A, Davis D. Do specific delirium aetiologies have different associations with death? A longitudinal cohort of hospitalised patients. Eur Geriatr Med. 2021 Aug;12(4):787-91.

25. Chan KY, Cheng LS, Mak IW, Ng SW, Yiu MG, Chu CM. Delirium is a Strong Predictor of Mortality in Patients Receiving Non-invasive Positive Pressure Ventilation. Lung. 2016 Feb;195(1):115-25.

26. Chan EKW, Shen Q, Cordato D, Kneebone I, Xu YH, Chan DKY. Delirium post-stroke: short- to long-term effect on anxiety and depression compared to effect on cognition. Top Stroke Rehabil. 2017 Dec;24(8):597-600.

27. Chen Y, Ding S, Tao X, Feng X, Lu S, Shen Y, et al. The quality of life of patients developed delirium after coronary artery bypass grafting is determined by cognitive function after discharge: A cross-sectional study. Int J Nurs Pract. 2017 Oct;23(5).

28. Cheong JL, Shariffuddin II, Danaee M, Khor HM, Teang SC, San Loh P. Understanding risk factors for postoperative delirium after elective surgery in a university-based tertiary hospital. Neurology Asia. 2021;26(1).

29. Cirbus J, MacLullich AMJ, Noel C, Ely EW, Chandrasekhar R, Han JH. Delirium etiology subtypes and their effect on six-month function and cognition in older emergency department patients. Int Psychogeriatr. 2019 Feb;31(2):267-76.

30. Cole MG, You Y, McCusker J, Ciampi A, Belzile E. The 6 and 12 month outcomes of older medical inpatients who recover from delirium. Int J Geriatr Psychiatry. 2008 Mar;23(3):301-7.

31. Curyto KJ, Johnson J, TenHave T, Mossey J, Knott K, Katz IR. Survival of Hospitalized Elderly Patients With Delirium: A Prospective Study. The American Journal of Geriatric Psychiatry. 2001;9(2):141-7.

32. Czyzycki M, Klimiec-Moskal E, Chrobak AA, Pera J, Slowik A, Dziedzic T. Subtypes of delirium after ischaemic stroke-predisposing factors and outcomes: a prospective observational study (PROPOLIS). Eur J Neurol. 2022 Feb;29(2):478-85.

33. Daiello LA, Racine AM, Yun Gou R, Marcantonio ER, Xie Z, Kunze LJ, et al. Postoperative Delirium and Postoperative Cognitive Dysfunction: Overlap and Divergence. Anesthesiology. 2019 Sep;131(3):477-91.

34. Dani M, Owen LH, Jackson TA, Rockwood K, Sampson EL, Davis D. Delirium, Frailty, and Mortality: Interactions in a Prospective Study of Hospitalized Older People. J Gerontol A Biol Sci Med Sci. 2018 Mar 2;73(3):415-8.

35. Davis DH, Muniz Terrera G, Keage H, Rahkonen T, Oinas M, Matthews FE, et al. Delirium is a strong risk factor for dementia in the oldest-old: a population-based cohort study. Brain. 2012 Sep;135(Pt 9):2809-16.

36. Davis DH, Barnes LE, Stephan BC, MacLullich AM, Meagher D, Copeland J, et al. The descriptive epidemiology of delirium symptoms in a large population-based cohort study: results from the Medical Research Council Cognitive Function and Ageing Study (MRC CFAS). BMC geriatrics. 2014;14:1-8.

37. DeCrane SK, Culp KR, Wakefield B. Twelve-month mortality among delirium subtypes. Clin Nurs Res. 2011 Nov;20(4):404-21.

38. DeCrane SK, Culp KR, Wakefield B. Twelve-month fall outcomes among delirium subtypes. J Healthc Qual. 2012 Nov-Dec;34(6):13-20.

39. de Haan E, van Rijckevorsel V, Bod P, Roukema GR, de Jong L, Dutch Hip Fracture Registry C. Delirium After Surgery for Proximal Femoral Fractures in the Frail Elderly Patient: Risk Factors and Clinical Outcomes. Clin Interv Aging. 2023;18:193-203.

40. de Jong L, van Rijckevorsel V, Raats JW, Klem T, Kuijper TM, Roukema GR. Delirium after hip hemiarthroplasty for proximal femoral fractures in elderly patients: risk factors and clinical outcomes. Clin Interv Aging. 2019;14:427-35.

41. de la Varga-Martinez O, Gutierrez-Bustillo R, Munoz-Moreno MF, Lopez-Herrero R, Gomez-Sanchez E, Tamayo E. Postoperative delirium: An independent risk factor for poorer quality of life with long-term cognitive and functional decline after cardiac surgery. J Clin Anesth. 2023 May;85:111030.

42. Ditzel FL, van Montfort SJT, Vernooij LM, Kant IMJ, Aarts E, Spies CD, et al. Functional brain network and trail making test changes following major surgery and postoperative delirium: a prospective, multicentre, observational cohort study. Br J Anaesth. 2023 Feb;130(2):e281-e8.

43. Diwell RA, Davis DH, Vickerstaff V, Sampson EL. Key components of the delirium syndrome and mortality: greater impact of acute change and disorganised thinking in a prospective cohort study. BMC Geriatr. 2018 Jan 25;18(1):24.

44. Dostović Z, Ć Ibrahimagić O, Smajlović D, Kunić S, Čustović A. Cognitive functionality of patients with delirium after stroke. Psychiatria Danubina. 2021;33(suppl 4):503-10.

45. Drews T, Franck M, Radtke FM, Weiss B, Krampe H, Brockhaus WR, et al. Postoperative delirium is an independent risk factor for posttraumatic stress disorder in the elderly patient: a prospective observational study. Eur J Anaesthesiol. 2015 Mar;32(3):147-51.

46. Dros J, Kowalska K, Pasinska P, Szyper-Maciejowska A, Gorzkowska A, Klimkowicz-Mrowiec A. Delirium Post-Stroke-Influence on Post-Stroke Dementia (Research Study-Part of the PROPOLIS Study). J Clin Med. 2020 Jul 9;9(7).

47. Duppils GS, Wikblad K. Cognitive function and health-related quality of life after delirium in connection with hip surgery: a six-month follow-up. Orthopaedic nursing. 2004;23(3):195-203.

48. Duprey MS, van den Boogaard M, van der Hoeven JG, Pickkers P, Briesacher BA, Saczynski JS, et al. Association between incident delirium and 28- and 90-day mortality in critically ill adults: a secondary analysis. Crit Care. 2020 Apr 20;24(1):161.

49. Durlach M, Khoury M, Donato CL, Pérez EA, Iezzi NH, López R, et al. Delirium and subsyndromal delirium in the intensive care unit: In-hospital outcomes and prognosis at discharge. Medicina Clínica (English Edition). 2023;161(7):286-92.

50. Edelstein DM, Aharonoff GB, Karp A, Capla EL, Zuckerman JD, Koval KJ. Effect of postoperative delirium on outcome after hip fracture. Clin Orthop Relat Res. 2004 May(422):195-200.

51. Edlund A, Lundstrom M, Karlsson S, Brannstrom B, Bucht G, Gustafson Y. Delirium in older patients admitted to general internal medicine. J Geriatr Psychiatry Neurol. 2006 Jun;19(2):83-90.

52. Eeles EM, Hubbard RE, White SV, O'Mahony MS, Savva GM, Bayer AJ. Hospital use, institutionalisation and mortality associated with delirium. Age Ageing. 2010 Jul;39(4):470-5.

53. Eeles EM, White SV, O'Mahony SM, Bayer AJ, Hubbard RE. The impact of frailty and delirium on mortality in older inpatients. Age Ageing. 2012 May;41(3):412-6.

54. Eide LS, Ranhoff AH, Fridlund B, Haaverstad R, Hufthammer KO, Kuiper KK, et al. Delirium as a Predictor of Physical and Cognitive Function in Individuals Aged 80 and Older After Transcatheter Aortic Valve Implantation or Surgical Aortic Valve Replacement. J Am Geriatr Soc. 2016 Jun;64(6):1178-86.

55. Eide LS, Ranhoff AH, Fridlund B, Haaverstad R, Hufthammer KO, Kuiper KK, et al. Readmissions and mortality in delirious versus non-delirious octogenarian patients after aortic valve therapy: a prospective cohort study. BMJ Open. 2016 Oct 5;6(10):e012683.

56. Elsayem AF, Bruera E, Valentine A, Warneke CL, Wood GL, Yeung SJ, et al. Advance Directives, Hospitalization, and Survival Among Advanced Cancer Patients with Delirium Presenting to the Emergency Department: A Prospective Study. Oncologist. 2017 Nov;22(11):1368-73.

57. Evensen S, Hylen Ranhoff A, Lydersen S, Saltvedt I. The delirium screening tool 4AT in routine clinical practice: prediction of mortality, sensitivity and specificity. Eur Geriatr Med. 2021 Aug;12(4):793-800.

58. Falsini G, Grotti S, Porto I, Toccafondi G, Fraticelli A, Angioli P, et al. Long-term prognostic value of delirium in elderly patients with acute cardiac diseases admitted to two cardiac intensive care units: a prospective study (DELIRIUM CORDIS). Eur Heart J Acute Cardiovasc Care. 2018 Oct;7(7):661-70.

59. Fialho Silva IT, Assis Lopes P, Timotio Almeida T, Ramos SC, Caliman Fontes AT, Guimaraes Silva D, et al. Impact of Delirium and Its Motor Subtypes on Stroke Outcomes. Stroke. 2021 Apr;52(4):1322-9.

60. Fick DM, Steis MR, Waller JL, Inouye SK. Delirium superimposed on dementia is associated with prolonged length of stay and poor outcomes in hospitalized older adults. J Hosp Med. 2013 Sep;8(9):500-5.

61. Francis J, Martin D, Kapoor WN. A Prospective Study of Delirium in Hospitalized Elderly. JAMA. 1990;263(8):1097-101.

62. Francis J, Kapoor WN. Prognosis after hospital discharge of older medical patients with delirium. Journal of the American geriatrics society. 1992;40(6):601-6.

63. Franck M, Nerlich K, Neuner B, Schlattmann P, Brockhaus WR, Spies CD, et al. No convincing association between post-operative delirium and post-operative cognitive dysfunction: a secondary analysis. Acta Anaesthesiol Scand. 2016 Nov;60(10):1404-14.

64. Furlaneto ME, Garcez-Leme LE. Impact of delirium on mortality and cognitive and functional performance among elderly people with femoral fractures. Clinics (Sao Paulo). 2007 Oct;62(5):545-52.

65. Gandossi CM, Zambon A, Ferrara MC, Tassistro E, Castoldi G, Colombo F, et al. Frailty and post-operative delirium influence on functional status in patients with hip fracture: the GIOG 2.0 study. Aging Clin Exp Res. 2023 Nov;35(11):2499-506.

66. Garcez FB, Apolinario D, Campora F, Curiati JAE, Jacob-Filho W, Avelino-Silva TJ. Delirium and post-discharge dementia: results from a cohort of older adults without baseline cognitive impairment. Age Ageing. 2019 Nov 1;48(6):845-51.

67. George J, Bleasdale S, Singleton SJ. Causes and prognosis of delirium in elderly patients admitted to a district general hospital. Age and ageing. 1997;26(6):423-7.

68. Giroux M, Emond M, Nadeau A, Boucher V, Carmichael PH, Voyer P, et al. Functional and cognitive decline in older delirious adults after an emergency department visit. Age Ageing. 2021 Jan 8;50(1):135-40.

69. Givens JL, Sanft TB, Marcantonio ER. Functional recovery after hip fracture: the combined effects of depressive symptoms, cognitive impairment, and delirium. J Am Geriatr Soc. 2008 Jun;56(6):1075-9.

70. Givens JL, Jones RN, Inouye SK. The overlap syndrome of depression and delirium in older hospitalized patients. J Am Geriatr Soc. 2009 Aug;57(8):1347-53.

71. Gleason LJ, Schmitt EM, Kosar CM, Tabloski P, Saczynski JS, Robinson T, et al. Effect of Delirium and Other Major Complications on Outcomes After Elective Surgery in Older Adults. JAMA Surg. 2015 Dec;150(12):1134-40.

72. Goncalves NG, Aliberti MJR, Bertola L, Avelino-Silva T, Dias MB, Apolinario D, et al. Dissipating the fog: Cognitive trajectories and risk factors 1 year after COVID-19 hospitalization. Alzheimers Dement. 2023 Sep;19(9):3771-82.

73. González M, Pablo Jd, Valdés M, Matrai S, Peri JM. Delirium: A predictor of mortality in the elderly. The European journal of psychiatry. 2005;19(3):165-71.

74. Gonzalez M, Martinez G, Calderon J, Villarroel L, Yuri F, Rojas C, et al. Impact of delirium on short-term mortality in elderly inpatients: a prospective cohort study. Psychosomatics. 2009 May-Jun;50(3):234-8.

75. Gottschalk A, Hubbs J, Vikani AR, Gottschalk LB, Sieber FE. The Impact of Incident Postoperative Delirium on Survival of Elderly Patients After Surgery for Hip Fracture Repair. Anesth Analg. 2015 Nov;121(5):1336-43.

76. Gou RY, Hshieh TT, Marcantonio ER, Cooper Z, Jones RN, Travison TG, et al. One-Year Medicare Costs Associated With Delirium in Older Patients Undergoing Major Elective Surgery. JAMA Surg. 2021 May 1;156(5):430-42.

77. Goudzwaard JA, de Ronde-Tillmans M, de Jager TAJ, Lenzen MJ, Nuis RJ, van Mieghem NM, et al. Incidence, determinants and consequences of delirium in older patients after transcatheter aortic valve implantation. Age Ageing. 2020 Apr 27;49(3):389-94.

78. Gual N, Morandi A, Perez LM, Britez L, Burbano P, Man F, et al. Risk Factors and Outcomes of Delirium in Older Patients Admitted to Postacute Care with and without Dementia. Dement Geriatr Cogn Disord. 2018;45(1-2):121-9.

79. Guenther U, Hoffmann F, Dewald O, Malek R, Brimmers K, Theuerkauf N, et al. Preoperative Cognitive Impairment and Postoperative Delirium Predict Decline in Activities of Daily Living after Cardiac Surgery-A Prospective, Observational Cohort Study. Geriatrics (Basel). 2020 Oct 3;5(4).

80. Han JH, Shintani A, Eden S, Morandi A, Solberg LM, Schnelle J, et al. Delirium in the emergency department: an independent predictor of death within 6 months. Ann Emerg Med. 2010 Sep;56(3):244-52 e1.

81. Han JH, Brummel NE, Chandrasekhar R, Wilson JE, Liu X, Vasilevskis EE, et al. Exploring Delirium's Heterogeneity: Association Between Arousal Subtypes at Initial Presentation and 6-Month Mortality in Older Emergency Department Patients. Am J Geriatr Psychiatry. 2017 Mar;25(3):233-42.

82. Han JH, McNaughton CD, Stubblefield WB, Pang PS, Levy PD, Miller KF, et al. Delirium and its association with short-term outcomes in younger and older patients with acute heart failure. PLoS One. 2022;17(7):e0270889.

83. Hapca S, Guthrie B, Cvoro V, Bu F, Rutherford AC, Reynish E, et al. Mortality in people with dementia, delirium, and unspecified cognitive impairment in the general hospital: prospective cohort study of 6,724 patients with 2 years follow-up. Clin Epidemiol. 2018;10:1743-53.

84. Hawley S, Inman D, Gregson CL, Whitehouse M, Johansen A, Judge A. Risk Factors and 120-Day Functional Outcomes of Delirium After Hip Fracture Surgery: A Prospective Cohort Study Using the UK National Hip Fracture Database (NHFD). J Am Med Dir Assoc. 2023 May;24(5):694-701 e7.

85. Hempenius L, Slaets JP, van Asselt D, de Bock TH, Wiggers T, van Leeuwen BL. Long Term Outcomes of a Geriatric Liaison Intervention in Frail Elderly Cancer Patients. PLoS One. 2016;11(2):e0143364.

86. Hofhuis JGM, Schermer T, Spronk PE. Mental health-related quality of life is related to delirium in intensive care patients. Intensive Care Med. 2022 Sep;48(9):1197-205.

87. Holtta E, Laakkonen ML, Laurila JV, Strandberg TE, Tilvis R, Kautiainen H, et al. The overlap of delirium with neuropsychiatric symptoms among patients with dementia. Am J Geriatr Psychiatry. 2011 Dec;19(12):1034-41.

88. Honda S, Nagai T, Sugano Y, Okada A, Asaumi Y, Aiba T, et al. Prevalence, determinants, and prognostic significance of delirium in patients with acute heart failure. International journal of cardiology. 2016;222:521-7.

89. Hoogma DF, Venmans E, Al Tmimi L, Tournoy J, Verbrugghe P, Jacobs S, et al. Postoperative delirium and quality of life after transcatheter and surgical aortic valve replacement: A prospective observational study. J Thorac Cardiovasc Surg. 2023 Jul;166(1):156-66 e6.

90. Hshieh TT, Saczynski J, Gou RY, Marcantonio E, Jones RN, Schmitt E, et al. Trajectory of Functional Recovery After Postoperative Delirium in Elective Surgery. Ann Surg. 2017 Apr;265(4):647-53.

91. Hshieh TT, Gou RY, Jones RN, Leslie DL, Marcantonio ER, Xu G, et al. One-year Medicare costs associated with delirium in older hospitalized patients with and without Alzheimer's disease dementia and related disorders. Alzheimers Dement. 2023 May;19(5):1901-12.

92. Hughes CG, Hayhurst CJ, Pandharipande PP, Shotwell MS, Feng X, Wilson JE, et al. Association of Delirium during Critical Illness With Mortality: Multicenter Prospective Cohort Study. Anesth Analg. 2021 Nov 1;133(5):1152-61.

93. Humbert M, Bula CJ, Muller O, Krief H, Monney P. Delirium in older patients undergoing aortic valve replacement: incidence, predictors, and cognitive prognosis. BMC Geriatr. 2021 Mar 2;21(1):153.

94. Inouye SK, Peduzzi PN, Robison JT, Hughes JS, Horwitz RI, Concato J. Importance of Functional Measures in Predicting Mortality Among Older Hospitalized Patients. JAMA. 1998;279(15):1187-93.

95. Inouye SK, Marcantonio ER, Kosar CM, Tommet D, Schmitt EM, Travison TG, et al. The short-term and long-term relationship between delirium and cognitive trajectory in older surgical patients. Alzheimers Dement. 2016 Jul;12(7):766-75.

96. Isaia G, Astengo MA, Tibaldi V, Zanocchi M, Bardelli B, Obialero R, et al. Delirium in elderly home-treated patients: a prospective study with 6-month follow-up. Age (Dordr). 2009 Jun;31(2):109-17.

97. Iwata E, Kondo T, Kato T, Okumura T, Nishiyama I, Kazama S, et al. Prognostic Value of Delirium in Patients With Acute Heart Failure in the Intensive Care Unit. Can J Cardiol. 2020 Oct;36(10):1649-57.

98. Jackson JC, Pandharipande PP, Girard TD, Brummel NE, Thompson JL, Hughes CG, et al. Depression, post-traumatic stress disorder, and functional disability in survivors of critical illness in the BRAIN-ICU study: a longitudinal cohort study. Lancet Respir Med. 2014 May;2(5):369-79.

99. Jankowski CJ, Trenerry MR, Cook DJ, Buenvenida SL, Stevens SR, Schroeder DR, et al. Cognitive and functional predictors and sequelae of postoperative delirium in elderly patients undergoing elective joint arthroplasty. Anesth Analg. 2011 May;112(5):1186-93.

100. Janssen TL, de Vries J, Lodder P, Faes MC, Ho GH, Gobardhan PD, et al. The effects of elective aortic repair, colorectal cancer surgery and subsequent postoperative delirium on long-term quality of life, cognitive functioning and depressive symptoms in older patients. Aging Ment Health. 2021 May;25(5):896-905.

101. Juliebo V, Krogseth M, Skovlund E, Engedal K, Ranhoff AH, Wyller TB. Delirium is not associated with mortality in elderly hip fracture patients. Dement Geriatr Cogn Disord. 2010;30(2):112-20.

102. Kainz E, Stuff K, Kahl U, Wiessner C, Yu Y, von Breunig F, et al. Impact of postanesthesia care unit delirium on self-reported cognitive function and perceived health status: a prospective observational cohort study. Qual Life Res. 2022 Aug;31(8):2397-410.

103. Kakuma R, du Fort GG, Arsenault L, Perrault A, Platt RW, Monette J, et al. Delirium in older emergency department patients discharged home: effect on survival. J Am Geriatr Soc. 2003 Apr;51(4):443-50.

104. Kat MG, Vreeswijk R, de Jonghe JF, van der Ploeg T, van Gool WA, Eikelenboom P, et al. Long-term cognitive outcome of delirium in elderly hip surgery patients. A prospective matched controlled study over two and a half years. Dement Geriatr Cogn Disord. 2008;26(1):1-8.

105. Kat MG, de Jonghe JF, Vreeswijk R, van der Ploeg T, van Gool WA, Eikelenboom P, et al. Mortality associated with delirium after hip-surgery: a 2-year follow-up study. Age Ageing. 2011 May;40(3):312-8.

106. Katz IR, Curyto KJ, TenHave T, Mossey J, Sands L, Kallan MJ. Validating the diagnosis of delirium and evaluating its association with deterioration over a one-year period. Focus. 2001;9(2):148-323.

107. Kennedy M, Enander RA, Tadiri SP, Wolfe RE, Shapiro NI, Marcantonio ER. Delirium risk prediction, healthcare use and mortality of elderly adults in the emergency department. J Am Geriatr Soc. 2014 Mar;62(3):462-9.

108. Kilicaslan I, Thet MS, Karahacioglu B, Sevim M, Ulger Z, Aslaner MA, et al. Delirium, dementia, and depression (3D) assessment of older patients in the emergency department: 5-year survival analysis. Turk J Med Sci. 2022 Apr;52(2):380-96.

109. Knauf T, Bucking B, Bargello M, Ploch S, Bliemel C, Knobe M, et al. Predictors of long-term survival after hip fractures?-5-year results of a prospective study in Germany. Arch Osteoporos. 2019 Mar 16;14(1):40.

110. Korber MI, Schafer M, Vimalathasan R, Mauri V, Iliadis C, Metze C, et al. Periinterventional inflammation and blood transfusions predict postprocedural delirium after percutaneous repair of mitral and tricuspid valves. Clin Res Cardiol. 2021 Dec;110(12):1921-9.

111. Koster S, Hensens AG, Schuurmans MJ, van der Palen J. Consequences of delirium after cardiac operations. Ann Thorac Surg. 2012 Mar;93(3):705-11.

112. Kotfis K, Bott-Olejnik M, Szylinska A, Listewnik M, Rotter I. Characteristics, Risk Factors And Outcome Of Early-Onset Delirium In Elderly Patients With First Ever Acute Ischemic Stroke - A Prospective Observational Cohort Study. Clin Interv Aging. 2019;14:1771-82.

113. Krogseth M, Wyller TB, Engedal K, Juliebo V. Delirium is an important predictor of incident dementia among elderly hip fracture patients. Dement Geriatr Cogn Disord. 2011;31(1):63-70.

114. Krogseth M, Wyller TB, Engedal K, Juliebo V. Delirium is a risk factor for institutionalization and functional decline in older hip fracture patients. J Psychosom Res. 2014 Jan;76(1):68-74.

115. Krogseth M, Watne LO, Juliebo V, Skovlund E, Engedal K, Frihagen F, et al. Delirium is a risk factor for further cognitive decline in cognitively impaired hip fracture patients. Arch Gerontol Geriatr. 2016 May-Jun;64:38-44.

116. Krogseth M, Davis D, Jackson TA, Zetterberg H, Watne LO, Lindberg M, et al. Delirium, neurofilament light chain, and progressive cognitive impairment: analysis of a prospective Norwegian population-based cohort. Lancet Healthy Longev. 2023 Aug;4(8):e399-e408.

117. Krzych LJ, Wybraniec MT, Krupka-Matuszczyk I, Skrzypek M, Bolkowska A, Wilczynski M, et al. Detailed insight into the impact of postoperative neuropsychiatric complications on mortality in a cohort of cardiac surgery subjects: a 23,000-patient-year analysis. J Cardiothorac Vasc Anesth. 2014 Jun;28(3):448-57.

118. Kunicki ZJ, Ngo LH, Marcantonio ER, Tommet D, Feng Y, Fong TG, et al. Six-Year Cognitive Trajectory in Older Adults Following Major Surgery and Delirium. JAMA Intern Med. 2023 May 1;183(5):442-50.

119. Labaste F, Porterie J, Bousquet P, Marcheix B, Sanchez-Verlaan P, Frances B, et al. Postoperative Delirium is a Risk Factor of Poor Evolution Three Years After Cardiac Surgery: An Observational Cohort Study. Clin Interv Aging. 2020;15:2375-81.

120. Labaste F, Delort F, Ferre F, Bounes F, Reina N, Valet P, et al. Postoperative delirium is a risk factor of institutionalization after hip fracture: an observational cohort study. Front Med (Lausanne). 2023;10:1165734.

121. Large MC, Reichard C, Williams JT, Chang C, Prasad S, Leung Y, et al. Incidence, risk factors, and complications of postoperative delirium in elderly patients undergoing radical cystectomy. Urology. 2013 Jan;81(1):123-8.

122. Lee KH, Ha YC, Lee YK, Kang H, Koo KH. Frequency, risk factors, and prognosis of prolonged delirium in elderly patients after hip fracture surgery. Clin Orthop Relat Res. 2011 Sep;469(9):2612-20.

123. Lee HB, Oldham MA, Sieber FE, Oh ES. Impact of Delirium After Hip Fracture Surgery on One-Year Mortality in Patients With or Without Dementia: A Case of Effect Modification. Am J Geriatr Psychiatry. 2017 Mar;25(3):308-15.

124. Lee A, Mu JL, Chiu CH, Gin T, Underwood MJ, Joynt GM. Effect of motor subtypes of delirium in the intensive care unit on fast-track failure after cardiac surgery. J Thorac Cardiovasc Surg. 2018 Jan;155(1):268-75 e1.

125. Leslie DL, Zhang Y, Holford TR, Bogardus ST, Leo-Summers LS, Inouye SK. Premature death associated with delirium at 1-year follow-up. Archives of internal medicine. 2005;165(14):1657-62.

126. Leslie DL, Marcantonio ER, Zhang Y, Leo-Summers L, Inouye SK. One-year health care costs associated with delirium in the elderly population. Archives of internal medicine. 2008;168(1):27-32.

127. Lewis LM, Miller DK, Morley JE, Nork MJ, Lasater LC. Unrecognized delirium in ED geriatric patients. The American journal of emergency medicine. 1995;13(2):142-5.

128. Li S, Zhang XH, Zhou GD, Wang JF. Delirium after primary percutaneous coronary intervention in aged individuals with acute ST-segment elevation myocardial infarction: A retrospective study. Exp Ther Med. 2019 May;17(5):3807-13.

129. Li HC, Wei YC, Hsu RB, Chi NH, Wang SS, Chen YS, et al. Surviving and Thriving 1 Year After Cardiac Surgery: Frailty and Delirium Matter. Ann Thorac Surg. 2021 May;111(5):1578-84.

130. Liang CK, Chu CL, Chou MY, Lin YT, Lu T, Hsu CJ, et al. Interrelationship of postoperative delirium and cognitive impairment and their impact on the functional status in older patients undergoing orthopaedic surgery: a prospective cohort study. PLoS One. 2014;9(11):e110339.

131. Lima DP, Ochiai ME, Lima AB, Curiati JA, Farfel JM, Filho WJ. Delirium in hospitalized elderly patients and post-discharge mortality. Clinics (Sao Paulo). 2010 Mar;65(3):251-5.

132. Lingehall HC, Smulter NS, Lindahl E, Lindkvist M, Engstrom KG, Gustafson YG, et al. Preoperative Cognitive Performance and Postoperative Delirium Are Independently Associated With Future Dementia in Older People Who Have Undergone Cardiac Surgery: A Longitudinal Cohort Study. Crit Care Med. 2017 Aug;45(8):1295-303.

133. Lundstrom M, Edlund A, Bucht G, Karlsson S, Gustafson Y. Dementia after delirium in patients with femoral neck fractures. J Am Geriatr Soc. 2003 Jul;51(7):1002-6.

134. Luz L, Santos MCD, Ramos TA, Almeida CB, Rover MC, Dal'Pizzol CP, et al. Delirium and quality of life in critically ill patients: a prospective cohort study. Rev Bras Ter Intensiva. 2020 Jul-Sep;32(3):426-32.

135. MacLullich AM, Shenkin SD, Goodacre S, Godfrey M, Hanley J, Stiobhairt A, et al. The 4 'A's test for detecting delirium in acute medical patients: a diagnostic accuracy study. Health Technol Assess. 2019 Aug;23(40):1-194.

136. Marcantonio ER, Kiely DK, Simon SE, John Orav E, Jones RN, Murphy KM, et al. Outcomes of older people admitted to postacute facilities with delirium. J Am Geriatr Soc. 2005 Jun;53(6):963-9.

137. Mariz J, Santos NC, Afonso H, Rodrigues P, Faria A, Sousa N, et al. Risk and clinical-outcome indicators of delirium in an emergency department intermediate care unit (EDIMCU): an observational prospective study. BMC emergency medicine. 2013;13:1-8.

138. Marrama F, Kyheng M, Pasi M, Pierre Rutgers M, Moulin S, Diomedi M, et al. Early-onset delirium after spontaneous intracerebral hemorrhage. Int J Stroke. 2022 Oct;17(9):1030-8.

139. Mathies F, Lange C, Maurer A, Apostolova I, Klutmann S, Buchert R. Brain FDG PET for the Etiological Diagnosis of Clinically Uncertain Cognitive Impairment During Delirium in Remission. J Alzheimers Dis. 2020;77(4):1609-22.

140. Mauri V, Reuter K, Korber MI, Wienemann H, Lee S, Eghbalzadeh K, et al. Incidence, Risk Factors and Impact on Long-Term Outcome of Postoperative Delirium After Transcatheter Aortic Valve Replacement. Front Cardiovasc Med. 2021;8:645724.

141. Mazzola P, Bellelli G, Broggini V, Anzuini A, Corsi M, Berruti D, et al. Postoperative delirium and pre-fracture disability predict 6-month mortality among the oldest old hip fracture patients. Aging Clin Exp Res. 2015 Feb;27(1):53-60.

142. McAvay GJ, Van Ness PH, Bogardus ST, Jr., Zhang Y, Leslie DL, Leo-Summers LS, et al. Older adults discharged from the hospital with delirium: 1-year outcomes. J Am Geriatr Soc. 2006 Aug;54(8):1245-50.

143. McCusker J, Cole M, Dendukuri N, Belzile É, Primeau F. Delirium in older medical inpatients and subsequent cognitive and functional status: a prospective study. Cmaj. 2001;165(5):575-83.

144. McCusker J, Cole M, Abrahamowicz M, Primeau F, Belzile E. Delirium Predicts 12-Month Mortality. Archives of Internal Medicine. 2002;162(4):457-63.

145. McCusker J, Cole MG, Voyer P, Monette J, Champoux N, Ciampi A, et al. Six-month outcomes of co-occurring delirium, depression, and dementia in long-term care. J Am Geriatr Soc. 2014 Dec;62(12):2296-302.

146. Minden SL, Carbone LA, Barsky A, Borus JF, Fife A, Fricchione GL, et al. Predictors and outcomes of delirium. Gen Hosp Psychiatry. 2005 May-Jun;27(3):209-14.

147. Miu DK, Yeung JC. Incidence of post-stroke delirium and 1-year outcome. Geriatr Gerontol Int. 2013 Jan;13(1):123-9.

148. Miyamoto K, Shibata M, Shima N, Nakashima T, Kida M, Matsumoto H, et al. Combination of delirium and coma predicts psychiatric symptoms at twelve months in critically ill patients: A longitudinal cohort study. J Crit Care. 2021 Jun;63:76-82.

149. Monacelli F, Pizzonia M, Signori A, Nencioni A, Giannotti C, Minaglia C, et al. The In-Hospital Length of Stay after Hip Fracture in Octogenarians: Do Delirium and Dementia Shape a New Care Process? J Alzheimers Dis. 2018;66(1):281-8.

150. Moon KJ, Park H. Outcomes of patients with delirium in long-term care facilities: A prospective cohort study. Journal of Gerontological Nursing. 2018;44(9):41-50.

151. Morandi A, Davis D, Fick DM, Turco R, Boustani M, Lucchi E, et al. Delirium superimposed on dementia strongly predicts worse outcomes in older rehabilitation inpatients. J Am Med Dir Assoc. 2014 May;15(5):349-54.

152. Moreno-Gaviño L, Ruiz-Cantero A, Bernabeu-Wittel M, Tejera-Concepción A, Romero-Jiménez M, Soria MÁ, et al. Impact of Cognitive Impairment in a Multicentric Cohort of Polypathological Patients. International Journal of Gerontology. 2012;6(2):84-9.

153. Moskowitz EE, Overbey DM, Jones TS, Jones EL, Arcomano TR, Moore JT, et al. Post-operative delirium is associated with increased 5-year mortality. Am J Surg. 2017 Dec;214(6):1036-8.

154. Muller J, Nowak S, Weidemeier M, Vogelgesang A, Ruhnau J, von Sarnowski B, et al. Duration of Surgery and Intraoperative Blood Pressure Management Are Modifiable Risk Factors for Postoperative Neurocognitive Disorders After Spine Surgery: Results of the Prospective CONFESS Study. Spine (Phila Pa 1976). 2023 Aug 15;48(16):1127-37.

155. Muresan ML, Adamis D, Murray O, O'Mahony E, McCarthy G. Delirium, how does it end? Mortality as an outcome in older medical inpatients. Int J Geriatr Psychiatry. 2016 Apr;31(4):349-54.

156. Murray AM, Levkoff SE, Wetle TT, Beckett L, Cleary PD, Schor JD, et al. Acute delirium and functional decline in the hospitalized elderly patient. Journal of gerontology. 1993;48(5):M181-M6.

157. Muzzana C, Mantovan F, Huber MK, Trevisani K, Niederbacher S, Kugler A, et al. Delirium in elderly postoperative patients: A prospective cohort study. Nurs Open. 2022 Sep;9(5):2461-72.

158. Naidech AM, Beaumont JL, Rosenberg NF, Maas MB, Kosteva AR, Ault ML, et al. Intracerebral hemorrhage and delirium symptoms. Length of stay, function, and quality of life in a 114-patient cohort. Am J Respir Crit Care Med. 2013 Dec 1;188(11):1331-7.

159. Neerland BE, Krogseth M, Juliebo V, Hylen Ranhoff A, Engedal K, Frihagen F, et al. Perioperative hemodynamics and risk for delirium and new onset dementia in hip fracture patients; A prospective follow-up study. PLoS One. 2017;12(7):e0180641.

160. Nerdal V, Gjestad E, Saltvedt I, Munthe-Kaas R, Ihle-Hansen H, Ryum T, et al. The relationship of acute delirium with cognitive and psychiatric symptoms after stroke: a longitudinal study. BMC Neurol. 2022 Jun 27;22(1):234.

161. Neufeld KJ, Leoutsakos JM, Oh E, Sieber FE, Chandra A, Ghosh A, et al. Long-Term Outcomes of Older Adults with and Without Delirium Immediately After Recovery from General Anesthesia for Surgery. Am J Geriatr Psychiatry. 2015 Oct;23(10):1067-74.

162. Nguyen Q, Uminski K, Hiebert BM, Tangri N, Arora RC. Midterm outcomes after postoperative delirium on cognition and mood in patients after cardiac surgery. J Thorac Cardiovasc Surg. 2018 Feb;155(2):660-7 e2.

163. Nishizawa Y, Yamanashi T, Saito T, Marra P, Crutchley KJ, Wahba NE, et al. Bispectral EEG (BSEEG) Algorithm Captures High Mortality Risk Among 1,077 Patients: Its Relationship to Delirium Motor Subtype. Am J Geriatr Psychiatry. 2023 Sep;31(9):704-15.

164. Noriega FJ, Vidan MT, Sanchez E, Diaz A, Serra-Rexach JA, Fernandez-Aviles F, et al. Incidence and impact of delirium on clinical and functional outcomes in older patients hospitalized for acute cardiac diseases. Am Heart J. 2015 Nov;170(5):938-44.

165. Ogawa M, Izawa KP, Satomi-Kobayashi S, Tsuboi Y, Komaki K, Gotake Y, et al. Impact of delirium on postoperative frailty and long term cardiovascular events after cardiac surgery. PLoS One. 2017;12(12):e0190359.

166. Ojagbemi A, Bello T, Elugbadebo O, Owolabi M, Baiyewu O. Different Cognitive and Functional Outcomes in Attenuated and Full Delirium Syndromes Among Recent Stroke Survivors. J Stroke Cerebrovasc Dis. 2020 Nov;29(11):105251.

167. Ojagbemi A, Bello T, Owolabi M, Baiyewu O. The Independent Association of Prestroke Psychiatric Symptoms and Acute Phase Delirium with Poststroke Mortality at One Year in Nigeria. J Stroke Cerebrovasc Dis. 2021 Apr;30(4):105622.

168. O'Keeffe S, Lavan J. The prognostic significance of delirium in older hospital patients. J Am Geriatr Soc. 1997 Feb;45(2):174-8.

169. Oldenbeuving A, De Kort P, Jansen B, Algra A, Kappelle L, Roks G. Delirium in the acute phase after stroke: incidence, risk factors, and outcome. Neurology. 2011;76(11):993-9.

170. Olofsson B, Persson M, Bellelli G, Morandi A, Gustafson Y, Stenvall M. Development of dementia in patients with femoral neck fracture who experience postoperative delirium-A three-year follow-up study. Int J Geriatr Psychiatry. 2018 Apr;33(4):623-32.

171. Pak M, Hara M, Miura S, Furuya M, Tamaki M, Okada T, et al. Delirium is associated with high mortality in older adult patients with acute decompensated heart failure. BMC Geriatr. 2020 Dec 3;20(1):524.

172. Pandharipande PP, Girard TD, Jackson JC, Morandi A, Thompson JL, Pun BT, et al. Long-term cognitive impairment after critical illness. N Engl J Med. 2013 Oct 3;369(14):1306-16.

173. Pasinska P, Wilk A, Kowalska K, Szyper-Maciejowska A, Klimkowicz-Mrowiec A. The long-term prognosis of patients with delirium in the acute phase of stroke: PRospective Observational POLIsh Study (PROPOLIS). J Neurol. 2019 Nov;266(11):2710-7.

174. Patel SB, Poston JT, Pohlman A, Hall JB, Kress JP. Rapidly reversible, sedation-related delirium versus persistent delirium in the intensive care unit. Am J Respir Crit Care Med. 2014 Mar 15;189(6):658-65.

175. Paulino MC, Conceicao C, Silvestre J, Lopes MI, Goncalves H, Dias CC, et al. Subsyndromal Delirium in Critically Ill Patients-Cognitive and Functional Long-Term Outcomes. J Clin Med. 2023 Oct 4;12(19).

176. Pendlebury ST, Lovett NG, Smith SC, Dutta N, Bendon C, Lloyd-Lavery A, et al. Observational, longitudinal study of delirium in consecutive unselected acute medical admissions: age-specific rates and associated factors, mortality and re-admission. BMJ Open. 2015 Nov 16;5(11):e007808.

177. Penfold RS, Hall AJ, Anand A, Clement ND, Duckworth AD, MacLullich AMJ. Delirium in hip fracture patients admitted from home during the COVID-19 pandemic is associated with higher mortality, longer total length of stay, need for post-acute inpatient rehabilitation, and readmission to acute services. Bone Jt Open. 2023 Jun 16;4(6):447-56.

178. Pitkala KH, Laurila JV, Strandberg TE, Tilvis RS. Prognostic significance of delirium in frail older people. Dement Geriatr Cogn Disord. 2005;19(2-3):158-63.

179. Pompei P, Foreman M, Rudberg MA, Inouye SK, Braund V, Cassel CK. Delirium in hospitalized older persons: outcomes and predictors. Journal of the American Geriatrics Society. 1994;42(8):809-15.

180. Praditsuwan R, Sirisuwat A, Assanasen J, Eiamjinnasuwat W, Pakdeewongse S, Limmathuroskul D, et al. Short-term clinical outcomes in delirious older patients: a study at general medical wards in a university hospital in Thailand. Geriatr Gerontol Int. 2013 Oct;13(4):972-7.

181. Qu J, Chen Y, Luo G, Zhong H, Xiao W, Yin H. Delirium in the Acute Phase of Ischemic Stroke: Incidence, Risk Factors, and Effects on Functional Outcome. J Stroke Cerebrovasc Dis. 2018 Oct;27(10):2641-7.

182. Quinlan N, Rudolph JL. Postoperative delirium and functional decline after noncardiac surgery. J Am Geriatr Soc. 2011 Nov;59 Suppl 2:S301-4.

183. Raats JW, van Eijsden WA, Crolla RM, Steyerberg EW, van der Laan L. Risk Factors and Outcomes for Postoperative Delirium after Major Surgery in Elderly Patients. PLoS One. 2015;10(8):e0136071.

184. Racine AM, Fong TG, Gou Y, Travison TG, Tommet D, Erickson K, et al. Clinical outcomes in older surgical patients with mild cognitive impairment. Alzheimers Dement. 2018 May;14(5):590-600.

185. Racine AM, Touroutoglou A, Abrantes T, Wong B, Fong TG, Cavallari M, et al. Older Patients with Alzheimer's Disease-Related Cortical Atrophy Who Develop Post-Operative Delirium May Be at Increased Risk of Long-Term Cognitive Decline After Surgery. J Alzheimers Dis. 2020;75(1):187-99.

186. Radcliffe NJ, Lau L, Hack E, Huynh A, Puri A, Yao H, et al. Site of care and factors associated with mortality in unvaccinated Australian aged care residents during COVID-19 outbreaks. Intern Med J. 2023 May;53(5):690-9.

187. Radinovic KS, Markovic-Denic L, Dubljanin-Raspopovic E, Marinkovic J, Jovanovic LB, Bumbasirevic V. Effect of the overlap syndrome of depressive symptoms and delirium on outcomes in elderly adults with hip fracture: a prospective cohort study. J Am Geriatr Soc. 2014 Sep;62(9):1640-8.

188. Rahkonen T, Eloniemi-Sulkava U, Halonen P, Verkkoniemi A, Niinisto L, Notkola IL, et al. Delirium in the non-demented oldest old in the general population: risk factors and prognosis. Int J Geriatr Psychiatry. 2001 Apr;16(4):415-21.

189. Rawle MJ, McCue L, Sampson EL, Davis D, Vickerstaff V. Anticholinergic Burden Does Not Influence Delirium Subtype or the Delirium-Mortality Association in Hospitalized Older Adults: Results from a Prospective Cohort Study. Drugs Aging. 2021 Mar;38(3):233-42.

190. Rego LLD, Salluh JIF, Souza-Dantas VC, Silva J, Povoa P, Serafim RB. Delirium severity and outcomes of critically ill COVID-19 patients. Crit Care Sci. 2023 Oct-Dec;35(4):394-401.

191. Reynish EL, Hapca SM, De Souza N, Cvoro V, Donnan PT, Guthrie B. Epidemiology and outcomes of people with dementia, delirium, and unspecified cognitive impairment in the general hospital: prospective cohort study of 10,014 admissions. BMC Med. 2017 Jul 27;15(1):140.

192. Richardson SJ, Davis DHJ, Stephan BCM, Robinson L, Brayne C, Barnes LE, et al. Recurrent delirium over 12 months predicts dementia: results of the Delirium and Cognitive Impact in Dementia (DECIDE) study. Age Ageing. 2021 May 5;50(3):914-20.

193. Richardson SJ, Lawson R, Davis DHJ, Stephan BCM, Robinson L, Matthews FE, et al. Hospitalisation without delirium is not associated with cognitive decline in a population-based sample of older people-results from a nested, longitudinal cohort study. Age Ageing. 2021 Sep 11;50(5):1675-81.

194. Rizzi MA, Torres Bonafonte OH, Alquezar A, Herrera Mateo S, Pinera P, Puig M, et al. Prognostic value and risk factors of delirium in emergency patients with decompensated heart failure. J Am Med Dir Assoc. 2015 Sep 1;16(9):799 e1-6.

195. Robinson TN, Raeburn CD, Tran ZV, Angles EM, Brenner LA, Moss M. Postoperative delirium in the elderly: risk factors and outcomes. Ann Surg. 2009 Jan;249(1):173-8.

196. Robinson TN, Raeburn CD, Tran ZV, Brenner LA, Moss M. Motor subtypes of postoperative delirium in older adults. Archives of Surgery. 2011;146(3):295-300.

197. Rockwood K, Cosway S, Carver D, Jarrett P, Stadnyk K, Fisk J. The risk of dementia and death after delirium. Age and ageing. 1999;28(6):551-6.

198. Rolandi E, Zaccaria D, Vaccaro R, Abbondanza S, Pettinato L, Davin A, et al. Estimating the potential for dementia prevention through modifiable risk factors elimination in the real-world setting: a population-based study. Alzheimers Res Ther. 2020 Aug 7;12(1):94.

199. Rollo E, Brunetti V, Scala I, Callea A, Marotta J, Vollono C, et al. Impact of delirium on the outcome of stroke: a prospective, observational, cohort study. J Neurol. 2022 Dec;269(12):6467-75.

200. Rosenthal LJ, Francis BA, Beaumont JL, Cella D, Berman MD, Maas MB, et al. Agitation, Delirium, and Cognitive Outcomes in Intracerebral Hemorrhage. Psychosomatics. 2017 Jan-Feb;58(1):19-27.

201. Rudolph JL, Marcantonio ER, Culley DJ, Silverstein JH, Rasmussen LS, Crosby GJ, et al. Delirium is associated with early postoperative cognitive dysfunction. Anaesthesia. 2008 Sep;63(9):941-7.

202. Rudolph JL, Inouye SK, Jones RN, Yang FM, Fong TG, Levkoff SE, et al. Delirium: an independent predictor of functional decline after cardiac surgery. J Am Geriatr Soc. 2010 Apr;58(4):643-9.

203. Ruggiero C, Bonamassa L, Pelini L, Prioletta I, Cianferotti L, Metozzi A, et al. Early post-surgical cognitive dysfunction is a risk factor for mortality among hip fracture hospitalized older persons. Osteoporos Int. 2017 Feb;28(2):667-75.

204. Saczynski JS, Marcantonio ER, Quach L, Fong TG, Gross A, Inouye SK, et al. Cognitive trajectories after postoperative delirium. N Engl J Med. 2012 Jul 5;367(1):30-9.

205. Sánchez-Lozano ML, Restrepo-Ramírez CD, Serna-Echeverri LS, Franco-Ramírez JD, Gutiérrez-Segura JC, García-Cuevas AM. Delirium: Cognitive, Functionality, and Quality of Life Outcomes in Critically Ill Patients. Revista Colombiana de Psiquiatría. 2023.

206. Sanguanwit P, Ninlamal S, Prachanukool T. Thirty-day mortality among patients with acute delirium in the emergency department. Heliyon. 2023 Oct;9(10):e20554.

207. Sasajima Y, Sasajima T, Azuma N, Akazawa K, Saito Y, Inaba M, et al. Factors related to postoperative delirium in patients with lower limb ischaemia: a prospective cohort study. Eur J Vasc Endovasc Surg. 2012 Oct;44(4):411-5.

208. Sato K, Kubota K, Oda H, Taniguchi T. The impact of delirium on outcomes in acute, non-intubated cardiac patients. Eur Heart J Acute Cardiovasc Care. 2017 Sep;6(6):553-9.

209. Sauer AC, Veldhuijzen DS, Ottens TH, Slooter AJC, Kalkman CJ, van Dijk D. Association between delirium and cognitive change after cardiac surgery. Br J Anaesth. 2017 Aug 1;119(2):308-15.

210. Serrano-Duenas M, Bleda MJ. Delirium in Parkinson's disease patients. a five-year follow-up study. Parkinsonism Relat Disord. 2005 Sep;11(6):387-92.

211. Sheng AZ, Shen Q, Cordato D, Zhang YY, Yin Chan DK. Delirium within three days of stroke in a cohort of elderly patients. J Am Geriatr Soc. 2006 Aug;54(8):1192-8.

212. Shi SM, Sung M, Afilalo J, Lipsitz LA, Kim CA, Popma JJ, et al. Delirium Incidence and Functional Outcomes After Transcatheter and Surgical Aortic Valve Replacement. J Am Geriatr Soc. 2019 Jul;67(7):1393-401.

213. Shi Z, Mei X, Li C, Chen Y, Zheng H, Wu Y, et al. Postoperative Delirium Is Associated with Long-term Decline in Activities of Daily Living. Anesthesiology. 2019 Sep;131(3):492-500.

214. Shim J, DePalma G, Sands LP, Leung JM. Prognostic Significance of Postoperative Subsyndromal Delirium. Psychosomatics. 2015 Nov-Dec;56(6):644-51.

215. Shintani AK, Girard TD, Eden SK, Arbogast PG, Moons KG, Ely EW. Immortal time bias in critical care research: application of time-varying Cox regression for observational cohort studies. Crit Care Med. 2009 Nov;37(11):2939-45.

216. Singler K, Thiem U, Christ M, Zenk P, Biber R, Sieber CC, et al. Aspects and assessment of delirium in old age. First data from a German interdisciplinary emergency department. Z Gerontol Geriatr. 2014 Dec;47(8):680-5.

217. Slor CJ, Witlox J, Jansen RW, Adamis D, Meagher DJ, Tieken E, et al. Affective functioning after delirium in elderly hip fracture patients. Int Psychogeriatr. 2013 Mar;25(3):445-55.

218. Sri-on J, Tirrell GP, Vanichkulbodee A, Niruntarai S, Liu SW. The prevalence, risk factors and short-term outcomes of delirium in Thai elderly emergency department patients. Emerg Med J. 2016 Jan;33(1):17-22.

219. Suraarunsumrit P, Pathonsmith C, Srinonprasert V, Sangarunakul N, Jiraphorncharas C, Siriussawakul A. Postoperative cognitive dysfunction in older surgical patients associated with increased healthcare utilization: a prospective study from an upper-middle-income country. BMC Geriatr. 2022 Mar 16;22(1):213.

220. Svenningsen H, Tonnesen EK, Videbech P, Frydenberg M, Christensen D, Egerod I. Intensive care delirium - effect on memories and health-related quality of life - a follow-up study. J Clin Nurs. 2014 Mar;23(5-6):634-44.

221. Svenningsen H, Egerod I, Christensen D, Tonnesen EK, Frydenberg M, Videbech P. Symptoms of Posttraumatic Stress after Intensive Care Delirium. Biomed Res Int. 2015;2015:876947.

222. Tahir M, Malik SS, Ahmed U, Kozdryk J, Naqvi SH, Malik A. Risk factors for onset of delirium after neck of femur fracture surgery: a prospective observational study. SICOT J. 2018;4:27.

223. Tan AH, Scott J. Association of point prevalence diagnosis of delirium on length of stay, 6-month mortality, and level of care on discharge at Waitemata District Health Board, Auckland. The New Zealand Medical Journal (Online). 2015;128(1411):68.

224. Tavares JPA, Nunes L, Gracio JCG. Hospitalized older adult: predictors of functional decline. Rev Lat Am Enfermagem. 2021;29:e3399.

225. To-Adithep P, Chittawatanarat K, Mueankwan S, Morakul S, Luetrakool P, Dilokpattanamongkol P, et al. Long-term outcomes of delirium in critically ill surgical patients: A multicenter prospective cohort study. J Psychosom Res. 2023 Sep;172:111427.

226. Traissac T, Videau M-N, Bourdil M-J, Bourdel-Marchasson I, Salles N. The short mean length of stay of post-emergency geriatric units is associated with the rate of early readmission in frail elderly. Aging clinical and experimental research. 2011;23:217-22.

227. Trevisan C, Grande G, Rebora P, Zucchelli A, Valsecchi MG, Ecarnot F, et al. Early onset delirium during hospitalization increases in-hospital and postdischarge mortality in COVID-19 patients: a multicenter prospective study. The Journal of Clinical Psychiatry. 2023;84(5):48598.

228. Tripathy S, Mishra JC, Dash SC. Critically ill elderly patients in a developing world--mortality and functional outcome at 1 year: a prospective single-center study. J Crit Care. 2014 Jun;29(3):474 e7-13.

229. Tsai MC, Weng HH, Chou SY, Tsai CS, Hung TH, Su JA. Three-year mortality of delirium among elderly inpatients in consultation-liaison service. Gen Hosp Psychiatry. 2012 Jan-Feb;34(1):66-71.

230. Uthamalingam S, Gurm GS, Daley M, Flynn J, Capodilupo R. Usefulness of acute delirium as a predictor of adverse outcomes in patients >65 years of age with acute decompensated heart failure. Am J Cardiol. 2011 Aug 1;108(3):402-8.

231. van den Boogaard M, Schoonhoven L, Evers AW, van der Hoeven JG, van Achterberg T, Pickkers P. Delirium in critically ill patients: impact on long-term health-related quality of life and cognitive functioning. Crit Care Med. 2012 Jan;40(1):112-8.

232. van der Heijden EFM, Kooken RWJ, Zegers M, Simons KS, van den Boogaard M. Differences in long-term outcomes between ICU patients with persistent delirium, non-persistent delirium and no delirium: A longitudinal cohort study. J Crit Care. 2023 Aug;76:154277.

233. van der Wulp K, van Wely M, van Heijningen L, van Bakel B, Schoon Y, Verkroost M, et al. Delirium After Transcatheter Aortic Valve Implantation Under General Anesthesia: Incidence, Predictors, and Relation to Long-Term Survival. J Am Geriatr Soc. 2019 Nov;67(11):2325-30.

234. van Eijsden WA, Raats JW, Mulder PG, van der Laan L. New aspects of delirium in elderly patients with critical limb ischemia. Clin Interv Aging. 2015;10:1537-46.

235. van Rijsbergen MW, Oldenbeuving AW, Nieuwenhuis-Mark RE, Nys GM, Las SG, Roks G, et al. Delirium in acute stroke: a predictor of subsequent cognitive impairment? A two-year follow-up study. J Neurol Sci. 2011 Jul 15;306(1-2):138-42.

236. Van Rompaey B, Schuurmans MJ, Shortridge-Baggett LM, Truijen S, Elseviers M, Bossaert L. Long term outcome after delirium in the intensive care unit. J Clin Nurs. 2009 Dec;18(23):3349-57.

237. Vasunilashorn SM, Marcantonio ER, Gou Y, Pisani MA, Travison TG, Schmitt EM, et al. Quantifying the Severity of a Delirium Episode Throughout Hospitalization: the Combined Importance of Intensity and Duration. J Gen Intern Med. 2016 Oct;31(10):1164-71.

238. Vasunilashorn SM, Fong TG, Albuquerque A, Marcantonio ER, Schmitt EM, Tommet D, et al. Delirium Severity Post-Surgery and its Relationship with Long-Term Cognitive Decline in a Cohort of Patients without Dementia. J Alzheimers Dis. 2018;61(1):347-58.

239. Vasunilashorn SM, Fong TG, Helfand BKI, Hshieh TT, Marcantonio ER, Metzger ED, et al. Psychometric Properties of a Delirium Severity Score for Older Adults and Association With Hospital and Posthospital Outcomes. JAMA Netw Open. 2022 Mar 1;5(3):e226129.

240. Veiga D, Luis C, Parente D, Fernandes V, Botelho M, Santos P, et al. Postoperative delirium in intensive care patients: risk factors and outcome. Rev Bras Anestesiol. 2012 Jul;62(4):469-83.

241. Verloo H, Goulet C, Morin D, von Gunten A. Association between frailty and delirium in older adult patients discharged from hospital. Clin Interv Aging. 2016;11:55-63.

242. Vida S, Galbaud du Fort G, Kakuma R, Arsenault L, Platt RW, Wolfson CM. An 18-month prospective cohort study of functional outcome of delirium in elderly patients: activities of daily living. Int Psychogeriatr. 2006 Dec;18(4):681-700.

243. Visser L, Prent A, van der Laan MJ, van Leeuwen BL, Izaks GJ, Zeebregts CJ, et al. Predicting postoperative delirium after vascular surgical procedures. J Vasc Surg. 2015 Jul;62(1):183-9.

244. Vives-Borras M, Martinez-Selles M, Ariza-Sole A, Vidan MT, Formiga F, Bueno H, et al. Clinical and prognostic implications of delirium in elderly patients with non-ST-segment elevation acute coronary syndromes. J Geriatr Cardiol. 2019 Feb;16(2):121-8.

245. Wang Y, Liu W, Chen K, Shen X. Postoperative Delirium is Not Associated with Long-Term Decline in Activities of Daily Living or Mortality After Laryngectomy. Clin Interv Aging. 2021;16:823-31.

246. Weng CF, Lin KP, Lu FP, Chen JH, Wen CJ, Peng JH, et al. Effects of depression, dementia and delirium on activities of daily living in elderly patients after discharge. BMC Geriatr. 2019 Oct 11;19(1):261.

247. Whittamore KH, Goldberg SE, Gladman JR, Bradshaw LE, Jones RG, Harwood RH. The diagnosis, prevalence and outcome of delirium in a cohort of older people with mental health problems on general hospital wards. Int J Geriatr Psychiatry. 2014 Jan;29(1):32-40.

248. Witlox J, Slor CJ, Jansen RW, Kalisvaart KJ, van Stijn MF, Houdijk AP, et al. The neuropsychological sequelae of delirium in elderly patients with hip fracture three months after hospital discharge. Int Psychogeriatr. 2013 Sep;25(9):1521-31.

249. Wolters AE, van Dijk D, Pasma W, Cremer OL, Looije MF, de Lange DW, et al. Long-term outcome of delirium during intensive care unit stay in survivors of critical illness: a prospective cohort study. Critical Care. 2014;18:1-7.

250. Wolters AE, Peelen LM, Veldhuijzen DS, Zaal IJ, de Lange DW, Pasma W, et al. Long-Term Self-Reported Cognitive Problems After Delirium in the Intensive Care Unit and the Effect of Systemic Inflammation. J Am Geriatr Soc. 2017 Apr;65(4):786-91.

251. Zakriya K, Sieber FE, Christmas C, Wenz JF, Sr., Franckowiak S. Brief postoperative delirium in hip fracture patients affects functional outcome at three months. Anesth Analg. 2004 Jun;98(6):1798-802.

252. Ziman N, Sands LP, Tang C, Zhu J, Leung JM. Does postoperative delirium following elective noncardiac surgery predict long-term mortality? Age Ageing. 2020 Oct 23;49(6):1020-7.

253. Zipprich HM, Arends MC, Schumacher U, Bahr V, Scherag A, Kwetkat A, et al. Outcome of Older Patients with Acute Neuropsychological Symptoms Not Fulfilling Criteria of Delirium. J Am Geriatr Soc. 2020 Jul;68(7):1469-75.
